# Supplementary material for: Fitness effects of new mutations in Chlamydomonas reinhardtii across two stress gradients
Source: J Evol Biol. 2016 Jan 5;29(3):583–93. doi: 10.1111/jeb.12807 (PMC4982031; doi:10.1111/jeb.12807)
Supplement: Supplementary file 1 — Figure S1 Schematic showing G × E due to (a) changes in the genetic correlation of growth (the change of the rank order of fitness) and (b) due to changes in the expressed genetic variation. [file JEB-29-583-s001.pdf]

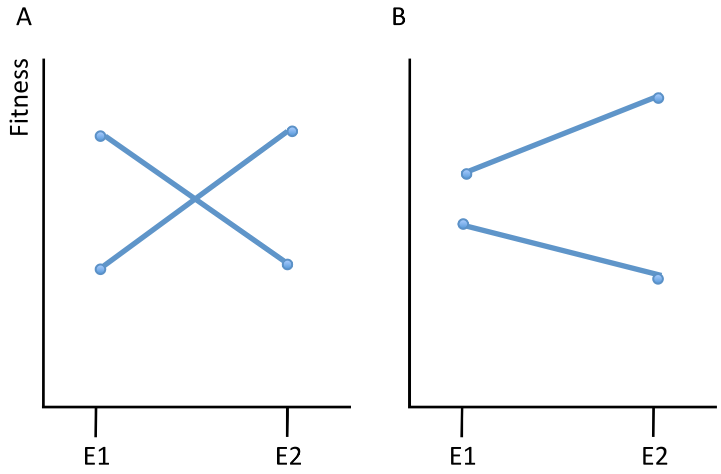

Supp. Fig. 1: Schematic showing GxE due to A) changes in the genetic correlation of growth (the change of the rank order of fitness) and B) due to changes in the expressed genetic variation.
