# Supplementary material for: Fitness effects of new mutations in Chlamydomonas reinhardtii across two stress gradients
Source: J Evol Biol. 2016 Jan 5;29(3):583–93. doi: 10.1111/jeb.12807 (PMC4982031; doi:10.1111/jeb.12807)
Supplement: Supplementary file 2 — Figure S2 Relative fitness of the MA line genotypes, scaled by time, under seven levels of two different stress regimes (0.6–0.001 g L−1 KH2PO4 and 0–6 g L−1 of NaCl). [file JEB-29-583-s002.pdf]

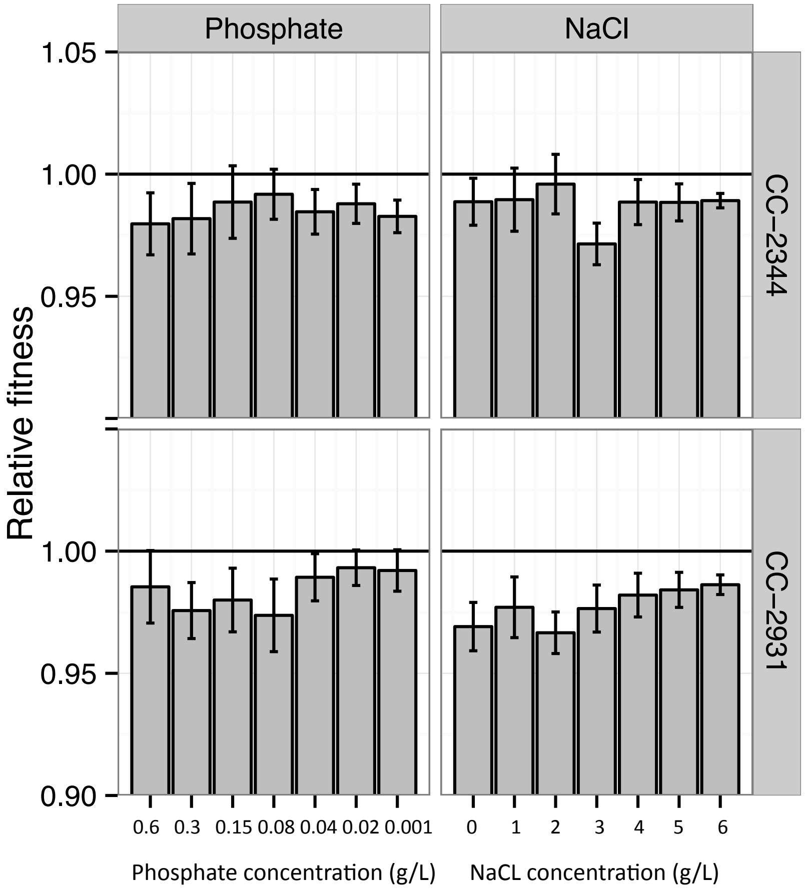

Supplemental Figure 2: Relative fitness of the MA line genotypes, scaled by time, under seven levels of two different stress regimes (0.6 to 0.001gl^{-1} KH_2PO_4 and 0 to 6 gl^{-1} of NaCl). Error bars show 95% confidence intervals.
