# Supplementary material for: Fitness effects of new mutations in Chlamydomonas reinhardtii across two stress gradients
Source: J Evol Biol. 2016 Jan 5;29(3):583–93. doi: 10.1111/jeb.12807 (PMC4982031; doi:10.1111/jeb.12807)
Supplement: Supplementary file 3 — Data S1 Methods. [file JEB-29-583-s003.pdf]

title: **"Deteriorating environments_rel"** output: pdf_document

```
## Loading required package: Matrix
##
## Attaching package: 'lme4'
##
## The following object is masked from 'package:nlme':
##
##     lmList
##
## This is mgcv 1.8-7. For overview type 'help("mgcv-package")'.
##
## Attaching package: 'lmerTest'
##
## The following object is masked from 'package:lme4':
##
##     lmer
##
## The following object is masked from 'package:stats':
##
##     step
```

Description of data sets used

Overall effect of the environment on growth rates

2344_phosphate

```
fit2<-lme(growth~treatment2,random=~1|line/plate,data2344p1)
summary(fit2)
```

```
## Linear mixed-effects model fit by REML
##  Data: data2344p1
##        AIC       BIC   logLik
##   -1292.617 -1255.316 656.3086
##
## Random effects:
##  Formula: ~1 | line
##          (Intercept)
## StdDev: 0.0009808425
##
##  Formula: ~1 | plate %in% line
##         (Intercept)    Residual
## StdDev:  0.02683088 0.006017075
##
## Fixed effects: growth ~ treatment2
##                   Value   Std.Error  DF    t-value p-value
## (Intercept)  0.12266868 0.004106871 294  29.869135  0.0000
## treatment21  0.00652474 0.005796939 294   1.125549  0.2613
```

```
## treatment22 -0.00746538 0.005796939 294  -1.287814  0.1988
## treatment23 -0.01764848 0.005796939 294  -3.044449  0.0025
## treatment24 -0.02627339 0.005796939 294  -4.532287  0.0000
## treatment25 -0.05615219 0.005796939 294  -9.686524  0.0000
## treatment26 -0.06378939 0.005796939 294 -11.003978  0.0000
##  Correlation:
##             (Intr) trtm21 trtm22 trtm23 trtm24 trtm25
## treatment21 -0.706
## treatment22 -0.706  0.500
## treatment23 -0.706  0.500  0.500
## treatment24 -0.706  0.500  0.500  0.500
## treatment25 -0.706  0.500  0.500  0.500  0.500
## treatment26 -0.706  0.500  0.500  0.500  0.500  0.500
##
## Standardized Within-Group Residuals:
##          Min           Q1          Med           Q3          Max
## -0.946430914 -0.128502188 -0.007127313  0.139537263  0.748996487
##
## Number of Observations: 315
## Number of Groups:
##            line plate %in% line
##              15             315
```

```r
anova(fit2)
```

```
##             numDF denDF  F-value p-value
## (Intercept)     1   294 3987.007  <.0001
## treatment2      6   294   44.063  <.0001
```

2931_phosphate

```r
fit2<-lme(growth~treatment2,random=~1|line/plate,data2931p1)
summary(fit2)
```

```
## Linear mixed-effects model fit by REML
##  Data: data2931p1
##         AIC       BIC   logLik
##   -1061.769 -1025.174 540.8845
##
## Random effects:
##  Formula: ~1 | line
##          (Intercept)
## StdDev: 1.232639e-06
##
##  Formula: ~1 | plate %in% line
##         (Intercept)    Residual
## StdDev:  0.03511372 0.000263986
##
## Fixed effects: growth ~ treatment2
##                   Value   Std.Error  DF    t-value p-value
## (Intercept)  0.13621406 0.005418318 274  25.139547  0.0000
```

```
## treatment21 -0.01432626 0.007662659 274  -1.869620  0.0626
## treatment22 -0.01515261 0.007662659 274  -1.977461  0.0490
## treatment23 -0.01814962 0.007662659 274  -2.368580  0.0186
## treatment24 -0.05334480 0.007662659 274  -6.961657  0.0000
## treatment25 -0.09657687 0.007662659 274 -12.603571  0.0000
## treatment26 -0.09599839 0.007662659 274 -12.528077  0.0000
##  Correlation:
##             (Intr) trtm21 trtm22 trtm23 trtm24 trtm25
## treatment21 -0.707
## treatment22 -0.707  0.500
## treatment23 -0.707  0.500  0.500
## treatment24 -0.707  0.500  0.500  0.500
## treatment25 -0.707  0.500  0.500  0.500  0.500
## treatment26 -0.707  0.500  0.500  0.500  0.500  0.500
##
## Standardized Within-Group Residuals:
##           Min            Q1           Med            Q3           Max
## -0.0233243456 -0.0042740231 -0.0004189989  0.0036341903  0.0509052198
##
## Number of Observations: 294
## Number of Groups:
##            line plate %in% line
##              14             294
```

```
anova(fit2)
```

```
##             numDF denDF   F-value p-value
## (Intercept)     1   274 2119.3118  <.0001
## treatment2      6   274   55.8765  <.0001
```

2344_salt

```
fit2<-lme(growth~treatment2,random=~1|line/plate,datan2344s1)
summary(fit2)
```

```
## Linear mixed-effects model fit by REML
##  Data: datan2344s1
##        AIC       BIC   logLik
##   -1257.468 -1220.167 638.7341
##
## Random effects:
##  Formula: ~1 | line
##         (Intercept)
## StdDev: 0.001760467
##
##  Formula: ~1 | plate %in% line
##         (Intercept)    Residual
## StdDev:  0.02797246 0.007950299
##
## Fixed effects: growth ~ treatment2
##                   Value   Std.Error  DF    t-value p-value
```

```
## (Intercept)  0.09542412 0.004358805 294  21.892264  0.0000
## treatment21  0.00087990 0.006130672 294   0.143524  0.8860
## treatment22 -0.01521281 0.006130672 294  -2.481427  0.0136
## treatment23 -0.02269116 0.006130672 294  -3.701252  0.0003
## treatment24 -0.03960709 0.006130672 294  -6.460481  0.0000
## treatment25 -0.05508080 0.006130672 294  -8.984463  0.0000
## treatment26 -0.07794199 0.006130672 294 -12.713450  0.0000
##  Correlation:
##             (Intr) trtm21 trtm22 trtm23 trtm24 trtm25
## treatment21 -0.703
## treatment22 -0.703  0.500
## treatment23 -0.703  0.500  0.500
## treatment24 -0.703  0.500  0.500  0.500
## treatment25 -0.703  0.500  0.500  0.500  0.500
## treatment26 -0.703  0.500  0.500  0.500  0.500  0.500
##
## Standardized Within-Group Residuals:
##          Min           Q1          Med           Q3          Max
## -0.884388603 -0.142621697  0.006073893  0.140529387  1.303301741
##
## Number of Observations: 315
## Number of Groups:
##           line plate %in% line
##             15             315
```

```
anova(fit2)
```

```
##             numDF denDF   F-value p-value
## (Intercept)     1   294 1482.6662  <.0001
## treatment2      6   294   45.6334  <.0001
```

2931__salt

```
fit2<-lme(growth~treatment2,random=~1|line/plate,datan2931s1)
summary(fit2)
```

```
## Linear mixed-effects model fit by REML
##  Data: datan2931s1
##         AIC       BIC   logLik
##   -1174.172 -1137.577 597.0861
##
## Random effects:
##  Formula: ~1 | line
##          (Intercept)
## StdDev: 9.847235e-07
##
##  Formula: ~1 | plate %in% line
##         (Intercept)     Residual
## StdDev:  0.02886901 0.0002144115
##
## Fixed effects: growth ~ treatment2
```

```
##                   Value   Std.Error  DF    t-value p-value
## (Intercept)  0.11586541 0.004454707 274  26.009657  0.0000
## treatment21 -0.01040640 0.006299907 274  -1.651833  0.0997
## treatment22 -0.00640259 0.006299907 274  -1.016299  0.3104
## treatment23 -0.05074798 0.006299907 274  -8.055353  0.0000
## treatment24 -0.05502551 0.006299907 274  -8.734337  0.0000
## treatment25 -0.06839018 0.006299907 274 -10.855743  0.0000
## treatment26 -0.09044634 0.006299907 274 -14.356774  0.0000
##  Correlation:
##             (Intr) trtm21 trtm22 trtm23 trtm24 trtm25
## treatment21 -0.707
## treatment22 -0.707  0.500
## treatment23 -0.707  0.500  0.500
## treatment24 -0.707  0.500  0.500  0.500
## treatment25 -0.707  0.500  0.500  0.500  0.500
## treatment26 -0.707  0.500  0.500  0.500  0.500  0.500
##
## Standardized Within-Group Residuals:
##           Min            Q1           Med            Q3           Max
## -0.0199364681 -0.0043732272 -0.0005586649  0.0035893223  0.0324903617
##
## Number of Observations: 294
## Number of Groups:
##            line plate %in% line
##              14             294
```

```
anova(fit2)
```

```
##             numDF denDF  F-value p-value
## (Intercept)     1   274 2019.405  <.0001
## treatment2      6   274   61.280  <.0001
```

Does stress affect fitness of MA lines and ancestors differently?

2344p

```
fit2<-lme(growth~treatment2*type,random=~1|plate,data2344p)
anova(fit2)
```

```
##                 numDF denDF   F-value p-value
## (Intercept)         1   602 1447.8175  <.0001
## treatment2          6    14   17.7849  <.0001
## type                1   602   33.2883  <.0001
## treatment2:type     6   602    0.4041  0.8764
```

```
summary(fit2)
```

```
## Linear mixed-effects model fit by REML
##  Data: data2344p
##         AIC       BIC   logLik
```

```
##    -2388.178 -2317.406 1210.089
##
## Random effects:
##  Formula: ~1 | plate
##         (Intercept)   Residual
## StdDev: 0.009377508 0.03202968
##
## Fixed effects: growth ~ treatment2 * type
##                         Value   Std.Error  DF   t-value p-value
## (Intercept)         0.12266868 0.007218749 602 16.993066  0.0000
## treatment21         0.00652474 0.010208853  14  0.639126  0.5331
## treatment22        -0.00746538 0.010208853  14 -0.731265  0.4767
## treatment23        -0.01764848 0.010208853  14 -1.728743  0.1058
## treatment24        -0.02627339 0.010208853  14 -2.573589  0.0221
## treatment25        -0.05615219 0.010208853  14 -5.500343  0.0001
## treatment26        -0.06378939 0.010208853  14 -6.248439  0.0000
## typeMA             -0.02034114 0.006752449 602 -3.012409  0.0027
## treatment21:typeMA  0.00211500 0.009549405 602  0.221480  0.8248
## treatment22:typeMA  0.00891206 0.009549405 602  0.933258  0.3511
## treatment23:typeMA  0.01210379 0.009549405 602  1.267492  0.2055
## treatment24:typeMA  0.00492609 0.009549405 602  0.515853  0.6061
## treatment25:typeMA  0.00820960 0.009549405 602  0.859698  0.3903
## treatment26:typeMA  0.00304577 0.009549405 602  0.318948  0.7499
##  Correlation:
##                    (Intr) trtm21 trtm22 trtm23 trtm24 trtm25 trtm26 typeMA
## treatment21        -0.707
## treatment22        -0.707  0.500
## treatment23        -0.707  0.500  0.500
## treatment24        -0.707  0.500  0.500  0.500
## treatment25        -0.707  0.500  0.500  0.500  0.500
## treatment26        -0.707  0.500  0.500  0.500  0.500  0.500
## typeMA             -0.468  0.331  0.331  0.331  0.331  0.331  0.331
## treatment21:typeMA  0.331 -0.468 -0.234 -0.234 -0.234 -0.234 -0.234 -0.707
## treatment22:typeMA  0.331 -0.234 -0.468 -0.234 -0.234 -0.234 -0.234 -0.707
## treatment23:typeMA  0.331 -0.234 -0.234 -0.468 -0.234 -0.234 -0.234 -0.707
## treatment24:typeMA  0.331 -0.234 -0.234 -0.234 -0.468 -0.234 -0.234 -0.707
## treatment25:typeMA  0.331 -0.234 -0.234 -0.234 -0.234 -0.468 -0.234 -0.707
## treatment26:typeMA  0.331 -0.234 -0.234 -0.234 -0.234 -0.234 -0.468 -0.707
##                    t21:MA t22:MA t23:MA t24:MA t25:MA
## treatment21
## treatment22
## treatment23
## treatment24
## treatment25
## treatment26
## typeMA
## treatment21:typeMA
## treatment22:typeMA  0.500
## treatment23:typeMA  0.500  0.500
## treatment24:typeMA  0.500  0.500  0.500
## treatment25:typeMA  0.500  0.500  0.500  0.500
## treatment26:typeMA  0.500  0.500  0.500  0.500  0.500
##
## Standardized Within-Group Residuals:
```

```
##          Min           Q1          Med           Q3          Max 
## -3.696434082 -0.535840634  0.004939777  0.618876429  4.026061800 
## 
## Number of Observations: 630
## Number of Groups: 21
```

2931p

```
fit2<-lme(growth~treatment2*type,random=~1|plate,data2931p)
anova(fit2)
```

```
##                 numDF denDF  F-value p-value
## (Intercept)         1   560 980.0459  <.0001
## treatment2          6    14  26.3060  <.0001
## type                1   560  29.0814  <.0001
## treatment2:type     6   560   1.0293  0.4051
```

```
summary(fit2)
```

```
## Linear mixed-effects model fit by REML
##  Data: data2931p 
##         AIC       BIC   logLik
##   -2100.504 -2030.862 1066.252
## 
## Random effects:
##  Formula: ~1 | plate
##         (Intercept)   Residual
## StdDev:  0.01071473 0.03552412
## 
## Fixed effects: growth ~ treatment2 * type 
##                           Value   Std.Error  DF   t-value p-value
## (Intercept)          0.13621406 0.008265301 560 16.480230  0.0000
## treatment21         -0.01432626 0.011688901  14 -1.225629  0.2406
## treatment22         -0.01515261 0.011688901  14 -1.296325  0.2158
## treatment23         -0.01814962 0.011688901  14 -1.552723  0.1428
## treatment24         -0.05334480 0.011688901  14 -4.563714  0.0004
## treatment25         -0.09657687 0.011688901  14 -8.262271  0.0000
## treatment26         -0.09599839 0.011688901  14 -8.212781  0.0000
## typeMA              -0.01460115 0.007751999 560 -1.883534  0.0601
## treatment21:typeMA -0.00972038 0.010962982 560 -0.886655  0.3756
## treatment22:typeMA -0.00540601 0.010962982 560 -0.493115  0.6221
## treatment23:typeMA -0.01168036 0.010962982 560 -1.065436  0.2871
## treatment24:typeMA  0.00391170 0.010962982 560  0.356810  0.7214
## treatment25:typeMA  0.00781714 0.010962982 560  0.713049  0.4761
## treatment26:typeMA  0.00668202 0.010962982 560  0.609508  0.5424
##  Correlation: 
##                    (Intr) trtm21 trtm22 trtm23 trtm24 trtm25 trtm26 typeMA
## treatment21        -0.707                                                 
## treatment22        -0.707  0.500                                          
## treatment23        -0.707  0.500  0.500                                   
## treatment24        -0.707  0.500  0.500  0.500                            
```

```
## treatment25        -0.707  0.500  0.500  0.500  0.500
## treatment26        -0.707  0.500  0.500  0.500  0.500  0.500
## typeMA             -0.469  0.332  0.332  0.332  0.332  0.332  0.332
## treatment21:typeMA  0.332 -0.469 -0.234 -0.234 -0.234 -0.234 -0.234 -0.707
## treatment22:typeMA  0.332 -0.234 -0.469 -0.234 -0.234 -0.234 -0.234 -0.707
## treatment23:typeMA  0.332 -0.234 -0.234 -0.469 -0.234 -0.234 -0.234 -0.707
## treatment24:typeMA  0.332 -0.234 -0.234 -0.234 -0.469 -0.234 -0.234 -0.707
## treatment25:typeMA  0.332 -0.234 -0.234 -0.234 -0.234 -0.469 -0.234 -0.707
## treatment26:typeMA  0.332 -0.234 -0.234 -0.234 -0.234 -0.234 -0.469 -0.707
##                    t21:MA t22:MA t23:MA t24:MA t25:MA
## treatment21
## treatment22
## treatment23
## treatment24
## treatment25
## treatment26
## typeMA
## treatment21:typeMA
## treatment22:typeMA  0.500
## treatment23:typeMA  0.500  0.500
## treatment24:typeMA  0.500  0.500  0.500
## treatment25:typeMA  0.500  0.500  0.500  0.500
## treatment26:typeMA  0.500  0.500  0.500  0.500  0.500
##
## Standardized Within-Group Residuals:
##        Min         Q1        Med         Q3        Max
## -3.5608423 -0.5214562  0.0518235  0.5084290  6.4154951
##
## Number of Observations: 588
## Number of Groups: 21
```

2344s

```
fit2<-lme(growth~treatment2*type,random=~1|plate,datan2344s)
anova(fit2)
```

```
##                  numDF denDF  F-value p-value
## (Intercept)          1   602 333.9603  <.0001
## treatment2           6    14  11.8543  0.0001
## type                 1   602  31.5764  <.0001
## treatment2:type      6   602   1.6112  0.1415
```

```
summary(fit2)
```

```
## Linear mixed-effects model fit by REML
##  Data: datan2344s
##        AIC       BIC   logLik
##   -2537.228 -2466.456 1284.614
##
## Random effects:
##  Formula: ~1 | plate
```

```
##         (Intercept)   Residual
## StdDev:  0.01392493 0.02810682
##
## Fixed effects: growth ~ treatment2 * type
##                          Value   Std.Error  DF   t-value p-value
## (Intercept)         0.09542412 0.009065869 602 10.525646  0.0000
## treatment21         0.00087990 0.012821074  14  0.068629  0.9463
## treatment22        -0.01521281 0.012821074  14 -1.186547  0.2552
## treatment23        -0.02269116 0.012821074  14 -1.769833  0.0985
## treatment24        -0.03960709 0.012821074  14 -3.089218  0.0080
## treatment25        -0.05508080 0.012821074  14 -4.296114  0.0007
## treatment26        -0.07794199 0.012821074  14 -6.079209  0.0000
## typeMA             -0.01130481 0.005925438 602 -1.907845  0.0569
## treatment21:typeMA  0.00084652 0.008379834 602  0.101019  0.9196
## treatment22:typeMA  0.00721255 0.008379834 602  0.860704  0.3897
## treatment23:typeMA -0.01725935 0.008379834 602 -2.059629  0.0399
## treatment24:typeMA  0.00004023 0.008379834 602  0.004801  0.9962
## treatment25:typeMA -0.00025919 0.008379834 602 -0.030930  0.9753
## treatment26:typeMA  0.00045803 0.008379834 602  0.054658  0.9564
##  Correlation:
##                    (Intr) trtm21 trtm22 trtm23 trtm24 trtm25 trtm26 typeMA
## treatment21        -0.707
## treatment22        -0.707  0.500
## treatment23        -0.707  0.500  0.500
## treatment24        -0.707  0.500  0.500  0.500
## treatment25        -0.707  0.500  0.500  0.500  0.500
## treatment26        -0.707  0.500  0.500  0.500  0.500  0.500
## typeMA             -0.327  0.231  0.231  0.231  0.231  0.231  0.231
## treatment21:typeMA  0.231 -0.327 -0.163 -0.163 -0.163 -0.163 -0.163 -0.707
## treatment22:typeMA  0.231 -0.163 -0.327 -0.163 -0.163 -0.163 -0.163 -0.707
## treatment23:typeMA  0.231 -0.163 -0.163 -0.327 -0.163 -0.163 -0.163 -0.707
## treatment24:typeMA  0.231 -0.163 -0.163 -0.163 -0.327 -0.163 -0.163 -0.707
## treatment25:typeMA  0.231 -0.163 -0.163 -0.163 -0.163 -0.327 -0.163 -0.707
## treatment26:typeMA  0.231 -0.163 -0.163 -0.163 -0.163 -0.163 -0.327 -0.707
##                    t21:MA t22:MA t23:MA t24:MA t25:MA
## treatment21
## treatment22
## treatment23
## treatment24
## treatment25
## treatment26
## typeMA
## treatment21:typeMA
## treatment22:typeMA  0.500
## treatment23:typeMA  0.500  0.500
## treatment24:typeMA  0.500  0.500  0.500
## treatment25:typeMA  0.500  0.500  0.500  0.500
## treatment26:typeMA  0.500  0.500  0.500  0.500  0.500
##
## Standardized Within-Group Residuals:
##         Min          Q1         Med          Q3         Max
## -2.84982072 -0.54067456 -0.05630079  0.51590725  4.88151993
##
## Number of Observations: 630
```

```
## Number of Groups: 21
```

2931s

```r
fit2<-lme(growth~treatment2*type,random=~1|plate,datan2931s)
anova(fit2)
```

```
##                 numDF denDF   F-value p-value
## (Intercept)         1   560 1255.5462  <.0001
## treatment2          6    14   43.1671  <.0001
## type                1   560   96.8585  <.0001
## treatment2:type     6   560    1.4908   0.179
```

```r
summary(fit2)
```

```
## Linear mixed-effects model fit by REML
##  Data: datan2931s
##         AIC       BIC   logLik
##   -2382.915 -2313.273 1207.458
##
## Random effects:
##  Formula: ~1 | plate
##         (Intercept)   Residual
## StdDev: 0.006437774 0.02789701
##
## Fixed effects: growth ~ treatment2 * type
##                           Value   Std.Error  DF    t-value p-value
## (Intercept)          0.11586541 0.005687230 560  20.372908  0.0000
## treatment21         -0.01040640 0.008042957  14  -1.293852  0.2167
## treatment22         -0.00640259 0.008042957  14  -0.796049  0.4393
## treatment23         -0.05074798 0.008042957  14  -6.309616  0.0000
## treatment24         -0.05502551 0.008042957  14  -6.841453  0.0000
## treatment25         -0.06839018 0.008042957  14  -8.503113  0.0000
## treatment26         -0.09044634 0.008042957  14 -11.245409  0.0000
## typeMA              -0.03088402 0.006087627 560  -5.073244  0.0000
## treatment21:typeMA   0.00790398 0.008609205 560   0.918085  0.3590
## treatment22:typeMA  -0.00251471 0.008609205 560  -0.292096  0.7703
## treatment23:typeMA   0.00724063 0.008609205 560   0.841033  0.4007
## treatment24:typeMA   0.01287264 0.008609205 560   1.495218  0.1354
## treatment25:typeMA   0.01503182 0.008609205 560   1.746017  0.0814
## treatment26:typeMA   0.01714038 0.008609205 560   1.990936  0.0470
##  Correlation:
##                     (Intr) trtm21 trtm22 trtm23 trtm24 trtm25 trtm26 typeMA
## treatment21         -0.707
## treatment22         -0.707  0.500
## treatment23         -0.707  0.500  0.500
## treatment24         -0.707  0.500  0.500  0.500
## treatment25         -0.707  0.500  0.500  0.500  0.500
## treatment26         -0.707  0.500  0.500  0.500  0.500  0.500
## typeMA              -0.535  0.378  0.378  0.378  0.378  0.378  0.378
## treatment21:typeMA   0.378 -0.535 -0.268 -0.268 -0.268 -0.268 -0.268 -0.707
```

```
## treatment22:typeMA  0.378 -0.268 -0.535 -0.268 -0.268 -0.268 -0.268 -0.707
## treatment23:typeMA  0.378 -0.268 -0.268 -0.535 -0.268 -0.268 -0.268 -0.707
## treatment24:typeMA  0.378 -0.268 -0.268 -0.268 -0.535 -0.268 -0.268 -0.707
## treatment25:typeMA  0.378 -0.268 -0.268 -0.268 -0.268 -0.535 -0.268 -0.707
## treatment26:typeMA  0.378 -0.268 -0.268 -0.268 -0.268 -0.268 -0.535 -0.707
##                    t21:MA t22:MA t23:MA t24:MA t25:MA
## treatment21
## treatment22
## treatment23
## treatment24
## treatment25
## treatment26
## typeMA
## treatment21:typeMA
## treatment22:typeMA  0.500
## treatment23:typeMA  0.500  0.500
## treatment24:typeMA  0.500  0.500  0.500
## treatment25:typeMA  0.500  0.500  0.500  0.500
## treatment26:typeMA  0.500  0.500  0.500  0.500  0.500
##
## Standardized Within-Group Residuals:
##         Min          Q1         Med          Q3         Max
## -2.72706030 -0.56941331 -0.05115317  0.54205207  4.50938760
##
## Number of Observations: 588
## Number of Groups: 21
```

Does the environment affect the relative fitness (scaled by generations)

2344p

```
data<-read.table("~/Desktop/rsync/deteriorating treatments/phosphate/2344_phosphate_v2_[0].csv",h=T)
data$treatment2<-as.factor(data$treatment)
data$pair<-as.factor(data$pair)
fit2<-lme(relfitav~treatment2*pair,random=~1|plate,data)
anova(fit2)
```

```
##                  numDF denDF   F-value p-value
## (Intercept)          1   196 2187.9676  <.0001
## treatment2           6    14    1.1138  0.4027
## pair                14   196    4.1446  <.0001
## treatment2:pair     84   196    1.2061  0.1467
```

```
summary(fit2)
```

```
## Linear mixed-effects model fit by REML
##  Data: data
##       AIC      BIC    logLik
##   314.508 672.6485 -50.25401
##
## Random effects:
```

```
##  Formula: ~1 | plate
##         (Intercept)  Residual
## StdDev:  0.06462241 0.2274718
##
## Fixed effects: relfitav ~ treatment2 * pair
##                          Value Std.Error  DF   t-value p-value
## (Intercept)          0.8290761 0.1365277 196  6.072583  0.0000
## treatment21         -0.1777987 0.1930794  14 -0.920858  0.3727
## treatment22         -0.1723476 0.1930794  14 -0.892625  0.3871
## treatment23          0.0053831 0.1930794  14  0.027880  0.9782
## treatment24         -0.1074132 0.1930794  14 -0.556316  0.5868
## treatment25         -0.0714102 0.1930794  14 -0.369849  0.7170
## treatment26         -0.0169481 0.1930794  14 -0.087778  0.9313
## pair2               -0.1345965 0.1857300 196 -0.724689  0.4695
## pair3                0.1241829 0.1857300 196  0.668621  0.5045
## pair4               -0.1976025 0.1857300 196 -1.063924  0.2887
## pair5                0.0324047 0.1857300 196  0.174472  0.8617
## pair6                0.2772789 0.1857300 196  1.492914  0.1371
## pair7                0.0731467 0.1857300 196  0.393834  0.6941
## pair8                0.2984404 0.1857300 196  1.606851  0.1097
## pair9               -0.1151588 0.1857300 196 -0.620033  0.5360
## pair10              -0.0102776 0.1857300 196 -0.055336  0.9559
## pair11               0.0167273 0.1857300 196  0.090063  0.9283
## pair12              -0.0491906 0.1857300 196 -0.264850  0.7914
## pair13               0.1713502 0.1857300 196  0.922577  0.3574
## pair14               0.3313624 0.1857300 196  1.784108  0.0760
## pair15               0.0086106 0.1857300 196  0.046361  0.9631
## treatment21:pair2    0.4854478 0.2626618 196  1.848186  0.0661
## treatment22:pair2    0.6774010 0.2626618 196  2.578986  0.0106
## treatment23:pair2    0.4791950 0.2626618 196  1.824380  0.0696
## treatment24:pair2    0.3528814 0.2626618 196  1.343482  0.1807
## treatment25:pair2    0.3440389 0.2626618 196  1.309817  0.1918
## treatment26:pair2    0.0739557 0.2626618 196  0.281562  0.7786
## treatment21:pair3   -0.0723743 0.2626618 196 -0.275542  0.7832
## treatment22:pair3   -0.1675335 0.2626618 196 -0.637830  0.5243
## treatment23:pair3   -0.1438302 0.2626618 196 -0.547587  0.5846
## treatment24:pair3    0.0324939 0.2626618 196  0.123710  0.9017
## treatment25:pair3    0.3579712 0.2626618 196  1.362860  0.1745
## treatment26:pair3   -0.3205804 0.2626618 196 -1.220506  0.2237
## treatment21:pair4    0.4172476 0.2626618 196  1.588536  0.1138
## treatment22:pair4    0.5473828 0.2626618 196  2.083983  0.0385
## treatment23:pair4    0.0959892 0.2626618 196  0.365448  0.7152
## treatment24:pair4    0.2544920 0.2626618 196  0.968896  0.3338
## treatment25:pair4    0.0616179 0.2626618 196  0.234590  0.8148
## treatment26:pair4    0.4071621 0.2626618 196  1.550138  0.1227
## treatment21:pair5    0.2066163 0.2626618 196  0.786625  0.4325
## treatment22:pair5    0.5656135 0.2626618 196  2.153391  0.0325
## treatment23:pair5   -0.0689985 0.2626618 196 -0.262690  0.7931
## treatment24:pair5    0.3401833 0.2626618 196  1.295138  0.1968
## treatment25:pair5    0.2500043 0.2626618 196  0.951810  0.3424
## treatment26:pair5    0.0473568 0.2626618 196  0.180296  0.8571
## treatment21:pair6    0.0117762 0.2626618 196  0.044834  0.9643
## treatment22:pair6    0.0016438 0.2626618 196  0.006258  0.9950
## treatment23:pair6   -0.1176835 0.2626618 196 -0.448042  0.6546
```

```
## treatment24:pair6  -0.0714566 0.2626618 196 -0.272048  0.7859
## treatment25:pair6  -0.1686279 0.2626618 196 -0.641996  0.5216
## treatment26:pair6  -0.3037683 0.2626618 196 -1.156500  0.2489
## treatment21:pair7   0.0654544 0.2626618 196  0.249197  0.8035
## treatment22:pair7   0.4155195 0.2626618 196  1.581956  0.1153
## treatment23:pair7   0.2145697 0.2626618 196  0.816905  0.4150
## treatment24:pair7   0.2402146 0.2626618 196  0.914540  0.3616
## treatment25:pair7   0.1386486 0.2626618 196  0.527860  0.5982
## treatment26:pair7   0.1636456 0.2626618 196  0.623028  0.5340
## treatment21:pair8   0.1712968 0.2626618 196  0.652157  0.5151
## treatment22:pair8   0.0200064 0.2626618 196  0.076168  0.9394
## treatment23:pair8  -0.1303408 0.2626618 196 -0.496230  0.6203
## treatment24:pair8   0.0329648 0.2626618 196  0.125503  0.9003
## treatment25:pair8  -0.2924001 0.2626618 196 -1.113219  0.2670
## treatment26:pair8  -0.1291016 0.2626618 196 -0.491513  0.6236
## treatment21:pair9   0.3559054 0.2626618 196  1.354995  0.1770
## treatment22:pair9   0.1903330 0.2626618 196  0.724631  0.4695
## treatment23:pair9  -0.1637510 0.2626618 196 -0.623429  0.5337
## treatment24:pair9   0.1692066 0.2626618 196  0.644200  0.5202
## treatment25:pair9   0.1578626 0.2626618 196  0.601011  0.5485
## treatment26:pair9  -0.1673545 0.2626618 196 -0.637148  0.5248
## treatment21:pair10  0.3250994 0.2626618 196  1.237711  0.2173
## treatment22:pair10  0.2674842 0.2626618 196  1.018360  0.3098
## treatment23:pair10  0.1441656 0.2626618 196  0.548864  0.5837
## treatment24:pair10  0.3094658 0.2626618 196  1.178191  0.2401
## treatment25:pair10  0.1617544 0.2626618 196  0.615828  0.5387
## treatment26:pair10 -0.2100328 0.2626618 196 -0.799632  0.4249
## treatment21:pair11  0.2680133 0.2626618 196  1.020374  0.3088
## treatment22:pair11 -0.0236699 0.2626618 196 -0.090116  0.9283
## treatment23:pair11  0.0099849 0.2626618 196  0.038014  0.9697
## treatment24:pair11 -0.0393783 0.2626618 196 -0.149920  0.8810
## treatment25:pair11 -0.0578751 0.2626618 196 -0.220341  0.8258
## treatment26:pair11  0.0399933 0.2626618 196  0.152262  0.8791
## treatment21:pair12  0.3857899 0.2626618 196  1.468771  0.1435
## treatment22:pair12  0.3909126 0.2626618 196  1.488273  0.1383
## treatment23:pair12  0.1246814 0.2626618 196  0.474684  0.6355
## treatment24:pair12 -0.0620420 0.2626618 196 -0.236205  0.8135
## treatment25:pair12 -0.0419270 0.2626618 196 -0.159624  0.8733
## treatment26:pair12 -0.2409662 0.2626618 196 -0.917401  0.3601
## treatment21:pair13  0.0801504 0.2626618 196  0.305147  0.7606
## treatment22:pair13  0.1588428 0.2626618 196  0.604743  0.5460
## treatment23:pair13  0.0858660 0.2626618 196  0.326907  0.7441
## treatment24:pair13  0.2220126 0.2626618 196  0.845241  0.3990
## treatment25:pair13  0.0221283 0.2626618 196  0.084246  0.9329
## treatment26:pair13 -0.1520454 0.2626618 196 -0.578864  0.5633
## treatment21:pair14  0.0670417 0.2626618 196  0.255240  0.7988
## treatment22:pair14 -0.0542597 0.2626618 196 -0.206576  0.8366
## treatment23:pair14 -0.0192114 0.2626618 196 -0.073141  0.9418
## treatment24:pair14 -0.1754902 0.2626618 196 -0.668122  0.5048
## treatment25:pair14  0.0505239 0.2626618 196  0.192353  0.8477
## treatment26:pair14 -0.4836283 0.2626618 196 -1.841259  0.0671
## treatment21:pair15  0.1690127 0.2626618 196  0.643461  0.5207
## treatment22:pair15  0.4261393 0.2626618 196  1.622388  0.1063
## treatment23:pair15  0.3356728 0.2626618 196  1.277966  0.2028
```

```
## treatment24:pair15  0.1955001 0.2626618 196  0.744304  0.4576
## treatment25:pair15  0.1202987 0.2626618 196  0.457998  0.6475
## treatment26:pair15  0.0317279 0.2626618 196  0.120794  0.9040
##  Correlation:
##                     (Intr) trtm21 trtm22 trtm23 trtm24 trtm25 trtm26 pair2
## treatment21         -0.707
## treatment22         -0.707  0.500
## treatment23         -0.707  0.500  0.500
## treatment24         -0.707  0.500  0.500  0.500
## treatment25         -0.707  0.500  0.500  0.500  0.500
## treatment26         -0.707  0.500  0.500  0.500  0.500  0.500
## pair2               -0.680  0.481  0.481  0.481  0.481  0.481  0.481
## pair3               -0.680  0.481  0.481  0.481  0.481  0.481  0.481  0.500
## pair4               -0.680  0.481  0.481  0.481  0.481  0.481  0.481  0.500
## pair5               -0.680  0.481  0.481  0.481  0.481  0.481  0.481  0.500
## pair6               -0.680  0.481  0.481  0.481  0.481  0.481  0.481  0.500
## pair7               -0.680  0.481  0.481  0.481  0.481  0.481  0.481  0.500
## pair8               -0.680  0.481  0.481  0.481  0.481  0.481  0.481  0.500
## pair9               -0.680  0.481  0.481  0.481  0.481  0.481  0.481  0.500
## pair10              -0.680  0.481  0.481  0.481  0.481  0.481  0.481  0.500
## pair11              -0.680  0.481  0.481  0.481  0.481  0.481  0.481  0.500
## pair12              -0.680  0.481  0.481  0.481  0.481  0.481  0.481  0.500
## pair13              -0.680  0.481  0.481  0.481  0.481  0.481  0.481  0.500
## pair14              -0.680  0.481  0.481  0.481  0.481  0.481  0.481  0.500
## pair15              -0.680  0.481  0.481  0.481  0.481  0.481  0.481  0.500
## treatment21:pair2    0.481 -0.680 -0.340 -0.340 -0.340 -0.340 -0.340 -0.707
## treatment22:pair2    0.481 -0.340 -0.680 -0.340 -0.340 -0.340 -0.340 -0.707
## treatment23:pair2    0.481 -0.340 -0.340 -0.680 -0.340 -0.340 -0.340 -0.707
## treatment24:pair2    0.481 -0.340 -0.340 -0.340 -0.680 -0.340 -0.340 -0.707
## treatment25:pair2    0.481 -0.340 -0.340 -0.340 -0.340 -0.680 -0.340 -0.707
## treatment26:pair2    0.481 -0.340 -0.340 -0.340 -0.340 -0.340 -0.680 -0.707
## treatment21:pair3    0.481 -0.680 -0.340 -0.340 -0.340 -0.340 -0.340 -0.354
## treatment22:pair3    0.481 -0.340 -0.680 -0.340 -0.340 -0.340 -0.340 -0.354
## treatment23:pair3    0.481 -0.340 -0.340 -0.680 -0.340 -0.340 -0.340 -0.354
## treatment24:pair3    0.481 -0.340 -0.340 -0.340 -0.680 -0.340 -0.340 -0.354
## treatment25:pair3    0.481 -0.340 -0.340 -0.340 -0.340 -0.680 -0.340 -0.354
## treatment26:pair3    0.481 -0.340 -0.340 -0.340 -0.340 -0.340 -0.680 -0.354
## treatment21:pair4    0.481 -0.680 -0.340 -0.340 -0.340 -0.340 -0.340 -0.354
## treatment22:pair4    0.481 -0.340 -0.680 -0.340 -0.340 -0.340 -0.340 -0.354
## treatment23:pair4    0.481 -0.340 -0.340 -0.680 -0.340 -0.340 -0.340 -0.354
## treatment24:pair4    0.481 -0.340 -0.340 -0.340 -0.680 -0.340 -0.340 -0.354
## treatment25:pair4    0.481 -0.340 -0.340 -0.340 -0.340 -0.680 -0.340 -0.354
## treatment26:pair4    0.481 -0.340 -0.340 -0.340 -0.340 -0.340 -0.680 -0.354
## treatment21:pair5    0.481 -0.680 -0.340 -0.340 -0.340 -0.340 -0.340 -0.354
## treatment22:pair5    0.481 -0.340 -0.680 -0.340 -0.340 -0.340 -0.340 -0.354
## treatment23:pair5    0.481 -0.340 -0.340 -0.680 -0.340 -0.340 -0.340 -0.354
## treatment24:pair5    0.481 -0.340 -0.340 -0.340 -0.680 -0.340 -0.340 -0.354
## treatment25:pair5    0.481 -0.340 -0.340 -0.340 -0.340 -0.680 -0.340 -0.354
## treatment26:pair5    0.481 -0.340 -0.340 -0.340 -0.340 -0.340 -0.680 -0.354
## treatment21:pair6    0.481 -0.680 -0.340 -0.340 -0.340 -0.340 -0.340 -0.354
## treatment22:pair6    0.481 -0.340 -0.680 -0.340 -0.340 -0.340 -0.340 -0.354
## treatment23:pair6    0.481 -0.340 -0.340 -0.680 -0.340 -0.340 -0.340 -0.354
## treatment24:pair6    0.481 -0.340 -0.340 -0.340 -0.680 -0.340 -0.340 -0.354
## treatment25:pair6    0.481 -0.340 -0.340 -0.340 -0.340 -0.680 -0.340 -0.354
```

```
## treatment26:pair6   0.481 -0.340 -0.340 -0.340 -0.340 -0.340 -0.680 -0.354
## treatment21:pair7   0.481 -0.680 -0.340 -0.340 -0.340 -0.340 -0.340 -0.354
## treatment22:pair7   0.481 -0.340 -0.680 -0.340 -0.340 -0.340 -0.340 -0.354
## treatment23:pair7   0.481 -0.340 -0.340 -0.680 -0.340 -0.340 -0.340 -0.354
## treatment24:pair7   0.481 -0.340 -0.340 -0.340 -0.680 -0.340 -0.340 -0.354
## treatment25:pair7   0.481 -0.340 -0.340 -0.340 -0.340 -0.680 -0.340 -0.354
## treatment26:pair7   0.481 -0.340 -0.340 -0.340 -0.340 -0.340 -0.680 -0.354
## treatment21:pair8   0.481 -0.680 -0.340 -0.340 -0.340 -0.340 -0.340 -0.354
## treatment22:pair8   0.481 -0.340 -0.680 -0.340 -0.340 -0.340 -0.340 -0.354
## treatment23:pair8   0.481 -0.340 -0.340 -0.680 -0.340 -0.340 -0.340 -0.354
## treatment24:pair8   0.481 -0.340 -0.340 -0.340 -0.680 -0.340 -0.340 -0.354
## treatment25:pair8   0.481 -0.340 -0.340 -0.340 -0.340 -0.680 -0.340 -0.354
## treatment26:pair8   0.481 -0.340 -0.340 -0.340 -0.340 -0.340 -0.680 -0.354
## treatment21:pair9   0.481 -0.680 -0.340 -0.340 -0.340 -0.340 -0.340 -0.354
## treatment22:pair9   0.481 -0.340 -0.680 -0.340 -0.340 -0.340 -0.340 -0.354
## treatment23:pair9   0.481 -0.340 -0.340 -0.680 -0.340 -0.340 -0.340 -0.354
## treatment24:pair9   0.481 -0.340 -0.340 -0.340 -0.680 -0.340 -0.340 -0.354
## treatment25:pair9   0.481 -0.340 -0.340 -0.340 -0.340 -0.680 -0.340 -0.354
## treatment26:pair9   0.481 -0.340 -0.340 -0.340 -0.340 -0.340 -0.680 -0.354
## treatment21:pair10  0.481 -0.680 -0.340 -0.340 -0.340 -0.340 -0.340 -0.354
## treatment22:pair10  0.481 -0.340 -0.680 -0.340 -0.340 -0.340 -0.340 -0.354
## treatment23:pair10  0.481 -0.340 -0.340 -0.680 -0.340 -0.340 -0.340 -0.354
## treatment24:pair10  0.481 -0.340 -0.340 -0.340 -0.680 -0.340 -0.340 -0.354
## treatment25:pair10  0.481 -0.340 -0.340 -0.340 -0.340 -0.680 -0.340 -0.354
## treatment26:pair10  0.481 -0.340 -0.340 -0.340 -0.340 -0.340 -0.680 -0.354
## treatment21:pair11  0.481 -0.680 -0.340 -0.340 -0.340 -0.340 -0.340 -0.354
## treatment22:pair11  0.481 -0.340 -0.680 -0.340 -0.340 -0.340 -0.340 -0.354
## treatment23:pair11  0.481 -0.340 -0.340 -0.680 -0.340 -0.340 -0.340 -0.354
## treatment24:pair11  0.481 -0.340 -0.340 -0.340 -0.680 -0.340 -0.340 -0.354
## treatment25:pair11  0.481 -0.340 -0.340 -0.340 -0.340 -0.680 -0.340 -0.354
## treatment26:pair11  0.481 -0.340 -0.340 -0.340 -0.340 -0.340 -0.680 -0.354
## treatment21:pair12  0.481 -0.680 -0.340 -0.340 -0.340 -0.340 -0.340 -0.354
## treatment22:pair12  0.481 -0.340 -0.680 -0.340 -0.340 -0.340 -0.340 -0.354
## treatment23:pair12  0.481 -0.340 -0.340 -0.680 -0.340 -0.340 -0.340 -0.354
## treatment24:pair12  0.481 -0.340 -0.340 -0.340 -0.680 -0.340 -0.340 -0.354
## treatment25:pair12  0.481 -0.340 -0.340 -0.340 -0.340 -0.680 -0.340 -0.354
## treatment26:pair12  0.481 -0.340 -0.340 -0.340 -0.340 -0.340 -0.680 -0.354
## treatment21:pair13  0.481 -0.680 -0.340 -0.340 -0.340 -0.340 -0.340 -0.354
## treatment22:pair13  0.481 -0.340 -0.680 -0.340 -0.340 -0.340 -0.340 -0.354
## treatment23:pair13  0.481 -0.340 -0.340 -0.680 -0.340 -0.340 -0.340 -0.354
## treatment24:pair13  0.481 -0.340 -0.340 -0.340 -0.680 -0.340 -0.340 -0.354
## treatment25:pair13  0.481 -0.340 -0.340 -0.340 -0.340 -0.680 -0.340 -0.354
## treatment26:pair13  0.481 -0.340 -0.340 -0.340 -0.340 -0.340 -0.680 -0.354
## treatment21:pair14  0.481 -0.680 -0.340 -0.340 -0.340 -0.340 -0.340 -0.354
## treatment22:pair14  0.481 -0.340 -0.680 -0.340 -0.340 -0.340 -0.340 -0.354
## treatment23:pair14  0.481 -0.340 -0.340 -0.680 -0.340 -0.340 -0.340 -0.354
## treatment24:pair14  0.481 -0.340 -0.340 -0.340 -0.680 -0.340 -0.340 -0.354
## treatment25:pair14  0.481 -0.340 -0.340 -0.340 -0.340 -0.680 -0.340 -0.354
## treatment26:pair14  0.481 -0.340 -0.340 -0.340 -0.340 -0.340 -0.680 -0.354
## treatment21:pair15  0.481 -0.680 -0.340 -0.340 -0.340 -0.340 -0.340 -0.354
## treatment22:pair15  0.481 -0.340 -0.680 -0.340 -0.340 -0.340 -0.340 -0.354
## treatment23:pair15  0.481 -0.340 -0.340 -0.680 -0.340 -0.340 -0.340 -0.354
## treatment24:pair15  0.481 -0.340 -0.340 -0.340 -0.680 -0.340 -0.340 -0.354
## treatment25:pair15  0.481 -0.340 -0.340 -0.340 -0.340 -0.680 -0.340 -0.354
```

```
## treatment26:pair15  0.481 -0.340 -0.340 -0.340 -0.340 -0.340 -0.680 -0.354
##                     pair3  pair4  pair5  pair6  pair7  pair8  pair9  pair10
## treatment21
## treatment22
## treatment23
## treatment24
## treatment25
## treatment26
## pair2
## pair3
## pair4                0.500
## pair5                0.500  0.500
## pair6                0.500  0.500  0.500
## pair7                0.500  0.500  0.500  0.500
## pair8                0.500  0.500  0.500  0.500  0.500
## pair9                0.500  0.500  0.500  0.500  0.500  0.500
## pair10               0.500  0.500  0.500  0.500  0.500  0.500  0.500
## pair11               0.500  0.500  0.500  0.500  0.500  0.500  0.500  0.500
## pair12               0.500  0.500  0.500  0.500  0.500  0.500  0.500  0.500
## pair13               0.500  0.500  0.500  0.500  0.500  0.500  0.500  0.500
## pair14               0.500  0.500  0.500  0.500  0.500  0.500  0.500  0.500
## pair15               0.500  0.500  0.500  0.500  0.500  0.500  0.500  0.500
## treatment21:pair2   -0.354 -0.354 -0.354 -0.354 -0.354 -0.354 -0.354 -0.354
## treatment22:pair2   -0.354 -0.354 -0.354 -0.354 -0.354 -0.354 -0.354 -0.354
## treatment23:pair2   -0.354 -0.354 -0.354 -0.354 -0.354 -0.354 -0.354 -0.354
## treatment24:pair2   -0.354 -0.354 -0.354 -0.354 -0.354 -0.354 -0.354 -0.354
## treatment25:pair2   -0.354 -0.354 -0.354 -0.354 -0.354 -0.354 -0.354 -0.354
## treatment26:pair2   -0.354 -0.354 -0.354 -0.354 -0.354 -0.354 -0.354 -0.354
## treatment21:pair3   -0.707 -0.354 -0.354 -0.354 -0.354 -0.354 -0.354 -0.354
## treatment22:pair3   -0.707 -0.354 -0.354 -0.354 -0.354 -0.354 -0.354 -0.354
## treatment23:pair3   -0.707 -0.354 -0.354 -0.354 -0.354 -0.354 -0.354 -0.354
## treatment24:pair3   -0.707 -0.354 -0.354 -0.354 -0.354 -0.354 -0.354 -0.354
## treatment25:pair3   -0.707 -0.354 -0.354 -0.354 -0.354 -0.354 -0.354 -0.354
## treatment26:pair3   -0.707 -0.354 -0.354 -0.354 -0.354 -0.354 -0.354 -0.354
## treatment21:pair4   -0.354 -0.707 -0.354 -0.354 -0.354 -0.354 -0.354 -0.354
## treatment22:pair4   -0.354 -0.707 -0.354 -0.354 -0.354 -0.354 -0.354 -0.354
## treatment23:pair4   -0.354 -0.707 -0.354 -0.354 -0.354 -0.354 -0.354 -0.354
## treatment24:pair4   -0.354 -0.707 -0.354 -0.354 -0.354 -0.354 -0.354 -0.354
## treatment25:pair4   -0.354 -0.707 -0.354 -0.354 -0.354 -0.354 -0.354 -0.354
## treatment26:pair4   -0.354 -0.707 -0.354 -0.354 -0.354 -0.354 -0.354 -0.354
## treatment21:pair5   -0.354 -0.354 -0.707 -0.354 -0.354 -0.354 -0.354 -0.354
## treatment22:pair5   -0.354 -0.354 -0.707 -0.354 -0.354 -0.354 -0.354 -0.354
## treatment23:pair5   -0.354 -0.354 -0.707 -0.354 -0.354 -0.354 -0.354 -0.354
## treatment24:pair5   -0.354 -0.354 -0.707 -0.354 -0.354 -0.354 -0.354 -0.354
## treatment25:pair5   -0.354 -0.354 -0.707 -0.354 -0.354 -0.354 -0.354 -0.354
## treatment26:pair5   -0.354 -0.354 -0.707 -0.354 -0.354 -0.354 -0.354 -0.354
## treatment21:pair6   -0.354 -0.354 -0.354 -0.707 -0.354 -0.354 -0.354 -0.354
## treatment22:pair6   -0.354 -0.354 -0.354 -0.707 -0.354 -0.354 -0.354 -0.354
## treatment23:pair6   -0.354 -0.354 -0.354 -0.707 -0.354 -0.354 -0.354 -0.354
## treatment24:pair6   -0.354 -0.354 -0.354 -0.707 -0.354 -0.354 -0.354 -0.354
## treatment25:pair6   -0.354 -0.354 -0.354 -0.707 -0.354 -0.354 -0.354 -0.354
## treatment26:pair6   -0.354 -0.354 -0.354 -0.707 -0.354 -0.354 -0.354 -0.354
## treatment21:pair7   -0.354 -0.354 -0.354 -0.354 -0.707 -0.354 -0.354 -0.354
## treatment22:pair7   -0.354 -0.354 -0.354 -0.354 -0.707 -0.354 -0.354 -0.354
```

```
## treatment23:pair7  -0.354 -0.354 -0.354 -0.354 -0.707 -0.354 -0.354 -0.354
## treatment24:pair7  -0.354 -0.354 -0.354 -0.354 -0.707 -0.354 -0.354 -0.354
## treatment25:pair7  -0.354 -0.354 -0.354 -0.354 -0.707 -0.354 -0.354 -0.354
## treatment26:pair7  -0.354 -0.354 -0.354 -0.354 -0.707 -0.354 -0.354 -0.354
## treatment21:pair8  -0.354 -0.354 -0.354 -0.354 -0.354 -0.707 -0.354 -0.354
## treatment22:pair8  -0.354 -0.354 -0.354 -0.354 -0.354 -0.707 -0.354 -0.354
## treatment23:pair8  -0.354 -0.354 -0.354 -0.354 -0.354 -0.707 -0.354 -0.354
## treatment24:pair8  -0.354 -0.354 -0.354 -0.354 -0.354 -0.707 -0.354 -0.354
## treatment25:pair8  -0.354 -0.354 -0.354 -0.354 -0.354 -0.707 -0.354 -0.354
## treatment26:pair8  -0.354 -0.354 -0.354 -0.354 -0.354 -0.707 -0.354 -0.354
## treatment21:pair9  -0.354 -0.354 -0.354 -0.354 -0.354 -0.354 -0.707 -0.354
## treatment22:pair9  -0.354 -0.354 -0.354 -0.354 -0.354 -0.354 -0.707 -0.354
## treatment23:pair9  -0.354 -0.354 -0.354 -0.354 -0.354 -0.354 -0.707 -0.354
## treatment24:pair9  -0.354 -0.354 -0.354 -0.354 -0.354 -0.354 -0.707 -0.354
## treatment25:pair9  -0.354 -0.354 -0.354 -0.354 -0.354 -0.354 -0.707 -0.354
## treatment26:pair9  -0.354 -0.354 -0.354 -0.354 -0.354 -0.354 -0.707 -0.354
## treatment21:pair10 -0.354 -0.354 -0.354 -0.354 -0.354 -0.354 -0.354 -0.707
## treatment22:pair10 -0.354 -0.354 -0.354 -0.354 -0.354 -0.354 -0.354 -0.707
## treatment23:pair10 -0.354 -0.354 -0.354 -0.354 -0.354 -0.354 -0.354 -0.707
## treatment24:pair10 -0.354 -0.354 -0.354 -0.354 -0.354 -0.354 -0.354 -0.707
## treatment25:pair10 -0.354 -0.354 -0.354 -0.354 -0.354 -0.354 -0.354 -0.707
## treatment26:pair10 -0.354 -0.354 -0.354 -0.354 -0.354 -0.354 -0.354 -0.707
## treatment21:pair11 -0.354 -0.354 -0.354 -0.354 -0.354 -0.354 -0.354 -0.354
## treatment22:pair11 -0.354 -0.354 -0.354 -0.354 -0.354 -0.354 -0.354 -0.354
## treatment23:pair11 -0.354 -0.354 -0.354 -0.354 -0.354 -0.354 -0.354 -0.354
## treatment24:pair11 -0.354 -0.354 -0.354 -0.354 -0.354 -0.354 -0.354 -0.354
## treatment25:pair11 -0.354 -0.354 -0.354 -0.354 -0.354 -0.354 -0.354 -0.354
## treatment26:pair11 -0.354 -0.354 -0.354 -0.354 -0.354 -0.354 -0.354 -0.354
## treatment21:pair12 -0.354 -0.354 -0.354 -0.354 -0.354 -0.354 -0.354 -0.354
## treatment22:pair12 -0.354 -0.354 -0.354 -0.354 -0.354 -0.354 -0.354 -0.354
## treatment23:pair12 -0.354 -0.354 -0.354 -0.354 -0.354 -0.354 -0.354 -0.354
## treatment24:pair12 -0.354 -0.354 -0.354 -0.354 -0.354 -0.354 -0.354 -0.354
## treatment25:pair12 -0.354 -0.354 -0.354 -0.354 -0.354 -0.354 -0.354 -0.354
## treatment26:pair12 -0.354 -0.354 -0.354 -0.354 -0.354 -0.354 -0.354 -0.354
## treatment21:pair13 -0.354 -0.354 -0.354 -0.354 -0.354 -0.354 -0.354 -0.354
## treatment22:pair13 -0.354 -0.354 -0.354 -0.354 -0.354 -0.354 -0.354 -0.354
## treatment23:pair13 -0.354 -0.354 -0.354 -0.354 -0.354 -0.354 -0.354 -0.354
## treatment24:pair13 -0.354 -0.354 -0.354 -0.354 -0.354 -0.354 -0.354 -0.354
## treatment25:pair13 -0.354 -0.354 -0.354 -0.354 -0.354 -0.354 -0.354 -0.354
## treatment26:pair13 -0.354 -0.354 -0.354 -0.354 -0.354 -0.354 -0.354 -0.354
## treatment21:pair14 -0.354 -0.354 -0.354 -0.354 -0.354 -0.354 -0.354 -0.354
## treatment22:pair14 -0.354 -0.354 -0.354 -0.354 -0.354 -0.354 -0.354 -0.354
## treatment23:pair14 -0.354 -0.354 -0.354 -0.354 -0.354 -0.354 -0.354 -0.354
## treatment24:pair14 -0.354 -0.354 -0.354 -0.354 -0.354 -0.354 -0.354 -0.354
## treatment25:pair14 -0.354 -0.354 -0.354 -0.354 -0.354 -0.354 -0.354 -0.354
## treatment26:pair14 -0.354 -0.354 -0.354 -0.354 -0.354 -0.354 -0.354 -0.354
## treatment21:pair15 -0.354 -0.354 -0.354 -0.354 -0.354 -0.354 -0.354 -0.354
## treatment22:pair15 -0.354 -0.354 -0.354 -0.354 -0.354 -0.354 -0.354 -0.354
## treatment23:pair15 -0.354 -0.354 -0.354 -0.354 -0.354 -0.354 -0.354 -0.354
## treatment24:pair15 -0.354 -0.354 -0.354 -0.354 -0.354 -0.354 -0.354 -0.354
## treatment25:pair15 -0.354 -0.354 -0.354 -0.354 -0.354 -0.354 -0.354 -0.354
## treatment26:pair15 -0.354 -0.354 -0.354 -0.354 -0.354 -0.354 -0.354 -0.354
##                    pair11 pair12 pair13 pair14 pair15 tr21:2 tr22:2 tr23:2
## treatment21
```

```
## treatment22
## treatment23
## treatment24
## treatment25
## treatment26
## pair2
## pair3
## pair4
## pair5
## pair6
## pair7
## pair8
## pair9
## pair10
## pair11
## pair12              0.500
## pair13              0.500  0.500
## pair14              0.500  0.500  0.500
## pair15              0.500  0.500  0.500  0.500
## treatment21:pair2  -0.354 -0.354 -0.354 -0.354 -0.354
## treatment22:pair2  -0.354 -0.354 -0.354 -0.354 -0.354  0.500
## treatment23:pair2  -0.354 -0.354 -0.354 -0.354 -0.354  0.500  0.500
## treatment24:pair2  -0.354 -0.354 -0.354 -0.354 -0.354  0.500  0.500  0.500
## treatment25:pair2  -0.354 -0.354 -0.354 -0.354 -0.354  0.500  0.500  0.500
## treatment26:pair2  -0.354 -0.354 -0.354 -0.354 -0.354  0.500  0.500  0.500
## treatment21:pair3  -0.354 -0.354 -0.354 -0.354 -0.354  0.500  0.250  0.250
## treatment22:pair3  -0.354 -0.354 -0.354 -0.354 -0.354  0.250  0.500  0.250
## treatment23:pair3  -0.354 -0.354 -0.354 -0.354 -0.354  0.250  0.250  0.500
## treatment24:pair3  -0.354 -0.354 -0.354 -0.354 -0.354  0.250  0.250  0.250
## treatment25:pair3  -0.354 -0.354 -0.354 -0.354 -0.354  0.250  0.250  0.250
## treatment26:pair3  -0.354 -0.354 -0.354 -0.354 -0.354  0.250  0.250  0.250
## treatment21:pair4  -0.354 -0.354 -0.354 -0.354 -0.354  0.500  0.250  0.250
## treatment22:pair4  -0.354 -0.354 -0.354 -0.354 -0.354  0.250  0.500  0.250
## treatment23:pair4  -0.354 -0.354 -0.354 -0.354 -0.354  0.250  0.250  0.500
## treatment24:pair4  -0.354 -0.354 -0.354 -0.354 -0.354  0.250  0.250  0.250
## treatment25:pair4  -0.354 -0.354 -0.354 -0.354 -0.354  0.250  0.250  0.250
## treatment26:pair4  -0.354 -0.354 -0.354 -0.354 -0.354  0.250  0.250  0.250
## treatment21:pair5  -0.354 -0.354 -0.354 -0.354 -0.354  0.500  0.250  0.250
## treatment22:pair5  -0.354 -0.354 -0.354 -0.354 -0.354  0.250  0.500  0.250
## treatment23:pair5  -0.354 -0.354 -0.354 -0.354 -0.354  0.250  0.250  0.500
## treatment24:pair5  -0.354 -0.354 -0.354 -0.354 -0.354  0.250  0.250  0.250
## treatment25:pair5  -0.354 -0.354 -0.354 -0.354 -0.354  0.250  0.250  0.250
## treatment26:pair5  -0.354 -0.354 -0.354 -0.354 -0.354  0.250  0.250  0.250
## treatment21:pair6  -0.354 -0.354 -0.354 -0.354 -0.354  0.500  0.250  0.250
## treatment22:pair6  -0.354 -0.354 -0.354 -0.354 -0.354  0.250  0.500  0.250
## treatment23:pair6  -0.354 -0.354 -0.354 -0.354 -0.354  0.250  0.250  0.500
## treatment24:pair6  -0.354 -0.354 -0.354 -0.354 -0.354  0.250  0.250  0.250
## treatment25:pair6  -0.354 -0.354 -0.354 -0.354 -0.354  0.250  0.250  0.250
## treatment26:pair6  -0.354 -0.354 -0.354 -0.354 -0.354  0.250  0.250  0.250
## treatment21:pair7  -0.354 -0.354 -0.354 -0.354 -0.354  0.500  0.250  0.250
## treatment22:pair7  -0.354 -0.354 -0.354 -0.354 -0.354  0.250  0.500  0.250
## treatment23:pair7  -0.354 -0.354 -0.354 -0.354 -0.354  0.250  0.250  0.500
## treatment24:pair7  -0.354 -0.354 -0.354 -0.354 -0.354  0.250  0.250  0.250
## treatment25:pair7  -0.354 -0.354 -0.354 -0.354 -0.354  0.250  0.250  0.250
```

```
## treatment26:pair7  -0.354 -0.354 -0.354 -0.354 -0.354  0.250  0.250  0.250
## treatment21:pair8  -0.354 -0.354 -0.354 -0.354 -0.354  0.500  0.250  0.250
## treatment22:pair8  -0.354 -0.354 -0.354 -0.354 -0.354  0.250  0.500  0.250
## treatment23:pair8  -0.354 -0.354 -0.354 -0.354 -0.354  0.250  0.250  0.500
## treatment24:pair8  -0.354 -0.354 -0.354 -0.354 -0.354  0.250  0.250  0.250
## treatment25:pair8  -0.354 -0.354 -0.354 -0.354 -0.354  0.250  0.250  0.250
## treatment26:pair8  -0.354 -0.354 -0.354 -0.354 -0.354  0.250  0.250  0.250
## treatment21:pair9  -0.354 -0.354 -0.354 -0.354 -0.354  0.500  0.250  0.250
## treatment22:pair9  -0.354 -0.354 -0.354 -0.354 -0.354  0.250  0.500  0.250
## treatment23:pair9  -0.354 -0.354 -0.354 -0.354 -0.354  0.250  0.250  0.500
## treatment24:pair9  -0.354 -0.354 -0.354 -0.354 -0.354  0.250  0.250  0.250
## treatment25:pair9  -0.354 -0.354 -0.354 -0.354 -0.354  0.250  0.250  0.250
## treatment26:pair9  -0.354 -0.354 -0.354 -0.354 -0.354  0.250  0.250  0.250
## treatment21:pair10 -0.354 -0.354 -0.354 -0.354 -0.354  0.500  0.250  0.250
## treatment22:pair10 -0.354 -0.354 -0.354 -0.354 -0.354  0.250  0.500  0.250
## treatment23:pair10 -0.354 -0.354 -0.354 -0.354 -0.354  0.250  0.250  0.500
## treatment24:pair10 -0.354 -0.354 -0.354 -0.354 -0.354  0.250  0.250  0.250
## treatment25:pair10 -0.354 -0.354 -0.354 -0.354 -0.354  0.250  0.250  0.250
## treatment26:pair10 -0.354 -0.354 -0.354 -0.354 -0.354  0.250  0.250  0.250
## treatment21:pair11 -0.707 -0.354 -0.354 -0.354 -0.354  0.500  0.250  0.250
## treatment22:pair11 -0.707 -0.354 -0.354 -0.354 -0.354  0.250  0.500  0.250
## treatment23:pair11 -0.707 -0.354 -0.354 -0.354 -0.354  0.250  0.250  0.500
## treatment24:pair11 -0.707 -0.354 -0.354 -0.354 -0.354  0.250  0.250  0.250
## treatment25:pair11 -0.707 -0.354 -0.354 -0.354 -0.354  0.250  0.250  0.250
## treatment26:pair11 -0.707 -0.354 -0.354 -0.354 -0.354  0.250  0.250  0.250
## treatment21:pair12 -0.354 -0.707 -0.354 -0.354 -0.354  0.500  0.250  0.250
## treatment22:pair12 -0.354 -0.707 -0.354 -0.354 -0.354  0.250  0.500  0.250
## treatment23:pair12 -0.354 -0.707 -0.354 -0.354 -0.354  0.250  0.250  0.500
## treatment24:pair12 -0.354 -0.707 -0.354 -0.354 -0.354  0.250  0.250  0.250
## treatment25:pair12 -0.354 -0.707 -0.354 -0.354 -0.354  0.250  0.250  0.250
## treatment26:pair12 -0.354 -0.707 -0.354 -0.354 -0.354  0.250  0.250  0.250
## treatment21:pair13 -0.354 -0.354 -0.707 -0.354 -0.354  0.500  0.250  0.250
## treatment22:pair13 -0.354 -0.354 -0.707 -0.354 -0.354  0.250  0.500  0.250
## treatment23:pair13 -0.354 -0.354 -0.707 -0.354 -0.354  0.250  0.250  0.500
## treatment24:pair13 -0.354 -0.354 -0.707 -0.354 -0.354  0.250  0.250  0.250
## treatment25:pair13 -0.354 -0.354 -0.707 -0.354 -0.354  0.250  0.250  0.250
## treatment26:pair13 -0.354 -0.354 -0.707 -0.354 -0.354  0.250  0.250  0.250
## treatment21:pair14 -0.354 -0.354 -0.354 -0.707 -0.354  0.500  0.250  0.250
## treatment22:pair14 -0.354 -0.354 -0.354 -0.707 -0.354  0.250  0.500  0.250
## treatment23:pair14 -0.354 -0.354 -0.354 -0.707 -0.354  0.250  0.250  0.500
## treatment24:pair14 -0.354 -0.354 -0.354 -0.707 -0.354  0.250  0.250  0.250
## treatment25:pair14 -0.354 -0.354 -0.354 -0.707 -0.354  0.250  0.250  0.250
## treatment26:pair14 -0.354 -0.354 -0.354 -0.707 -0.354  0.250  0.250  0.250
## treatment21:pair15 -0.354 -0.354 -0.354 -0.354 -0.707  0.500  0.250  0.250
## treatment22:pair15 -0.354 -0.354 -0.354 -0.354 -0.707  0.250  0.500  0.250
## treatment23:pair15 -0.354 -0.354 -0.354 -0.354 -0.707  0.250  0.250  0.500
## treatment24:pair15 -0.354 -0.354 -0.354 -0.354 -0.707  0.250  0.250  0.250
## treatment25:pair15 -0.354 -0.354 -0.354 -0.354 -0.707  0.250  0.250  0.250
## treatment26:pair15 -0.354 -0.354 -0.354 -0.354 -0.707  0.250  0.250  0.250
##                    tr24:2 tr25:2 tr26:2 tr21:3 tr22:3 tr23:3 tr24:3 tr25:3
## treatment21
## treatment22
## treatment23
## treatment24
```

```
## treatment25
## treatment26
## pair2
## pair3
## pair4
## pair5
## pair6
## pair7
## pair8
## pair9
## pair10
## pair11
## pair12
## pair13
## pair14
## pair15
## treatment21:pair2
## treatment22:pair2
## treatment23:pair2
## treatment24:pair2
## treatment25:pair2   0.500
## treatment26:pair2   0.500  0.500
## treatment21:pair3   0.250  0.250  0.250
## treatment22:pair3   0.250  0.250  0.250  0.500
## treatment23:pair3   0.250  0.250  0.250  0.500  0.500
## treatment24:pair3   0.500  0.250  0.250  0.500  0.500  0.500
## treatment25:pair3   0.250  0.500  0.250  0.500  0.500  0.500  0.500
## treatment26:pair3   0.250  0.250  0.500  0.500  0.500  0.500  0.500  0.500
## treatment21:pair4   0.250  0.250  0.250  0.500  0.250  0.250  0.250  0.250
## treatment22:pair4   0.250  0.250  0.250  0.250  0.500  0.250  0.250  0.250
## treatment23:pair4   0.250  0.250  0.250  0.250  0.250  0.500  0.250  0.250
## treatment24:pair4   0.500  0.250  0.250  0.250  0.250  0.250  0.500  0.250
## treatment25:pair4   0.250  0.500  0.250  0.250  0.250  0.250  0.250  0.500
## treatment26:pair4   0.250  0.250  0.500  0.250  0.250  0.250  0.250  0.250
## treatment21:pair5   0.250  0.250  0.250  0.500  0.250  0.250  0.250  0.250
## treatment22:pair5   0.250  0.250  0.250  0.250  0.500  0.250  0.250  0.250
## treatment23:pair5   0.250  0.250  0.250  0.250  0.250  0.500  0.250  0.250
## treatment24:pair5   0.500  0.250  0.250  0.250  0.250  0.250  0.500  0.250
## treatment25:pair5   0.250  0.500  0.250  0.250  0.250  0.250  0.250  0.500
## treatment26:pair5   0.250  0.250  0.500  0.250  0.250  0.250  0.250  0.250
## treatment21:pair6   0.250  0.250  0.250  0.500  0.250  0.250  0.250  0.250
## treatment22:pair6   0.250  0.250  0.250  0.250  0.500  0.250  0.250  0.250
## treatment23:pair6   0.250  0.250  0.250  0.250  0.250  0.500  0.250  0.250
## treatment24:pair6   0.500  0.250  0.250  0.250  0.250  0.250  0.500  0.250
## treatment25:pair6   0.250  0.500  0.250  0.250  0.250  0.250  0.250  0.500
## treatment26:pair6   0.250  0.250  0.500  0.250  0.250  0.250  0.250  0.250
## treatment21:pair7   0.250  0.250  0.250  0.500  0.250  0.250  0.250  0.250
## treatment22:pair7   0.250  0.250  0.250  0.250  0.500  0.250  0.250  0.250
## treatment23:pair7   0.250  0.250  0.250  0.250  0.250  0.500  0.250  0.250
## treatment24:pair7   0.500  0.250  0.250  0.250  0.250  0.250  0.500  0.250
## treatment25:pair7   0.250  0.500  0.250  0.250  0.250  0.250  0.250  0.500
## treatment26:pair7   0.250  0.250  0.500  0.250  0.250  0.250  0.250  0.250
## treatment21:pair8   0.250  0.250  0.250  0.500  0.250  0.250  0.250  0.250
## treatment22:pair8   0.250  0.250  0.250  0.250  0.500  0.250  0.250  0.250
```

```
## treatment23:pair8   0.250  0.250  0.250  0.250  0.250  0.500  0.250  0.250
## treatment24:pair8   0.500  0.250  0.250  0.250  0.250  0.250  0.500  0.250
## treatment25:pair8   0.250  0.500  0.250  0.250  0.250  0.250  0.250  0.500
## treatment26:pair8   0.250  0.250  0.500  0.250  0.250  0.250  0.250  0.250
## treatment21:pair9   0.250  0.250  0.250  0.500  0.250  0.250  0.250  0.250
## treatment22:pair9   0.250  0.250  0.250  0.250  0.500  0.250  0.250  0.250
## treatment23:pair9   0.250  0.250  0.250  0.250  0.250  0.500  0.250  0.250
## treatment24:pair9   0.500  0.250  0.250  0.250  0.250  0.250  0.500  0.250
## treatment25:pair9   0.250  0.500  0.250  0.250  0.250  0.250  0.250  0.500
## treatment26:pair9   0.250  0.250  0.500  0.250  0.250  0.250  0.250  0.250
## treatment21:pair10  0.250  0.250  0.250  0.500  0.250  0.250  0.250  0.250
## treatment22:pair10  0.250  0.250  0.250  0.250  0.500  0.250  0.250  0.250
## treatment23:pair10  0.250  0.250  0.250  0.250  0.250  0.500  0.250  0.250
## treatment24:pair10  0.500  0.250  0.250  0.250  0.250  0.250  0.500  0.250
## treatment25:pair10  0.250  0.500  0.250  0.250  0.250  0.250  0.250  0.500
## treatment26:pair10  0.250  0.250  0.500  0.250  0.250  0.250  0.250  0.250
## treatment21:pair11  0.250  0.250  0.250  0.500  0.250  0.250  0.250  0.250
## treatment22:pair11  0.250  0.250  0.250  0.250  0.500  0.250  0.250  0.250
## treatment23:pair11  0.250  0.250  0.250  0.250  0.250  0.500  0.250  0.250
## treatment24:pair11  0.500  0.250  0.250  0.250  0.250  0.250  0.500  0.250
## treatment25:pair11  0.250  0.500  0.250  0.250  0.250  0.250  0.250  0.500
## treatment26:pair11  0.250  0.250  0.500  0.250  0.250  0.250  0.250  0.250
## treatment21:pair12  0.250  0.250  0.250  0.500  0.250  0.250  0.250  0.250
## treatment22:pair12  0.250  0.250  0.250  0.250  0.500  0.250  0.250  0.250
## treatment23:pair12  0.250  0.250  0.250  0.250  0.250  0.500  0.250  0.250
## treatment24:pair12  0.500  0.250  0.250  0.250  0.250  0.250  0.500  0.250
## treatment25:pair12  0.250  0.500  0.250  0.250  0.250  0.250  0.250  0.500
## treatment26:pair12  0.250  0.250  0.500  0.250  0.250  0.250  0.250  0.250
## treatment21:pair13  0.250  0.250  0.250  0.500  0.250  0.250  0.250  0.250
## treatment22:pair13  0.250  0.250  0.250  0.250  0.500  0.250  0.250  0.250
## treatment23:pair13  0.250  0.250  0.250  0.250  0.250  0.500  0.250  0.250
## treatment24:pair13  0.500  0.250  0.250  0.250  0.250  0.250  0.500  0.250
## treatment25:pair13  0.250  0.500  0.250  0.250  0.250  0.250  0.250  0.500
## treatment26:pair13  0.250  0.250  0.500  0.250  0.250  0.250  0.250  0.250
## treatment21:pair14  0.250  0.250  0.250  0.500  0.250  0.250  0.250  0.250
## treatment22:pair14  0.250  0.250  0.250  0.250  0.500  0.250  0.250  0.250
## treatment23:pair14  0.250  0.250  0.250  0.250  0.250  0.500  0.250  0.250
## treatment24:pair14  0.500  0.250  0.250  0.250  0.250  0.250  0.500  0.250
## treatment25:pair14  0.250  0.500  0.250  0.250  0.250  0.250  0.250  0.500
## treatment26:pair14  0.250  0.250  0.500  0.250  0.250  0.250  0.250  0.250
## treatment21:pair15  0.250  0.250  0.250  0.500  0.250  0.250  0.250  0.250
## treatment22:pair15  0.250  0.250  0.250  0.250  0.500  0.250  0.250  0.250
## treatment23:pair15  0.250  0.250  0.250  0.250  0.250  0.500  0.250  0.250
## treatment24:pair15  0.500  0.250  0.250  0.250  0.250  0.250  0.500  0.250
## treatment25:pair15  0.250  0.500  0.250  0.250  0.250  0.250  0.250  0.500
## treatment26:pair15  0.250  0.250  0.500  0.250  0.250  0.250  0.250  0.250
##                    tr26:3 tr21:4 tr22:4 tr23:4 tr24:4 tr25:4 tr26:4 tr21:5
## treatment21
## treatment22
## treatment23
## treatment24
## treatment25
## treatment26
## pair2
```

```
## pair3
## pair4
## pair5
## pair6
## pair7
## pair8
## pair9
## pair10
## pair11
## pair12
## pair13
## pair14
## pair15
## treatment21:pair2
## treatment22:pair2
## treatment23:pair2
## treatment24:pair2
## treatment25:pair2
## treatment26:pair2
## treatment21:pair3
## treatment22:pair3
## treatment23:pair3
## treatment24:pair3
## treatment25:pair3
## treatment26:pair3
## treatment21:pair4   0.250
## treatment22:pair4   0.250  0.500
## treatment23:pair4   0.250  0.500  0.500
## treatment24:pair4   0.250  0.500  0.500  0.500
## treatment25:pair4   0.250  0.500  0.500  0.500  0.500
## treatment26:pair4   0.500  0.500  0.500  0.500  0.500  0.500
## treatment21:pair5   0.250  0.500  0.250  0.250  0.250  0.250  0.250
## treatment22:pair5   0.250  0.250  0.500  0.250  0.250  0.250  0.250  0.500
## treatment23:pair5   0.250  0.250  0.250  0.500  0.250  0.250  0.250  0.500
## treatment24:pair5   0.250  0.250  0.250  0.250  0.500  0.250  0.250  0.500
## treatment25:pair5   0.250  0.250  0.250  0.250  0.250  0.500  0.250  0.500
## treatment26:pair5   0.500  0.250  0.250  0.250  0.250  0.250  0.500  0.500
## treatment21:pair6   0.250  0.500  0.250  0.250  0.250  0.250  0.250  0.500
## treatment22:pair6   0.250  0.250  0.500  0.250  0.250  0.250  0.250  0.250
## treatment23:pair6   0.250  0.250  0.250  0.500  0.250  0.250  0.250  0.250
## treatment24:pair6   0.250  0.250  0.250  0.250  0.500  0.250  0.250  0.250
## treatment25:pair6   0.250  0.250  0.250  0.250  0.250  0.500  0.250  0.250
## treatment26:pair6   0.500  0.250  0.250  0.250  0.250  0.250  0.500  0.250
## treatment21:pair7   0.250  0.500  0.250  0.250  0.250  0.250  0.250  0.500
## treatment22:pair7   0.250  0.250  0.500  0.250  0.250  0.250  0.250  0.250
## treatment23:pair7   0.250  0.250  0.250  0.500  0.250  0.250  0.250  0.250
## treatment24:pair7   0.250  0.250  0.250  0.250  0.500  0.250  0.250  0.250
## treatment25:pair7   0.250  0.250  0.250  0.250  0.250  0.500  0.250  0.250
## treatment26:pair7   0.500  0.250  0.250  0.250  0.250  0.250  0.500  0.250
## treatment21:pair8   0.250  0.500  0.250  0.250  0.250  0.250  0.250  0.500
## treatment22:pair8   0.250  0.250  0.500  0.250  0.250  0.250  0.250  0.250
## treatment23:pair8   0.250  0.250  0.250  0.500  0.250  0.250  0.250  0.250
## treatment24:pair8   0.250  0.250  0.250  0.250  0.500  0.250  0.250  0.250
## treatment25:pair8   0.250  0.250  0.250  0.250  0.250  0.500  0.250  0.250
```

```
## treatment26:pair8   0.500  0.250  0.250  0.250  0.250  0.250  0.500  0.250
## treatment21:pair9   0.250  0.500  0.250  0.250  0.250  0.250  0.250  0.500
## treatment22:pair9   0.250  0.250  0.500  0.250  0.250  0.250  0.250  0.250
## treatment23:pair9   0.250  0.250  0.250  0.500  0.250  0.250  0.250  0.250
## treatment24:pair9   0.250  0.250  0.250  0.250  0.500  0.250  0.250  0.250
## treatment25:pair9   0.250  0.250  0.250  0.250  0.250  0.500  0.250  0.250
## treatment26:pair9   0.500  0.250  0.250  0.250  0.250  0.250  0.500  0.250
## treatment21:pair10  0.250  0.500  0.250  0.250  0.250  0.250  0.250  0.500
## treatment22:pair10  0.250  0.250  0.500  0.250  0.250  0.250  0.250  0.250
## treatment23:pair10  0.250  0.250  0.250  0.500  0.250  0.250  0.250  0.250
## treatment24:pair10  0.250  0.250  0.250  0.250  0.500  0.250  0.250  0.250
## treatment25:pair10  0.250  0.250  0.250  0.250  0.250  0.500  0.250  0.250
## treatment26:pair10  0.500  0.250  0.250  0.250  0.250  0.250  0.500  0.250
## treatment21:pair11  0.250  0.500  0.250  0.250  0.250  0.250  0.250  0.500
## treatment22:pair11  0.250  0.250  0.500  0.250  0.250  0.250  0.250  0.250
## treatment23:pair11  0.250  0.250  0.250  0.500  0.250  0.250  0.250  0.250
## treatment24:pair11  0.250  0.250  0.250  0.250  0.500  0.250  0.250  0.250
## treatment25:pair11  0.250  0.250  0.250  0.250  0.250  0.500  0.250  0.250
## treatment26:pair11  0.500  0.250  0.250  0.250  0.250  0.250  0.500  0.250
## treatment21:pair12  0.250  0.500  0.250  0.250  0.250  0.250  0.250  0.500
## treatment22:pair12  0.250  0.250  0.500  0.250  0.250  0.250  0.250  0.250
## treatment23:pair12  0.250  0.250  0.250  0.500  0.250  0.250  0.250  0.250
## treatment24:pair12  0.250  0.250  0.250  0.250  0.500  0.250  0.250  0.250
## treatment25:pair12  0.250  0.250  0.250  0.250  0.250  0.500  0.250  0.250
## treatment26:pair12  0.500  0.250  0.250  0.250  0.250  0.250  0.500  0.250
## treatment21:pair13  0.250  0.500  0.250  0.250  0.250  0.250  0.250  0.500
## treatment22:pair13  0.250  0.250  0.500  0.250  0.250  0.250  0.250  0.250
## treatment23:pair13  0.250  0.250  0.250  0.500  0.250  0.250  0.250  0.250
## treatment24:pair13  0.250  0.250  0.250  0.250  0.500  0.250  0.250  0.250
## treatment25:pair13  0.250  0.250  0.250  0.250  0.250  0.500  0.250  0.250
## treatment26:pair13  0.500  0.250  0.250  0.250  0.250  0.250  0.500  0.250
## treatment21:pair14  0.250  0.500  0.250  0.250  0.250  0.250  0.250  0.500
## treatment22:pair14  0.250  0.250  0.500  0.250  0.250  0.250  0.250  0.250
## treatment23:pair14  0.250  0.250  0.250  0.500  0.250  0.250  0.250  0.250
## treatment24:pair14  0.250  0.250  0.250  0.250  0.500  0.250  0.250  0.250
## treatment25:pair14  0.250  0.250  0.250  0.250  0.250  0.500  0.250  0.250
## treatment26:pair14  0.500  0.250  0.250  0.250  0.250  0.250  0.500  0.250
## treatment21:pair15  0.250  0.500  0.250  0.250  0.250  0.250  0.250  0.500
## treatment22:pair15  0.250  0.250  0.500  0.250  0.250  0.250  0.250  0.250
## treatment23:pair15  0.250  0.250  0.250  0.500  0.250  0.250  0.250  0.250
## treatment24:pair15  0.250  0.250  0.250  0.250  0.500  0.250  0.250  0.250
## treatment25:pair15  0.250  0.250  0.250  0.250  0.250  0.500  0.250  0.250
## treatment26:pair15  0.500  0.250  0.250  0.250  0.250  0.250  0.500  0.250
##                    tr22:5 tr23:5 tr24:5 tr25:5 tr26:5 tr21:6 tr22:6 tr23:6
## treatment21
## treatment22
## treatment23
## treatment24
## treatment25
## treatment26
## pair2
## pair3
## pair4
## pair5
```

```
## pair6
## pair7
## pair8
## pair9
## pair10
## pair11
## pair12
## pair13
## pair14
## pair15
## treatment21:pair2
## treatment22:pair2
## treatment23:pair2
## treatment24:pair2
## treatment25:pair2
## treatment26:pair2
## treatment21:pair3
## treatment22:pair3
## treatment23:pair3
## treatment24:pair3
## treatment25:pair3
## treatment26:pair3
## treatment21:pair4
## treatment22:pair4
## treatment23:pair4
## treatment24:pair4
## treatment25:pair4
## treatment26:pair4
## treatment21:pair5
## treatment22:pair5
## treatment23:pair5   0.500
## treatment24:pair5   0.500  0.500
## treatment25:pair5   0.500  0.500  0.500
## treatment26:pair5   0.500  0.500  0.500  0.500
## treatment21:pair6   0.250  0.250  0.250  0.250  0.250
## treatment22:pair6   0.500  0.250  0.250  0.250  0.250  0.500
## treatment23:pair6   0.250  0.500  0.250  0.250  0.250  0.500  0.500
## treatment24:pair6   0.250  0.250  0.500  0.250  0.250  0.500  0.500  0.500
## treatment25:pair6   0.250  0.250  0.250  0.500  0.250  0.500  0.500  0.500
## treatment26:pair6   0.250  0.250  0.250  0.250  0.500  0.500  0.500  0.500
## treatment21:pair7   0.250  0.250  0.250  0.250  0.250  0.500  0.250  0.250
## treatment22:pair7   0.500  0.250  0.250  0.250  0.250  0.250  0.500  0.250
## treatment23:pair7   0.250  0.500  0.250  0.250  0.250  0.250  0.250  0.500
## treatment24:pair7   0.250  0.250  0.500  0.250  0.250  0.250  0.250  0.250
## treatment25:pair7   0.250  0.250  0.250  0.500  0.250  0.250  0.250  0.250
## treatment26:pair7   0.250  0.250  0.250  0.250  0.500  0.250  0.250  0.250
## treatment21:pair8   0.250  0.250  0.250  0.250  0.250  0.500  0.250  0.250
## treatment22:pair8   0.500  0.250  0.250  0.250  0.250  0.250  0.500  0.250
## treatment23:pair8   0.250  0.500  0.250  0.250  0.250  0.250  0.250  0.500
## treatment24:pair8   0.250  0.250  0.500  0.250  0.250  0.250  0.250  0.250
## treatment25:pair8   0.250  0.250  0.250  0.500  0.250  0.250  0.250  0.250
## treatment26:pair8   0.250  0.250  0.250  0.250  0.500  0.250  0.250  0.250
## treatment21:pair9   0.250  0.250  0.250  0.250  0.250  0.500  0.250  0.250
## treatment22:pair9   0.500  0.250  0.250  0.250  0.250  0.250  0.500  0.250
```

```
## treatment23:pair9   0.250  0.500  0.250  0.250  0.250  0.250  0.250  0.500
## treatment24:pair9   0.250  0.250  0.500  0.250  0.250  0.250  0.250  0.250
## treatment25:pair9   0.250  0.250  0.250  0.500  0.250  0.250  0.250  0.250
## treatment26:pair9   0.250  0.250  0.250  0.250  0.500  0.250  0.250  0.250
## treatment21:pair10  0.250  0.250  0.250  0.250  0.250  0.500  0.250  0.250
## treatment22:pair10  0.500  0.250  0.250  0.250  0.250  0.250  0.500  0.250
## treatment23:pair10  0.250  0.500  0.250  0.250  0.250  0.250  0.250  0.500
## treatment24:pair10  0.250  0.250  0.500  0.250  0.250  0.250  0.250  0.250
## treatment25:pair10  0.250  0.250  0.250  0.500  0.250  0.250  0.250  0.250
## treatment26:pair10  0.250  0.250  0.250  0.250  0.500  0.250  0.250  0.250
## treatment21:pair11  0.250  0.250  0.250  0.250  0.250  0.500  0.250  0.250
## treatment22:pair11  0.500  0.250  0.250  0.250  0.250  0.250  0.500  0.250
## treatment23:pair11  0.250  0.500  0.250  0.250  0.250  0.250  0.250  0.500
## treatment24:pair11  0.250  0.250  0.500  0.250  0.250  0.250  0.250  0.250
## treatment25:pair11  0.250  0.250  0.250  0.500  0.250  0.250  0.250  0.250
## treatment26:pair11  0.250  0.250  0.250  0.250  0.500  0.250  0.250  0.250
## treatment21:pair12  0.250  0.250  0.250  0.250  0.250  0.500  0.250  0.250
## treatment22:pair12  0.500  0.250  0.250  0.250  0.250  0.250  0.500  0.250
## treatment23:pair12  0.250  0.500  0.250  0.250  0.250  0.250  0.250  0.500
## treatment24:pair12  0.250  0.250  0.500  0.250  0.250  0.250  0.250  0.250
## treatment25:pair12  0.250  0.250  0.250  0.500  0.250  0.250  0.250  0.250
## treatment26:pair12  0.250  0.250  0.250  0.250  0.500  0.250  0.250  0.250
## treatment21:pair13  0.250  0.250  0.250  0.250  0.250  0.500  0.250  0.250
## treatment22:pair13  0.500  0.250  0.250  0.250  0.250  0.250  0.500  0.250
## treatment23:pair13  0.250  0.500  0.250  0.250  0.250  0.250  0.250  0.500
## treatment24:pair13  0.250  0.250  0.500  0.250  0.250  0.250  0.250  0.250
## treatment25:pair13  0.250  0.250  0.250  0.500  0.250  0.250  0.250  0.250
## treatment26:pair13  0.250  0.250  0.250  0.250  0.500  0.250  0.250  0.250
## treatment21:pair14  0.250  0.250  0.250  0.250  0.250  0.500  0.250  0.250
## treatment22:pair14  0.500  0.250  0.250  0.250  0.250  0.250  0.500  0.250
## treatment23:pair14  0.250  0.500  0.250  0.250  0.250  0.250  0.250  0.500
## treatment24:pair14  0.250  0.250  0.500  0.250  0.250  0.250  0.250  0.250
## treatment25:pair14  0.250  0.250  0.250  0.500  0.250  0.250  0.250  0.250
## treatment26:pair14  0.250  0.250  0.250  0.250  0.500  0.250  0.250  0.250
## treatment21:pair15  0.250  0.250  0.250  0.250  0.250  0.500  0.250  0.250
## treatment22:pair15  0.500  0.250  0.250  0.250  0.250  0.250  0.500  0.250
## treatment23:pair15  0.250  0.500  0.250  0.250  0.250  0.250  0.250  0.500
## treatment24:pair15  0.250  0.250  0.500  0.250  0.250  0.250  0.250  0.250
## treatment25:pair15  0.250  0.250  0.250  0.500  0.250  0.250  0.250  0.250
## treatment26:pair15  0.250  0.250  0.250  0.250  0.500  0.250  0.250  0.250
##                    tr24:6 tr25:6 tr26:6 tr21:7 tr22:7 tr23:7 tr24:7 tr25:7
## treatment21
## treatment22
## treatment23
## treatment24
## treatment25
## treatment26
## pair2
## pair3
## pair4
## pair5
## pair6
## pair7
## pair8
```

```
## pair9
## pair10
## pair11
## pair12
## pair13
## pair14
## pair15
## treatment21:pair2
## treatment22:pair2
## treatment23:pair2
## treatment24:pair2
## treatment25:pair2
## treatment26:pair2
## treatment21:pair3
## treatment22:pair3
## treatment23:pair3
## treatment24:pair3
## treatment25:pair3
## treatment26:pair3
## treatment21:pair4
## treatment22:pair4
## treatment23:pair4
## treatment24:pair4
## treatment25:pair4
## treatment26:pair4
## treatment21:pair5
## treatment22:pair5
## treatment23:pair5
## treatment24:pair5
## treatment25:pair5
## treatment26:pair5
## treatment21:pair6
## treatment22:pair6
## treatment23:pair6
## treatment24:pair6
## treatment25:pair6   0.500
## treatment26:pair6   0.500  0.500
## treatment21:pair7   0.250  0.250  0.250
## treatment22:pair7   0.250  0.250  0.250  0.500
## treatment23:pair7   0.250  0.250  0.250  0.500  0.500
## treatment24:pair7   0.500  0.250  0.250  0.500  0.500  0.500
## treatment25:pair7   0.250  0.500  0.250  0.500  0.500  0.500  0.500
## treatment26:pair7   0.250  0.250  0.500  0.500  0.500  0.500  0.500  0.500
## treatment21:pair8   0.250  0.250  0.250  0.500  0.250  0.250  0.250  0.250
## treatment22:pair8   0.250  0.250  0.250  0.250  0.500  0.250  0.250  0.250
## treatment23:pair8   0.250  0.250  0.250  0.250  0.250  0.500  0.250  0.250
## treatment24:pair8   0.500  0.250  0.250  0.250  0.250  0.250  0.500  0.250
## treatment25:pair8   0.250  0.500  0.250  0.250  0.250  0.250  0.250  0.500
## treatment26:pair8   0.250  0.250  0.500  0.250  0.250  0.250  0.250  0.250
## treatment21:pair9   0.250  0.250  0.250  0.500  0.250  0.250  0.250  0.250
## treatment22:pair9   0.250  0.250  0.250  0.250  0.500  0.250  0.250  0.250
## treatment23:pair9   0.250  0.250  0.250  0.250  0.250  0.500  0.250  0.250
## treatment24:pair9   0.500  0.250  0.250  0.250  0.250  0.250  0.500  0.250
## treatment25:pair9   0.250  0.500  0.250  0.250  0.250  0.250  0.250  0.500
```

```
## treatment26:pair9   0.250  0.250  0.500  0.250  0.250  0.250  0.250  0.250
## treatment21:pair10  0.250  0.250  0.250  0.500  0.250  0.250  0.250  0.250
## treatment22:pair10  0.250  0.250  0.250  0.250  0.500  0.250  0.250  0.250
## treatment23:pair10  0.250  0.250  0.250  0.250  0.250  0.500  0.250  0.250
## treatment24:pair10  0.500  0.250  0.250  0.250  0.250  0.250  0.500  0.250
## treatment25:pair10  0.250  0.500  0.250  0.250  0.250  0.250  0.250  0.500
## treatment26:pair10  0.250  0.250  0.500  0.250  0.250  0.250  0.250  0.250
## treatment21:pair11  0.250  0.250  0.250  0.500  0.250  0.250  0.250  0.250
## treatment22:pair11  0.250  0.250  0.250  0.250  0.500  0.250  0.250  0.250
## treatment23:pair11  0.250  0.250  0.250  0.250  0.250  0.500  0.250  0.250
## treatment24:pair11  0.500  0.250  0.250  0.250  0.250  0.250  0.500  0.250
## treatment25:pair11  0.250  0.500  0.250  0.250  0.250  0.250  0.250  0.500
## treatment26:pair11  0.250  0.250  0.500  0.250  0.250  0.250  0.250  0.250
## treatment21:pair12  0.250  0.250  0.250  0.500  0.250  0.250  0.250  0.250
## treatment22:pair12  0.250  0.250  0.250  0.250  0.500  0.250  0.250  0.250
## treatment23:pair12  0.250  0.250  0.250  0.250  0.250  0.500  0.250  0.250
## treatment24:pair12  0.500  0.250  0.250  0.250  0.250  0.250  0.500  0.250
## treatment25:pair12  0.250  0.500  0.250  0.250  0.250  0.250  0.250  0.500
## treatment26:pair12  0.250  0.250  0.500  0.250  0.250  0.250  0.250  0.250
## treatment21:pair13  0.250  0.250  0.250  0.500  0.250  0.250  0.250  0.250
## treatment22:pair13  0.250  0.250  0.250  0.250  0.500  0.250  0.250  0.250
## treatment23:pair13  0.250  0.250  0.250  0.250  0.250  0.500  0.250  0.250
## treatment24:pair13  0.500  0.250  0.250  0.250  0.250  0.250  0.500  0.250
## treatment25:pair13  0.250  0.500  0.250  0.250  0.250  0.250  0.250  0.500
## treatment26:pair13  0.250  0.250  0.500  0.250  0.250  0.250  0.250  0.250
## treatment21:pair14  0.250  0.250  0.250  0.500  0.250  0.250  0.250  0.250
## treatment22:pair14  0.250  0.250  0.250  0.250  0.500  0.250  0.250  0.250
## treatment23:pair14  0.250  0.250  0.250  0.250  0.250  0.500  0.250  0.250
## treatment24:pair14  0.500  0.250  0.250  0.250  0.250  0.250  0.500  0.250
## treatment25:pair14  0.250  0.500  0.250  0.250  0.250  0.250  0.250  0.500
## treatment26:pair14  0.250  0.250  0.500  0.250  0.250  0.250  0.250  0.250
## treatment21:pair15  0.250  0.250  0.250  0.500  0.250  0.250  0.250  0.250
## treatment22:pair15  0.250  0.250  0.250  0.250  0.500  0.250  0.250  0.250
## treatment23:pair15  0.250  0.250  0.250  0.250  0.250  0.500  0.250  0.250
## treatment24:pair15  0.500  0.250  0.250  0.250  0.250  0.250  0.500  0.250
## treatment25:pair15  0.250  0.500  0.250  0.250  0.250  0.250  0.250  0.500
## treatment26:pair15  0.250  0.250  0.500  0.250  0.250  0.250  0.250  0.250
##                    tr26:7 tr21:8 tr22:8 tr23:8 tr24:8 tr25:8 tr26:8 tr21:9
## treatment21
## treatment22
## treatment23
## treatment24
## treatment25
## treatment26
## pair2
## pair3
## pair4
## pair5
## pair6
## pair7
## pair8
## pair9
## pair10
## pair11
```

```
## pair12
## pair13
## pair14
## pair15
## treatment21:pair2
## treatment22:pair2
## treatment23:pair2
## treatment24:pair2
## treatment25:pair2
## treatment26:pair2
## treatment21:pair3
## treatment22:pair3
## treatment23:pair3
## treatment24:pair3
## treatment25:pair3
## treatment26:pair3
## treatment21:pair4
## treatment22:pair4
## treatment23:pair4
## treatment24:pair4
## treatment25:pair4
## treatment26:pair4
## treatment21:pair5
## treatment22:pair5
## treatment23:pair5
## treatment24:pair5
## treatment25:pair5
## treatment26:pair5
## treatment21:pair6
## treatment22:pair6
## treatment23:pair6
## treatment24:pair6
## treatment25:pair6
## treatment26:pair6
## treatment21:pair7
## treatment22:pair7
## treatment23:pair7
## treatment24:pair7
## treatment25:pair7
## treatment26:pair7
## treatment21:pair8   0.250
## treatment22:pair8   0.250  0.500
## treatment23:pair8   0.250  0.500  0.500
## treatment24:pair8   0.250  0.500  0.500  0.500
## treatment25:pair8   0.250  0.500  0.500  0.500  0.500
## treatment26:pair8   0.500  0.500  0.500  0.500  0.500  0.500
## treatment21:pair9   0.250  0.500  0.250  0.250  0.250  0.250  0.250
## treatment22:pair9   0.250  0.250  0.500  0.250  0.250  0.250  0.250  0.500
## treatment23:pair9   0.250  0.250  0.250  0.500  0.250  0.250  0.250  0.500
## treatment24:pair9   0.250  0.250  0.250  0.250  0.500  0.250  0.250  0.500
## treatment25:pair9   0.250  0.250  0.250  0.250  0.250  0.500  0.250  0.500
## treatment26:pair9   0.500  0.250  0.250  0.250  0.250  0.250  0.500  0.500
## treatment21:pair10  0.250  0.500  0.250  0.250  0.250  0.250  0.250  0.500
## treatment22:pair10  0.250  0.250  0.500  0.250  0.250  0.250  0.250  0.250
```

```
## treatment23:pair10  0.250  0.250  0.250  0.500  0.250  0.250  0.250  0.250
## treatment24:pair10  0.250  0.250  0.250  0.250  0.500  0.250  0.250  0.250
## treatment25:pair10  0.250  0.250  0.250  0.250  0.250  0.500  0.250  0.250
## treatment26:pair10  0.500  0.250  0.250  0.250  0.250  0.250  0.500  0.250
## treatment21:pair11  0.250  0.500  0.250  0.250  0.250  0.250  0.250  0.500
## treatment22:pair11  0.250  0.250  0.500  0.250  0.250  0.250  0.250  0.250
## treatment23:pair11  0.250  0.250  0.250  0.500  0.250  0.250  0.250  0.250
## treatment24:pair11  0.250  0.250  0.250  0.250  0.500  0.250  0.250  0.250
## treatment25:pair11  0.250  0.250  0.250  0.250  0.250  0.500  0.250  0.250
## treatment26:pair11  0.500  0.250  0.250  0.250  0.250  0.250  0.500  0.250
## treatment21:pair12  0.250  0.500  0.250  0.250  0.250  0.250  0.250  0.500
## treatment22:pair12  0.250  0.250  0.500  0.250  0.250  0.250  0.250  0.250
## treatment23:pair12  0.250  0.250  0.250  0.500  0.250  0.250  0.250  0.250
## treatment24:pair12  0.250  0.250  0.250  0.250  0.500  0.250  0.250  0.250
## treatment25:pair12  0.250  0.250  0.250  0.250  0.250  0.500  0.250  0.250
## treatment26:pair12  0.500  0.250  0.250  0.250  0.250  0.250  0.500  0.250
## treatment21:pair13  0.250  0.500  0.250  0.250  0.250  0.250  0.250  0.500
## treatment22:pair13  0.250  0.250  0.500  0.250  0.250  0.250  0.250  0.250
## treatment23:pair13  0.250  0.250  0.250  0.500  0.250  0.250  0.250  0.250
## treatment24:pair13  0.250  0.250  0.250  0.250  0.500  0.250  0.250  0.250
## treatment25:pair13  0.250  0.250  0.250  0.250  0.250  0.500  0.250  0.250
## treatment26:pair13  0.500  0.250  0.250  0.250  0.250  0.250  0.500  0.250
## treatment21:pair14  0.250  0.500  0.250  0.250  0.250  0.250  0.250  0.500
## treatment22:pair14  0.250  0.250  0.500  0.250  0.250  0.250  0.250  0.250
## treatment23:pair14  0.250  0.250  0.250  0.500  0.250  0.250  0.250  0.250
## treatment24:pair14  0.250  0.250  0.250  0.250  0.500  0.250  0.250  0.250
## treatment25:pair14  0.250  0.250  0.250  0.250  0.250  0.500  0.250  0.250
## treatment26:pair14  0.500  0.250  0.250  0.250  0.250  0.250  0.500  0.250
## treatment21:pair15  0.250  0.500  0.250  0.250  0.250  0.250  0.250  0.500
## treatment22:pair15  0.250  0.250  0.500  0.250  0.250  0.250  0.250  0.250
## treatment23:pair15  0.250  0.250  0.250  0.500  0.250  0.250  0.250  0.250
## treatment24:pair15  0.250  0.250  0.250  0.250  0.500  0.250  0.250  0.250
## treatment25:pair15  0.250  0.250  0.250  0.250  0.250  0.500  0.250  0.250
## treatment26:pair15  0.500  0.250  0.250  0.250  0.250  0.250  0.500  0.250
##                    tr22:9 tr23:9 tr24:9 tr25:9 tr26:9 t21:10 t22:10 t23:10
## treatment21
## treatment22
## treatment23
## treatment24
## treatment25
## treatment26
## pair2
## pair3
## pair4
## pair5
## pair6
## pair7
## pair8
## pair9
## pair10
## pair11
## pair12
## pair13
## pair14
```

```
## pair15
## treatment21:pair2
## treatment22:pair2
## treatment23:pair2
## treatment24:pair2
## treatment25:pair2
## treatment26:pair2
## treatment21:pair3
## treatment22:pair3
## treatment23:pair3
## treatment24:pair3
## treatment25:pair3
## treatment26:pair3
## treatment21:pair4
## treatment22:pair4
## treatment23:pair4
## treatment24:pair4
## treatment25:pair4
## treatment26:pair4
## treatment21:pair5
## treatment22:pair5
## treatment23:pair5
## treatment24:pair5
## treatment25:pair5
## treatment26:pair5
## treatment21:pair6
## treatment22:pair6
## treatment23:pair6
## treatment24:pair6
## treatment25:pair6
## treatment26:pair6
## treatment21:pair7
## treatment22:pair7
## treatment23:pair7
## treatment24:pair7
## treatment25:pair7
## treatment26:pair7
## treatment21:pair8
## treatment22:pair8
## treatment23:pair8
## treatment24:pair8
## treatment25:pair8
## treatment26:pair8
## treatment21:pair9
## treatment22:pair9
## treatment23:pair9   0.500
## treatment24:pair9   0.500  0.500
## treatment25:pair9   0.500  0.500  0.500
## treatment26:pair9   0.500  0.500  0.500  0.500
## treatment21:pair10  0.250  0.250  0.250  0.250  0.250
## treatment22:pair10  0.500  0.250  0.250  0.250  0.250  0.500
## treatment23:pair10  0.250  0.500  0.250  0.250  0.250  0.500  0.500
## treatment24:pair10  0.250  0.250  0.500  0.250  0.250  0.500  0.500  0.500
## treatment25:pair10  0.250  0.250  0.250  0.500  0.250  0.500  0.500  0.500
```

```
## treatment26:pair10  0.250  0.250  0.250  0.250  0.500  0.500  0.500  0.500
## treatment21:pair11  0.250  0.250  0.250  0.250  0.250  0.500  0.250  0.250
## treatment22:pair11  0.500  0.250  0.250  0.250  0.250  0.250  0.500  0.250
## treatment23:pair11  0.250  0.500  0.250  0.250  0.250  0.250  0.250  0.500
## treatment24:pair11  0.250  0.250  0.500  0.250  0.250  0.250  0.250  0.250
## treatment25:pair11  0.250  0.250  0.250  0.500  0.250  0.250  0.250  0.250
## treatment26:pair11  0.250  0.250  0.250  0.250  0.500  0.250  0.250  0.250
## treatment21:pair12  0.250  0.250  0.250  0.250  0.250  0.500  0.250  0.250
## treatment22:pair12  0.500  0.250  0.250  0.250  0.250  0.250  0.500  0.250
## treatment23:pair12  0.250  0.500  0.250  0.250  0.250  0.250  0.250  0.500
## treatment24:pair12  0.250  0.250  0.500  0.250  0.250  0.250  0.250  0.250
## treatment25:pair12  0.250  0.250  0.250  0.500  0.250  0.250  0.250  0.250
## treatment26:pair12  0.250  0.250  0.250  0.250  0.500  0.250  0.250  0.250
## treatment21:pair13  0.250  0.250  0.250  0.250  0.250  0.500  0.250  0.250
## treatment22:pair13  0.500  0.250  0.250  0.250  0.250  0.250  0.500  0.250
## treatment23:pair13  0.250  0.500  0.250  0.250  0.250  0.250  0.250  0.500
## treatment24:pair13  0.250  0.250  0.500  0.250  0.250  0.250  0.250  0.250
## treatment25:pair13  0.250  0.250  0.250  0.500  0.250  0.250  0.250  0.250
## treatment26:pair13  0.250  0.250  0.250  0.250  0.500  0.250  0.250  0.250
## treatment21:pair14  0.250  0.250  0.250  0.250  0.250  0.500  0.250  0.250
## treatment22:pair14  0.500  0.250  0.250  0.250  0.250  0.250  0.500  0.250
## treatment23:pair14  0.250  0.500  0.250  0.250  0.250  0.250  0.250  0.500
## treatment24:pair14  0.250  0.250  0.500  0.250  0.250  0.250  0.250  0.250
## treatment25:pair14  0.250  0.250  0.250  0.500  0.250  0.250  0.250  0.250
## treatment26:pair14  0.250  0.250  0.250  0.250  0.500  0.250  0.250  0.250
## treatment21:pair15  0.250  0.250  0.250  0.250  0.250  0.500  0.250  0.250
## treatment22:pair15  0.500  0.250  0.250  0.250  0.250  0.250  0.500  0.250
## treatment23:pair15  0.250  0.500  0.250  0.250  0.250  0.250  0.250  0.500
## treatment24:pair15  0.250  0.250  0.500  0.250  0.250  0.250  0.250  0.250
## treatment25:pair15  0.250  0.250  0.250  0.500  0.250  0.250  0.250  0.250
## treatment26:pair15  0.250  0.250  0.250  0.250  0.500  0.250  0.250  0.250
##                    t24:10 t25:10 t26:10 t21:11 t22:11 t23:11 t24:11 t25:11
## treatment21
## treatment22
## treatment23
## treatment24
## treatment25
## treatment26
## pair2
## pair3
## pair4
## pair5
## pair6
## pair7
## pair8
## pair9
## pair10
## pair11
## pair12
## pair13
## pair14
## pair15
## treatment21:pair2
## treatment22:pair2
```

```
## treatment23:pair2
## treatment24:pair2
## treatment25:pair2
## treatment26:pair2
## treatment21:pair3
## treatment22:pair3
## treatment23:pair3
## treatment24:pair3
## treatment25:pair3
## treatment26:pair3
## treatment21:pair4
## treatment22:pair4
## treatment23:pair4
## treatment24:pair4
## treatment25:pair4
## treatment26:pair4
## treatment21:pair5
## treatment22:pair5
## treatment23:pair5
## treatment24:pair5
## treatment25:pair5
## treatment26:pair5
## treatment21:pair6
## treatment22:pair6
## treatment23:pair6
## treatment24:pair6
## treatment25:pair6
## treatment26:pair6
## treatment21:pair7
## treatment22:pair7
## treatment23:pair7
## treatment24:pair7
## treatment25:pair7
## treatment26:pair7
## treatment21:pair8
## treatment22:pair8
## treatment23:pair8
## treatment24:pair8
## treatment25:pair8
## treatment26:pair8
## treatment21:pair9
## treatment22:pair9
## treatment23:pair9
## treatment24:pair9
## treatment25:pair9
## treatment26:pair9
## treatment21:pair10
## treatment22:pair10
## treatment23:pair10
## treatment24:pair10
## treatment25:pair10  0.500
## treatment26:pair10  0.500  0.500
## treatment21:pair11  0.250  0.250  0.250
## treatment22:pair11  0.250  0.250  0.250  0.500
```

```
## treatment23:pair11  0.250  0.250  0.250  0.500  0.500
## treatment24:pair11  0.500  0.250  0.250  0.500  0.500  0.500
## treatment25:pair11  0.250  0.500  0.250  0.500  0.500  0.500  0.500
## treatment26:pair11  0.250  0.250  0.500  0.500  0.500  0.500  0.500  0.500
## treatment21:pair12  0.250  0.250  0.250  0.500  0.250  0.250  0.250  0.250
## treatment22:pair12  0.250  0.250  0.250  0.250  0.500  0.250  0.250  0.250
## treatment23:pair12  0.250  0.250  0.250  0.250  0.250  0.500  0.250  0.250
## treatment24:pair12  0.500  0.250  0.250  0.250  0.250  0.250  0.500  0.250
## treatment25:pair12  0.250  0.500  0.250  0.250  0.250  0.250  0.250  0.500
## treatment26:pair12  0.250  0.250  0.500  0.250  0.250  0.250  0.250  0.250
## treatment21:pair13  0.250  0.250  0.250  0.500  0.250  0.250  0.250  0.250
## treatment22:pair13  0.250  0.250  0.250  0.250  0.500  0.250  0.250  0.250
## treatment23:pair13  0.250  0.250  0.250  0.250  0.250  0.500  0.250  0.250
## treatment24:pair13  0.500  0.250  0.250  0.250  0.250  0.250  0.500  0.250
## treatment25:pair13  0.250  0.500  0.250  0.250  0.250  0.250  0.250  0.500
## treatment26:pair13  0.250  0.250  0.500  0.250  0.250  0.250  0.250  0.250
## treatment21:pair14  0.250  0.250  0.250  0.500  0.250  0.250  0.250  0.250
## treatment22:pair14  0.250  0.250  0.250  0.250  0.500  0.250  0.250  0.250
## treatment23:pair14  0.250  0.250  0.250  0.250  0.250  0.500  0.250  0.250
## treatment24:pair14  0.500  0.250  0.250  0.250  0.250  0.250  0.500  0.250
## treatment25:pair14  0.250  0.500  0.250  0.250  0.250  0.250  0.250  0.500
## treatment26:pair14  0.250  0.250  0.500  0.250  0.250  0.250  0.250  0.250
## treatment21:pair15  0.250  0.250  0.250  0.500  0.250  0.250  0.250  0.250
## treatment22:pair15  0.250  0.250  0.250  0.250  0.500  0.250  0.250  0.250
## treatment23:pair15  0.250  0.250  0.250  0.250  0.250  0.500  0.250  0.250
## treatment24:pair15  0.500  0.250  0.250  0.250  0.250  0.250  0.500  0.250
## treatment25:pair15  0.250  0.500  0.250  0.250  0.250  0.250  0.250  0.500
## treatment26:pair15  0.250  0.250  0.500  0.250  0.250  0.250  0.250  0.250
##                    t26:11 t21:12 t22:12 t23:12 t24:12 t25:12 t26:12 t21:13
## treatment21
## treatment22
## treatment23
## treatment24
## treatment25
## treatment26
## pair2
## pair3
## pair4
## pair5
## pair6
## pair7
## pair8
## pair9
## pair10
## pair11
## pair12
## pair13
## pair14
## pair15
## treatment21:pair2
## treatment22:pair2
## treatment23:pair2
## treatment24:pair2
## treatment25:pair2
```

```
## treatment26:pair2
## treatment21:pair3
## treatment22:pair3
## treatment23:pair3
## treatment24:pair3
## treatment25:pair3
## treatment26:pair3
## treatment21:pair4
## treatment22:pair4
## treatment23:pair4
## treatment24:pair4
## treatment25:pair4
## treatment26:pair4
## treatment21:pair5
## treatment22:pair5
## treatment23:pair5
## treatment24:pair5
## treatment25:pair5
## treatment26:pair5
## treatment21:pair6
## treatment22:pair6
## treatment23:pair6
## treatment24:pair6
## treatment25:pair6
## treatment26:pair6
## treatment21:pair7
## treatment22:pair7
## treatment23:pair7
## treatment24:pair7
## treatment25:pair7
## treatment26:pair7
## treatment21:pair8
## treatment22:pair8
## treatment23:pair8
## treatment24:pair8
## treatment25:pair8
## treatment26:pair8
## treatment21:pair9
## treatment22:pair9
## treatment23:pair9
## treatment24:pair9
## treatment25:pair9
## treatment26:pair9
## treatment21:pair10
## treatment22:pair10
## treatment23:pair10
## treatment24:pair10
## treatment25:pair10
## treatment26:pair10
## treatment21:pair11
## treatment22:pair11
## treatment23:pair11
## treatment24:pair11
## treatment25:pair11
```

```
## treatment26:pair11
## treatment21:pair12  0.250
## treatment22:pair12  0.250  0.500
## treatment23:pair12  0.250  0.500  0.500
## treatment24:pair12  0.250  0.500  0.500  0.500
## treatment25:pair12  0.250  0.500  0.500  0.500  0.500
## treatment26:pair12  0.500  0.500  0.500  0.500  0.500  0.500
## treatment21:pair13  0.250  0.500  0.250  0.250  0.250  0.250  0.250
## treatment22:pair13  0.250  0.250  0.500  0.250  0.250  0.250  0.250  0.500
## treatment23:pair13  0.250  0.250  0.250  0.500  0.250  0.250  0.250  0.500
## treatment24:pair13  0.250  0.250  0.250  0.250  0.500  0.250  0.250  0.500
## treatment25:pair13  0.250  0.250  0.250  0.250  0.250  0.500  0.250  0.500
## treatment26:pair13  0.500  0.250  0.250  0.250  0.250  0.250  0.500  0.500
## treatment21:pair14  0.250  0.500  0.250  0.250  0.250  0.250  0.250  0.500
## treatment22:pair14  0.250  0.250  0.500  0.250  0.250  0.250  0.250  0.250
## treatment23:pair14  0.250  0.250  0.250  0.500  0.250  0.250  0.250  0.250
## treatment24:pair14  0.250  0.250  0.250  0.250  0.500  0.250  0.250  0.250
## treatment25:pair14  0.250  0.250  0.250  0.250  0.250  0.500  0.250  0.250
## treatment26:pair14  0.500  0.250  0.250  0.250  0.250  0.250  0.500  0.250
## treatment21:pair15  0.250  0.500  0.250  0.250  0.250  0.250  0.250  0.500
## treatment22:pair15  0.250  0.250  0.500  0.250  0.250  0.250  0.250  0.250
## treatment23:pair15  0.250  0.250  0.250  0.500  0.250  0.250  0.250  0.250
## treatment24:pair15  0.250  0.250  0.250  0.250  0.500  0.250  0.250  0.250
## treatment25:pair15  0.250  0.250  0.250  0.250  0.250  0.500  0.250  0.250
## treatment26:pair15  0.500  0.250  0.250  0.250  0.250  0.250  0.500  0.250
##                    t22:13 t23:13 t24:13 t25:13 t26:13 t21:14 t22:14 t23:14
## treatment21
## treatment22
## treatment23
## treatment24
## treatment25
## treatment26
## pair2
## pair3
## pair4
## pair5
## pair6
## pair7
## pair8
## pair9
## pair10
## pair11
## pair12
## pair13
## pair14
## pair15
## treatment21:pair2
## treatment22:pair2
## treatment23:pair2
## treatment24:pair2
## treatment25:pair2
## treatment26:pair2
## treatment21:pair3
## treatment22:pair3
```

```
## treatment23:pair3
## treatment24:pair3
## treatment25:pair3
## treatment26:pair3
## treatment21:pair4
## treatment22:pair4
## treatment23:pair4
## treatment24:pair4
## treatment25:pair4
## treatment26:pair4
## treatment21:pair5
## treatment22:pair5
## treatment23:pair5
## treatment24:pair5
## treatment25:pair5
## treatment26:pair5
## treatment21:pair6
## treatment22:pair6
## treatment23:pair6
## treatment24:pair6
## treatment25:pair6
## treatment26:pair6
## treatment21:pair7
## treatment22:pair7
## treatment23:pair7
## treatment24:pair7
## treatment25:pair7
## treatment26:pair7
## treatment21:pair8
## treatment22:pair8
## treatment23:pair8
## treatment24:pair8
## treatment25:pair8
## treatment26:pair8
## treatment21:pair9
## treatment22:pair9
## treatment23:pair9
## treatment24:pair9
## treatment25:pair9
## treatment26:pair9
## treatment21:pair10
## treatment22:pair10
## treatment23:pair10
## treatment24:pair10
## treatment25:pair10
## treatment26:pair10
## treatment21:pair11
## treatment22:pair11
## treatment23:pair11
## treatment24:pair11
## treatment25:pair11
## treatment26:pair11
## treatment21:pair12
## treatment22:pair12
```

```
## treatment23:pair12
## treatment24:pair12
## treatment25:pair12
## treatment26:pair12
## treatment21:pair13
## treatment22:pair13
## treatment23:pair13  0.500
## treatment24:pair13  0.500  0.500
## treatment25:pair13  0.500  0.500  0.500
## treatment26:pair13  0.500  0.500  0.500  0.500
## treatment21:pair14  0.250  0.250  0.250  0.250  0.250
## treatment22:pair14  0.500  0.250  0.250  0.250  0.250  0.500
## treatment23:pair14  0.250  0.500  0.250  0.250  0.250  0.500  0.500
## treatment24:pair14  0.250  0.250  0.500  0.250  0.250  0.500  0.500  0.500
## treatment25:pair14  0.250  0.250  0.250  0.500  0.250  0.500  0.500  0.500
## treatment26:pair14  0.250  0.250  0.250  0.250  0.500  0.500  0.500  0.500
## treatment21:pair15  0.250  0.250  0.250  0.250  0.250  0.500  0.250  0.250
## treatment22:pair15  0.500  0.250  0.250  0.250  0.250  0.250  0.500  0.250
## treatment23:pair15  0.250  0.500  0.250  0.250  0.250  0.250  0.250  0.500
## treatment24:pair15  0.250  0.250  0.500  0.250  0.250  0.250  0.250  0.250
## treatment25:pair15  0.250  0.250  0.250  0.500  0.250  0.250  0.250  0.250
## treatment26:pair15  0.250  0.250  0.250  0.250  0.500  0.250  0.250  0.250
##                    t24:14 t25:14 t26:14 t21:15 t22:15 t23:15 t24:15 t25:15
## treatment21
## treatment22
## treatment23
## treatment24
## treatment25
## treatment26
## pair2
## pair3
## pair4
## pair5
## pair6
## pair7
## pair8
## pair9
## pair10
## pair11
## pair12
## pair13
## pair14
## pair15
## treatment21:pair2
## treatment22:pair2
## treatment23:pair2
## treatment24:pair2
## treatment25:pair2
## treatment26:pair2
## treatment21:pair3
## treatment22:pair3
## treatment23:pair3
## treatment24:pair3
## treatment25:pair3
```

```
## treatment26:pair3
## treatment21:pair4
## treatment22:pair4
## treatment23:pair4
## treatment24:pair4
## treatment25:pair4
## treatment26:pair4
## treatment21:pair5
## treatment22:pair5
## treatment23:pair5
## treatment24:pair5
## treatment25:pair5
## treatment26:pair5
## treatment21:pair6
## treatment22:pair6
## treatment23:pair6
## treatment24:pair6
## treatment25:pair6
## treatment26:pair6
## treatment21:pair7
## treatment22:pair7
## treatment23:pair7
## treatment24:pair7
## treatment25:pair7
## treatment26:pair7
## treatment21:pair8
## treatment22:pair8
## treatment23:pair8
## treatment24:pair8
## treatment25:pair8
## treatment26:pair8
## treatment21:pair9
## treatment22:pair9
## treatment23:pair9
## treatment24:pair9
## treatment25:pair9
## treatment26:pair9
## treatment21:pair10
## treatment22:pair10
## treatment23:pair10
## treatment24:pair10
## treatment25:pair10
## treatment26:pair10
## treatment21:pair11
## treatment22:pair11
## treatment23:pair11
## treatment24:pair11
## treatment25:pair11
## treatment26:pair11
## treatment21:pair12
## treatment22:pair12
## treatment23:pair12
## treatment24:pair12
## treatment25:pair12
```

```
## treatment26:pair12
## treatment21:pair13
## treatment22:pair13
## treatment23:pair13
## treatment24:pair13
## treatment25:pair13
## treatment26:pair13
## treatment21:pair14
## treatment22:pair14
## treatment23:pair14
## treatment24:pair14
## treatment25:pair14  0.500
## treatment26:pair14  0.500  0.500
## treatment21:pair15  0.250  0.250  0.250
## treatment22:pair15  0.250  0.250  0.250  0.500
## treatment23:pair15  0.250  0.250  0.250  0.500  0.500
## treatment24:pair15  0.500  0.250  0.250  0.500  0.500  0.500
## treatment25:pair15  0.250  0.500  0.250  0.500  0.500  0.500  0.500
## treatment26:pair15  0.250  0.250  0.500  0.500  0.500  0.500  0.500  0.500
##
## Standardized Within-Group Residuals:
##         Min          Q1         Med          Q3         Max
## -2.15734005 -0.56337583 -0.01329857  0.48226706  2.73174374
##
## Number of Observations: 315
## Number of Groups: 21
```

2931p

```r
data<-read.table("~/Desktop/rsync/deteriorating treatments/phosphate/2931_phosphate_v2_[0].csv",h=T)
data$treatment2<-as.factor(data$treatment)
data$pair<-as.factor(data$pair)
fit2<-lme(relfitav~treatment2*pair,random=~1|plate,data)
anova(fit2)
```

```
##                  numDF denDF   F-value p-value
## (Intercept)          1   182 2607.3690  <.0001
## treatment2           6    14    0.2812  0.9364
## pair                13   182    2.1842  0.0118
## treatment2:pair     78   182    0.9727  0.5472
```

```r
summary(fit2)
```

```
## Linear mixed-effects model fit by REML
##  Data: data
##        AIC      BIC    logLik
##   388.0566 715.8681 -94.02832
##
## Random effects:
##  Formula: ~1 | plate
##         (Intercept)  Residual
```

```
## StdDev: 1.468658e-05 0.2970499
##
## Fixed effects: relfitav ~ treatment2 * pair
##                          Value Std.Error  DF   t-value p-value
## (Intercept)          0.9287644 0.1715018 182  5.415478  0.0000
## treatment21         -0.1359681 0.2425402  14 -0.560600  0.5839
## treatment22         -0.1156395 0.2425402  14 -0.476785  0.6409
## treatment23         -0.1117960 0.2425402  14 -0.460938  0.6519
## treatment24          0.0087871 0.2425402  14  0.036230  0.9716
## treatment25         -0.0334112 0.2425402  14 -0.137755  0.8924
## treatment26          0.0831450 0.2425402  14  0.342809  0.7368
## pair2               -0.0540513 0.2425402 182 -0.222855  0.8239
## pair3               -0.2163985 0.2425402 182 -0.892217  0.3735
## pair4                0.0530628 0.2425402 182  0.218779  0.8271
## pair5                0.1079635 0.2425402 182  0.445136  0.6568
## pair6                0.0505155 0.2425402 182  0.208277  0.8352
## pair7                0.2244208 0.2425402 182  0.925293  0.3560
## pair9                0.0067280 0.2425402 182  0.027740  0.9779
## pair10              -0.0164361 0.2425402 182 -0.067766  0.9460
## pair11              -0.0090081 0.2425402 182 -0.037141  0.9704
## pair12              -0.4669461 0.2425402 182 -1.925232  0.0558
## pair13               0.0503276 0.2425402 182  0.207502  0.8358
## pair14               0.1058053 0.2425402 182  0.436238  0.6632
## pair15               0.0478200 0.2425402 182  0.197163  0.8439
## treatment21:pair2    0.1331033 0.3430036 182  0.388052  0.6984
## treatment22:pair2    0.1705623 0.3430036 182  0.497261  0.6196
## treatment23:pair2    0.1248217 0.3430036 182  0.363908  0.7163
## treatment24:pair2    0.0824298 0.3430036 182  0.240318  0.8104
## treatment25:pair2    0.1052977 0.3430036 182  0.306987  0.7592
## treatment26:pair2   -0.0868392 0.3430036 182 -0.253173  0.8004
## treatment21:pair3    0.4711295 0.3430036 182  1.373541  0.1713
## treatment22:pair3    0.3980783 0.3430036 182  1.160566  0.2473
## treatment23:pair3    0.2578120 0.3430036 182  0.751630  0.4532
## treatment24:pair3    0.2131410 0.3430036 182  0.621396  0.5351
## treatment25:pair3    0.5998481 0.3430036 182  1.748810  0.0820
## treatment26:pair3    0.2972316 0.3430036 182  0.866555  0.3873
## treatment21:pair4   -0.0480803 0.3430036 182 -0.140174  0.8887
## treatment22:pair4    0.1753801 0.3430036 182  0.511307  0.6098
## treatment23:pair4   -0.1014323 0.3430036 182 -0.295718  0.7678
## treatment24:pair4   -0.2177860 0.3430036 182 -0.634938  0.5263
## treatment25:pair4    0.1365316 0.3430036 182  0.398047  0.6911
## treatment26:pair4   -0.4533049 0.3430036 182 -1.321575  0.1880
## treatment21:pair5    0.0574422 0.3430036 182  0.167468  0.8672
## treatment22:pair5   -0.1779402 0.3430036 182 -0.518771  0.6046
## treatment23:pair5    0.1223783 0.3430036 182  0.356784  0.7217
## treatment24:pair5   -0.1738195 0.3430036 182 -0.506757  0.6129
## treatment25:pair5   -0.3760250 0.3430036 182 -1.096271  0.2744
## treatment26:pair5   -0.0373905 0.3430036 182 -0.109009  0.9133
## treatment21:pair6    0.0668566 0.3430036 182  0.194915  0.8457
## treatment22:pair6    0.0140941 0.3430036 182  0.041090  0.9673
## treatment23:pair6    0.1272935 0.3430036 182  0.371114  0.7110
## treatment24:pair6   -0.0522555 0.3430036 182 -0.152347  0.8791
## treatment25:pair6    0.1698173 0.3430036 182  0.495089  0.6211
## treatment26:pair6   -0.2438475 0.3430036 182 -0.710918  0.4780
```

```
## treatment21:pair7   0.0891248 0.3430036 182  0.259836  0.7953
## treatment22:pair7   0.0142249 0.3430036 182  0.041472  0.9670
## treatment23:pair7  -0.3171413 0.3430036 182 -0.924600  0.3564
## treatment24:pair7  -0.2082477 0.3430036 182 -0.607130  0.5445
## treatment25:pair7   0.1165429 0.3430036 182  0.339772  0.7344
## treatment26:pair7  -0.2264813 0.3430036 182 -0.660288  0.5099
## treatment21:pair9   0.1523555 0.3430036 182  0.444180  0.6574
## treatment22:pair9  -0.1745678 0.3430036 182 -0.508939  0.6114
## treatment23:pair9   0.1089588 0.3430036 182  0.317661  0.7511
## treatment24:pair9   0.1677917 0.3430036 182  0.489183  0.6253
## treatment25:pair9   0.3366468 0.3430036 182  0.981467  0.3277
## treatment26:pair9   0.0990915 0.3430036 182  0.288893  0.7730
## treatment21:pair10  0.1027705 0.3430036 182  0.299619  0.7648
## treatment22:pair10 -0.1465012 0.3430036 182 -0.427113  0.6698
## treatment23:pair10  0.2608290 0.3430036 182  0.760426  0.4480
## treatment24:pair10 -0.1831873 0.3430036 182 -0.534068  0.5939
## treatment25:pair10 -0.0783176 0.3430036 182 -0.228329  0.8196
## treatment26:pair10  0.0010178 0.3430036 182  0.002967  0.9976
## treatment21:pair11  0.0043669 0.3430036 182  0.012731  0.9899
## treatment22:pair11  0.1827620 0.3430036 182  0.532828  0.5948
## treatment23:pair11 -0.0350581 0.3430036 182 -0.102209  0.9187
## treatment24:pair11  0.0768163 0.3430036 182  0.223952  0.8230
## treatment25:pair11 -0.2106667 0.3430036 182 -0.614182  0.5399
## treatment26:pair11 -0.5387601 0.3430036 182 -1.570712  0.1180
## treatment21:pair12  0.2223897 0.3430036 182  0.648360  0.5176
## treatment22:pair12  0.3100666 0.3430036 182  0.903975  0.3672
## treatment23:pair12  0.1516049 0.3430036 182  0.441992  0.6590
## treatment24:pair12  0.5133700 0.3430036 182  1.496690  0.1362
## treatment25:pair12  0.3764427 0.3430036 182  1.097489  0.2739
## treatment26:pair12  0.4048985 0.3430036 182  1.180449  0.2394
## treatment21:pair13  0.1265884 0.3430036 182  0.369059  0.7125
## treatment22:pair13  0.2498945 0.3430036 182  0.728548  0.4672
## treatment23:pair13  0.0985237 0.3430036 182  0.287238  0.7743
## treatment24:pair13 -0.1577845 0.3430036 182 -0.460008  0.6461
## treatment25:pair13 -0.2038368 0.3430036 182 -0.594270  0.5531
## treatment26:pair13 -0.0540327 0.3430036 182 -0.157528  0.8750
## treatment21:pair14 -0.1529131 0.3430036 182 -0.445806  0.6563
## treatment22:pair14 -0.0283336 0.3430036 182 -0.082604  0.9343
## treatment23:pair14 -0.2287492 0.3430036 182 -0.666900  0.5057
## treatment24:pair14 -0.0505002 0.3430036 182 -0.147229  0.8831
## treatment25:pair14 -0.5470290 0.3430036 182 -1.594820  0.1125
## treatment26:pair14 -0.6363946 0.3430036 182 -1.855358  0.0652
## treatment21:pair15 -0.0664240 0.3430036 182 -0.193654  0.8467
## treatment22:pair15  0.1356546 0.3430036 182  0.395490  0.6929
## treatment23:pair15  0.0359156 0.3430036 182  0.104709  0.9167
## treatment24:pair15 -0.2510630 0.3430036 182 -0.731954  0.4651
## treatment25:pair15 -0.4881259 0.3430036 182 -1.423093  0.1564
## treatment26:pair15 -0.3534401 0.3430036 182 -1.030427  0.3042
##  Correlation:
##                    (Intr) trtm21 trtm22 trtm23 trtm24 trtm25 trtm26 pair2
## treatment21        -0.707
## treatment22        -0.707  0.500
## treatment23        -0.707  0.500  0.500
## treatment24        -0.707  0.500  0.500  0.500
```

```
## treatment25        -0.707  0.500  0.500  0.500  0.500
## treatment26        -0.707  0.500  0.500  0.500  0.500  0.500
## pair2              -0.707  0.500  0.500  0.500  0.500  0.500  0.500
## pair3              -0.707  0.500  0.500  0.500  0.500  0.500  0.500  0.500
## pair4              -0.707  0.500  0.500  0.500  0.500  0.500  0.500  0.500
## pair5              -0.707  0.500  0.500  0.500  0.500  0.500  0.500  0.500
## pair6              -0.707  0.500  0.500  0.500  0.500  0.500  0.500  0.500
## pair7              -0.707  0.500  0.500  0.500  0.500  0.500  0.500  0.500
## pair9              -0.707  0.500  0.500  0.500  0.500  0.500  0.500  0.500
## pair10             -0.707  0.500  0.500  0.500  0.500  0.500  0.500  0.500
## pair11             -0.707  0.500  0.500  0.500  0.500  0.500  0.500  0.500
## pair12             -0.707  0.500  0.500  0.500  0.500  0.500  0.500  0.500
## pair13             -0.707  0.500  0.500  0.500  0.500  0.500  0.500  0.500
## pair14             -0.707  0.500  0.500  0.500  0.500  0.500  0.500  0.500
## pair15             -0.707  0.500  0.500  0.500  0.500  0.500  0.500  0.500
## treatment21:pair2   0.500 -0.707 -0.354 -0.354 -0.354 -0.354 -0.354 -0.707
## treatment22:pair2   0.500 -0.354 -0.707 -0.354 -0.354 -0.354 -0.354 -0.707
## treatment23:pair2   0.500 -0.354 -0.354 -0.707 -0.354 -0.354 -0.354 -0.707
## treatment24:pair2   0.500 -0.354 -0.354 -0.354 -0.707 -0.354 -0.354 -0.707
## treatment25:pair2   0.500 -0.354 -0.354 -0.354 -0.354 -0.707 -0.354 -0.707
## treatment26:pair2   0.500 -0.354 -0.354 -0.354 -0.354 -0.354 -0.707 -0.707
## treatment21:pair3   0.500 -0.707 -0.354 -0.354 -0.354 -0.354 -0.354 -0.354
## treatment22:pair3   0.500 -0.354 -0.707 -0.354 -0.354 -0.354 -0.354 -0.354
## treatment23:pair3   0.500 -0.354 -0.354 -0.707 -0.354 -0.354 -0.354 -0.354
## treatment24:pair3   0.500 -0.354 -0.354 -0.354 -0.707 -0.354 -0.354 -0.354
## treatment25:pair3   0.500 -0.354 -0.354 -0.354 -0.354 -0.707 -0.354 -0.354
## treatment26:pair3   0.500 -0.354 -0.354 -0.354 -0.354 -0.354 -0.707 -0.354
## treatment21:pair4   0.500 -0.707 -0.354 -0.354 -0.354 -0.354 -0.354 -0.354
## treatment22:pair4   0.500 -0.354 -0.707 -0.354 -0.354 -0.354 -0.354 -0.354
## treatment23:pair4   0.500 -0.354 -0.354 -0.707 -0.354 -0.354 -0.354 -0.354
## treatment24:pair4   0.500 -0.354 -0.354 -0.354 -0.707 -0.354 -0.354 -0.354
## treatment25:pair4   0.500 -0.354 -0.354 -0.354 -0.354 -0.707 -0.354 -0.354
## treatment26:pair4   0.500 -0.354 -0.354 -0.354 -0.354 -0.354 -0.707 -0.354
## treatment21:pair5   0.500 -0.707 -0.354 -0.354 -0.354 -0.354 -0.354 -0.354
## treatment22:pair5   0.500 -0.354 -0.707 -0.354 -0.354 -0.354 -0.354 -0.354
## treatment23:pair5   0.500 -0.354 -0.354 -0.707 -0.354 -0.354 -0.354 -0.354
## treatment24:pair5   0.500 -0.354 -0.354 -0.354 -0.707 -0.354 -0.354 -0.354
## treatment25:pair5   0.500 -0.354 -0.354 -0.354 -0.354 -0.707 -0.354 -0.354
## treatment26:pair5   0.500 -0.354 -0.354 -0.354 -0.354 -0.354 -0.707 -0.354
## treatment21:pair6   0.500 -0.707 -0.354 -0.354 -0.354 -0.354 -0.354 -0.354
## treatment22:pair6   0.500 -0.354 -0.707 -0.354 -0.354 -0.354 -0.354 -0.354
## treatment23:pair6   0.500 -0.354 -0.354 -0.707 -0.354 -0.354 -0.354 -0.354
## treatment24:pair6   0.500 -0.354 -0.354 -0.354 -0.707 -0.354 -0.354 -0.354
## treatment25:pair6   0.500 -0.354 -0.354 -0.354 -0.354 -0.707 -0.354 -0.354
## treatment26:pair6   0.500 -0.354 -0.354 -0.354 -0.354 -0.354 -0.707 -0.354
## treatment21:pair7   0.500 -0.707 -0.354 -0.354 -0.354 -0.354 -0.354 -0.354
## treatment22:pair7   0.500 -0.354 -0.707 -0.354 -0.354 -0.354 -0.354 -0.354
## treatment23:pair7   0.500 -0.354 -0.354 -0.707 -0.354 -0.354 -0.354 -0.354
## treatment24:pair7   0.500 -0.354 -0.354 -0.354 -0.707 -0.354 -0.354 -0.354
## treatment25:pair7   0.500 -0.354 -0.354 -0.354 -0.354 -0.707 -0.354 -0.354
## treatment26:pair7   0.500 -0.354 -0.354 -0.354 -0.354 -0.354 -0.707 -0.354
## treatment21:pair9   0.500 -0.707 -0.354 -0.354 -0.354 -0.354 -0.354 -0.354
## treatment22:pair9   0.500 -0.354 -0.707 -0.354 -0.354 -0.354 -0.354 -0.354
## treatment23:pair9   0.500 -0.354 -0.354 -0.707 -0.354 -0.354 -0.354 -0.354
```

```
## treatment24:pair9   0.500 -0.354 -0.354 -0.354 -0.707 -0.354 -0.354 -0.354
## treatment25:pair9   0.500 -0.354 -0.354 -0.354 -0.354 -0.707 -0.354 -0.354
## treatment26:pair9   0.500 -0.354 -0.354 -0.354 -0.354 -0.354 -0.707 -0.354
## treatment21:pair10  0.500 -0.707 -0.354 -0.354 -0.354 -0.354 -0.354 -0.354
## treatment22:pair10  0.500 -0.354 -0.707 -0.354 -0.354 -0.354 -0.354 -0.354
## treatment23:pair10  0.500 -0.354 -0.354 -0.707 -0.354 -0.354 -0.354 -0.354
## treatment24:pair10  0.500 -0.354 -0.354 -0.354 -0.707 -0.354 -0.354 -0.354
## treatment25:pair10  0.500 -0.354 -0.354 -0.354 -0.354 -0.707 -0.354 -0.354
## treatment26:pair10  0.500 -0.354 -0.354 -0.354 -0.354 -0.354 -0.707 -0.354
## treatment21:pair11  0.500 -0.707 -0.354 -0.354 -0.354 -0.354 -0.354 -0.354
## treatment22:pair11  0.500 -0.354 -0.707 -0.354 -0.354 -0.354 -0.354 -0.354
## treatment23:pair11  0.500 -0.354 -0.354 -0.707 -0.354 -0.354 -0.354 -0.354
## treatment24:pair11  0.500 -0.354 -0.354 -0.354 -0.707 -0.354 -0.354 -0.354
## treatment25:pair11  0.500 -0.354 -0.354 -0.354 -0.354 -0.707 -0.354 -0.354
## treatment26:pair11  0.500 -0.354 -0.354 -0.354 -0.354 -0.354 -0.707 -0.354
## treatment21:pair12  0.500 -0.707 -0.354 -0.354 -0.354 -0.354 -0.354 -0.354
## treatment22:pair12  0.500 -0.354 -0.707 -0.354 -0.354 -0.354 -0.354 -0.354
## treatment23:pair12  0.500 -0.354 -0.354 -0.707 -0.354 -0.354 -0.354 -0.354
## treatment24:pair12  0.500 -0.354 -0.354 -0.354 -0.707 -0.354 -0.354 -0.354
## treatment25:pair12  0.500 -0.354 -0.354 -0.354 -0.354 -0.707 -0.354 -0.354
## treatment26:pair12  0.500 -0.354 -0.354 -0.354 -0.354 -0.354 -0.707 -0.354
## treatment21:pair13  0.500 -0.707 -0.354 -0.354 -0.354 -0.354 -0.354 -0.354
## treatment22:pair13  0.500 -0.354 -0.707 -0.354 -0.354 -0.354 -0.354 -0.354
## treatment23:pair13  0.500 -0.354 -0.354 -0.707 -0.354 -0.354 -0.354 -0.354
## treatment24:pair13  0.500 -0.354 -0.354 -0.354 -0.707 -0.354 -0.354 -0.354
## treatment25:pair13  0.500 -0.354 -0.354 -0.354 -0.354 -0.707 -0.354 -0.354
## treatment26:pair13  0.500 -0.354 -0.354 -0.354 -0.354 -0.354 -0.707 -0.354
## treatment21:pair14  0.500 -0.707 -0.354 -0.354 -0.354 -0.354 -0.354 -0.354
## treatment22:pair14  0.500 -0.354 -0.707 -0.354 -0.354 -0.354 -0.354 -0.354
## treatment23:pair14  0.500 -0.354 -0.354 -0.707 -0.354 -0.354 -0.354 -0.354
## treatment24:pair14  0.500 -0.354 -0.354 -0.354 -0.707 -0.354 -0.354 -0.354
## treatment25:pair14  0.500 -0.354 -0.354 -0.354 -0.354 -0.707 -0.354 -0.354
## treatment26:pair14  0.500 -0.354 -0.354 -0.354 -0.354 -0.354 -0.707 -0.354
## treatment21:pair15  0.500 -0.707 -0.354 -0.354 -0.354 -0.354 -0.354 -0.354
## treatment22:pair15  0.500 -0.354 -0.707 -0.354 -0.354 -0.354 -0.354 -0.354
## treatment23:pair15  0.500 -0.354 -0.354 -0.707 -0.354 -0.354 -0.354 -0.354
## treatment24:pair15  0.500 -0.354 -0.354 -0.354 -0.707 -0.354 -0.354 -0.354
## treatment25:pair15  0.500 -0.354 -0.354 -0.354 -0.354 -0.707 -0.354 -0.354
## treatment26:pair15  0.500 -0.354 -0.354 -0.354 -0.354 -0.354 -0.707 -0.354
##                    pair3  pair4  pair5  pair6  pair7  pair9  pair10 pair11
## treatment21
## treatment22
## treatment23
## treatment24
## treatment25
## treatment26
## pair2
## pair3
## pair4               0.500
## pair5               0.500  0.500
## pair6               0.500  0.500  0.500
## pair7               0.500  0.500  0.500  0.500
## pair9               0.500  0.500  0.500  0.500  0.500
## pair10              0.500  0.500  0.500  0.500  0.500  0.500
```

```
## pair11               0.500  0.500  0.500  0.500  0.500  0.500  0.500
## pair12               0.500  0.500  0.500  0.500  0.500  0.500  0.500  0.500
## pair13               0.500  0.500  0.500  0.500  0.500  0.500  0.500  0.500
## pair14               0.500  0.500  0.500  0.500  0.500  0.500  0.500  0.500
## pair15               0.500  0.500  0.500  0.500  0.500  0.500  0.500  0.500
## treatment21:pair2  -0.354 -0.354 -0.354 -0.354 -0.354 -0.354 -0.354 -0.354
## treatment22:pair2  -0.354 -0.354 -0.354 -0.354 -0.354 -0.354 -0.354 -0.354
## treatment23:pair2  -0.354 -0.354 -0.354 -0.354 -0.354 -0.354 -0.354 -0.354
## treatment24:pair2  -0.354 -0.354 -0.354 -0.354 -0.354 -0.354 -0.354 -0.354
## treatment25:pair2  -0.354 -0.354 -0.354 -0.354 -0.354 -0.354 -0.354 -0.354
## treatment26:pair2  -0.354 -0.354 -0.354 -0.354 -0.354 -0.354 -0.354 -0.354
## treatment21:pair3  -0.707 -0.354 -0.354 -0.354 -0.354 -0.354 -0.354 -0.354
## treatment22:pair3  -0.707 -0.354 -0.354 -0.354 -0.354 -0.354 -0.354 -0.354
## treatment23:pair3  -0.707 -0.354 -0.354 -0.354 -0.354 -0.354 -0.354 -0.354
## treatment24:pair3  -0.707 -0.354 -0.354 -0.354 -0.354 -0.354 -0.354 -0.354
## treatment25:pair3  -0.707 -0.354 -0.354 -0.354 -0.354 -0.354 -0.354 -0.354
## treatment26:pair3  -0.707 -0.354 -0.354 -0.354 -0.354 -0.354 -0.354 -0.354
## treatment21:pair4  -0.354 -0.707 -0.354 -0.354 -0.354 -0.354 -0.354 -0.354
## treatment22:pair4  -0.354 -0.707 -0.354 -0.354 -0.354 -0.354 -0.354 -0.354
## treatment23:pair4  -0.354 -0.707 -0.354 -0.354 -0.354 -0.354 -0.354 -0.354
## treatment24:pair4  -0.354 -0.707 -0.354 -0.354 -0.354 -0.354 -0.354 -0.354
## treatment25:pair4  -0.354 -0.707 -0.354 -0.354 -0.354 -0.354 -0.354 -0.354
## treatment26:pair4  -0.354 -0.707 -0.354 -0.354 -0.354 -0.354 -0.354 -0.354
## treatment21:pair5  -0.354 -0.354 -0.707 -0.354 -0.354 -0.354 -0.354 -0.354
## treatment22:pair5  -0.354 -0.354 -0.707 -0.354 -0.354 -0.354 -0.354 -0.354
## treatment23:pair5  -0.354 -0.354 -0.707 -0.354 -0.354 -0.354 -0.354 -0.354
## treatment24:pair5  -0.354 -0.354 -0.707 -0.354 -0.354 -0.354 -0.354 -0.354
## treatment25:pair5  -0.354 -0.354 -0.707 -0.354 -0.354 -0.354 -0.354 -0.354
## treatment26:pair5  -0.354 -0.354 -0.707 -0.354 -0.354 -0.354 -0.354 -0.354
## treatment21:pair6  -0.354 -0.354 -0.354 -0.707 -0.354 -0.354 -0.354 -0.354
## treatment22:pair6  -0.354 -0.354 -0.354 -0.707 -0.354 -0.354 -0.354 -0.354
## treatment23:pair6  -0.354 -0.354 -0.354 -0.707 -0.354 -0.354 -0.354 -0.354
## treatment24:pair6  -0.354 -0.354 -0.354 -0.707 -0.354 -0.354 -0.354 -0.354
## treatment25:pair6  -0.354 -0.354 -0.354 -0.707 -0.354 -0.354 -0.354 -0.354
## treatment26:pair6  -0.354 -0.354 -0.354 -0.707 -0.354 -0.354 -0.354 -0.354
## treatment21:pair7  -0.354 -0.354 -0.354 -0.354 -0.707 -0.354 -0.354 -0.354
## treatment22:pair7  -0.354 -0.354 -0.354 -0.354 -0.707 -0.354 -0.354 -0.354
## treatment23:pair7  -0.354 -0.354 -0.354 -0.354 -0.707 -0.354 -0.354 -0.354
## treatment24:pair7  -0.354 -0.354 -0.354 -0.354 -0.707 -0.354 -0.354 -0.354
## treatment25:pair7  -0.354 -0.354 -0.354 -0.354 -0.707 -0.354 -0.354 -0.354
## treatment26:pair7  -0.354 -0.354 -0.354 -0.354 -0.707 -0.354 -0.354 -0.354
## treatment21:pair9  -0.354 -0.354 -0.354 -0.354 -0.354 -0.707 -0.354 -0.354
## treatment22:pair9  -0.354 -0.354 -0.354 -0.354 -0.354 -0.707 -0.354 -0.354
## treatment23:pair9  -0.354 -0.354 -0.354 -0.354 -0.354 -0.707 -0.354 -0.354
## treatment24:pair9  -0.354 -0.354 -0.354 -0.354 -0.354 -0.707 -0.354 -0.354
## treatment25:pair9  -0.354 -0.354 -0.354 -0.354 -0.354 -0.707 -0.354 -0.354
## treatment26:pair9  -0.354 -0.354 -0.354 -0.354 -0.354 -0.707 -0.354 -0.354
## treatment21:pair10 -0.354 -0.354 -0.354 -0.354 -0.354 -0.354 -0.707 -0.354
## treatment22:pair10 -0.354 -0.354 -0.354 -0.354 -0.354 -0.354 -0.707 -0.354
## treatment23:pair10 -0.354 -0.354 -0.354 -0.354 -0.354 -0.354 -0.707 -0.354
## treatment24:pair10 -0.354 -0.354 -0.354 -0.354 -0.354 -0.354 -0.707 -0.354
## treatment25:pair10 -0.354 -0.354 -0.354 -0.354 -0.354 -0.354 -0.707 -0.354
## treatment26:pair10 -0.354 -0.354 -0.354 -0.354 -0.354 -0.354 -0.707 -0.354
## treatment21:pair11 -0.354 -0.354 -0.354 -0.354 -0.354 -0.354 -0.354 -0.707
```

```
## treatment22:pair11 -0.354 -0.354 -0.354 -0.354 -0.354 -0.354 -0.354 -0.707
## treatment23:pair11 -0.354 -0.354 -0.354 -0.354 -0.354 -0.354 -0.354 -0.707
## treatment24:pair11 -0.354 -0.354 -0.354 -0.354 -0.354 -0.354 -0.354 -0.707
## treatment25:pair11 -0.354 -0.354 -0.354 -0.354 -0.354 -0.354 -0.354 -0.707
## treatment26:pair11 -0.354 -0.354 -0.354 -0.354 -0.354 -0.354 -0.354 -0.707
## treatment21:pair12 -0.354 -0.354 -0.354 -0.354 -0.354 -0.354 -0.354 -0.354
## treatment22:pair12 -0.354 -0.354 -0.354 -0.354 -0.354 -0.354 -0.354 -0.354
## treatment23:pair12 -0.354 -0.354 -0.354 -0.354 -0.354 -0.354 -0.354 -0.354
## treatment24:pair12 -0.354 -0.354 -0.354 -0.354 -0.354 -0.354 -0.354 -0.354
## treatment25:pair12 -0.354 -0.354 -0.354 -0.354 -0.354 -0.354 -0.354 -0.354
## treatment26:pair12 -0.354 -0.354 -0.354 -0.354 -0.354 -0.354 -0.354 -0.354
## treatment21:pair13 -0.354 -0.354 -0.354 -0.354 -0.354 -0.354 -0.354 -0.354
## treatment22:pair13 -0.354 -0.354 -0.354 -0.354 -0.354 -0.354 -0.354 -0.354
## treatment23:pair13 -0.354 -0.354 -0.354 -0.354 -0.354 -0.354 -0.354 -0.354
## treatment24:pair13 -0.354 -0.354 -0.354 -0.354 -0.354 -0.354 -0.354 -0.354
## treatment25:pair13 -0.354 -0.354 -0.354 -0.354 -0.354 -0.354 -0.354 -0.354
## treatment26:pair13 -0.354 -0.354 -0.354 -0.354 -0.354 -0.354 -0.354 -0.354
## treatment21:pair14 -0.354 -0.354 -0.354 -0.354 -0.354 -0.354 -0.354 -0.354
## treatment22:pair14 -0.354 -0.354 -0.354 -0.354 -0.354 -0.354 -0.354 -0.354
## treatment23:pair14 -0.354 -0.354 -0.354 -0.354 -0.354 -0.354 -0.354 -0.354
## treatment24:pair14 -0.354 -0.354 -0.354 -0.354 -0.354 -0.354 -0.354 -0.354
## treatment25:pair14 -0.354 -0.354 -0.354 -0.354 -0.354 -0.354 -0.354 -0.354
## treatment26:pair14 -0.354 -0.354 -0.354 -0.354 -0.354 -0.354 -0.354 -0.354
## treatment21:pair15 -0.354 -0.354 -0.354 -0.354 -0.354 -0.354 -0.354 -0.354
## treatment22:pair15 -0.354 -0.354 -0.354 -0.354 -0.354 -0.354 -0.354 -0.354
## treatment23:pair15 -0.354 -0.354 -0.354 -0.354 -0.354 -0.354 -0.354 -0.354
## treatment24:pair15 -0.354 -0.354 -0.354 -0.354 -0.354 -0.354 -0.354 -0.354
## treatment25:pair15 -0.354 -0.354 -0.354 -0.354 -0.354 -0.354 -0.354 -0.354
## treatment26:pair15 -0.354 -0.354 -0.354 -0.354 -0.354 -0.354 -0.354 -0.354
##                    pair12 pair13 pair14 pair15 tr21:2 tr22:2 tr23:2 tr24:2
## treatment21
## treatment22
## treatment23
## treatment24
## treatment25
## treatment26
## pair2
## pair3
## pair4
## pair5
## pair6
## pair7
## pair9
## pair10
## pair11
## pair12
## pair13              0.500
## pair14              0.500  0.500
## pair15              0.500  0.500  0.500
## treatment21:pair2  -0.354 -0.354 -0.354 -0.354
## treatment22:pair2  -0.354 -0.354 -0.354 -0.354  0.500
## treatment23:pair2  -0.354 -0.354 -0.354 -0.354  0.500  0.500
## treatment24:pair2  -0.354 -0.354 -0.354 -0.354  0.500  0.500  0.500
## treatment25:pair2  -0.354 -0.354 -0.354 -0.354  0.500  0.500  0.500  0.500
```

```
## treatment26:pair2  -0.354 -0.354 -0.354 -0.354  0.500  0.500  0.500  0.500
## treatment21:pair3  -0.354 -0.354 -0.354 -0.354  0.500  0.250  0.250  0.250
## treatment22:pair3  -0.354 -0.354 -0.354 -0.354  0.250  0.500  0.250  0.250
## treatment23:pair3  -0.354 -0.354 -0.354 -0.354  0.250  0.250  0.500  0.250
## treatment24:pair3  -0.354 -0.354 -0.354 -0.354  0.250  0.250  0.250  0.500
## treatment25:pair3  -0.354 -0.354 -0.354 -0.354  0.250  0.250  0.250  0.250
## treatment26:pair3  -0.354 -0.354 -0.354 -0.354  0.250  0.250  0.250  0.250
## treatment21:pair4  -0.354 -0.354 -0.354 -0.354  0.500  0.250  0.250  0.250
## treatment22:pair4  -0.354 -0.354 -0.354 -0.354  0.250  0.500  0.250  0.250
## treatment23:pair4  -0.354 -0.354 -0.354 -0.354  0.250  0.250  0.500  0.250
## treatment24:pair4  -0.354 -0.354 -0.354 -0.354  0.250  0.250  0.250  0.500
## treatment25:pair4  -0.354 -0.354 -0.354 -0.354  0.250  0.250  0.250  0.250
## treatment26:pair4  -0.354 -0.354 -0.354 -0.354  0.250  0.250  0.250  0.250
## treatment21:pair5  -0.354 -0.354 -0.354 -0.354  0.500  0.250  0.250  0.250
## treatment22:pair5  -0.354 -0.354 -0.354 -0.354  0.250  0.500  0.250  0.250
## treatment23:pair5  -0.354 -0.354 -0.354 -0.354  0.250  0.250  0.500  0.250
## treatment24:pair5  -0.354 -0.354 -0.354 -0.354  0.250  0.250  0.250  0.500
## treatment25:pair5  -0.354 -0.354 -0.354 -0.354  0.250  0.250  0.250  0.250
## treatment26:pair5  -0.354 -0.354 -0.354 -0.354  0.250  0.250  0.250  0.250
## treatment21:pair6  -0.354 -0.354 -0.354 -0.354  0.500  0.250  0.250  0.250
## treatment22:pair6  -0.354 -0.354 -0.354 -0.354  0.250  0.500  0.250  0.250
## treatment23:pair6  -0.354 -0.354 -0.354 -0.354  0.250  0.250  0.500  0.250
## treatment24:pair6  -0.354 -0.354 -0.354 -0.354  0.250  0.250  0.250  0.500
## treatment25:pair6  -0.354 -0.354 -0.354 -0.354  0.250  0.250  0.250  0.250
## treatment26:pair6  -0.354 -0.354 -0.354 -0.354  0.250  0.250  0.250  0.250
## treatment21:pair7  -0.354 -0.354 -0.354 -0.354  0.500  0.250  0.250  0.250
## treatment22:pair7  -0.354 -0.354 -0.354 -0.354  0.250  0.500  0.250  0.250
## treatment23:pair7  -0.354 -0.354 -0.354 -0.354  0.250  0.250  0.500  0.250
## treatment24:pair7  -0.354 -0.354 -0.354 -0.354  0.250  0.250  0.250  0.500
## treatment25:pair7  -0.354 -0.354 -0.354 -0.354  0.250  0.250  0.250  0.250
## treatment26:pair7  -0.354 -0.354 -0.354 -0.354  0.250  0.250  0.250  0.250
## treatment21:pair9  -0.354 -0.354 -0.354 -0.354  0.500  0.250  0.250  0.250
## treatment22:pair9  -0.354 -0.354 -0.354 -0.354  0.250  0.500  0.250  0.250
## treatment23:pair9  -0.354 -0.354 -0.354 -0.354  0.250  0.250  0.500  0.250
## treatment24:pair9  -0.354 -0.354 -0.354 -0.354  0.250  0.250  0.250  0.500
## treatment25:pair9  -0.354 -0.354 -0.354 -0.354  0.250  0.250  0.250  0.250
## treatment26:pair9  -0.354 -0.354 -0.354 -0.354  0.250  0.250  0.250  0.250
## treatment21:pair10 -0.354 -0.354 -0.354 -0.354  0.500  0.250  0.250  0.250
## treatment22:pair10 -0.354 -0.354 -0.354 -0.354  0.250  0.500  0.250  0.250
## treatment23:pair10 -0.354 -0.354 -0.354 -0.354  0.250  0.250  0.500  0.250
## treatment24:pair10 -0.354 -0.354 -0.354 -0.354  0.250  0.250  0.250  0.500
## treatment25:pair10 -0.354 -0.354 -0.354 -0.354  0.250  0.250  0.250  0.250
## treatment26:pair10 -0.354 -0.354 -0.354 -0.354  0.250  0.250  0.250  0.250
## treatment21:pair11 -0.354 -0.354 -0.354 -0.354  0.500  0.250  0.250  0.250
## treatment22:pair11 -0.354 -0.354 -0.354 -0.354  0.250  0.500  0.250  0.250
## treatment23:pair11 -0.354 -0.354 -0.354 -0.354  0.250  0.250  0.500  0.250
## treatment24:pair11 -0.354 -0.354 -0.354 -0.354  0.250  0.250  0.250  0.500
## treatment25:pair11 -0.354 -0.354 -0.354 -0.354  0.250  0.250  0.250  0.250
## treatment26:pair11 -0.354 -0.354 -0.354 -0.354  0.250  0.250  0.250  0.250
## treatment21:pair12 -0.707 -0.354 -0.354 -0.354  0.500  0.250  0.250  0.250
## treatment22:pair12 -0.707 -0.354 -0.354 -0.354  0.250  0.500  0.250  0.250
## treatment23:pair12 -0.707 -0.354 -0.354 -0.354  0.250  0.250  0.500  0.250
## treatment24:pair12 -0.707 -0.354 -0.354 -0.354  0.250  0.250  0.250  0.500
## treatment25:pair12 -0.707 -0.354 -0.354 -0.354  0.250  0.250  0.250  0.250
```

```
## treatment26:pair12 -0.707 -0.354 -0.354 -0.354  0.250  0.250  0.250  0.250
## treatment21:pair13 -0.354 -0.707 -0.354 -0.354  0.500  0.250  0.250  0.250
## treatment22:pair13 -0.354 -0.707 -0.354 -0.354  0.250  0.500  0.250  0.250
## treatment23:pair13 -0.354 -0.707 -0.354 -0.354  0.250  0.250  0.500  0.250
## treatment24:pair13 -0.354 -0.707 -0.354 -0.354  0.250  0.250  0.250  0.500
## treatment25:pair13 -0.354 -0.707 -0.354 -0.354  0.250  0.250  0.250  0.250
## treatment26:pair13 -0.354 -0.707 -0.354 -0.354  0.250  0.250  0.250  0.250
## treatment21:pair14 -0.354 -0.354 -0.707 -0.354  0.500  0.250  0.250  0.250
## treatment22:pair14 -0.354 -0.354 -0.707 -0.354  0.250  0.500  0.250  0.250
## treatment23:pair14 -0.354 -0.354 -0.707 -0.354  0.250  0.250  0.500  0.250
## treatment24:pair14 -0.354 -0.354 -0.707 -0.354  0.250  0.250  0.250  0.500
## treatment25:pair14 -0.354 -0.354 -0.707 -0.354  0.250  0.250  0.250  0.250
## treatment26:pair14 -0.354 -0.354 -0.707 -0.354  0.250  0.250  0.250  0.250
## treatment21:pair15 -0.354 -0.354 -0.354 -0.707  0.500  0.250  0.250  0.250
## treatment22:pair15 -0.354 -0.354 -0.354 -0.707  0.250  0.500  0.250  0.250
## treatment23:pair15 -0.354 -0.354 -0.354 -0.707  0.250  0.250  0.500  0.250
## treatment24:pair15 -0.354 -0.354 -0.354 -0.707  0.250  0.250  0.250  0.500
## treatment25:pair15 -0.354 -0.354 -0.354 -0.707  0.250  0.250  0.250  0.250
## treatment26:pair15 -0.354 -0.354 -0.354 -0.707  0.250  0.250  0.250  0.250
##                    tr25:2 tr26:2 tr21:3 tr22:3 tr23:3 tr24:3 tr25:3 tr26:3
## treatment21
## treatment22
## treatment23
## treatment24
## treatment25
## treatment26
## pair2
## pair3
## pair4
## pair5
## pair6
## pair7
## pair9
## pair10
## pair11
## pair12
## pair13
## pair14
## pair15
## treatment21:pair2
## treatment22:pair2
## treatment23:pair2
## treatment24:pair2
## treatment25:pair2
## treatment26:pair2   0.500
## treatment21:pair3   0.250  0.250
## treatment22:pair3   0.250  0.250  0.500
## treatment23:pair3   0.250  0.250  0.500  0.500
## treatment24:pair3   0.250  0.250  0.500  0.500  0.500
## treatment25:pair3   0.500  0.250  0.500  0.500  0.500  0.500
## treatment26:pair3   0.250  0.500  0.500  0.500  0.500  0.500  0.500
## treatment21:pair4   0.250  0.250  0.500  0.250  0.250  0.250  0.250  0.250
## treatment22:pair4   0.250  0.250  0.250  0.500  0.250  0.250  0.250  0.250
## treatment23:pair4   0.250  0.250  0.250  0.250  0.500  0.250  0.250  0.250
```

```
## treatment24:pair4   0.250  0.250  0.250  0.250  0.250  0.500  0.250  0.250
## treatment25:pair4   0.500  0.250  0.250  0.250  0.250  0.250  0.500  0.250
## treatment26:pair4   0.250  0.500  0.250  0.250  0.250  0.250  0.250  0.500
## treatment21:pair5   0.250  0.250  0.500  0.250  0.250  0.250  0.250  0.250
## treatment22:pair5   0.250  0.250  0.250  0.500  0.250  0.250  0.250  0.250
## treatment23:pair5   0.250  0.250  0.250  0.250  0.500  0.250  0.250  0.250
## treatment24:pair5   0.250  0.250  0.250  0.250  0.250  0.500  0.250  0.250
## treatment25:pair5   0.500  0.250  0.250  0.250  0.250  0.250  0.500  0.250
## treatment26:pair5   0.250  0.500  0.250  0.250  0.250  0.250  0.250  0.500
## treatment21:pair6   0.250  0.250  0.500  0.250  0.250  0.250  0.250  0.250
## treatment22:pair6   0.250  0.250  0.250  0.500  0.250  0.250  0.250  0.250
## treatment23:pair6   0.250  0.250  0.250  0.250  0.500  0.250  0.250  0.250
## treatment24:pair6   0.250  0.250  0.250  0.250  0.250  0.500  0.250  0.250
## treatment25:pair6   0.500  0.250  0.250  0.250  0.250  0.250  0.500  0.250
## treatment26:pair6   0.250  0.500  0.250  0.250  0.250  0.250  0.250  0.500
## treatment21:pair7   0.250  0.250  0.500  0.250  0.250  0.250  0.250  0.250
## treatment22:pair7   0.250  0.250  0.250  0.500  0.250  0.250  0.250  0.250
## treatment23:pair7   0.250  0.250  0.250  0.250  0.500  0.250  0.250  0.250
## treatment24:pair7   0.250  0.250  0.250  0.250  0.250  0.500  0.250  0.250
## treatment25:pair7   0.500  0.250  0.250  0.250  0.250  0.250  0.500  0.250
## treatment26:pair7   0.250  0.500  0.250  0.250  0.250  0.250  0.250  0.500
## treatment21:pair9   0.250  0.250  0.500  0.250  0.250  0.250  0.250  0.250
## treatment22:pair9   0.250  0.250  0.250  0.500  0.250  0.250  0.250  0.250
## treatment23:pair9   0.250  0.250  0.250  0.250  0.500  0.250  0.250  0.250
## treatment24:pair9   0.250  0.250  0.250  0.250  0.250  0.500  0.250  0.250
## treatment25:pair9   0.500  0.250  0.250  0.250  0.250  0.250  0.500  0.250
## treatment26:pair9   0.250  0.500  0.250  0.250  0.250  0.250  0.250  0.500
## treatment21:pair10  0.250  0.250  0.500  0.250  0.250  0.250  0.250  0.250
## treatment22:pair10  0.250  0.250  0.250  0.500  0.250  0.250  0.250  0.250
## treatment23:pair10  0.250  0.250  0.250  0.250  0.500  0.250  0.250  0.250
## treatment24:pair10  0.250  0.250  0.250  0.250  0.250  0.500  0.250  0.250
## treatment25:pair10  0.500  0.250  0.250  0.250  0.250  0.250  0.500  0.250
## treatment26:pair10  0.250  0.500  0.250  0.250  0.250  0.250  0.250  0.500
## treatment21:pair11  0.250  0.250  0.500  0.250  0.250  0.250  0.250  0.250
## treatment22:pair11  0.250  0.250  0.250  0.500  0.250  0.250  0.250  0.250
## treatment23:pair11  0.250  0.250  0.250  0.250  0.500  0.250  0.250  0.250
## treatment24:pair11  0.250  0.250  0.250  0.250  0.250  0.500  0.250  0.250
## treatment25:pair11  0.500  0.250  0.250  0.250  0.250  0.250  0.500  0.250
## treatment26:pair11  0.250  0.500  0.250  0.250  0.250  0.250  0.250  0.500
## treatment21:pair12  0.250  0.250  0.500  0.250  0.250  0.250  0.250  0.250
## treatment22:pair12  0.250  0.250  0.250  0.500  0.250  0.250  0.250  0.250
## treatment23:pair12  0.250  0.250  0.250  0.250  0.500  0.250  0.250  0.250
## treatment24:pair12  0.250  0.250  0.250  0.250  0.250  0.500  0.250  0.250
## treatment25:pair12  0.500  0.250  0.250  0.250  0.250  0.250  0.500  0.250
## treatment26:pair12  0.250  0.500  0.250  0.250  0.250  0.250  0.250  0.500
## treatment21:pair13  0.250  0.250  0.500  0.250  0.250  0.250  0.250  0.250
## treatment22:pair13  0.250  0.250  0.250  0.500  0.250  0.250  0.250  0.250
## treatment23:pair13  0.250  0.250  0.250  0.250  0.500  0.250  0.250  0.250
## treatment24:pair13  0.250  0.250  0.250  0.250  0.250  0.500  0.250  0.250
## treatment25:pair13  0.500  0.250  0.250  0.250  0.250  0.250  0.500  0.250
## treatment26:pair13  0.250  0.500  0.250  0.250  0.250  0.250  0.250  0.500
## treatment21:pair14  0.250  0.250  0.500  0.250  0.250  0.250  0.250  0.250
## treatment22:pair14  0.250  0.250  0.250  0.500  0.250  0.250  0.250  0.250
## treatment23:pair14  0.250  0.250  0.250  0.250  0.500  0.250  0.250  0.250
```

```
## treatment24:pair14  0.250  0.250  0.250  0.250  0.250  0.500  0.250  0.250
## treatment25:pair14  0.500  0.250  0.250  0.250  0.250  0.250  0.500  0.250
## treatment26:pair14  0.250  0.500  0.250  0.250  0.250  0.250  0.250  0.500
## treatment21:pair15  0.250  0.250  0.500  0.250  0.250  0.250  0.250  0.250
## treatment22:pair15  0.250  0.250  0.250  0.500  0.250  0.250  0.250  0.250
## treatment23:pair15  0.250  0.250  0.250  0.250  0.500  0.250  0.250  0.250
## treatment24:pair15  0.250  0.250  0.250  0.250  0.250  0.500  0.250  0.250
## treatment25:pair15  0.500  0.250  0.250  0.250  0.250  0.250  0.500  0.250
## treatment26:pair15  0.250  0.500  0.250  0.250  0.250  0.250  0.250  0.500
##                    tr21:4 tr22:4 tr23:4 tr24:4 tr25:4 tr26:4 tr21:5 tr22:5
## treatment21
## treatment22
## treatment23
## treatment24
## treatment25
## treatment26
## pair2
## pair3
## pair4
## pair5
## pair6
## pair7
## pair9
## pair10
## pair11
## pair12
## pair13
## pair14
## pair15
## treatment21:pair2
## treatment22:pair2
## treatment23:pair2
## treatment24:pair2
## treatment25:pair2
## treatment26:pair2
## treatment21:pair3
## treatment22:pair3
## treatment23:pair3
## treatment24:pair3
## treatment25:pair3
## treatment26:pair3
## treatment21:pair4
## treatment22:pair4   0.500
## treatment23:pair4   0.500  0.500
## treatment24:pair4   0.500  0.500  0.500
## treatment25:pair4   0.500  0.500  0.500  0.500
## treatment26:pair4   0.500  0.500  0.500  0.500  0.500
## treatment21:pair5   0.500  0.250  0.250  0.250  0.250  0.250
## treatment22:pair5   0.250  0.500  0.250  0.250  0.250  0.250  0.500
## treatment23:pair5   0.250  0.250  0.500  0.250  0.250  0.250  0.500  0.500
## treatment24:pair5   0.250  0.250  0.250  0.500  0.250  0.250  0.500  0.500
## treatment25:pair5   0.250  0.250  0.250  0.250  0.500  0.250  0.500  0.500
## treatment26:pair5   0.250  0.250  0.250  0.250  0.250  0.500  0.500  0.500
## treatment21:pair6   0.500  0.250  0.250  0.250  0.250  0.250  0.500  0.250
```

```
## treatment22:pair6   0.250  0.500  0.250  0.250  0.250  0.250  0.250  0.500
## treatment23:pair6   0.250  0.250  0.500  0.250  0.250  0.250  0.250  0.250
## treatment24:pair6   0.250  0.250  0.250  0.500  0.250  0.250  0.250  0.250
## treatment25:pair6   0.250  0.250  0.250  0.250  0.500  0.250  0.250  0.250
## treatment26:pair6   0.250  0.250  0.250  0.250  0.250  0.500  0.250  0.250
## treatment21:pair7   0.500  0.250  0.250  0.250  0.250  0.250  0.500  0.250
## treatment22:pair7   0.250  0.500  0.250  0.250  0.250  0.250  0.250  0.500
## treatment23:pair7   0.250  0.250  0.500  0.250  0.250  0.250  0.250  0.250
## treatment24:pair7   0.250  0.250  0.250  0.500  0.250  0.250  0.250  0.250
## treatment25:pair7   0.250  0.250  0.250  0.250  0.500  0.250  0.250  0.250
## treatment26:pair7   0.250  0.250  0.250  0.250  0.250  0.500  0.250  0.250
## treatment21:pair9   0.500  0.250  0.250  0.250  0.250  0.250  0.500  0.250
## treatment22:pair9   0.250  0.500  0.250  0.250  0.250  0.250  0.250  0.500
## treatment23:pair9   0.250  0.250  0.500  0.250  0.250  0.250  0.250  0.250
## treatment24:pair9   0.250  0.250  0.250  0.500  0.250  0.250  0.250  0.250
## treatment25:pair9   0.250  0.250  0.250  0.250  0.500  0.250  0.250  0.250
## treatment26:pair9   0.250  0.250  0.250  0.250  0.250  0.500  0.250  0.250
## treatment21:pair10  0.500  0.250  0.250  0.250  0.250  0.250  0.500  0.250
## treatment22:pair10  0.250  0.500  0.250  0.250  0.250  0.250  0.250  0.500
## treatment23:pair10  0.250  0.250  0.500  0.250  0.250  0.250  0.250  0.250
## treatment24:pair10  0.250  0.250  0.250  0.500  0.250  0.250  0.250  0.250
## treatment25:pair10  0.250  0.250  0.250  0.250  0.500  0.250  0.250  0.250
## treatment26:pair10  0.250  0.250  0.250  0.250  0.250  0.500  0.250  0.250
## treatment21:pair11  0.500  0.250  0.250  0.250  0.250  0.250  0.500  0.250
## treatment22:pair11  0.250  0.500  0.250  0.250  0.250  0.250  0.250  0.500
## treatment23:pair11  0.250  0.250  0.500  0.250  0.250  0.250  0.250  0.250
## treatment24:pair11  0.250  0.250  0.250  0.500  0.250  0.250  0.250  0.250
## treatment25:pair11  0.250  0.250  0.250  0.250  0.500  0.250  0.250  0.250
## treatment26:pair11  0.250  0.250  0.250  0.250  0.250  0.500  0.250  0.250
## treatment21:pair12  0.500  0.250  0.250  0.250  0.250  0.250  0.500  0.250
## treatment22:pair12  0.250  0.500  0.250  0.250  0.250  0.250  0.250  0.500
## treatment23:pair12  0.250  0.250  0.500  0.250  0.250  0.250  0.250  0.250
## treatment24:pair12  0.250  0.250  0.250  0.500  0.250  0.250  0.250  0.250
## treatment25:pair12  0.250  0.250  0.250  0.250  0.500  0.250  0.250  0.250
## treatment26:pair12  0.250  0.250  0.250  0.250  0.250  0.500  0.250  0.250
## treatment21:pair13  0.500  0.250  0.250  0.250  0.250  0.250  0.500  0.250
## treatment22:pair13  0.250  0.500  0.250  0.250  0.250  0.250  0.250  0.500
## treatment23:pair13  0.250  0.250  0.500  0.250  0.250  0.250  0.250  0.250
## treatment24:pair13  0.250  0.250  0.250  0.500  0.250  0.250  0.250  0.250
## treatment25:pair13  0.250  0.250  0.250  0.250  0.500  0.250  0.250  0.250
## treatment26:pair13  0.250  0.250  0.250  0.250  0.250  0.500  0.250  0.250
## treatment21:pair14  0.500  0.250  0.250  0.250  0.250  0.250  0.500  0.250
## treatment22:pair14  0.250  0.500  0.250  0.250  0.250  0.250  0.250  0.500
## treatment23:pair14  0.250  0.250  0.500  0.250  0.250  0.250  0.250  0.250
## treatment24:pair14  0.250  0.250  0.250  0.500  0.250  0.250  0.250  0.250
## treatment25:pair14  0.250  0.250  0.250  0.250  0.500  0.250  0.250  0.250
## treatment26:pair14  0.250  0.250  0.250  0.250  0.250  0.500  0.250  0.250
## treatment21:pair15  0.500  0.250  0.250  0.250  0.250  0.250  0.500  0.250
## treatment22:pair15  0.250  0.500  0.250  0.250  0.250  0.250  0.250  0.500
## treatment23:pair15  0.250  0.250  0.500  0.250  0.250  0.250  0.250  0.250
## treatment24:pair15  0.250  0.250  0.250  0.500  0.250  0.250  0.250  0.250
## treatment25:pair15  0.250  0.250  0.250  0.250  0.500  0.250  0.250  0.250
## treatment26:pair15  0.250  0.250  0.250  0.250  0.250  0.500  0.250  0.250
##                    tr23:5 tr24:5 tr25:5 tr26:5 tr21:6 tr22:6 tr23:6 tr24:6
```

```
## treatment21
## treatment22
## treatment23
## treatment24
## treatment25
## treatment26
## pair2
## pair3
## pair4
## pair5
## pair6
## pair7
## pair9
## pair10
## pair11
## pair12
## pair13
## pair14
## pair15
## treatment21:pair2
## treatment22:pair2
## treatment23:pair2
## treatment24:pair2
## treatment25:pair2
## treatment26:pair2
## treatment21:pair3
## treatment22:pair3
## treatment23:pair3
## treatment24:pair3
## treatment25:pair3
## treatment26:pair3
## treatment21:pair4
## treatment22:pair4
## treatment23:pair4
## treatment24:pair4
## treatment25:pair4
## treatment26:pair4
## treatment21:pair5
## treatment22:pair5
## treatment23:pair5
## treatment24:pair5   0.500
## treatment25:pair5   0.500  0.500
## treatment26:pair5   0.500  0.500  0.500
## treatment21:pair6   0.250  0.250  0.250  0.250
## treatment22:pair6   0.250  0.250  0.250  0.250  0.500
## treatment23:pair6   0.500  0.250  0.250  0.250  0.500  0.500
## treatment24:pair6   0.250  0.500  0.250  0.250  0.500  0.500  0.500
## treatment25:pair6   0.250  0.250  0.500  0.250  0.500  0.500  0.500  0.500
## treatment26:pair6   0.250  0.250  0.250  0.500  0.500  0.500  0.500  0.500
## treatment21:pair7   0.250  0.250  0.250  0.250  0.500  0.250  0.250  0.250
## treatment22:pair7   0.250  0.250  0.250  0.250  0.250  0.500  0.250  0.250
## treatment23:pair7   0.500  0.250  0.250  0.250  0.250  0.250  0.500  0.250
## treatment24:pair7   0.250  0.500  0.250  0.250  0.250  0.250  0.250  0.500
## treatment25:pair7   0.250  0.250  0.500  0.250  0.250  0.250  0.250  0.250
```

```
## treatment26:pair7   0.250  0.250  0.250  0.500  0.250  0.250  0.250  0.250
## treatment21:pair9   0.250  0.250  0.250  0.250  0.500  0.250  0.250  0.250
## treatment22:pair9   0.250  0.250  0.250  0.250  0.250  0.500  0.250  0.250
## treatment23:pair9   0.500  0.250  0.250  0.250  0.250  0.250  0.500  0.250
## treatment24:pair9   0.250  0.500  0.250  0.250  0.250  0.250  0.250  0.500
## treatment25:pair9   0.250  0.250  0.500  0.250  0.250  0.250  0.250  0.250
## treatment26:pair9   0.250  0.250  0.250  0.500  0.250  0.250  0.250  0.250
## treatment21:pair10  0.250  0.250  0.250  0.250  0.500  0.250  0.250  0.250
## treatment22:pair10  0.250  0.250  0.250  0.250  0.250  0.500  0.250  0.250
## treatment23:pair10  0.500  0.250  0.250  0.250  0.250  0.250  0.500  0.250
## treatment24:pair10  0.250  0.500  0.250  0.250  0.250  0.250  0.250  0.500
## treatment25:pair10  0.250  0.250  0.500  0.250  0.250  0.250  0.250  0.250
## treatment26:pair10  0.250  0.250  0.250  0.500  0.250  0.250  0.250  0.250
## treatment21:pair11  0.250  0.250  0.250  0.250  0.500  0.250  0.250  0.250
## treatment22:pair11  0.250  0.250  0.250  0.250  0.250  0.500  0.250  0.250
## treatment23:pair11  0.500  0.250  0.250  0.250  0.250  0.250  0.500  0.250
## treatment24:pair11  0.250  0.500  0.250  0.250  0.250  0.250  0.250  0.500
## treatment25:pair11  0.250  0.250  0.500  0.250  0.250  0.250  0.250  0.250
## treatment26:pair11  0.250  0.250  0.250  0.500  0.250  0.250  0.250  0.250
## treatment21:pair12  0.250  0.250  0.250  0.250  0.500  0.250  0.250  0.250
## treatment22:pair12  0.250  0.250  0.250  0.250  0.250  0.500  0.250  0.250
## treatment23:pair12  0.500  0.250  0.250  0.250  0.250  0.250  0.500  0.250
## treatment24:pair12  0.250  0.500  0.250  0.250  0.250  0.250  0.250  0.500
## treatment25:pair12  0.250  0.250  0.500  0.250  0.250  0.250  0.250  0.250
## treatment26:pair12  0.250  0.250  0.250  0.500  0.250  0.250  0.250  0.250
## treatment21:pair13  0.250  0.250  0.250  0.250  0.500  0.250  0.250  0.250
## treatment22:pair13  0.250  0.250  0.250  0.250  0.250  0.500  0.250  0.250
## treatment23:pair13  0.500  0.250  0.250  0.250  0.250  0.250  0.500  0.250
## treatment24:pair13  0.250  0.500  0.250  0.250  0.250  0.250  0.250  0.500
## treatment25:pair13  0.250  0.250  0.500  0.250  0.250  0.250  0.250  0.250
## treatment26:pair13  0.250  0.250  0.250  0.500  0.250  0.250  0.250  0.250
## treatment21:pair14  0.250  0.250  0.250  0.250  0.500  0.250  0.250  0.250
## treatment22:pair14  0.250  0.250  0.250  0.250  0.250  0.500  0.250  0.250
## treatment23:pair14  0.500  0.250  0.250  0.250  0.250  0.250  0.500  0.250
## treatment24:pair14  0.250  0.500  0.250  0.250  0.250  0.250  0.250  0.500
## treatment25:pair14  0.250  0.250  0.500  0.250  0.250  0.250  0.250  0.250
## treatment26:pair14  0.250  0.250  0.250  0.500  0.250  0.250  0.250  0.250
## treatment21:pair15  0.250  0.250  0.250  0.250  0.500  0.250  0.250  0.250
## treatment22:pair15  0.250  0.250  0.250  0.250  0.250  0.500  0.250  0.250
## treatment23:pair15  0.500  0.250  0.250  0.250  0.250  0.250  0.500  0.250
## treatment24:pair15  0.250  0.500  0.250  0.250  0.250  0.250  0.250  0.500
## treatment25:pair15  0.250  0.250  0.500  0.250  0.250  0.250  0.250  0.250
## treatment26:pair15  0.250  0.250  0.250  0.500  0.250  0.250  0.250  0.250
##                    tr25:6 tr26:6 tr21:7 tr22:7 tr23:7 tr24:7 tr25:7 tr26:7
## treatment21
## treatment22
## treatment23
## treatment24
## treatment25
## treatment26
## pair2
## pair3
## pair4
## pair5
```

```
## pair6
## pair7
## pair9
## pair10
## pair11
## pair12
## pair13
## pair14
## pair15
## treatment21:pair2
## treatment22:pair2
## treatment23:pair2
## treatment24:pair2
## treatment25:pair2
## treatment26:pair2
## treatment21:pair3
## treatment22:pair3
## treatment23:pair3
## treatment24:pair3
## treatment25:pair3
## treatment26:pair3
## treatment21:pair4
## treatment22:pair4
## treatment23:pair4
## treatment24:pair4
## treatment25:pair4
## treatment26:pair4
## treatment21:pair5
## treatment22:pair5
## treatment23:pair5
## treatment24:pair5
## treatment25:pair5
## treatment26:pair5
## treatment21:pair6
## treatment22:pair6
## treatment23:pair6
## treatment24:pair6
## treatment25:pair6
## treatment26:pair6   0.500
## treatment21:pair7   0.250  0.250
## treatment22:pair7   0.250  0.250  0.500
## treatment23:pair7   0.250  0.250  0.500  0.500
## treatment24:pair7   0.250  0.250  0.500  0.500  0.500
## treatment25:pair7   0.500  0.250  0.500  0.500  0.500  0.500
## treatment26:pair7   0.250  0.500  0.500  0.500  0.500  0.500  0.500
## treatment21:pair9   0.250  0.250  0.500  0.250  0.250  0.250  0.250  0.250
## treatment22:pair9   0.250  0.250  0.250  0.500  0.250  0.250  0.250  0.250
## treatment23:pair9   0.250  0.250  0.250  0.250  0.500  0.250  0.250  0.250
## treatment24:pair9   0.250  0.250  0.250  0.250  0.250  0.500  0.250  0.250
## treatment25:pair9   0.500  0.250  0.250  0.250  0.250  0.250  0.500  0.250
## treatment26:pair9   0.250  0.500  0.250  0.250  0.250  0.250  0.250  0.500
## treatment21:pair10  0.250  0.250  0.500  0.250  0.250  0.250  0.250  0.250
## treatment22:pair10  0.250  0.250  0.250  0.500  0.250  0.250  0.250  0.250
## treatment23:pair10  0.250  0.250  0.250  0.250  0.500  0.250  0.250  0.250
```

```
## treatment24:pair10  0.250  0.250  0.250  0.250  0.250  0.500  0.250  0.250
## treatment25:pair10  0.500  0.250  0.250  0.250  0.250  0.250  0.500  0.250
## treatment26:pair10  0.250  0.500  0.250  0.250  0.250  0.250  0.250  0.500
## treatment21:pair11  0.250  0.250  0.500  0.250  0.250  0.250  0.250  0.250
## treatment22:pair11  0.250  0.250  0.250  0.500  0.250  0.250  0.250  0.250
## treatment23:pair11  0.250  0.250  0.250  0.250  0.500  0.250  0.250  0.250
## treatment24:pair11  0.250  0.250  0.250  0.250  0.250  0.500  0.250  0.250
## treatment25:pair11  0.500  0.250  0.250  0.250  0.250  0.250  0.500  0.250
## treatment26:pair11  0.250  0.500  0.250  0.250  0.250  0.250  0.250  0.500
## treatment21:pair12  0.250  0.250  0.500  0.250  0.250  0.250  0.250  0.250
## treatment22:pair12  0.250  0.250  0.250  0.500  0.250  0.250  0.250  0.250
## treatment23:pair12  0.250  0.250  0.250  0.250  0.500  0.250  0.250  0.250
## treatment24:pair12  0.250  0.250  0.250  0.250  0.250  0.500  0.250  0.250
## treatment25:pair12  0.500  0.250  0.250  0.250  0.250  0.250  0.500  0.250
## treatment26:pair12  0.250  0.500  0.250  0.250  0.250  0.250  0.250  0.500
## treatment21:pair13  0.250  0.250  0.500  0.250  0.250  0.250  0.250  0.250
## treatment22:pair13  0.250  0.250  0.250  0.500  0.250  0.250  0.250  0.250
## treatment23:pair13  0.250  0.250  0.250  0.250  0.500  0.250  0.250  0.250
## treatment24:pair13  0.250  0.250  0.250  0.250  0.250  0.500  0.250  0.250
## treatment25:pair13  0.500  0.250  0.250  0.250  0.250  0.250  0.500  0.250
## treatment26:pair13  0.250  0.500  0.250  0.250  0.250  0.250  0.250  0.500
## treatment21:pair14  0.250  0.250  0.500  0.250  0.250  0.250  0.250  0.250
## treatment22:pair14  0.250  0.250  0.250  0.500  0.250  0.250  0.250  0.250
## treatment23:pair14  0.250  0.250  0.250  0.250  0.500  0.250  0.250  0.250
## treatment24:pair14  0.250  0.250  0.250  0.250  0.250  0.500  0.250  0.250
## treatment25:pair14  0.500  0.250  0.250  0.250  0.250  0.250  0.500  0.250
## treatment26:pair14  0.250  0.500  0.250  0.250  0.250  0.250  0.250  0.500
## treatment21:pair15  0.250  0.250  0.500  0.250  0.250  0.250  0.250  0.250
## treatment22:pair15  0.250  0.250  0.250  0.500  0.250  0.250  0.250  0.250
## treatment23:pair15  0.250  0.250  0.250  0.250  0.500  0.250  0.250  0.250
## treatment24:pair15  0.250  0.250  0.250  0.250  0.250  0.500  0.250  0.250
## treatment25:pair15  0.500  0.250  0.250  0.250  0.250  0.250  0.500  0.250
## treatment26:pair15  0.250  0.500  0.250  0.250  0.250  0.250  0.250  0.500
##                    tr21:9 tr22:9 tr23:9 tr24:9 tr25:9 tr26:9 t21:10 t22:10
## treatment21
## treatment22
## treatment23
## treatment24
## treatment25
## treatment26
## pair2
## pair3
## pair4
## pair5
## pair6
## pair7
## pair9
## pair10
## pair11
## pair12
## pair13
## pair14
## pair15
## treatment21:pair2
```

```
## treatment22:pair2
## treatment23:pair2
## treatment24:pair2
## treatment25:pair2
## treatment26:pair2
## treatment21:pair3
## treatment22:pair3
## treatment23:pair3
## treatment24:pair3
## treatment25:pair3
## treatment26:pair3
## treatment21:pair4
## treatment22:pair4
## treatment23:pair4
## treatment24:pair4
## treatment25:pair4
## treatment26:pair4
## treatment21:pair5
## treatment22:pair5
## treatment23:pair5
## treatment24:pair5
## treatment25:pair5
## treatment26:pair5
## treatment21:pair6
## treatment22:pair6
## treatment23:pair6
## treatment24:pair6
## treatment25:pair6
## treatment26:pair6
## treatment21:pair7
## treatment22:pair7
## treatment23:pair7
## treatment24:pair7
## treatment25:pair7
## treatment26:pair7
## treatment21:pair9
## treatment22:pair9   0.500
## treatment23:pair9   0.500  0.500
## treatment24:pair9   0.500  0.500  0.500
## treatment25:pair9   0.500  0.500  0.500  0.500
## treatment26:pair9   0.500  0.500  0.500  0.500  0.500
## treatment21:pair10  0.500  0.250  0.250  0.250  0.250  0.250
## treatment22:pair10  0.250  0.500  0.250  0.250  0.250  0.250  0.500
## treatment23:pair10  0.250  0.250  0.500  0.250  0.250  0.250  0.500  0.500
## treatment24:pair10  0.250  0.250  0.250  0.500  0.250  0.250  0.500  0.500
## treatment25:pair10  0.250  0.250  0.250  0.250  0.500  0.250  0.500  0.500
## treatment26:pair10  0.250  0.250  0.250  0.250  0.250  0.500  0.500  0.500
## treatment21:pair11  0.500  0.250  0.250  0.250  0.250  0.250  0.500  0.250
## treatment22:pair11  0.250  0.500  0.250  0.250  0.250  0.250  0.250  0.500
## treatment23:pair11  0.250  0.250  0.500  0.250  0.250  0.250  0.250  0.250
## treatment24:pair11  0.250  0.250  0.250  0.500  0.250  0.250  0.250  0.250
## treatment25:pair11  0.250  0.250  0.250  0.250  0.500  0.250  0.250  0.250
## treatment26:pair11  0.250  0.250  0.250  0.250  0.250  0.500  0.250  0.250
## treatment21:pair12  0.500  0.250  0.250  0.250  0.250  0.250  0.500  0.250
```

```
## treatment22:pair12  0.250  0.500  0.250  0.250  0.250  0.250  0.250  0.500
## treatment23:pair12  0.250  0.250  0.500  0.250  0.250  0.250  0.250  0.250
## treatment24:pair12  0.250  0.250  0.250  0.500  0.250  0.250  0.250  0.250
## treatment25:pair12  0.250  0.250  0.250  0.250  0.500  0.250  0.250  0.250
## treatment26:pair12  0.250  0.250  0.250  0.250  0.250  0.500  0.250  0.250
## treatment21:pair13  0.500  0.250  0.250  0.250  0.250  0.250  0.500  0.250
## treatment22:pair13  0.250  0.500  0.250  0.250  0.250  0.250  0.250  0.500
## treatment23:pair13  0.250  0.250  0.500  0.250  0.250  0.250  0.250  0.250
## treatment24:pair13  0.250  0.250  0.250  0.500  0.250  0.250  0.250  0.250
## treatment25:pair13  0.250  0.250  0.250  0.250  0.500  0.250  0.250  0.250
## treatment26:pair13  0.250  0.250  0.250  0.250  0.250  0.500  0.250  0.250
## treatment21:pair14  0.500  0.250  0.250  0.250  0.250  0.250  0.500  0.250
## treatment22:pair14  0.250  0.500  0.250  0.250  0.250  0.250  0.250  0.500
## treatment23:pair14  0.250  0.250  0.500  0.250  0.250  0.250  0.250  0.250
## treatment24:pair14  0.250  0.250  0.250  0.500  0.250  0.250  0.250  0.250
## treatment25:pair14  0.250  0.250  0.250  0.250  0.500  0.250  0.250  0.250
## treatment26:pair14  0.250  0.250  0.250  0.250  0.250  0.500  0.250  0.250
## treatment21:pair15  0.500  0.250  0.250  0.250  0.250  0.250  0.500  0.250
## treatment22:pair15  0.250  0.500  0.250  0.250  0.250  0.250  0.250  0.500
## treatment23:pair15  0.250  0.250  0.500  0.250  0.250  0.250  0.250  0.250
## treatment24:pair15  0.250  0.250  0.250  0.500  0.250  0.250  0.250  0.250
## treatment25:pair15  0.250  0.250  0.250  0.250  0.500  0.250  0.250  0.250
## treatment26:pair15  0.250  0.250  0.250  0.250  0.250  0.500  0.250  0.250
##                    t23:10 t24:10 t25:10 t26:10 t21:11 t22:11 t23:11 t24:11
## treatment21
## treatment22
## treatment23
## treatment24
## treatment25
## treatment26
## pair2
## pair3
## pair4
## pair5
## pair6
## pair7
## pair9
## pair10
## pair11
## pair12
## pair13
## pair14
## pair15
## treatment21:pair2
## treatment22:pair2
## treatment23:pair2
## treatment24:pair2
## treatment25:pair2
## treatment26:pair2
## treatment21:pair3
## treatment22:pair3
## treatment23:pair3
## treatment24:pair3
## treatment25:pair3
```

```
## treatment26:pair3
## treatment21:pair4
## treatment22:pair4
## treatment23:pair4
## treatment24:pair4
## treatment25:pair4
## treatment26:pair4
## treatment21:pair5
## treatment22:pair5
## treatment23:pair5
## treatment24:pair5
## treatment25:pair5
## treatment26:pair5
## treatment21:pair6
## treatment22:pair6
## treatment23:pair6
## treatment24:pair6
## treatment25:pair6
## treatment26:pair6
## treatment21:pair7
## treatment22:pair7
## treatment23:pair7
## treatment24:pair7
## treatment25:pair7
## treatment26:pair7
## treatment21:pair9
## treatment22:pair9
## treatment23:pair9
## treatment24:pair9
## treatment25:pair9
## treatment26:pair9
## treatment21:pair10
## treatment22:pair10
## treatment23:pair10
## treatment24:pair10  0.500
## treatment25:pair10  0.500  0.500
## treatment26:pair10  0.500  0.500  0.500
## treatment21:pair11  0.250  0.250  0.250  0.250
## treatment22:pair11  0.250  0.250  0.250  0.250  0.500
## treatment23:pair11  0.500  0.250  0.250  0.250  0.500  0.500
## treatment24:pair11  0.250  0.500  0.250  0.250  0.500  0.500  0.500
## treatment25:pair11  0.250  0.250  0.500  0.250  0.500  0.500  0.500  0.500
## treatment26:pair11  0.250  0.250  0.250  0.500  0.500  0.500  0.500  0.500
## treatment21:pair12  0.250  0.250  0.250  0.250  0.500  0.250  0.250  0.250
## treatment22:pair12  0.250  0.250  0.250  0.250  0.250  0.500  0.250  0.250
## treatment23:pair12  0.500  0.250  0.250  0.250  0.250  0.250  0.500  0.250
## treatment24:pair12  0.250  0.500  0.250  0.250  0.250  0.250  0.250  0.500
## treatment25:pair12  0.250  0.250  0.500  0.250  0.250  0.250  0.250  0.250
## treatment26:pair12  0.250  0.250  0.250  0.500  0.250  0.250  0.250  0.250
## treatment21:pair13  0.250  0.250  0.250  0.250  0.500  0.250  0.250  0.250
## treatment22:pair13  0.250  0.250  0.250  0.250  0.250  0.500  0.250  0.250
## treatment23:pair13  0.500  0.250  0.250  0.250  0.250  0.250  0.500  0.250
## treatment24:pair13  0.250  0.500  0.250  0.250  0.250  0.250  0.250  0.500
## treatment25:pair13  0.250  0.250  0.500  0.250  0.250  0.250  0.250  0.250
```

```
## treatment26:pair13  0.250  0.250  0.250  0.500  0.250  0.250  0.250  0.250
## treatment21:pair14  0.250  0.250  0.250  0.250  0.500  0.250  0.250  0.250
## treatment22:pair14  0.250  0.250  0.250  0.250  0.250  0.500  0.250  0.250
## treatment23:pair14  0.500  0.250  0.250  0.250  0.250  0.250  0.500  0.250
## treatment24:pair14  0.250  0.500  0.250  0.250  0.250  0.250  0.250  0.500
## treatment25:pair14  0.250  0.250  0.500  0.250  0.250  0.250  0.250  0.250
## treatment26:pair14  0.250  0.250  0.250  0.500  0.250  0.250  0.250  0.250
## treatment21:pair15  0.250  0.250  0.250  0.250  0.500  0.250  0.250  0.250
## treatment22:pair15  0.250  0.250  0.250  0.250  0.250  0.500  0.250  0.250
## treatment23:pair15  0.500  0.250  0.250  0.250  0.250  0.250  0.500  0.250
## treatment24:pair15  0.250  0.500  0.250  0.250  0.250  0.250  0.250  0.500
## treatment25:pair15  0.250  0.250  0.500  0.250  0.250  0.250  0.250  0.250
## treatment26:pair15  0.250  0.250  0.250  0.500  0.250  0.250  0.250  0.250
##                    t25:11 t26:11 t21:12 t22:12 t23:12 t24:12 t25:12 t26:12
## treatment21
## treatment22
## treatment23
## treatment24
## treatment25
## treatment26
## pair2
## pair3
## pair4
## pair5
## pair6
## pair7
## pair9
## pair10
## pair11
## pair12
## pair13
## pair14
## pair15
## treatment21:pair2
## treatment22:pair2
## treatment23:pair2
## treatment24:pair2
## treatment25:pair2
## treatment26:pair2
## treatment21:pair3
## treatment22:pair3
## treatment23:pair3
## treatment24:pair3
## treatment25:pair3
## treatment26:pair3
## treatment21:pair4
## treatment22:pair4
## treatment23:pair4
## treatment24:pair4
## treatment25:pair4
## treatment26:pair4
## treatment21:pair5
## treatment22:pair5
## treatment23:pair5
```

```
## treatment24:pair5
## treatment25:pair5
## treatment26:pair5
## treatment21:pair6
## treatment22:pair6
## treatment23:pair6
## treatment24:pair6
## treatment25:pair6
## treatment26:pair6
## treatment21:pair7
## treatment22:pair7
## treatment23:pair7
## treatment24:pair7
## treatment25:pair7
## treatment26:pair7
## treatment21:pair9
## treatment22:pair9
## treatment23:pair9
## treatment24:pair9
## treatment25:pair9
## treatment26:pair9
## treatment21:pair10
## treatment22:pair10
## treatment23:pair10
## treatment24:pair10
## treatment25:pair10
## treatment26:pair10
## treatment21:pair11
## treatment22:pair11
## treatment23:pair11
## treatment24:pair11
## treatment25:pair11
## treatment26:pair11  0.500
## treatment21:pair12  0.250  0.250
## treatment22:pair12  0.250  0.250  0.500
## treatment23:pair12  0.250  0.250  0.500  0.500
## treatment24:pair12  0.250  0.250  0.500  0.500  0.500
## treatment25:pair12  0.500  0.250  0.500  0.500  0.500  0.500
## treatment26:pair12  0.250  0.500  0.500  0.500  0.500  0.500  0.500
## treatment21:pair13  0.250  0.250  0.500  0.250  0.250  0.250  0.250  0.250
## treatment22:pair13  0.250  0.250  0.250  0.500  0.250  0.250  0.250  0.250
## treatment23:pair13  0.250  0.250  0.250  0.250  0.500  0.250  0.250  0.250
## treatment24:pair13  0.250  0.250  0.250  0.250  0.250  0.500  0.250  0.250
## treatment25:pair13  0.500  0.250  0.250  0.250  0.250  0.250  0.500  0.250
## treatment26:pair13  0.250  0.500  0.250  0.250  0.250  0.250  0.250  0.500
## treatment21:pair14  0.250  0.250  0.500  0.250  0.250  0.250  0.250  0.250
## treatment22:pair14  0.250  0.250  0.250  0.500  0.250  0.250  0.250  0.250
## treatment23:pair14  0.250  0.250  0.250  0.250  0.500  0.250  0.250  0.250
## treatment24:pair14  0.250  0.250  0.250  0.250  0.250  0.500  0.250  0.250
## treatment25:pair14  0.500  0.250  0.250  0.250  0.250  0.250  0.500  0.250
## treatment26:pair14  0.250  0.500  0.250  0.250  0.250  0.250  0.250  0.500
## treatment21:pair15  0.250  0.250  0.500  0.250  0.250  0.250  0.250  0.250
## treatment22:pair15  0.250  0.250  0.250  0.500  0.250  0.250  0.250  0.250
## treatment23:pair15  0.250  0.250  0.250  0.250  0.500  0.250  0.250  0.250
```

```
## treatment24:pair15  0.250  0.250  0.250  0.250  0.250  0.500  0.250  0.250
## treatment25:pair15  0.500  0.250  0.250  0.250  0.250  0.250  0.500  0.250
## treatment26:pair15  0.250  0.500  0.250  0.250  0.250  0.250  0.250  0.500
##                    t21:13 t22:13 t23:13 t24:13 t25:13 t26:13 t21:14 t22:14
## treatment21
## treatment22
## treatment23
## treatment24
## treatment25
## treatment26
## pair2
## pair3
## pair4
## pair5
## pair6
## pair7
## pair9
## pair10
## pair11
## pair12
## pair13
## pair14
## pair15
## treatment21:pair2
## treatment22:pair2
## treatment23:pair2
## treatment24:pair2
## treatment25:pair2
## treatment26:pair2
## treatment21:pair3
## treatment22:pair3
## treatment23:pair3
## treatment24:pair3
## treatment25:pair3
## treatment26:pair3
## treatment21:pair4
## treatment22:pair4
## treatment23:pair4
## treatment24:pair4
## treatment25:pair4
## treatment26:pair4
## treatment21:pair5
## treatment22:pair5
## treatment23:pair5
## treatment24:pair5
## treatment25:pair5
## treatment26:pair5
## treatment21:pair6
## treatment22:pair6
## treatment23:pair6
## treatment24:pair6
## treatment25:pair6
## treatment26:pair6
## treatment21:pair7
```

```
## treatment22:pair7
## treatment23:pair7
## treatment24:pair7
## treatment25:pair7
## treatment26:pair7
## treatment21:pair9
## treatment22:pair9
## treatment23:pair9
## treatment24:pair9
## treatment25:pair9
## treatment26:pair9
## treatment21:pair10
## treatment22:pair10
## treatment23:pair10
## treatment24:pair10
## treatment25:pair10
## treatment26:pair10
## treatment21:pair11
## treatment22:pair11
## treatment23:pair11
## treatment24:pair11
## treatment25:pair11
## treatment26:pair11
## treatment21:pair12
## treatment22:pair12
## treatment23:pair12
## treatment24:pair12
## treatment25:pair12
## treatment26:pair12
## treatment21:pair13
## treatment22:pair13  0.500
## treatment23:pair13  0.500  0.500
## treatment24:pair13  0.500  0.500  0.500
## treatment25:pair13  0.500  0.500  0.500  0.500
## treatment26:pair13  0.500  0.500  0.500  0.500  0.500
## treatment21:pair14  0.500  0.250  0.250  0.250  0.250  0.250
## treatment22:pair14  0.250  0.500  0.250  0.250  0.250  0.250  0.500
## treatment23:pair14  0.250  0.250  0.500  0.250  0.250  0.250  0.500  0.500
## treatment24:pair14  0.250  0.250  0.250  0.500  0.250  0.250  0.500  0.500
## treatment25:pair14  0.250  0.250  0.250  0.250  0.500  0.250  0.500  0.500
## treatment26:pair14  0.250  0.250  0.250  0.250  0.250  0.500  0.500  0.500
## treatment21:pair15  0.500  0.250  0.250  0.250  0.250  0.250  0.500  0.250
## treatment22:pair15  0.250  0.500  0.250  0.250  0.250  0.250  0.250  0.500
## treatment23:pair15  0.250  0.250  0.500  0.250  0.250  0.250  0.250  0.250
## treatment24:pair15  0.250  0.250  0.250  0.500  0.250  0.250  0.250  0.250
## treatment25:pair15  0.250  0.250  0.250  0.250  0.500  0.250  0.250  0.250
## treatment26:pair15  0.250  0.250  0.250  0.250  0.250  0.500  0.250  0.250
##                    t23:14 t24:14 t25:14 t26:14 t21:15 t22:15 t23:15 t24:15
## treatment21
## treatment22
## treatment23
## treatment24
## treatment25
## treatment26
```

```
## pair2
## pair3
## pair4
## pair5
## pair6
## pair7
## pair9
## pair10
## pair11
## pair12
## pair13
## pair14
## pair15
## treatment21:pair2
## treatment22:pair2
## treatment23:pair2
## treatment24:pair2
## treatment25:pair2
## treatment26:pair2
## treatment21:pair3
## treatment22:pair3
## treatment23:pair3
## treatment24:pair3
## treatment25:pair3
## treatment26:pair3
## treatment21:pair4
## treatment22:pair4
## treatment23:pair4
## treatment24:pair4
## treatment25:pair4
## treatment26:pair4
## treatment21:pair5
## treatment22:pair5
## treatment23:pair5
## treatment24:pair5
## treatment25:pair5
## treatment26:pair5
## treatment21:pair6
## treatment22:pair6
## treatment23:pair6
## treatment24:pair6
## treatment25:pair6
## treatment26:pair6
## treatment21:pair7
## treatment22:pair7
## treatment23:pair7
## treatment24:pair7
## treatment25:pair7
## treatment26:pair7
## treatment21:pair9
## treatment22:pair9
## treatment23:pair9
## treatment24:pair9
## treatment25:pair9
```

```
## treatment26:pair9
## treatment21:pair10
## treatment22:pair10
## treatment23:pair10
## treatment24:pair10
## treatment25:pair10
## treatment26:pair10
## treatment21:pair11
## treatment22:pair11
## treatment23:pair11
## treatment24:pair11
## treatment25:pair11
## treatment26:pair11
## treatment21:pair12
## treatment22:pair12
## treatment23:pair12
## treatment24:pair12
## treatment25:pair12
## treatment26:pair12
## treatment21:pair13
## treatment22:pair13
## treatment23:pair13
## treatment24:pair13
## treatment25:pair13
## treatment26:pair13
## treatment21:pair14
## treatment22:pair14
## treatment23:pair14
## treatment24:pair14  0.500
## treatment25:pair14  0.500  0.500
## treatment26:pair14  0.500  0.500  0.500
## treatment21:pair15  0.250  0.250  0.250  0.250
## treatment22:pair15  0.250  0.250  0.250  0.250  0.500
## treatment23:pair15  0.500  0.250  0.250  0.250  0.500  0.500
## treatment24:pair15  0.250  0.500  0.250  0.250  0.500  0.500  0.500
## treatment25:pair15  0.250  0.250  0.500  0.250  0.500  0.500  0.500  0.500
## treatment26:pair15  0.250  0.250  0.250  0.500  0.500  0.500  0.500  0.500
##                    t25:15
## treatment21
## treatment22
## treatment23
## treatment24
## treatment25
## treatment26
## pair2
## pair3
## pair4
## pair5
## pair6
## pair7
## pair9
## pair10
## pair11
## pair12
```

```
## pair13
## pair14
## pair15
## treatment21:pair2
## treatment22:pair2
## treatment23:pair2
## treatment24:pair2
## treatment25:pair2
## treatment26:pair2
## treatment21:pair3
## treatment22:pair3
## treatment23:pair3
## treatment24:pair3
## treatment25:pair3
## treatment26:pair3
## treatment21:pair4
## treatment22:pair4
## treatment23:pair4
## treatment24:pair4
## treatment25:pair4
## treatment26:pair4
## treatment21:pair5
## treatment22:pair5
## treatment23:pair5
## treatment24:pair5
## treatment25:pair5
## treatment26:pair5
## treatment21:pair6
## treatment22:pair6
## treatment23:pair6
## treatment24:pair6
## treatment25:pair6
## treatment26:pair6
## treatment21:pair7
## treatment22:pair7
## treatment23:pair7
## treatment24:pair7
## treatment25:pair7
## treatment26:pair7
## treatment21:pair9
## treatment22:pair9
## treatment23:pair9
## treatment24:pair9
## treatment25:pair9
## treatment26:pair9
## treatment21:pair10
## treatment22:pair10
## treatment23:pair10
## treatment24:pair10
## treatment25:pair10
## treatment26:pair10
## treatment21:pair11
## treatment22:pair11
## treatment23:pair11
```

```
## treatment24:pair11
## treatment25:pair11
## treatment26:pair11
## treatment21:pair12
## treatment22:pair12
## treatment23:pair12
## treatment24:pair12
## treatment25:pair12
## treatment26:pair12
## treatment21:pair13
## treatment22:pair13
## treatment23:pair13
## treatment24:pair13
## treatment25:pair13
## treatment26:pair13
## treatment21:pair14
## treatment22:pair14
## treatment23:pair14
## treatment24:pair14
## treatment25:pair14
## treatment26:pair14
## treatment21:pair15
## treatment22:pair15
## treatment23:pair15
## treatment24:pair15
## treatment25:pair15
## treatment26:pair15  0.500
##
## Standardized Within-Group Residuals:
##        Min         Q1        Med         Q3        Max
## -2.6194082 -0.4981153  0.0212862  0.5095249  4.3292993
##
## Number of Observations: 294
## Number of Groups: 21
```

2344s

```
data<-read.table("~/Desktop/rsync/deteriorating treatments/salt/2344_salt_v2_[0].csv",h=T)
data<-subset(data,treatment<7)
data$pair<-as.factor(data$pair)
data$treatment2<-as.factor(data$treatment)
fit2<-lme(relfitav~treatment2*pair,random=~1|plate,data)
anova(fit2)
```

```
##                  numDF denDF   F-value p-value
## (Intercept)          1   196 1652.9881  <.0001
## treatment2           6    14    6.7136  0.0016
## pair                14   196    4.6379  <.0001
## treatment2:pair     84   196    1.5989  0.0042
```

```r
summary(fit2)
```

```
## Linear mixed-effects model fit by REML
##  Data: data
##        AIC      BIC    logLik
##   444.1124 802.2529 -115.0562
##
## Random effects:
##  Formula: ~1 | plate
##         (Intercept)  Residual
## StdDev:  0.04787761 0.3148591
##
## Fixed effects: relfitav ~ treatment2 * pair
##                          Value Std.Error  DF   t-value p-value
## (Intercept)          0.9002072 0.1838736 196  4.895794  0.0000
## treatment21         -0.1884449 0.2600365  14 -0.724686  0.4806
## treatment22         -0.0919516 0.2600365  14 -0.353610  0.7289
## treatment23         -0.3100512 0.2600365  14 -1.192337  0.2529
## treatment24         -0.4601903 0.2600365  14 -1.769714  0.0985
## treatment25         -0.5088674 0.2600365  14 -1.956907  0.0706
## treatment26         -0.5933544 0.2600365  14 -2.281812  0.0387
## pair2               -0.0266652 0.2570813 196 -0.103723  0.9175
## pair3               -0.1178834 0.2570813 196 -0.458545  0.6471
## pair4                0.1121517 0.2570813 196  0.436250  0.6631
## pair5                0.0122615 0.2570813 196  0.047695  0.9620
## pair6               -0.0565837 0.2570813 196 -0.220100  0.8260
## pair7                0.0757844 0.2570813 196  0.294787  0.7685
## pair8                0.2218575 0.2570813 196  0.862986  0.3892
## pair9               -0.0812490 0.2570813 196 -0.316044  0.7523
## pair10               0.1809333 0.2570813 196  0.703798  0.4824
## pair11              -0.0327714 0.2570813 196 -0.127475  0.8987
## pair12              -0.1727358 0.2570813 196 -0.671911  0.5024
## pair13               0.0496715 0.2570813 196  0.193213  0.8470
## pair14               0.2983923 0.2570813 196  1.160692  0.2472
## pair15              -0.0971434 0.2570813 196 -0.377870  0.7059
## treatment21:pair2    0.2563710 0.3635679 196  0.705153  0.4816
## treatment22:pair2    0.4729478 0.3635679 196  1.300851  0.1948
## treatment23:pair2    0.2927759 0.3635679 196  0.805285  0.4216
## treatment24:pair2    0.6729697 0.3635679 196  1.851015  0.0657
## treatment25:pair2    0.3555712 0.3635679 196  0.978005  0.3293
## treatment26:pair2    0.2819445 0.3635679 196  0.775493  0.4390
## treatment21:pair3    0.4935451 0.3635679 196  1.357505  0.1762
## treatment22:pair3    0.2649372 0.3635679 196  0.728714  0.4670
## treatment23:pair3    0.3109559 0.3635679 196  0.855290  0.3934
## treatment24:pair3    0.4533143 0.3635679 196  1.246849  0.2139
## treatment25:pair3    0.6554435 0.3635679 196  1.802809  0.0730
## treatment26:pair3    0.3169240 0.3635679 196  0.871705  0.3844
## treatment21:pair4    0.1678745 0.3635679 196  0.461742  0.6448
## treatment22:pair4   -0.1963264 0.3635679 196 -0.539999  0.5898
## treatment23:pair4   -0.1217449 0.3635679 196 -0.334862  0.7381
## treatment24:pair4    0.3085655 0.3635679 196  0.848715  0.3971
## treatment25:pair4    0.4512413 0.3635679 196  1.241147  0.2160
## treatment26:pair4   -0.1121517 0.3635679 196 -0.308475  0.7580
```

```
## treatment21:pair5   0.5064155 0.3635679 196  1.392905  0.1652
## treatment22:pair5   0.4588950 0.3635679 196  1.262199  0.2084
## treatment23:pair5   0.1842145 0.3635679 196  0.506685  0.6129
## treatment24:pair5   0.3401575 0.3635679 196  0.935609  0.3506
## treatment25:pair5   0.0619884 0.3635679 196  0.170500  0.8648
## treatment26:pair5   0.0265588 0.3635679 196  0.073050  0.9418
## treatment21:pair6   0.0184624 0.3635679 196  0.050781  0.9596
## treatment22:pair6   0.1993848 0.3635679 196  0.548411  0.5840
## treatment23:pair6   0.2831613 0.3635679 196  0.778840  0.4370
## treatment24:pair6   0.4281921 0.3635679 196  1.177750  0.2403
## treatment25:pair6   0.3835305 0.3635679 196  1.054907  0.2928
## treatment26:pair6   0.9102771 0.3635679 196  2.503733  0.0131
## treatment21:pair7   0.0031017 0.3635679 196  0.008531  0.9932
## treatment22:pair7   0.5020634 0.3635679 196  1.380934  0.1689
## treatment23:pair7   0.3033172 0.3635679 196  0.834279  0.4051
## treatment24:pair7   0.4260699 0.3635679 196  1.171913  0.2427
## treatment25:pair7   0.1902331 0.3635679 196  0.523240  0.6014
## treatment26:pair7   0.0963520 0.3635679 196  0.265018  0.7913
## treatment21:pair8   0.1019346 0.3635679 196  0.280373  0.7795
## treatment22:pair8   0.1895008 0.3635679 196  0.521225  0.6028
## treatment23:pair8  -0.2573080 0.3635679 196 -0.707730  0.4800
## treatment24:pair8   0.5959891 0.3635679 196  1.639278  0.1028
## treatment25:pair8   0.9209593 0.3635679 196  2.533115  0.0121
## treatment26:pair8   0.3897224 0.3635679 196  1.071938  0.2851
## treatment21:pair9   0.1790236 0.3635679 196  0.492407  0.6230
## treatment22:pair9  -0.1390359 0.3635679 196 -0.382421  0.7026
## treatment23:pair9  -0.2020541 0.3635679 196 -0.555753  0.5790
## treatment24:pair9   0.2539711 0.3635679 196  0.698552  0.4857
## treatment25:pair9  -0.0032380 0.3635679 196 -0.008906  0.9929
## treatment26:pair9   0.0812490 0.3635679 196  0.223477  0.8234
## treatment21:pair10  0.0510472 0.3635679 196  0.140406  0.8885
## treatment22:pair10 -0.3319216 0.3635679 196 -0.912956  0.3624
## treatment23:pair10  0.2732007 0.3635679 196  0.751443  0.4533
## treatment24:pair10  0.0195331 0.3635679 196  0.053726  0.9572
## treatment25:pair10  0.6879410 0.3635679 196  1.892194  0.0599
## treatment26:pair10 -0.1347573 0.3635679 196 -0.370652  0.7113
## treatment21:pair11  0.8310095 0.3635679 196  2.285706  0.0233
## treatment22:pair11 -0.0774331 0.3635679 196 -0.212981  0.8316
## treatment23:pair11  0.0793267 0.3635679 196  0.218189  0.8275
## treatment24:pair11  0.1380956 0.3635679 196  0.379834  0.7045
## treatment25:pair11  0.3042714 0.3635679 196  0.836904  0.4037
## treatment26:pair11  0.7825821 0.3635679 196  2.152506  0.0326
## treatment21:pair12  0.1603564 0.3635679 196  0.441063  0.6597
## treatment22:pair12  0.3368831 0.3635679 196  0.926603  0.3553
## treatment23:pair12  0.3000542 0.3635679 196  0.825304  0.4102
## treatment24:pair12  0.2152084 0.3635679 196  0.591934  0.5546
## treatment25:pair12  0.3683729 0.3635679 196  1.013216  0.3122
## treatment26:pair12  0.3982628 0.3635679 196  1.095429  0.2747
## treatment21:pair13  0.2811916 0.3635679 196  0.773423  0.4402
## treatment22:pair13  0.0746436 0.3635679 196  0.205309  0.8375
## treatment23:pair13  0.2329755 0.3635679 196  0.640803  0.5224
## treatment24:pair13  0.9136112 0.3635679 196  2.512904  0.0128
## treatment25:pair13  0.7167294 0.3635679 196  1.971377  0.0501
## treatment26:pair13  0.4902767 0.3635679 196  1.348515  0.1790
```

```
## treatment21:pair14 -0.0962233 0.3635679 196 -0.264664  0.7915
## treatment22:pair14  0.1353975 0.3635679 196  0.372413  0.7100
## treatment23:pair14  0.0028598 0.3635679 196  0.007866  0.9937
## treatment24:pair14  0.2854686 0.3635679 196  0.785186  0.4333
## treatment25:pair14 -0.0746930 0.3635679 196 -0.205444  0.8374
## treatment26:pair14  0.1699911 0.3635679 196  0.467563  0.6406
## treatment21:pair15  0.3925459 0.3635679 196  1.079704  0.2816
## treatment22:pair15  0.3083700 0.3635679 196  0.848177  0.3974
## treatment23:pair15  0.1248115 0.3635679 196  0.343296  0.7317
## treatment24:pair15  1.0756849 0.3635679 196  2.958690  0.0035
## treatment25:pair15  0.8085300 0.3635679 196  2.223876  0.0273
## treatment26:pair15  0.0971434 0.3635679 196  0.267195  0.7896
##  Correlation:
##                    (Intr) trtm21 trtm22 trtm23 trtm24 trtm25 trtm26 pair2
## treatment21        -0.707
## treatment22        -0.707  0.500
## treatment23        -0.707  0.500  0.500
## treatment24        -0.707  0.500  0.500  0.500
## treatment25        -0.707  0.500  0.500  0.500  0.500
## treatment26        -0.707  0.500  0.500  0.500  0.500  0.500
## pair2              -0.699  0.494  0.494  0.494  0.494  0.494  0.494
## pair3              -0.699  0.494  0.494  0.494  0.494  0.494  0.494  0.500
## pair4              -0.699  0.494  0.494  0.494  0.494  0.494  0.494  0.500
## pair5              -0.699  0.494  0.494  0.494  0.494  0.494  0.494  0.500
## pair6              -0.699  0.494  0.494  0.494  0.494  0.494  0.494  0.500
## pair7              -0.699  0.494  0.494  0.494  0.494  0.494  0.494  0.500
## pair8              -0.699  0.494  0.494  0.494  0.494  0.494  0.494  0.500
## pair9              -0.699  0.494  0.494  0.494  0.494  0.494  0.494  0.500
## pair10             -0.699  0.494  0.494  0.494  0.494  0.494  0.494  0.500
## pair11             -0.699  0.494  0.494  0.494  0.494  0.494  0.494  0.500
## pair12             -0.699  0.494  0.494  0.494  0.494  0.494  0.494  0.500
## pair13             -0.699  0.494  0.494  0.494  0.494  0.494  0.494  0.500
## pair14             -0.699  0.494  0.494  0.494  0.494  0.494  0.494  0.500
## pair15             -0.699  0.494  0.494  0.494  0.494  0.494  0.494  0.500
## treatment21:pair2   0.494 -0.699 -0.350 -0.350 -0.350 -0.350 -0.350 -0.707
## treatment22:pair2   0.494 -0.350 -0.699 -0.350 -0.350 -0.350 -0.350 -0.707
## treatment23:pair2   0.494 -0.350 -0.350 -0.699 -0.350 -0.350 -0.350 -0.707
## treatment24:pair2   0.494 -0.350 -0.350 -0.350 -0.699 -0.350 -0.350 -0.707
## treatment25:pair2   0.494 -0.350 -0.350 -0.350 -0.350 -0.699 -0.350 -0.707
## treatment26:pair2   0.494 -0.350 -0.350 -0.350 -0.350 -0.350 -0.699 -0.707
## treatment21:pair3   0.494 -0.699 -0.350 -0.350 -0.350 -0.350 -0.350 -0.354
## treatment22:pair3   0.494 -0.350 -0.699 -0.350 -0.350 -0.350 -0.350 -0.354
## treatment23:pair3   0.494 -0.350 -0.350 -0.699 -0.350 -0.350 -0.350 -0.354
## treatment24:pair3   0.494 -0.350 -0.350 -0.350 -0.699 -0.350 -0.350 -0.354
## treatment25:pair3   0.494 -0.350 -0.350 -0.350 -0.350 -0.699 -0.350 -0.354
## treatment26:pair3   0.494 -0.350 -0.350 -0.350 -0.350 -0.350 -0.699 -0.354
## treatment21:pair4   0.494 -0.699 -0.350 -0.350 -0.350 -0.350 -0.350 -0.354
## treatment22:pair4   0.494 -0.350 -0.699 -0.350 -0.350 -0.350 -0.350 -0.354
## treatment23:pair4   0.494 -0.350 -0.350 -0.699 -0.350 -0.350 -0.350 -0.354
## treatment24:pair4   0.494 -0.350 -0.350 -0.350 -0.699 -0.350 -0.350 -0.354
## treatment25:pair4   0.494 -0.350 -0.350 -0.350 -0.350 -0.699 -0.350 -0.354
## treatment26:pair4   0.494 -0.350 -0.350 -0.350 -0.350 -0.350 -0.699 -0.354
## treatment21:pair5   0.494 -0.699 -0.350 -0.350 -0.350 -0.350 -0.350 -0.354
## treatment22:pair5   0.494 -0.350 -0.699 -0.350 -0.350 -0.350 -0.350 -0.354
```

```
## treatment23:pair5   0.494 -0.350 -0.350 -0.699 -0.350 -0.350 -0.350 -0.354
## treatment24:pair5   0.494 -0.350 -0.350 -0.350 -0.699 -0.350 -0.350 -0.354
## treatment25:pair5   0.494 -0.350 -0.350 -0.350 -0.350 -0.699 -0.350 -0.354
## treatment26:pair5   0.494 -0.350 -0.350 -0.350 -0.350 -0.350 -0.699 -0.354
## treatment21:pair6   0.494 -0.699 -0.350 -0.350 -0.350 -0.350 -0.350 -0.354
## treatment22:pair6   0.494 -0.350 -0.699 -0.350 -0.350 -0.350 -0.350 -0.354
## treatment23:pair6   0.494 -0.350 -0.350 -0.699 -0.350 -0.350 -0.350 -0.354
## treatment24:pair6   0.494 -0.350 -0.350 -0.350 -0.699 -0.350 -0.350 -0.354
## treatment25:pair6   0.494 -0.350 -0.350 -0.350 -0.350 -0.699 -0.350 -0.354
## treatment26:pair6   0.494 -0.350 -0.350 -0.350 -0.350 -0.350 -0.699 -0.354
## treatment21:pair7   0.494 -0.699 -0.350 -0.350 -0.350 -0.350 -0.350 -0.354
## treatment22:pair7   0.494 -0.350 -0.699 -0.350 -0.350 -0.350 -0.350 -0.354
## treatment23:pair7   0.494 -0.350 -0.350 -0.699 -0.350 -0.350 -0.350 -0.354
## treatment24:pair7   0.494 -0.350 -0.350 -0.350 -0.699 -0.350 -0.350 -0.354
## treatment25:pair7   0.494 -0.350 -0.350 -0.350 -0.350 -0.699 -0.350 -0.354
## treatment26:pair7   0.494 -0.350 -0.350 -0.350 -0.350 -0.350 -0.699 -0.354
## treatment21:pair8   0.494 -0.699 -0.350 -0.350 -0.350 -0.350 -0.350 -0.354
## treatment22:pair8   0.494 -0.350 -0.699 -0.350 -0.350 -0.350 -0.350 -0.354
## treatment23:pair8   0.494 -0.350 -0.350 -0.699 -0.350 -0.350 -0.350 -0.354
## treatment24:pair8   0.494 -0.350 -0.350 -0.350 -0.699 -0.350 -0.350 -0.354
## treatment25:pair8   0.494 -0.350 -0.350 -0.350 -0.350 -0.699 -0.350 -0.354
## treatment26:pair8   0.494 -0.350 -0.350 -0.350 -0.350 -0.350 -0.699 -0.354
## treatment21:pair9   0.494 -0.699 -0.350 -0.350 -0.350 -0.350 -0.350 -0.354
## treatment22:pair9   0.494 -0.350 -0.699 -0.350 -0.350 -0.350 -0.350 -0.354
## treatment23:pair9   0.494 -0.350 -0.350 -0.699 -0.350 -0.350 -0.350 -0.354
## treatment24:pair9   0.494 -0.350 -0.350 -0.350 -0.699 -0.350 -0.350 -0.354
## treatment25:pair9   0.494 -0.350 -0.350 -0.350 -0.350 -0.699 -0.350 -0.354
## treatment26:pair9   0.494 -0.350 -0.350 -0.350 -0.350 -0.350 -0.699 -0.354
## treatment21:pair10  0.494 -0.699 -0.350 -0.350 -0.350 -0.350 -0.350 -0.354
## treatment22:pair10  0.494 -0.350 -0.699 -0.350 -0.350 -0.350 -0.350 -0.354
## treatment23:pair10  0.494 -0.350 -0.350 -0.699 -0.350 -0.350 -0.350 -0.354
## treatment24:pair10  0.494 -0.350 -0.350 -0.350 -0.699 -0.350 -0.350 -0.354
## treatment25:pair10  0.494 -0.350 -0.350 -0.350 -0.350 -0.699 -0.350 -0.354
## treatment26:pair10  0.494 -0.350 -0.350 -0.350 -0.350 -0.350 -0.699 -0.354
## treatment21:pair11  0.494 -0.699 -0.350 -0.350 -0.350 -0.350 -0.350 -0.354
## treatment22:pair11  0.494 -0.350 -0.699 -0.350 -0.350 -0.350 -0.350 -0.354
## treatment23:pair11  0.494 -0.350 -0.350 -0.699 -0.350 -0.350 -0.350 -0.354
## treatment24:pair11  0.494 -0.350 -0.350 -0.350 -0.699 -0.350 -0.350 -0.354
## treatment25:pair11  0.494 -0.350 -0.350 -0.350 -0.350 -0.699 -0.350 -0.354
## treatment26:pair11  0.494 -0.350 -0.350 -0.350 -0.350 -0.350 -0.699 -0.354
## treatment21:pair12  0.494 -0.699 -0.350 -0.350 -0.350 -0.350 -0.350 -0.354
## treatment22:pair12  0.494 -0.350 -0.699 -0.350 -0.350 -0.350 -0.350 -0.354
## treatment23:pair12  0.494 -0.350 -0.350 -0.699 -0.350 -0.350 -0.350 -0.354
## treatment24:pair12  0.494 -0.350 -0.350 -0.350 -0.699 -0.350 -0.350 -0.354
## treatment25:pair12  0.494 -0.350 -0.350 -0.350 -0.350 -0.699 -0.350 -0.354
## treatment26:pair12  0.494 -0.350 -0.350 -0.350 -0.350 -0.350 -0.699 -0.354
## treatment21:pair13  0.494 -0.699 -0.350 -0.350 -0.350 -0.350 -0.350 -0.354
## treatment22:pair13  0.494 -0.350 -0.699 -0.350 -0.350 -0.350 -0.350 -0.354
## treatment23:pair13  0.494 -0.350 -0.350 -0.699 -0.350 -0.350 -0.350 -0.354
## treatment24:pair13  0.494 -0.350 -0.350 -0.350 -0.699 -0.350 -0.350 -0.354
## treatment25:pair13  0.494 -0.350 -0.350 -0.350 -0.350 -0.699 -0.350 -0.354
## treatment26:pair13  0.494 -0.350 -0.350 -0.350 -0.350 -0.350 -0.699 -0.354
## treatment21:pair14  0.494 -0.699 -0.350 -0.350 -0.350 -0.350 -0.350 -0.354
## treatment22:pair14  0.494 -0.350 -0.699 -0.350 -0.350 -0.350 -0.350 -0.354
```

```
## treatment23:pair14  0.494 -0.350 -0.350 -0.699 -0.350 -0.350 -0.350 -0.354
## treatment24:pair14  0.494 -0.350 -0.350 -0.350 -0.699 -0.350 -0.350 -0.354
## treatment25:pair14  0.494 -0.350 -0.350 -0.350 -0.350 -0.699 -0.350 -0.354
## treatment26:pair14  0.494 -0.350 -0.350 -0.350 -0.350 -0.350 -0.699 -0.354
## treatment21:pair15  0.494 -0.699 -0.350 -0.350 -0.350 -0.350 -0.350 -0.354
## treatment22:pair15  0.494 -0.350 -0.699 -0.350 -0.350 -0.350 -0.350 -0.354
## treatment23:pair15  0.494 -0.350 -0.350 -0.699 -0.350 -0.350 -0.350 -0.354
## treatment24:pair15  0.494 -0.350 -0.350 -0.350 -0.699 -0.350 -0.350 -0.354
## treatment25:pair15  0.494 -0.350 -0.350 -0.350 -0.350 -0.699 -0.350 -0.354
## treatment26:pair15  0.494 -0.350 -0.350 -0.350 -0.350 -0.350 -0.699 -0.354
##                     pair3  pair4  pair5  pair6  pair7  pair8  pair9  pair10
## treatment21
## treatment22
## treatment23
## treatment24
## treatment25
## treatment26
## pair2
## pair3
## pair4                0.500
## pair5                0.500  0.500
## pair6                0.500  0.500  0.500
## pair7                0.500  0.500  0.500  0.500
## pair8                0.500  0.500  0.500  0.500  0.500
## pair9                0.500  0.500  0.500  0.500  0.500  0.500
## pair10               0.500  0.500  0.500  0.500  0.500  0.500  0.500
## pair11               0.500  0.500  0.500  0.500  0.500  0.500  0.500  0.500
## pair12               0.500  0.500  0.500  0.500  0.500  0.500  0.500  0.500
## pair13               0.500  0.500  0.500  0.500  0.500  0.500  0.500  0.500
## pair14               0.500  0.500  0.500  0.500  0.500  0.500  0.500  0.500
## pair15               0.500  0.500  0.500  0.500  0.500  0.500  0.500  0.500
## treatment21:pair2   -0.354 -0.354 -0.354 -0.354 -0.354 -0.354 -0.354 -0.354
## treatment22:pair2   -0.354 -0.354 -0.354 -0.354 -0.354 -0.354 -0.354 -0.354
## treatment23:pair2   -0.354 -0.354 -0.354 -0.354 -0.354 -0.354 -0.354 -0.354
## treatment24:pair2   -0.354 -0.354 -0.354 -0.354 -0.354 -0.354 -0.354 -0.354
## treatment25:pair2   -0.354 -0.354 -0.354 -0.354 -0.354 -0.354 -0.354 -0.354
## treatment26:pair2   -0.354 -0.354 -0.354 -0.354 -0.354 -0.354 -0.354 -0.354
## treatment21:pair3   -0.707 -0.354 -0.354 -0.354 -0.354 -0.354 -0.354 -0.354
## treatment22:pair3   -0.707 -0.354 -0.354 -0.354 -0.354 -0.354 -0.354 -0.354
## treatment23:pair3   -0.707 -0.354 -0.354 -0.354 -0.354 -0.354 -0.354 -0.354
## treatment24:pair3   -0.707 -0.354 -0.354 -0.354 -0.354 -0.354 -0.354 -0.354
## treatment25:pair3   -0.707 -0.354 -0.354 -0.354 -0.354 -0.354 -0.354 -0.354
## treatment26:pair3   -0.707 -0.354 -0.354 -0.354 -0.354 -0.354 -0.354 -0.354
## treatment21:pair4   -0.354 -0.707 -0.354 -0.354 -0.354 -0.354 -0.354 -0.354
## treatment22:pair4   -0.354 -0.707 -0.354 -0.354 -0.354 -0.354 -0.354 -0.354
## treatment23:pair4   -0.354 -0.707 -0.354 -0.354 -0.354 -0.354 -0.354 -0.354
## treatment24:pair4   -0.354 -0.707 -0.354 -0.354 -0.354 -0.354 -0.354 -0.354
## treatment25:pair4   -0.354 -0.707 -0.354 -0.354 -0.354 -0.354 -0.354 -0.354
## treatment26:pair4   -0.354 -0.707 -0.354 -0.354 -0.354 -0.354 -0.354 -0.354
## treatment21:pair5   -0.354 -0.354 -0.707 -0.354 -0.354 -0.354 -0.354 -0.354
## treatment22:pair5   -0.354 -0.354 -0.707 -0.354 -0.354 -0.354 -0.354 -0.354
## treatment23:pair5   -0.354 -0.354 -0.707 -0.354 -0.354 -0.354 -0.354 -0.354
## treatment24:pair5   -0.354 -0.354 -0.707 -0.354 -0.354 -0.354 -0.354 -0.354
## treatment25:pair5   -0.354 -0.354 -0.707 -0.354 -0.354 -0.354 -0.354 -0.354
```

```
## treatment26:pair5  -0.354 -0.354 -0.707 -0.354 -0.354 -0.354 -0.354 -0.354
## treatment21:pair6  -0.354 -0.354 -0.354 -0.707 -0.354 -0.354 -0.354 -0.354
## treatment22:pair6  -0.354 -0.354 -0.354 -0.707 -0.354 -0.354 -0.354 -0.354
## treatment23:pair6  -0.354 -0.354 -0.354 -0.707 -0.354 -0.354 -0.354 -0.354
## treatment24:pair6  -0.354 -0.354 -0.354 -0.707 -0.354 -0.354 -0.354 -0.354
## treatment25:pair6  -0.354 -0.354 -0.354 -0.707 -0.354 -0.354 -0.354 -0.354
## treatment26:pair6  -0.354 -0.354 -0.354 -0.707 -0.354 -0.354 -0.354 -0.354
## treatment21:pair7  -0.354 -0.354 -0.354 -0.354 -0.707 -0.354 -0.354 -0.354
## treatment22:pair7  -0.354 -0.354 -0.354 -0.354 -0.707 -0.354 -0.354 -0.354
## treatment23:pair7  -0.354 -0.354 -0.354 -0.354 -0.707 -0.354 -0.354 -0.354
## treatment24:pair7  -0.354 -0.354 -0.354 -0.354 -0.707 -0.354 -0.354 -0.354
## treatment25:pair7  -0.354 -0.354 -0.354 -0.354 -0.707 -0.354 -0.354 -0.354
## treatment26:pair7  -0.354 -0.354 -0.354 -0.354 -0.707 -0.354 -0.354 -0.354
## treatment21:pair8  -0.354 -0.354 -0.354 -0.354 -0.354 -0.707 -0.354 -0.354
## treatment22:pair8  -0.354 -0.354 -0.354 -0.354 -0.354 -0.707 -0.354 -0.354
## treatment23:pair8  -0.354 -0.354 -0.354 -0.354 -0.354 -0.707 -0.354 -0.354
## treatment24:pair8  -0.354 -0.354 -0.354 -0.354 -0.354 -0.707 -0.354 -0.354
## treatment25:pair8  -0.354 -0.354 -0.354 -0.354 -0.354 -0.707 -0.354 -0.354
## treatment26:pair8  -0.354 -0.354 -0.354 -0.354 -0.354 -0.707 -0.354 -0.354
## treatment21:pair9  -0.354 -0.354 -0.354 -0.354 -0.354 -0.354 -0.707 -0.354
## treatment22:pair9  -0.354 -0.354 -0.354 -0.354 -0.354 -0.354 -0.707 -0.354
## treatment23:pair9  -0.354 -0.354 -0.354 -0.354 -0.354 -0.354 -0.707 -0.354
## treatment24:pair9  -0.354 -0.354 -0.354 -0.354 -0.354 -0.354 -0.707 -0.354
## treatment25:pair9  -0.354 -0.354 -0.354 -0.354 -0.354 -0.354 -0.707 -0.354
## treatment26:pair9  -0.354 -0.354 -0.354 -0.354 -0.354 -0.354 -0.707 -0.354
## treatment21:pair10 -0.354 -0.354 -0.354 -0.354 -0.354 -0.354 -0.354 -0.707
## treatment22:pair10 -0.354 -0.354 -0.354 -0.354 -0.354 -0.354 -0.354 -0.707
## treatment23:pair10 -0.354 -0.354 -0.354 -0.354 -0.354 -0.354 -0.354 -0.707
## treatment24:pair10 -0.354 -0.354 -0.354 -0.354 -0.354 -0.354 -0.354 -0.707
## treatment25:pair10 -0.354 -0.354 -0.354 -0.354 -0.354 -0.354 -0.354 -0.707
## treatment26:pair10 -0.354 -0.354 -0.354 -0.354 -0.354 -0.354 -0.354 -0.707
## treatment21:pair11 -0.354 -0.354 -0.354 -0.354 -0.354 -0.354 -0.354 -0.354
## treatment22:pair11 -0.354 -0.354 -0.354 -0.354 -0.354 -0.354 -0.354 -0.354
## treatment23:pair11 -0.354 -0.354 -0.354 -0.354 -0.354 -0.354 -0.354 -0.354
## treatment24:pair11 -0.354 -0.354 -0.354 -0.354 -0.354 -0.354 -0.354 -0.354
## treatment25:pair11 -0.354 -0.354 -0.354 -0.354 -0.354 -0.354 -0.354 -0.354
## treatment26:pair11 -0.354 -0.354 -0.354 -0.354 -0.354 -0.354 -0.354 -0.354
## treatment21:pair12 -0.354 -0.354 -0.354 -0.354 -0.354 -0.354 -0.354 -0.354
## treatment22:pair12 -0.354 -0.354 -0.354 -0.354 -0.354 -0.354 -0.354 -0.354
## treatment23:pair12 -0.354 -0.354 -0.354 -0.354 -0.354 -0.354 -0.354 -0.354
## treatment24:pair12 -0.354 -0.354 -0.354 -0.354 -0.354 -0.354 -0.354 -0.354
## treatment25:pair12 -0.354 -0.354 -0.354 -0.354 -0.354 -0.354 -0.354 -0.354
## treatment26:pair12 -0.354 -0.354 -0.354 -0.354 -0.354 -0.354 -0.354 -0.354
## treatment21:pair13 -0.354 -0.354 -0.354 -0.354 -0.354 -0.354 -0.354 -0.354
## treatment22:pair13 -0.354 -0.354 -0.354 -0.354 -0.354 -0.354 -0.354 -0.354
## treatment23:pair13 -0.354 -0.354 -0.354 -0.354 -0.354 -0.354 -0.354 -0.354
## treatment24:pair13 -0.354 -0.354 -0.354 -0.354 -0.354 -0.354 -0.354 -0.354
## treatment25:pair13 -0.354 -0.354 -0.354 -0.354 -0.354 -0.354 -0.354 -0.354
## treatment26:pair13 -0.354 -0.354 -0.354 -0.354 -0.354 -0.354 -0.354 -0.354
## treatment21:pair14 -0.354 -0.354 -0.354 -0.354 -0.354 -0.354 -0.354 -0.354
## treatment22:pair14 -0.354 -0.354 -0.354 -0.354 -0.354 -0.354 -0.354 -0.354
## treatment23:pair14 -0.354 -0.354 -0.354 -0.354 -0.354 -0.354 -0.354 -0.354
## treatment24:pair14 -0.354 -0.354 -0.354 -0.354 -0.354 -0.354 -0.354 -0.354
## treatment25:pair14 -0.354 -0.354 -0.354 -0.354 -0.354 -0.354 -0.354 -0.354
```

```
## treatment26:pair14 -0.354 -0.354 -0.354 -0.354 -0.354 -0.354 -0.354 -0.354
## treatment21:pair15 -0.354 -0.354 -0.354 -0.354 -0.354 -0.354 -0.354 -0.354
## treatment22:pair15 -0.354 -0.354 -0.354 -0.354 -0.354 -0.354 -0.354 -0.354
## treatment23:pair15 -0.354 -0.354 -0.354 -0.354 -0.354 -0.354 -0.354 -0.354
## treatment24:pair15 -0.354 -0.354 -0.354 -0.354 -0.354 -0.354 -0.354 -0.354
## treatment25:pair15 -0.354 -0.354 -0.354 -0.354 -0.354 -0.354 -0.354 -0.354
## treatment26:pair15 -0.354 -0.354 -0.354 -0.354 -0.354 -0.354 -0.354 -0.354
##                    pair11 pair12 pair13 pair14 pair15 tr21:2 tr22:2 tr23:2
## treatment21
## treatment22
## treatment23
## treatment24
## treatment25
## treatment26
## pair2
## pair3
## pair4
## pair5
## pair6
## pair7
## pair8
## pair9
## pair10
## pair11
## pair12              0.500
## pair13              0.500  0.500
## pair14              0.500  0.500  0.500
## pair15              0.500  0.500  0.500  0.500
## treatment21:pair2  -0.354 -0.354 -0.354 -0.354 -0.354
## treatment22:pair2  -0.354 -0.354 -0.354 -0.354 -0.354  0.500
## treatment23:pair2  -0.354 -0.354 -0.354 -0.354 -0.354  0.500  0.500
## treatment24:pair2  -0.354 -0.354 -0.354 -0.354 -0.354  0.500  0.500  0.500
## treatment25:pair2  -0.354 -0.354 -0.354 -0.354 -0.354  0.500  0.500  0.500
## treatment26:pair2  -0.354 -0.354 -0.354 -0.354 -0.354  0.500  0.500  0.500
## treatment21:pair3  -0.354 -0.354 -0.354 -0.354 -0.354  0.500  0.250  0.250
## treatment22:pair3  -0.354 -0.354 -0.354 -0.354 -0.354  0.250  0.500  0.250
## treatment23:pair3  -0.354 -0.354 -0.354 -0.354 -0.354  0.250  0.250  0.500
## treatment24:pair3  -0.354 -0.354 -0.354 -0.354 -0.354  0.250  0.250  0.250
## treatment25:pair3  -0.354 -0.354 -0.354 -0.354 -0.354  0.250  0.250  0.250
## treatment26:pair3  -0.354 -0.354 -0.354 -0.354 -0.354  0.250  0.250  0.250
## treatment21:pair4  -0.354 -0.354 -0.354 -0.354 -0.354  0.500  0.250  0.250
## treatment22:pair4  -0.354 -0.354 -0.354 -0.354 -0.354  0.250  0.500  0.250
## treatment23:pair4  -0.354 -0.354 -0.354 -0.354 -0.354  0.250  0.250  0.500
## treatment24:pair4  -0.354 -0.354 -0.354 -0.354 -0.354  0.250  0.250  0.250
## treatment25:pair4  -0.354 -0.354 -0.354 -0.354 -0.354  0.250  0.250  0.250
## treatment26:pair4  -0.354 -0.354 -0.354 -0.354 -0.354  0.250  0.250  0.250
## treatment21:pair5  -0.354 -0.354 -0.354 -0.354 -0.354  0.500  0.250  0.250
## treatment22:pair5  -0.354 -0.354 -0.354 -0.354 -0.354  0.250  0.500  0.250
## treatment23:pair5  -0.354 -0.354 -0.354 -0.354 -0.354  0.250  0.250  0.500
## treatment24:pair5  -0.354 -0.354 -0.354 -0.354 -0.354  0.250  0.250  0.250
## treatment25:pair5  -0.354 -0.354 -0.354 -0.354 -0.354  0.250  0.250  0.250
## treatment26:pair5  -0.354 -0.354 -0.354 -0.354 -0.354  0.250  0.250  0.250
## treatment21:pair6  -0.354 -0.354 -0.354 -0.354 -0.354  0.500  0.250  0.250
## treatment22:pair6  -0.354 -0.354 -0.354 -0.354 -0.354  0.250  0.500  0.250
```

```
## treatment23:pair6  -0.354 -0.354 -0.354 -0.354 -0.354  0.250  0.250  0.500
## treatment24:pair6  -0.354 -0.354 -0.354 -0.354 -0.354  0.250  0.250  0.250
## treatment25:pair6  -0.354 -0.354 -0.354 -0.354 -0.354  0.250  0.250  0.250
## treatment26:pair6  -0.354 -0.354 -0.354 -0.354 -0.354  0.250  0.250  0.250
## treatment21:pair7  -0.354 -0.354 -0.354 -0.354 -0.354  0.500  0.250  0.250
## treatment22:pair7  -0.354 -0.354 -0.354 -0.354 -0.354  0.250  0.500  0.250
## treatment23:pair7  -0.354 -0.354 -0.354 -0.354 -0.354  0.250  0.250  0.500
## treatment24:pair7  -0.354 -0.354 -0.354 -0.354 -0.354  0.250  0.250  0.250
## treatment25:pair7  -0.354 -0.354 -0.354 -0.354 -0.354  0.250  0.250  0.250
## treatment26:pair7  -0.354 -0.354 -0.354 -0.354 -0.354  0.250  0.250  0.250
## treatment21:pair8  -0.354 -0.354 -0.354 -0.354 -0.354  0.500  0.250  0.250
## treatment22:pair8  -0.354 -0.354 -0.354 -0.354 -0.354  0.250  0.500  0.250
## treatment23:pair8  -0.354 -0.354 -0.354 -0.354 -0.354  0.250  0.250  0.500
## treatment24:pair8  -0.354 -0.354 -0.354 -0.354 -0.354  0.250  0.250  0.250
## treatment25:pair8  -0.354 -0.354 -0.354 -0.354 -0.354  0.250  0.250  0.250
## treatment26:pair8  -0.354 -0.354 -0.354 -0.354 -0.354  0.250  0.250  0.250
## treatment21:pair9  -0.354 -0.354 -0.354 -0.354 -0.354  0.500  0.250  0.250
## treatment22:pair9  -0.354 -0.354 -0.354 -0.354 -0.354  0.250  0.500  0.250
## treatment23:pair9  -0.354 -0.354 -0.354 -0.354 -0.354  0.250  0.250  0.500
## treatment24:pair9  -0.354 -0.354 -0.354 -0.354 -0.354  0.250  0.250  0.250
## treatment25:pair9  -0.354 -0.354 -0.354 -0.354 -0.354  0.250  0.250  0.250
## treatment26:pair9  -0.354 -0.354 -0.354 -0.354 -0.354  0.250  0.250  0.250
## treatment21:pair10 -0.354 -0.354 -0.354 -0.354 -0.354  0.500  0.250  0.250
## treatment22:pair10 -0.354 -0.354 -0.354 -0.354 -0.354  0.250  0.500  0.250
## treatment23:pair10 -0.354 -0.354 -0.354 -0.354 -0.354  0.250  0.250  0.500
## treatment24:pair10 -0.354 -0.354 -0.354 -0.354 -0.354  0.250  0.250  0.250
## treatment25:pair10 -0.354 -0.354 -0.354 -0.354 -0.354  0.250  0.250  0.250
## treatment26:pair10 -0.354 -0.354 -0.354 -0.354 -0.354  0.250  0.250  0.250
## treatment21:pair11 -0.707 -0.354 -0.354 -0.354 -0.354  0.500  0.250  0.250
## treatment22:pair11 -0.707 -0.354 -0.354 -0.354 -0.354  0.250  0.500  0.250
## treatment23:pair11 -0.707 -0.354 -0.354 -0.354 -0.354  0.250  0.250  0.500
## treatment24:pair11 -0.707 -0.354 -0.354 -0.354 -0.354  0.250  0.250  0.250
## treatment25:pair11 -0.707 -0.354 -0.354 -0.354 -0.354  0.250  0.250  0.250
## treatment26:pair11 -0.707 -0.354 -0.354 -0.354 -0.354  0.250  0.250  0.250
## treatment21:pair12 -0.354 -0.707 -0.354 -0.354 -0.354  0.500  0.250  0.250
## treatment22:pair12 -0.354 -0.707 -0.354 -0.354 -0.354  0.250  0.500  0.250
## treatment23:pair12 -0.354 -0.707 -0.354 -0.354 -0.354  0.250  0.250  0.500
## treatment24:pair12 -0.354 -0.707 -0.354 -0.354 -0.354  0.250  0.250  0.250
## treatment25:pair12 -0.354 -0.707 -0.354 -0.354 -0.354  0.250  0.250  0.250
## treatment26:pair12 -0.354 -0.707 -0.354 -0.354 -0.354  0.250  0.250  0.250
## treatment21:pair13 -0.354 -0.354 -0.707 -0.354 -0.354  0.500  0.250  0.250
## treatment22:pair13 -0.354 -0.354 -0.707 -0.354 -0.354  0.250  0.500  0.250
## treatment23:pair13 -0.354 -0.354 -0.707 -0.354 -0.354  0.250  0.250  0.500
## treatment24:pair13 -0.354 -0.354 -0.707 -0.354 -0.354  0.250  0.250  0.250
## treatment25:pair13 -0.354 -0.354 -0.707 -0.354 -0.354  0.250  0.250  0.250
## treatment26:pair13 -0.354 -0.354 -0.707 -0.354 -0.354  0.250  0.250  0.250
## treatment21:pair14 -0.354 -0.354 -0.354 -0.707 -0.354  0.500  0.250  0.250
## treatment22:pair14 -0.354 -0.354 -0.354 -0.707 -0.354  0.250  0.500  0.250
## treatment23:pair14 -0.354 -0.354 -0.354 -0.707 -0.354  0.250  0.250  0.500
## treatment24:pair14 -0.354 -0.354 -0.354 -0.707 -0.354  0.250  0.250  0.250
## treatment25:pair14 -0.354 -0.354 -0.354 -0.707 -0.354  0.250  0.250  0.250
## treatment26:pair14 -0.354 -0.354 -0.354 -0.707 -0.354  0.250  0.250  0.250
## treatment21:pair15 -0.354 -0.354 -0.354 -0.354 -0.707  0.500  0.250  0.250
## treatment22:pair15 -0.354 -0.354 -0.354 -0.354 -0.707  0.250  0.500  0.250
```

```
## treatment23:pair15 -0.354 -0.354 -0.354 -0.354 -0.707  0.250  0.250  0.500
## treatment24:pair15 -0.354 -0.354 -0.354 -0.354 -0.707  0.250  0.250  0.250
## treatment25:pair15 -0.354 -0.354 -0.354 -0.354 -0.707  0.250  0.250  0.250
## treatment26:pair15 -0.354 -0.354 -0.354 -0.354 -0.707  0.250  0.250  0.250
##                    tr24:2 tr25:2 tr26:2 tr21:3 tr22:3 tr23:3 tr24:3 tr25:3
## treatment21
## treatment22
## treatment23
## treatment24
## treatment25
## treatment26
## pair2
## pair3
## pair4
## pair5
## pair6
## pair7
## pair8
## pair9
## pair10
## pair11
## pair12
## pair13
## pair14
## pair15
## treatment21:pair2
## treatment22:pair2
## treatment23:pair2
## treatment24:pair2
## treatment25:pair2   0.500
## treatment26:pair2   0.500  0.500
## treatment21:pair3   0.250  0.250  0.250
## treatment22:pair3   0.250  0.250  0.250  0.500
## treatment23:pair3   0.250  0.250  0.250  0.500  0.500
## treatment24:pair3   0.500  0.250  0.250  0.500  0.500  0.500
## treatment25:pair3   0.250  0.500  0.250  0.500  0.500  0.500  0.500
## treatment26:pair3   0.250  0.250  0.500  0.500  0.500  0.500  0.500  0.500
## treatment21:pair4   0.250  0.250  0.250  0.500  0.250  0.250  0.250  0.250
## treatment22:pair4   0.250  0.250  0.250  0.250  0.500  0.250  0.250  0.250
## treatment23:pair4   0.250  0.250  0.250  0.250  0.250  0.500  0.250  0.250
## treatment24:pair4   0.500  0.250  0.250  0.250  0.250  0.250  0.500  0.250
## treatment25:pair4   0.250  0.500  0.250  0.250  0.250  0.250  0.250  0.500
## treatment26:pair4   0.250  0.250  0.500  0.250  0.250  0.250  0.250  0.250
## treatment21:pair5   0.250  0.250  0.250  0.500  0.250  0.250  0.250  0.250
## treatment22:pair5   0.250  0.250  0.250  0.250  0.500  0.250  0.250  0.250
## treatment23:pair5   0.250  0.250  0.250  0.250  0.250  0.500  0.250  0.250
## treatment24:pair5   0.500  0.250  0.250  0.250  0.250  0.250  0.500  0.250
## treatment25:pair5   0.250  0.500  0.250  0.250  0.250  0.250  0.250  0.500
## treatment26:pair5   0.250  0.250  0.500  0.250  0.250  0.250  0.250  0.250
## treatment21:pair6   0.250  0.250  0.250  0.500  0.250  0.250  0.250  0.250
## treatment22:pair6   0.250  0.250  0.250  0.250  0.500  0.250  0.250  0.250
## treatment23:pair6   0.250  0.250  0.250  0.250  0.250  0.500  0.250  0.250
## treatment24:pair6   0.500  0.250  0.250  0.250  0.250  0.250  0.500  0.250
## treatment25:pair6   0.250  0.500  0.250  0.250  0.250  0.250  0.250  0.500
```

```
## treatment26:pair6   0.250  0.250  0.500  0.250  0.250  0.250  0.250  0.250
## treatment21:pair7   0.250  0.250  0.250  0.500  0.250  0.250  0.250  0.250
## treatment22:pair7   0.250  0.250  0.250  0.250  0.500  0.250  0.250  0.250
## treatment23:pair7   0.250  0.250  0.250  0.250  0.250  0.500  0.250  0.250
## treatment24:pair7   0.500  0.250  0.250  0.250  0.250  0.250  0.500  0.250
## treatment25:pair7   0.250  0.500  0.250  0.250  0.250  0.250  0.250  0.500
## treatment26:pair7   0.250  0.250  0.500  0.250  0.250  0.250  0.250  0.250
## treatment21:pair8   0.250  0.250  0.250  0.500  0.250  0.250  0.250  0.250
## treatment22:pair8   0.250  0.250  0.250  0.250  0.500  0.250  0.250  0.250
## treatment23:pair8   0.250  0.250  0.250  0.250  0.250  0.500  0.250  0.250
## treatment24:pair8   0.500  0.250  0.250  0.250  0.250  0.250  0.500  0.250
## treatment25:pair8   0.250  0.500  0.250  0.250  0.250  0.250  0.250  0.500
## treatment26:pair8   0.250  0.250  0.500  0.250  0.250  0.250  0.250  0.250
## treatment21:pair9   0.250  0.250  0.250  0.500  0.250  0.250  0.250  0.250
## treatment22:pair9   0.250  0.250  0.250  0.250  0.500  0.250  0.250  0.250
## treatment23:pair9   0.250  0.250  0.250  0.250  0.250  0.500  0.250  0.250
## treatment24:pair9   0.500  0.250  0.250  0.250  0.250  0.250  0.500  0.250
## treatment25:pair9   0.250  0.500  0.250  0.250  0.250  0.250  0.250  0.500
## treatment26:pair9   0.250  0.250  0.500  0.250  0.250  0.250  0.250  0.250
## treatment21:pair10  0.250  0.250  0.250  0.500  0.250  0.250  0.250  0.250
## treatment22:pair10  0.250  0.250  0.250  0.250  0.500  0.250  0.250  0.250
## treatment23:pair10  0.250  0.250  0.250  0.250  0.250  0.500  0.250  0.250
## treatment24:pair10  0.500  0.250  0.250  0.250  0.250  0.250  0.500  0.250
## treatment25:pair10  0.250  0.500  0.250  0.250  0.250  0.250  0.250  0.500
## treatment26:pair10  0.250  0.250  0.500  0.250  0.250  0.250  0.250  0.250
## treatment21:pair11  0.250  0.250  0.250  0.500  0.250  0.250  0.250  0.250
## treatment22:pair11  0.250  0.250  0.250  0.250  0.500  0.250  0.250  0.250
## treatment23:pair11  0.250  0.250  0.250  0.250  0.250  0.500  0.250  0.250
## treatment24:pair11  0.500  0.250  0.250  0.250  0.250  0.250  0.500  0.250
## treatment25:pair11  0.250  0.500  0.250  0.250  0.250  0.250  0.250  0.500
## treatment26:pair11  0.250  0.250  0.500  0.250  0.250  0.250  0.250  0.250
## treatment21:pair12  0.250  0.250  0.250  0.500  0.250  0.250  0.250  0.250
## treatment22:pair12  0.250  0.250  0.250  0.250  0.500  0.250  0.250  0.250
## treatment23:pair12  0.250  0.250  0.250  0.250  0.250  0.500  0.250  0.250
## treatment24:pair12  0.500  0.250  0.250  0.250  0.250  0.250  0.500  0.250
## treatment25:pair12  0.250  0.500  0.250  0.250  0.250  0.250  0.250  0.500
## treatment26:pair12  0.250  0.250  0.500  0.250  0.250  0.250  0.250  0.250
## treatment21:pair13  0.250  0.250  0.250  0.500  0.250  0.250  0.250  0.250
## treatment22:pair13  0.250  0.250  0.250  0.250  0.500  0.250  0.250  0.250
## treatment23:pair13  0.250  0.250  0.250  0.250  0.250  0.500  0.250  0.250
## treatment24:pair13  0.500  0.250  0.250  0.250  0.250  0.250  0.500  0.250
## treatment25:pair13  0.250  0.500  0.250  0.250  0.250  0.250  0.250  0.500
## treatment26:pair13  0.250  0.250  0.500  0.250  0.250  0.250  0.250  0.250
## treatment21:pair14  0.250  0.250  0.250  0.500  0.250  0.250  0.250  0.250
## treatment22:pair14  0.250  0.250  0.250  0.250  0.500  0.250  0.250  0.250
## treatment23:pair14  0.250  0.250  0.250  0.250  0.250  0.500  0.250  0.250
## treatment24:pair14  0.500  0.250  0.250  0.250  0.250  0.250  0.500  0.250
## treatment25:pair14  0.250  0.500  0.250  0.250  0.250  0.250  0.250  0.500
## treatment26:pair14  0.250  0.250  0.500  0.250  0.250  0.250  0.250  0.250
## treatment21:pair15  0.250  0.250  0.250  0.500  0.250  0.250  0.250  0.250
## treatment22:pair15  0.250  0.250  0.250  0.250  0.500  0.250  0.250  0.250
## treatment23:pair15  0.250  0.250  0.250  0.250  0.250  0.500  0.250  0.250
## treatment24:pair15  0.500  0.250  0.250  0.250  0.250  0.250  0.500  0.250
## treatment25:pair15  0.250  0.500  0.250  0.250  0.250  0.250  0.250  0.500
```

```
## treatment26:pair15  0.250  0.250  0.500  0.250  0.250  0.250  0.250  0.250
##                     tr26:3 tr21:4 tr22:4 tr23:4 tr24:4 tr25:4 tr26:4 tr21:5
## treatment21
## treatment22
## treatment23
## treatment24
## treatment25
## treatment26
## pair2
## pair3
## pair4
## pair5
## pair6
## pair7
## pair8
## pair9
## pair10
## pair11
## pair12
## pair13
## pair14
## pair15
## treatment21:pair2
## treatment22:pair2
## treatment23:pair2
## treatment24:pair2
## treatment25:pair2
## treatment26:pair2
## treatment21:pair3
## treatment22:pair3
## treatment23:pair3
## treatment24:pair3
## treatment25:pair3
## treatment26:pair3
## treatment21:pair4   0.250
## treatment22:pair4   0.250  0.500
## treatment23:pair4   0.250  0.500  0.500
## treatment24:pair4   0.250  0.500  0.500  0.500
## treatment25:pair4   0.250  0.500  0.500  0.500  0.500
## treatment26:pair4   0.500  0.500  0.500  0.500  0.500  0.500
## treatment21:pair5   0.250  0.500  0.250  0.250  0.250  0.250  0.250
## treatment22:pair5   0.250  0.250  0.500  0.250  0.250  0.250  0.250  0.500
## treatment23:pair5   0.250  0.250  0.250  0.500  0.250  0.250  0.250  0.500
## treatment24:pair5   0.250  0.250  0.250  0.250  0.500  0.250  0.250  0.500
## treatment25:pair5   0.250  0.250  0.250  0.250  0.250  0.500  0.250  0.500
## treatment26:pair5   0.500  0.250  0.250  0.250  0.250  0.250  0.500  0.500
## treatment21:pair6   0.250  0.500  0.250  0.250  0.250  0.250  0.250  0.500
## treatment22:pair6   0.250  0.250  0.500  0.250  0.250  0.250  0.250  0.250
## treatment23:pair6   0.250  0.250  0.250  0.500  0.250  0.250  0.250  0.250
## treatment24:pair6   0.250  0.250  0.250  0.250  0.500  0.250  0.250  0.250
## treatment25:pair6   0.250  0.250  0.250  0.250  0.250  0.500  0.250  0.250
## treatment26:pair6   0.500  0.250  0.250  0.250  0.250  0.250  0.500  0.250
## treatment21:pair7   0.250  0.500  0.250  0.250  0.250  0.250  0.250  0.500
## treatment22:pair7   0.250  0.250  0.500  0.250  0.250  0.250  0.250  0.250
```

```
## treatment23:pair7   0.250  0.250  0.250  0.500  0.250  0.250  0.250  0.250
## treatment24:pair7   0.250  0.250  0.250  0.250  0.500  0.250  0.250  0.250
## treatment25:pair7   0.250  0.250  0.250  0.250  0.250  0.500  0.250  0.250
## treatment26:pair7   0.500  0.250  0.250  0.250  0.250  0.250  0.500  0.250
## treatment21:pair8   0.250  0.500  0.250  0.250  0.250  0.250  0.250  0.500
## treatment22:pair8   0.250  0.250  0.500  0.250  0.250  0.250  0.250  0.250
## treatment23:pair8   0.250  0.250  0.250  0.500  0.250  0.250  0.250  0.250
## treatment24:pair8   0.250  0.250  0.250  0.250  0.500  0.250  0.250  0.250
## treatment25:pair8   0.250  0.250  0.250  0.250  0.250  0.500  0.250  0.250
## treatment26:pair8   0.500  0.250  0.250  0.250  0.250  0.250  0.500  0.250
## treatment21:pair9   0.250  0.500  0.250  0.250  0.250  0.250  0.250  0.500
## treatment22:pair9   0.250  0.250  0.500  0.250  0.250  0.250  0.250  0.250
## treatment23:pair9   0.250  0.250  0.250  0.500  0.250  0.250  0.250  0.250
## treatment24:pair9   0.250  0.250  0.250  0.250  0.500  0.250  0.250  0.250
## treatment25:pair9   0.250  0.250  0.250  0.250  0.250  0.500  0.250  0.250
## treatment26:pair9   0.500  0.250  0.250  0.250  0.250  0.250  0.500  0.250
## treatment21:pair10  0.250  0.500  0.250  0.250  0.250  0.250  0.250  0.500
## treatment22:pair10  0.250  0.250  0.500  0.250  0.250  0.250  0.250  0.250
## treatment23:pair10  0.250  0.250  0.250  0.500  0.250  0.250  0.250  0.250
## treatment24:pair10  0.250  0.250  0.250  0.250  0.500  0.250  0.250  0.250
## treatment25:pair10  0.250  0.250  0.250  0.250  0.250  0.500  0.250  0.250
## treatment26:pair10  0.500  0.250  0.250  0.250  0.250  0.250  0.500  0.250
## treatment21:pair11  0.250  0.500  0.250  0.250  0.250  0.250  0.250  0.500
## treatment22:pair11  0.250  0.250  0.500  0.250  0.250  0.250  0.250  0.250
## treatment23:pair11  0.250  0.250  0.250  0.500  0.250  0.250  0.250  0.250
## treatment24:pair11  0.250  0.250  0.250  0.250  0.500  0.250  0.250  0.250
## treatment25:pair11  0.250  0.250  0.250  0.250  0.250  0.500  0.250  0.250
## treatment26:pair11  0.500  0.250  0.250  0.250  0.250  0.250  0.500  0.250
## treatment21:pair12  0.250  0.500  0.250  0.250  0.250  0.250  0.250  0.500
## treatment22:pair12  0.250  0.250  0.500  0.250  0.250  0.250  0.250  0.250
## treatment23:pair12  0.250  0.250  0.250  0.500  0.250  0.250  0.250  0.250
## treatment24:pair12  0.250  0.250  0.250  0.250  0.500  0.250  0.250  0.250
## treatment25:pair12  0.250  0.250  0.250  0.250  0.250  0.500  0.250  0.250
## treatment26:pair12  0.500  0.250  0.250  0.250  0.250  0.250  0.500  0.250
## treatment21:pair13  0.250  0.500  0.250  0.250  0.250  0.250  0.250  0.500
## treatment22:pair13  0.250  0.250  0.500  0.250  0.250  0.250  0.250  0.250
## treatment23:pair13  0.250  0.250  0.250  0.500  0.250  0.250  0.250  0.250
## treatment24:pair13  0.250  0.250  0.250  0.250  0.500  0.250  0.250  0.250
## treatment25:pair13  0.250  0.250  0.250  0.250  0.250  0.500  0.250  0.250
## treatment26:pair13  0.500  0.250  0.250  0.250  0.250  0.250  0.500  0.250
## treatment21:pair14  0.250  0.500  0.250  0.250  0.250  0.250  0.250  0.500
## treatment22:pair14  0.250  0.250  0.500  0.250  0.250  0.250  0.250  0.250
## treatment23:pair14  0.250  0.250  0.250  0.500  0.250  0.250  0.250  0.250
## treatment24:pair14  0.250  0.250  0.250  0.250  0.500  0.250  0.250  0.250
## treatment25:pair14  0.250  0.250  0.250  0.250  0.250  0.500  0.250  0.250
## treatment26:pair14  0.500  0.250  0.250  0.250  0.250  0.250  0.500  0.250
## treatment21:pair15  0.250  0.500  0.250  0.250  0.250  0.250  0.250  0.500
## treatment22:pair15  0.250  0.250  0.500  0.250  0.250  0.250  0.250  0.250
## treatment23:pair15  0.250  0.250  0.250  0.500  0.250  0.250  0.250  0.250
## treatment24:pair15  0.250  0.250  0.250  0.250  0.500  0.250  0.250  0.250
## treatment25:pair15  0.250  0.250  0.250  0.250  0.250  0.500  0.250  0.250
## treatment26:pair15  0.500  0.250  0.250  0.250  0.250  0.250  0.500  0.250
##                    tr22:5 tr23:5 tr24:5 tr25:5 tr26:5 tr21:6 tr22:6 tr23:6
## treatment21
```

```
## treatment22
## treatment23
## treatment24
## treatment25
## treatment26
## pair2
## pair3
## pair4
## pair5
## pair6
## pair7
## pair8
## pair9
## pair10
## pair11
## pair12
## pair13
## pair14
## pair15
## treatment21:pair2
## treatment22:pair2
## treatment23:pair2
## treatment24:pair2
## treatment25:pair2
## treatment26:pair2
## treatment21:pair3
## treatment22:pair3
## treatment23:pair3
## treatment24:pair3
## treatment25:pair3
## treatment26:pair3
## treatment21:pair4
## treatment22:pair4
## treatment23:pair4
## treatment24:pair4
## treatment25:pair4
## treatment26:pair4
## treatment21:pair5
## treatment22:pair5
## treatment23:pair5   0.500
## treatment24:pair5   0.500  0.500
## treatment25:pair5   0.500  0.500  0.500
## treatment26:pair5   0.500  0.500  0.500  0.500
## treatment21:pair6   0.250  0.250  0.250  0.250  0.250
## treatment22:pair6   0.500  0.250  0.250  0.250  0.250  0.500
## treatment23:pair6   0.250  0.500  0.250  0.250  0.250  0.500  0.500
## treatment24:pair6   0.250  0.250  0.500  0.250  0.250  0.500  0.500  0.500
## treatment25:pair6   0.250  0.250  0.250  0.500  0.250  0.500  0.500  0.500
## treatment26:pair6   0.250  0.250  0.250  0.250  0.500  0.500  0.500  0.500
## treatment21:pair7   0.250  0.250  0.250  0.250  0.250  0.500  0.250  0.250
## treatment22:pair7   0.500  0.250  0.250  0.250  0.250  0.250  0.500  0.250
## treatment23:pair7   0.250  0.500  0.250  0.250  0.250  0.250  0.250  0.500
## treatment24:pair7   0.250  0.250  0.500  0.250  0.250  0.250  0.250  0.250
## treatment25:pair7   0.250  0.250  0.250  0.500  0.250  0.250  0.250  0.250
```

```
## treatment26:pair7   0.250  0.250  0.250  0.250  0.500  0.250  0.250  0.250
## treatment21:pair8   0.250  0.250  0.250  0.250  0.250  0.500  0.250  0.250
## treatment22:pair8   0.500  0.250  0.250  0.250  0.250  0.250  0.500  0.250
## treatment23:pair8   0.250  0.500  0.250  0.250  0.250  0.250  0.250  0.500
## treatment24:pair8   0.250  0.250  0.500  0.250  0.250  0.250  0.250  0.250
## treatment25:pair8   0.250  0.250  0.250  0.500  0.250  0.250  0.250  0.250
## treatment26:pair8   0.250  0.250  0.250  0.250  0.500  0.250  0.250  0.250
## treatment21:pair9   0.250  0.250  0.250  0.250  0.250  0.500  0.250  0.250
## treatment22:pair9   0.500  0.250  0.250  0.250  0.250  0.250  0.500  0.250
## treatment23:pair9   0.250  0.500  0.250  0.250  0.250  0.250  0.250  0.500
## treatment24:pair9   0.250  0.250  0.500  0.250  0.250  0.250  0.250  0.250
## treatment25:pair9   0.250  0.250  0.250  0.500  0.250  0.250  0.250  0.250
## treatment26:pair9   0.250  0.250  0.250  0.250  0.500  0.250  0.250  0.250
## treatment21:pair10  0.250  0.250  0.250  0.250  0.250  0.500  0.250  0.250
## treatment22:pair10  0.500  0.250  0.250  0.250  0.250  0.250  0.500  0.250
## treatment23:pair10  0.250  0.500  0.250  0.250  0.250  0.250  0.250  0.500
## treatment24:pair10  0.250  0.250  0.500  0.250  0.250  0.250  0.250  0.250
## treatment25:pair10  0.250  0.250  0.250  0.500  0.250  0.250  0.250  0.250
## treatment26:pair10  0.250  0.250  0.250  0.250  0.500  0.250  0.250  0.250
## treatment21:pair11  0.250  0.250  0.250  0.250  0.250  0.500  0.250  0.250
## treatment22:pair11  0.500  0.250  0.250  0.250  0.250  0.250  0.500  0.250
## treatment23:pair11  0.250  0.500  0.250  0.250  0.250  0.250  0.250  0.500
## treatment24:pair11  0.250  0.250  0.500  0.250  0.250  0.250  0.250  0.250
## treatment25:pair11  0.250  0.250  0.250  0.500  0.250  0.250  0.250  0.250
## treatment26:pair11  0.250  0.250  0.250  0.250  0.500  0.250  0.250  0.250
## treatment21:pair12  0.250  0.250  0.250  0.250  0.250  0.500  0.250  0.250
## treatment22:pair12  0.500  0.250  0.250  0.250  0.250  0.250  0.500  0.250
## treatment23:pair12  0.250  0.500  0.250  0.250  0.250  0.250  0.250  0.500
## treatment24:pair12  0.250  0.250  0.500  0.250  0.250  0.250  0.250  0.250
## treatment25:pair12  0.250  0.250  0.250  0.500  0.250  0.250  0.250  0.250
## treatment26:pair12  0.250  0.250  0.250  0.250  0.500  0.250  0.250  0.250
## treatment21:pair13  0.250  0.250  0.250  0.250  0.250  0.500  0.250  0.250
## treatment22:pair13  0.500  0.250  0.250  0.250  0.250  0.250  0.500  0.250
## treatment23:pair13  0.250  0.500  0.250  0.250  0.250  0.250  0.250  0.500
## treatment24:pair13  0.250  0.250  0.500  0.250  0.250  0.250  0.250  0.250
## treatment25:pair13  0.250  0.250  0.250  0.500  0.250  0.250  0.250  0.250
## treatment26:pair13  0.250  0.250  0.250  0.250  0.500  0.250  0.250  0.250
## treatment21:pair14  0.250  0.250  0.250  0.250  0.250  0.500  0.250  0.250
## treatment22:pair14  0.500  0.250  0.250  0.250  0.250  0.250  0.500  0.250
## treatment23:pair14  0.250  0.500  0.250  0.250  0.250  0.250  0.250  0.500
## treatment24:pair14  0.250  0.250  0.500  0.250  0.250  0.250  0.250  0.250
## treatment25:pair14  0.250  0.250  0.250  0.500  0.250  0.250  0.250  0.250
## treatment26:pair14  0.250  0.250  0.250  0.250  0.500  0.250  0.250  0.250
## treatment21:pair15  0.250  0.250  0.250  0.250  0.250  0.500  0.250  0.250
## treatment22:pair15  0.500  0.250  0.250  0.250  0.250  0.250  0.500  0.250
## treatment23:pair15  0.250  0.500  0.250  0.250  0.250  0.250  0.250  0.500
## treatment24:pair15  0.250  0.250  0.500  0.250  0.250  0.250  0.250  0.250
## treatment25:pair15  0.250  0.250  0.250  0.500  0.250  0.250  0.250  0.250
## treatment26:pair15  0.250  0.250  0.250  0.250  0.500  0.250  0.250  0.250
##                    tr24:6 tr25:6 tr26:6 tr21:7 tr22:7 tr23:7 tr24:7 tr25:7
## treatment21
## treatment22
## treatment23
## treatment24
```

```
## treatment25
## treatment26
## pair2
## pair3
## pair4
## pair5
## pair6
## pair7
## pair8
## pair9
## pair10
## pair11
## pair12
## pair13
## pair14
## pair15
## treatment21:pair2
## treatment22:pair2
## treatment23:pair2
## treatment24:pair2
## treatment25:pair2
## treatment26:pair2
## treatment21:pair3
## treatment22:pair3
## treatment23:pair3
## treatment24:pair3
## treatment25:pair3
## treatment26:pair3
## treatment21:pair4
## treatment22:pair4
## treatment23:pair4
## treatment24:pair4
## treatment25:pair4
## treatment26:pair4
## treatment21:pair5
## treatment22:pair5
## treatment23:pair5
## treatment24:pair5
## treatment25:pair5
## treatment26:pair5
## treatment21:pair6
## treatment22:pair6
## treatment23:pair6
## treatment24:pair6
## treatment25:pair6   0.500
## treatment26:pair6   0.500  0.500
## treatment21:pair7   0.250  0.250  0.250
## treatment22:pair7   0.250  0.250  0.250  0.500
## treatment23:pair7   0.250  0.250  0.250  0.500  0.500
## treatment24:pair7   0.500  0.250  0.250  0.500  0.500  0.500
## treatment25:pair7   0.250  0.500  0.250  0.500  0.500  0.500  0.500
## treatment26:pair7   0.250  0.250  0.500  0.500  0.500  0.500  0.500  0.500
## treatment21:pair8   0.250  0.250  0.250  0.500  0.250  0.250  0.250  0.250
## treatment22:pair8   0.250  0.250  0.250  0.250  0.500  0.250  0.250  0.250
```

```
## treatment23:pair8   0.250  0.250  0.250  0.250  0.250  0.500  0.250  0.250
## treatment24:pair8   0.500  0.250  0.250  0.250  0.250  0.250  0.500  0.250
## treatment25:pair8   0.250  0.500  0.250  0.250  0.250  0.250  0.250  0.500
## treatment26:pair8   0.250  0.250  0.500  0.250  0.250  0.250  0.250  0.250
## treatment21:pair9   0.250  0.250  0.250  0.500  0.250  0.250  0.250  0.250
## treatment22:pair9   0.250  0.250  0.250  0.250  0.500  0.250  0.250  0.250
## treatment23:pair9   0.250  0.250  0.250  0.250  0.250  0.500  0.250  0.250
## treatment24:pair9   0.500  0.250  0.250  0.250  0.250  0.250  0.500  0.250
## treatment25:pair9   0.250  0.500  0.250  0.250  0.250  0.250  0.250  0.500
## treatment26:pair9   0.250  0.250  0.500  0.250  0.250  0.250  0.250  0.250
## treatment21:pair10  0.250  0.250  0.250  0.500  0.250  0.250  0.250  0.250
## treatment22:pair10  0.250  0.250  0.250  0.250  0.500  0.250  0.250  0.250
## treatment23:pair10  0.250  0.250  0.250  0.250  0.250  0.500  0.250  0.250
## treatment24:pair10  0.500  0.250  0.250  0.250  0.250  0.250  0.500  0.250
## treatment25:pair10  0.250  0.500  0.250  0.250  0.250  0.250  0.250  0.500
## treatment26:pair10  0.250  0.250  0.500  0.250  0.250  0.250  0.250  0.250
## treatment21:pair11  0.250  0.250  0.250  0.500  0.250  0.250  0.250  0.250
## treatment22:pair11  0.250  0.250  0.250  0.250  0.500  0.250  0.250  0.250
## treatment23:pair11  0.250  0.250  0.250  0.250  0.250  0.500  0.250  0.250
## treatment24:pair11  0.500  0.250  0.250  0.250  0.250  0.250  0.500  0.250
## treatment25:pair11  0.250  0.500  0.250  0.250  0.250  0.250  0.250  0.500
## treatment26:pair11  0.250  0.250  0.500  0.250  0.250  0.250  0.250  0.250
## treatment21:pair12  0.250  0.250  0.250  0.500  0.250  0.250  0.250  0.250
## treatment22:pair12  0.250  0.250  0.250  0.250  0.500  0.250  0.250  0.250
## treatment23:pair12  0.250  0.250  0.250  0.250  0.250  0.500  0.250  0.250
## treatment24:pair12  0.500  0.250  0.250  0.250  0.250  0.250  0.500  0.250
## treatment25:pair12  0.250  0.500  0.250  0.250  0.250  0.250  0.250  0.500
## treatment26:pair12  0.250  0.250  0.500  0.250  0.250  0.250  0.250  0.250
## treatment21:pair13  0.250  0.250  0.250  0.500  0.250  0.250  0.250  0.250
## treatment22:pair13  0.250  0.250  0.250  0.250  0.500  0.250  0.250  0.250
## treatment23:pair13  0.250  0.250  0.250  0.250  0.250  0.500  0.250  0.250
## treatment24:pair13  0.500  0.250  0.250  0.250  0.250  0.250  0.500  0.250
## treatment25:pair13  0.250  0.500  0.250  0.250  0.250  0.250  0.250  0.500
## treatment26:pair13  0.250  0.250  0.500  0.250  0.250  0.250  0.250  0.250
## treatment21:pair14  0.250  0.250  0.250  0.500  0.250  0.250  0.250  0.250
## treatment22:pair14  0.250  0.250  0.250  0.250  0.500  0.250  0.250  0.250
## treatment23:pair14  0.250  0.250  0.250  0.250  0.250  0.500  0.250  0.250
## treatment24:pair14  0.500  0.250  0.250  0.250  0.250  0.250  0.500  0.250
## treatment25:pair14  0.250  0.500  0.250  0.250  0.250  0.250  0.250  0.500
## treatment26:pair14  0.250  0.250  0.500  0.250  0.250  0.250  0.250  0.250
## treatment21:pair15  0.250  0.250  0.250  0.500  0.250  0.250  0.250  0.250
## treatment22:pair15  0.250  0.250  0.250  0.250  0.500  0.250  0.250  0.250
## treatment23:pair15  0.250  0.250  0.250  0.250  0.250  0.500  0.250  0.250
## treatment24:pair15  0.500  0.250  0.250  0.250  0.250  0.250  0.500  0.250
## treatment25:pair15  0.250  0.500  0.250  0.250  0.250  0.250  0.250  0.500
## treatment26:pair15  0.250  0.250  0.500  0.250  0.250  0.250  0.250  0.250
##                    tr26:7 tr21:8 tr22:8 tr23:8 tr24:8 tr25:8 tr26:8 tr21:9
## treatment21
## treatment22
## treatment23
## treatment24
## treatment25
## treatment26
## pair2
```

```
## pair3
## pair4
## pair5
## pair6
## pair7
## pair8
## pair9
## pair10
## pair11
## pair12
## pair13
## pair14
## pair15
## treatment21:pair2
## treatment22:pair2
## treatment23:pair2
## treatment24:pair2
## treatment25:pair2
## treatment26:pair2
## treatment21:pair3
## treatment22:pair3
## treatment23:pair3
## treatment24:pair3
## treatment25:pair3
## treatment26:pair3
## treatment21:pair4
## treatment22:pair4
## treatment23:pair4
## treatment24:pair4
## treatment25:pair4
## treatment26:pair4
## treatment21:pair5
## treatment22:pair5
## treatment23:pair5
## treatment24:pair5
## treatment25:pair5
## treatment26:pair5
## treatment21:pair6
## treatment22:pair6
## treatment23:pair6
## treatment24:pair6
## treatment25:pair6
## treatment26:pair6
## treatment21:pair7
## treatment22:pair7
## treatment23:pair7
## treatment24:pair7
## treatment25:pair7
## treatment26:pair7
## treatment21:pair8   0.250
## treatment22:pair8   0.250  0.500
## treatment23:pair8   0.250  0.500  0.500
## treatment24:pair8   0.250  0.500  0.500  0.500
## treatment25:pair8   0.250  0.500  0.500  0.500  0.500
```

```
## treatment26:pair8   0.500  0.500  0.500  0.500  0.500  0.500
## treatment21:pair9   0.250  0.500  0.250  0.250  0.250  0.250  0.250
## treatment22:pair9   0.250  0.250  0.500  0.250  0.250  0.250  0.250  0.500
## treatment23:pair9   0.250  0.250  0.250  0.500  0.250  0.250  0.250  0.500
## treatment24:pair9   0.250  0.250  0.250  0.250  0.500  0.250  0.250  0.500
## treatment25:pair9   0.250  0.250  0.250  0.250  0.250  0.500  0.250  0.500
## treatment26:pair9   0.500  0.250  0.250  0.250  0.250  0.250  0.500  0.500
## treatment21:pair10  0.250  0.500  0.250  0.250  0.250  0.250  0.250  0.500
## treatment22:pair10  0.250  0.250  0.500  0.250  0.250  0.250  0.250  0.250
## treatment23:pair10  0.250  0.250  0.250  0.500  0.250  0.250  0.250  0.250
## treatment24:pair10  0.250  0.250  0.250  0.250  0.500  0.250  0.250  0.250
## treatment25:pair10  0.250  0.250  0.250  0.250  0.250  0.500  0.250  0.250
## treatment26:pair10  0.500  0.250  0.250  0.250  0.250  0.250  0.500  0.250
## treatment21:pair11  0.250  0.500  0.250  0.250  0.250  0.250  0.250  0.500
## treatment22:pair11  0.250  0.250  0.500  0.250  0.250  0.250  0.250  0.250
## treatment23:pair11  0.250  0.250  0.250  0.500  0.250  0.250  0.250  0.250
## treatment24:pair11  0.250  0.250  0.250  0.250  0.500  0.250  0.250  0.250
## treatment25:pair11  0.250  0.250  0.250  0.250  0.250  0.500  0.250  0.250
## treatment26:pair11  0.500  0.250  0.250  0.250  0.250  0.250  0.500  0.250
## treatment21:pair12  0.250  0.500  0.250  0.250  0.250  0.250  0.250  0.500
## treatment22:pair12  0.250  0.250  0.500  0.250  0.250  0.250  0.250  0.250
## treatment23:pair12  0.250  0.250  0.250  0.500  0.250  0.250  0.250  0.250
## treatment24:pair12  0.250  0.250  0.250  0.250  0.500  0.250  0.250  0.250
## treatment25:pair12  0.250  0.250  0.250  0.250  0.250  0.500  0.250  0.250
## treatment26:pair12  0.500  0.250  0.250  0.250  0.250  0.250  0.500  0.250
## treatment21:pair13  0.250  0.500  0.250  0.250  0.250  0.250  0.250  0.500
## treatment22:pair13  0.250  0.250  0.500  0.250  0.250  0.250  0.250  0.250
## treatment23:pair13  0.250  0.250  0.250  0.500  0.250  0.250  0.250  0.250
## treatment24:pair13  0.250  0.250  0.250  0.250  0.500  0.250  0.250  0.250
## treatment25:pair13  0.250  0.250  0.250  0.250  0.250  0.500  0.250  0.250
## treatment26:pair13  0.500  0.250  0.250  0.250  0.250  0.250  0.500  0.250
## treatment21:pair14  0.250  0.500  0.250  0.250  0.250  0.250  0.250  0.500
## treatment22:pair14  0.250  0.250  0.500  0.250  0.250  0.250  0.250  0.250
## treatment23:pair14  0.250  0.250  0.250  0.500  0.250  0.250  0.250  0.250
## treatment24:pair14  0.250  0.250  0.250  0.250  0.500  0.250  0.250  0.250
## treatment25:pair14  0.250  0.250  0.250  0.250  0.250  0.500  0.250  0.250
## treatment26:pair14  0.500  0.250  0.250  0.250  0.250  0.250  0.500  0.250
## treatment21:pair15  0.250  0.500  0.250  0.250  0.250  0.250  0.250  0.500
## treatment22:pair15  0.250  0.250  0.500  0.250  0.250  0.250  0.250  0.250
## treatment23:pair15  0.250  0.250  0.250  0.500  0.250  0.250  0.250  0.250
## treatment24:pair15  0.250  0.250  0.250  0.250  0.500  0.250  0.250  0.250
## treatment25:pair15  0.250  0.250  0.250  0.250  0.250  0.500  0.250  0.250
## treatment26:pair15  0.500  0.250  0.250  0.250  0.250  0.250  0.500  0.250
##                    tr22:9 tr23:9 tr24:9 tr25:9 tr26:9 t21:10 t22:10 t23:10
## treatment21
## treatment22
## treatment23
## treatment24
## treatment25
## treatment26
## pair2
## pair3
## pair4
## pair5
```

```
## pair6
## pair7
## pair8
## pair9
## pair10
## pair11
## pair12
## pair13
## pair14
## pair15
## treatment21:pair2
## treatment22:pair2
## treatment23:pair2
## treatment24:pair2
## treatment25:pair2
## treatment26:pair2
## treatment21:pair3
## treatment22:pair3
## treatment23:pair3
## treatment24:pair3
## treatment25:pair3
## treatment26:pair3
## treatment21:pair4
## treatment22:pair4
## treatment23:pair4
## treatment24:pair4
## treatment25:pair4
## treatment26:pair4
## treatment21:pair5
## treatment22:pair5
## treatment23:pair5
## treatment24:pair5
## treatment25:pair5
## treatment26:pair5
## treatment21:pair6
## treatment22:pair6
## treatment23:pair6
## treatment24:pair6
## treatment25:pair6
## treatment26:pair6
## treatment21:pair7
## treatment22:pair7
## treatment23:pair7
## treatment24:pair7
## treatment25:pair7
## treatment26:pair7
## treatment21:pair8
## treatment22:pair8
## treatment23:pair8
## treatment24:pair8
## treatment25:pair8
## treatment26:pair8
## treatment21:pair9
## treatment22:pair9
```

```
## treatment23:pair9   0.500
## treatment24:pair9   0.500  0.500
## treatment25:pair9   0.500  0.500  0.500
## treatment26:pair9   0.500  0.500  0.500  0.500
## treatment21:pair10  0.250  0.250  0.250  0.250  0.250
## treatment22:pair10  0.500  0.250  0.250  0.250  0.250  0.500
## treatment23:pair10  0.250  0.500  0.250  0.250  0.250  0.500  0.500
## treatment24:pair10  0.250  0.250  0.500  0.250  0.250  0.500  0.500  0.500
## treatment25:pair10  0.250  0.250  0.250  0.500  0.250  0.500  0.500  0.500
## treatment26:pair10  0.250  0.250  0.250  0.250  0.500  0.500  0.500  0.500
## treatment21:pair11  0.250  0.250  0.250  0.250  0.250  0.500  0.250  0.250
## treatment22:pair11  0.500  0.250  0.250  0.250  0.250  0.250  0.500  0.250
## treatment23:pair11  0.250  0.500  0.250  0.250  0.250  0.250  0.250  0.500
## treatment24:pair11  0.250  0.250  0.500  0.250  0.250  0.250  0.250  0.250
## treatment25:pair11  0.250  0.250  0.250  0.500  0.250  0.250  0.250  0.250
## treatment26:pair11  0.250  0.250  0.250  0.250  0.500  0.250  0.250  0.250
## treatment21:pair12  0.250  0.250  0.250  0.250  0.250  0.500  0.250  0.250
## treatment22:pair12  0.500  0.250  0.250  0.250  0.250  0.250  0.500  0.250
## treatment23:pair12  0.250  0.500  0.250  0.250  0.250  0.250  0.250  0.500
## treatment24:pair12  0.250  0.250  0.500  0.250  0.250  0.250  0.250  0.250
## treatment25:pair12  0.250  0.250  0.250  0.500  0.250  0.250  0.250  0.250
## treatment26:pair12  0.250  0.250  0.250  0.250  0.500  0.250  0.250  0.250
## treatment21:pair13  0.250  0.250  0.250  0.250  0.250  0.500  0.250  0.250
## treatment22:pair13  0.500  0.250  0.250  0.250  0.250  0.250  0.500  0.250
## treatment23:pair13  0.250  0.500  0.250  0.250  0.250  0.250  0.250  0.500
## treatment24:pair13  0.250  0.250  0.500  0.250  0.250  0.250  0.250  0.250
## treatment25:pair13  0.250  0.250  0.250  0.500  0.250  0.250  0.250  0.250
## treatment26:pair13  0.250  0.250  0.250  0.250  0.500  0.250  0.250  0.250
## treatment21:pair14  0.250  0.250  0.250  0.250  0.250  0.500  0.250  0.250
## treatment22:pair14  0.500  0.250  0.250  0.250  0.250  0.250  0.500  0.250
## treatment23:pair14  0.250  0.500  0.250  0.250  0.250  0.250  0.250  0.500
## treatment24:pair14  0.250  0.250  0.500  0.250  0.250  0.250  0.250  0.250
## treatment25:pair14  0.250  0.250  0.250  0.500  0.250  0.250  0.250  0.250
## treatment26:pair14  0.250  0.250  0.250  0.250  0.500  0.250  0.250  0.250
## treatment21:pair15  0.250  0.250  0.250  0.250  0.250  0.500  0.250  0.250
## treatment22:pair15  0.500  0.250  0.250  0.250  0.250  0.250  0.500  0.250
## treatment23:pair15  0.250  0.500  0.250  0.250  0.250  0.250  0.250  0.500
## treatment24:pair15  0.250  0.250  0.500  0.250  0.250  0.250  0.250  0.250
## treatment25:pair15  0.250  0.250  0.250  0.500  0.250  0.250  0.250  0.250
## treatment26:pair15  0.250  0.250  0.250  0.250  0.500  0.250  0.250  0.250
##                    t24:10 t25:10 t26:10 t21:11 t22:11 t23:11 t24:11 t25:11
## treatment21
## treatment22
## treatment23
## treatment24
## treatment25
## treatment26
## pair2
## pair3
## pair4
## pair5
## pair6
## pair7
## pair8
```

```
## pair9
## pair10
## pair11
## pair12
## pair13
## pair14
## pair15
## treatment21:pair2
## treatment22:pair2
## treatment23:pair2
## treatment24:pair2
## treatment25:pair2
## treatment26:pair2
## treatment21:pair3
## treatment22:pair3
## treatment23:pair3
## treatment24:pair3
## treatment25:pair3
## treatment26:pair3
## treatment21:pair4
## treatment22:pair4
## treatment23:pair4
## treatment24:pair4
## treatment25:pair4
## treatment26:pair4
## treatment21:pair5
## treatment22:pair5
## treatment23:pair5
## treatment24:pair5
## treatment25:pair5
## treatment26:pair5
## treatment21:pair6
## treatment22:pair6
## treatment23:pair6
## treatment24:pair6
## treatment25:pair6
## treatment26:pair6
## treatment21:pair7
## treatment22:pair7
## treatment23:pair7
## treatment24:pair7
## treatment25:pair7
## treatment26:pair7
## treatment21:pair8
## treatment22:pair8
## treatment23:pair8
## treatment24:pair8
## treatment25:pair8
## treatment26:pair8
## treatment21:pair9
## treatment22:pair9
## treatment23:pair9
## treatment24:pair9
## treatment25:pair9
```

```
## treatment26:pair9
## treatment21:pair10
## treatment22:pair10
## treatment23:pair10
## treatment24:pair10
## treatment25:pair10  0.500
## treatment26:pair10  0.500  0.500
## treatment21:pair11  0.250  0.250  0.250
## treatment22:pair11  0.250  0.250  0.250  0.500
## treatment23:pair11  0.250  0.250  0.250  0.500  0.500
## treatment24:pair11  0.500  0.250  0.250  0.500  0.500  0.500
## treatment25:pair11  0.250  0.500  0.250  0.500  0.500  0.500  0.500
## treatment26:pair11  0.250  0.250  0.500  0.500  0.500  0.500  0.500  0.500
## treatment21:pair12  0.250  0.250  0.250  0.500  0.250  0.250  0.250  0.250
## treatment22:pair12  0.250  0.250  0.250  0.250  0.500  0.250  0.250  0.250
## treatment23:pair12  0.250  0.250  0.250  0.250  0.250  0.500  0.250  0.250
## treatment24:pair12  0.500  0.250  0.250  0.250  0.250  0.250  0.500  0.250
## treatment25:pair12  0.250  0.500  0.250  0.250  0.250  0.250  0.250  0.500
## treatment26:pair12  0.250  0.250  0.500  0.250  0.250  0.250  0.250  0.250
## treatment21:pair13  0.250  0.250  0.250  0.500  0.250  0.250  0.250  0.250
## treatment22:pair13  0.250  0.250  0.250  0.250  0.500  0.250  0.250  0.250
## treatment23:pair13  0.250  0.250  0.250  0.250  0.250  0.500  0.250  0.250
## treatment24:pair13  0.500  0.250  0.250  0.250  0.250  0.250  0.500  0.250
## treatment25:pair13  0.250  0.500  0.250  0.250  0.250  0.250  0.250  0.500
## treatment26:pair13  0.250  0.250  0.500  0.250  0.250  0.250  0.250  0.250
## treatment21:pair14  0.250  0.250  0.250  0.500  0.250  0.250  0.250  0.250
## treatment22:pair14  0.250  0.250  0.250  0.250  0.500  0.250  0.250  0.250
## treatment23:pair14  0.250  0.250  0.250  0.250  0.250  0.500  0.250  0.250
## treatment24:pair14  0.500  0.250  0.250  0.250  0.250  0.250  0.500  0.250
## treatment25:pair14  0.250  0.500  0.250  0.250  0.250  0.250  0.250  0.500
## treatment26:pair14  0.250  0.250  0.500  0.250  0.250  0.250  0.250  0.250
## treatment21:pair15  0.250  0.250  0.250  0.500  0.250  0.250  0.250  0.250
## treatment22:pair15  0.250  0.250  0.250  0.250  0.500  0.250  0.250  0.250
## treatment23:pair15  0.250  0.250  0.250  0.250  0.250  0.500  0.250  0.250
## treatment24:pair15  0.500  0.250  0.250  0.250  0.250  0.250  0.500  0.250
## treatment25:pair15  0.250  0.500  0.250  0.250  0.250  0.250  0.250  0.500
## treatment26:pair15  0.250  0.250  0.500  0.250  0.250  0.250  0.250  0.250
##                    t26:11 t21:12 t22:12 t23:12 t24:12 t25:12 t26:12 t21:13
## treatment21
## treatment22
## treatment23
## treatment24
## treatment25
## treatment26
## pair2
## pair3
## pair4
## pair5
## pair6
## pair7
## pair8
## pair9
## pair10
## pair11
```

```
## pair12
## pair13
## pair14
## pair15
## treatment21:pair2
## treatment22:pair2
## treatment23:pair2
## treatment24:pair2
## treatment25:pair2
## treatment26:pair2
## treatment21:pair3
## treatment22:pair3
## treatment23:pair3
## treatment24:pair3
## treatment25:pair3
## treatment26:pair3
## treatment21:pair4
## treatment22:pair4
## treatment23:pair4
## treatment24:pair4
## treatment25:pair4
## treatment26:pair4
## treatment21:pair5
## treatment22:pair5
## treatment23:pair5
## treatment24:pair5
## treatment25:pair5
## treatment26:pair5
## treatment21:pair6
## treatment22:pair6
## treatment23:pair6
## treatment24:pair6
## treatment25:pair6
## treatment26:pair6
## treatment21:pair7
## treatment22:pair7
## treatment23:pair7
## treatment24:pair7
## treatment25:pair7
## treatment26:pair7
## treatment21:pair8
## treatment22:pair8
## treatment23:pair8
## treatment24:pair8
## treatment25:pair8
## treatment26:pair8
## treatment21:pair9
## treatment22:pair9
## treatment23:pair9
## treatment24:pair9
## treatment25:pair9
## treatment26:pair9
## treatment21:pair10
## treatment22:pair10
```

```
## treatment23:pair10
## treatment24:pair10
## treatment25:pair10
## treatment26:pair10
## treatment21:pair11
## treatment22:pair11
## treatment23:pair11
## treatment24:pair11
## treatment25:pair11
## treatment26:pair11
## treatment21:pair12  0.250
## treatment22:pair12  0.250  0.500
## treatment23:pair12  0.250  0.500  0.500
## treatment24:pair12  0.250  0.500  0.500  0.500
## treatment25:pair12  0.250  0.500  0.500  0.500  0.500
## treatment26:pair12  0.500  0.500  0.500  0.500  0.500  0.500
## treatment21:pair13  0.250  0.500  0.250  0.250  0.250  0.250  0.250
## treatment22:pair13  0.250  0.250  0.500  0.250  0.250  0.250  0.250  0.500
## treatment23:pair13  0.250  0.250  0.250  0.500  0.250  0.250  0.250  0.500
## treatment24:pair13  0.250  0.250  0.250  0.250  0.500  0.250  0.250  0.500
## treatment25:pair13  0.250  0.250  0.250  0.250  0.250  0.500  0.250  0.500
## treatment26:pair13  0.500  0.250  0.250  0.250  0.250  0.250  0.500  0.500
## treatment21:pair14  0.250  0.500  0.250  0.250  0.250  0.250  0.250  0.500
## treatment22:pair14  0.250  0.250  0.500  0.250  0.250  0.250  0.250  0.250
## treatment23:pair14  0.250  0.250  0.250  0.500  0.250  0.250  0.250  0.250
## treatment24:pair14  0.250  0.250  0.250  0.250  0.500  0.250  0.250  0.250
## treatment25:pair14  0.250  0.250  0.250  0.250  0.250  0.500  0.250  0.250
## treatment26:pair14  0.500  0.250  0.250  0.250  0.250  0.250  0.500  0.250
## treatment21:pair15  0.250  0.500  0.250  0.250  0.250  0.250  0.250  0.500
## treatment22:pair15  0.250  0.250  0.500  0.250  0.250  0.250  0.250  0.250
## treatment23:pair15  0.250  0.250  0.250  0.500  0.250  0.250  0.250  0.250
## treatment24:pair15  0.250  0.250  0.250  0.250  0.500  0.250  0.250  0.250
## treatment25:pair15  0.250  0.250  0.250  0.250  0.250  0.500  0.250  0.250
## treatment26:pair15  0.500  0.250  0.250  0.250  0.250  0.250  0.500  0.250
##                    t22:13 t23:13 t24:13 t25:13 t26:13 t21:14 t22:14 t23:14
## treatment21
## treatment22
## treatment23
## treatment24
## treatment25
## treatment26
## pair2
## pair3
## pair4
## pair5
## pair6
## pair7
## pair8
## pair9
## pair10
## pair11
## pair12
## pair13
## pair14
```

```
## pair15
## treatment21:pair2
## treatment22:pair2
## treatment23:pair2
## treatment24:pair2
## treatment25:pair2
## treatment26:pair2
## treatment21:pair3
## treatment22:pair3
## treatment23:pair3
## treatment24:pair3
## treatment25:pair3
## treatment26:pair3
## treatment21:pair4
## treatment22:pair4
## treatment23:pair4
## treatment24:pair4
## treatment25:pair4
## treatment26:pair4
## treatment21:pair5
## treatment22:pair5
## treatment23:pair5
## treatment24:pair5
## treatment25:pair5
## treatment26:pair5
## treatment21:pair6
## treatment22:pair6
## treatment23:pair6
## treatment24:pair6
## treatment25:pair6
## treatment26:pair6
## treatment21:pair7
## treatment22:pair7
## treatment23:pair7
## treatment24:pair7
## treatment25:pair7
## treatment26:pair7
## treatment21:pair8
## treatment22:pair8
## treatment23:pair8
## treatment24:pair8
## treatment25:pair8
## treatment26:pair8
## treatment21:pair9
## treatment22:pair9
## treatment23:pair9
## treatment24:pair9
## treatment25:pair9
## treatment26:pair9
## treatment21:pair10
## treatment22:pair10
## treatment23:pair10
## treatment24:pair10
## treatment25:pair10
```

```
## treatment26:pair10
## treatment21:pair11
## treatment22:pair11
## treatment23:pair11
## treatment24:pair11
## treatment25:pair11
## treatment26:pair11
## treatment21:pair12
## treatment22:pair12
## treatment23:pair12
## treatment24:pair12
## treatment25:pair12
## treatment26:pair12
## treatment21:pair13
## treatment22:pair13
## treatment23:pair13  0.500
## treatment24:pair13  0.500  0.500
## treatment25:pair13  0.500  0.500  0.500
## treatment26:pair13  0.500  0.500  0.500  0.500
## treatment21:pair14  0.250  0.250  0.250  0.250  0.250
## treatment22:pair14  0.500  0.250  0.250  0.250  0.250  0.500
## treatment23:pair14  0.250  0.500  0.250  0.250  0.250  0.500  0.500
## treatment24:pair14  0.250  0.250  0.500  0.250  0.250  0.500  0.500  0.500
## treatment25:pair14  0.250  0.250  0.250  0.500  0.250  0.500  0.500  0.500
## treatment26:pair14  0.250  0.250  0.250  0.250  0.500  0.500  0.500  0.500
## treatment21:pair15  0.250  0.250  0.250  0.250  0.250  0.500  0.250  0.250
## treatment22:pair15  0.500  0.250  0.250  0.250  0.250  0.250  0.500  0.250
## treatment23:pair15  0.250  0.500  0.250  0.250  0.250  0.250  0.250  0.500
## treatment24:pair15  0.250  0.250  0.500  0.250  0.250  0.250  0.250  0.250
## treatment25:pair15  0.250  0.250  0.250  0.500  0.250  0.250  0.250  0.250
## treatment26:pair15  0.250  0.250  0.250  0.250  0.500  0.250  0.250  0.250
##                    t24:14 t25:14 t26:14 t21:15 t22:15 t23:15 t24:15 t25:15
## treatment21
## treatment22
## treatment23
## treatment24
## treatment25
## treatment26
## pair2
## pair3
## pair4
## pair5
## pair6
## pair7
## pair8
## pair9
## pair10
## pair11
## pair12
## pair13
## pair14
## pair15
## treatment21:pair2
## treatment22:pair2
```

```
## treatment23:pair2
## treatment24:pair2
## treatment25:pair2
## treatment26:pair2
## treatment21:pair3
## treatment22:pair3
## treatment23:pair3
## treatment24:pair3
## treatment25:pair3
## treatment26:pair3
## treatment21:pair4
## treatment22:pair4
## treatment23:pair4
## treatment24:pair4
## treatment25:pair4
## treatment26:pair4
## treatment21:pair5
## treatment22:pair5
## treatment23:pair5
## treatment24:pair5
## treatment25:pair5
## treatment26:pair5
## treatment21:pair6
## treatment22:pair6
## treatment23:pair6
## treatment24:pair6
## treatment25:pair6
## treatment26:pair6
## treatment21:pair7
## treatment22:pair7
## treatment23:pair7
## treatment24:pair7
## treatment25:pair7
## treatment26:pair7
## treatment21:pair8
## treatment22:pair8
## treatment23:pair8
## treatment24:pair8
## treatment25:pair8
## treatment26:pair8
## treatment21:pair9
## treatment22:pair9
## treatment23:pair9
## treatment24:pair9
## treatment25:pair9
## treatment26:pair9
## treatment21:pair10
## treatment22:pair10
## treatment23:pair10
## treatment24:pair10
## treatment25:pair10
## treatment26:pair10
## treatment21:pair11
## treatment22:pair11
```

```
## treatment23:pair11
## treatment24:pair11
## treatment25:pair11
## treatment26:pair11
## treatment21:pair12
## treatment22:pair12
## treatment23:pair12
## treatment24:pair12
## treatment25:pair12
## treatment26:pair12
## treatment21:pair13
## treatment22:pair13
## treatment23:pair13
## treatment24:pair13
## treatment25:pair13
## treatment26:pair13
## treatment21:pair14
## treatment22:pair14
## treatment23:pair14
## treatment24:pair14
## treatment25:pair14  0.500
## treatment26:pair14  0.500  0.500
## treatment21:pair15  0.250  0.250  0.250
## treatment22:pair15  0.250  0.250  0.250  0.500
## treatment23:pair15  0.250  0.250  0.250  0.500  0.500
## treatment24:pair15  0.500  0.250  0.250  0.500  0.500  0.500
## treatment25:pair15  0.250  0.500  0.250  0.500  0.500  0.500  0.500
## treatment26:pair15  0.250  0.250  0.500  0.500  0.500  0.500  0.500  0.500
##
## Standardized Within-Group Residuals:
##         Min          Q1         Med          Q3         Max
## -2.46091645 -0.47371752 -0.02213161  0.45482369  4.22699915
##
## Number of Observations: 315
## Number of Groups: 21
```

2931s

```
data<-read.table("~/Desktop/rsync/deteriorating treatments/salt/2931_salt_v2_[0].csv",h=T)
data<-subset(data,treatment<7)
data$pair<-as.factor(data$pair)
data$treatment2<-as.factor(data$treatment)
fit2<-lme(relfitav~treatment2*pair,random=~1|plate,data)
anova(fit2)
```

```
##                 numDF denDF  F-value p-value
## (Intercept)         1   182 850.9935  <.0001
## treatment2          6    14   0.7586  0.6137
## pair               13   182   6.9103  <.0001
## treatment2:pair    78   182   0.9207  0.6564
```

```r
summary(fit2)
```

```
## Linear mixed-effects model fit by REML
##  Data: data
##        AIC      BIC    logLik
##   329.8047 657.6161 -64.90233
##
## Random effects:
##  Formula: ~1 | plate
##         (Intercept)  Residual
## StdDev:   0.1033695 0.2448395
##
## Fixed effects: relfitav ~ treatment2 * pair
##                         Value Std.Error  DF   t-value p-value
## (Intercept)         0.9843263 0.1534401 182  6.415051  0.0000
## treatment21         0.0348075 0.2169971  14  0.160405  0.8749
## treatment22        -0.1526830 0.2169971  14 -0.703618  0.4932
## treatment23        -0.4062208 0.2169971  14 -1.872010  0.0822
## treatment24        -0.2116590 0.2169971  14 -0.975400  0.3459
## treatment25        -0.1697998 0.2169971  14 -0.782498  0.4470
## treatment26        -0.4718197 0.2169971  14 -2.174313  0.0473
## pair2              -0.2094629 0.1999106 182 -1.047783  0.2961
## pair3              -0.1197203 0.1999106 182 -0.598869  0.5500
## pair4              -0.0083854 0.1999106 182 -0.041946  0.9666
## pair5              -0.3231531 0.1999106 182 -1.616488  0.1077
## pair6              -0.1419631 0.1999106 182 -0.710133  0.4785
## pair7              -0.1654608 0.1999106 182 -0.827674  0.4089
## pair9              -0.2990419 0.1999106 182 -1.495878  0.1364
## pair10             -0.0175116 0.1999106 182 -0.087597  0.9303
## pair11             -0.2285862 0.1999106 182 -1.143442  0.2544
## pair12             -0.4839330 0.1999106 182 -2.420747  0.0165
## pair13             -0.1541423 0.1999106 182 -0.771056  0.4417
## pair14             -0.0969229 0.1999106 182 -0.484831  0.6284
## pair15             -0.1205426 0.1999106 182 -0.602983  0.5473
## treatment21:pair2  -0.3136128 0.2827163 182 -1.109284  0.2688
## treatment22:pair2   0.2089984 0.2827163 182  0.739251  0.4607
## treatment23:pair2   0.1503423 0.2827163 182  0.531778  0.5955
## treatment24:pair2  -0.1314992 0.2827163 182 -0.465128  0.6424
## treatment25:pair2  -0.2982107 0.2827163 182 -1.054806  0.2929
## treatment26:pair2   0.0781104 0.2827163 182  0.276286  0.7826
## treatment21:pair3  -0.0587808 0.2827163 182 -0.207915  0.8355
## treatment22:pair3   0.1883045 0.2827163 182  0.666055  0.5062
## treatment23:pair3   0.3273346 0.2827163 182  1.157820  0.2485
## treatment24:pair3   0.1331775 0.2827163 182  0.471064  0.6382
## treatment25:pair3   0.1051572 0.2827163 182  0.371953  0.7104
## treatment26:pair3   0.3502911 0.2827163 182  1.239020  0.2169
## treatment21:pair4  -0.2587570 0.2827163 182 -0.915253  0.3613
## treatment22:pair4  -0.1859178 0.2827163 182 -0.657612  0.5116
## treatment23:pair4  -0.0088950 0.2827163 182 -0.031463  0.9749
## treatment24:pair4  -0.0000969 0.2827163 182 -0.000343  0.9997
## treatment25:pair4   0.0996231 0.2827163 182  0.352378  0.7250
## treatment26:pair4  -0.1039735 0.2827163 182 -0.367766  0.7135
## treatment21:pair5   0.0719050 0.2827163 182  0.254336  0.7995
```

```
## treatment22:pair5   0.1939735 0.2827163 182  0.686106  0.4935
## treatment23:pair5   0.5266328 0.2827163 182  1.862761  0.0641
## treatment24:pair5   0.2173822 0.2827163 182  0.768906  0.4429
## treatment25:pair5   0.1838921 0.2827163 182  0.650447  0.5162
## treatment26:pair5   0.5988670 0.2827163 182  2.118261  0.0355
## treatment21:pair6  -0.1159927 0.2827163 182 -0.410280  0.6821
## treatment22:pair6   0.0840581 0.2827163 182  0.297323  0.7666
## treatment23:pair6   0.3186827 0.2827163 182  1.127217  0.2611
## treatment24:pair6   0.0909071 0.2827163 182  0.321549  0.7482
## treatment25:pair6  -0.1086985 0.2827163 182 -0.384479  0.7011
## treatment26:pair6   0.3711524 0.2827163 182  1.312809  0.1909
## treatment21:pair7   0.1795150 0.2827163 182  0.634965  0.5262
## treatment22:pair7   0.0876741 0.2827163 182  0.310113  0.7568
## treatment23:pair7   0.6316018 0.2827163 182  2.234048  0.0267
## treatment24:pair7   0.3475840 0.2827163 182  1.229445  0.2205
## treatment25:pair7   0.1791485 0.2827163 182  0.633669  0.5271
## treatment26:pair7   0.3619500 0.2827163 182  1.280259  0.2021
## treatment21:pair9   0.0220693 0.2827163 182  0.078062  0.9379
## treatment22:pair9   0.3014876 0.2827163 182  1.066396  0.2877
## treatment23:pair9   0.2447006 0.2827163 182  0.865534  0.3879
## treatment24:pair9   0.3701160 0.2827163 182  1.309143  0.1921
## treatment25:pair9   0.2023623 0.2827163 182  0.715779  0.4750
## treatment26:pair9   0.3655324 0.2827163 182  1.292930  0.1977
## treatment21:pair10  0.1591863 0.2827163 182  0.563060  0.5741
## treatment22:pair10  0.1644793 0.2827163 182  0.581782  0.5614
## treatment23:pair10  0.5684193 0.2827163 182  2.010564  0.0458
## treatment24:pair10  0.1352733 0.2827163 182  0.478477  0.6329
## treatment25:pair10  0.3361576 0.2827163 182  1.189028  0.2360
## treatment26:pair10  0.2252919 0.2827163 182  0.796883  0.4266
## treatment21:pair11 -0.0036066 0.2827163 182 -0.012757  0.9898
## treatment22:pair11  0.2166236 0.2827163 182  0.766222  0.4445
## treatment23:pair11  0.6611971 0.2827163 182  2.338730  0.0204
## treatment24:pair11  0.3932481 0.2827163 182  1.390964  0.1659
## treatment25:pair11  0.1843462 0.2827163 182  0.652054  0.5152
## treatment26:pair11  0.5472580 0.2827163 182  1.935714  0.0545
## treatment21:pair12  0.0397735 0.2827163 182  0.140683  0.8883
## treatment22:pair12  0.1462823 0.2827163 182  0.517417  0.6055
## treatment23:pair12  0.4913011 0.2827163 182  1.737788  0.0839
## treatment24:pair12  0.2945621 0.2827163 182  1.041900  0.2988
## treatment25:pair12  0.1864644 0.2827163 182  0.659546  0.5104
## treatment26:pair12  0.2782792 0.2827163 182  0.984306  0.3263
## treatment21:pair13  0.1432800 0.2827163 182  0.506798  0.6129
## treatment22:pair13  0.1686408 0.2827163 182  0.596502  0.5516
## treatment23:pair13  0.2991636 0.2827163 182  1.058176  0.2914
## treatment24:pair13  0.0267438 0.2827163 182  0.094596  0.9247
## treatment25:pair13  0.0240053 0.2827163 182  0.084909  0.9324
## treatment26:pair13  0.7120027 0.2827163 182  2.518435  0.0126
## treatment21:pair14  0.1246284 0.2827163 182  0.440825  0.6599
## treatment22:pair14  0.1083649 0.2827163 182  0.383299  0.7019
## treatment23:pair14  0.5320436 0.2827163 182  1.881899  0.0614
## treatment24:pair14  0.2106276 0.2827163 182  0.745014  0.4572
## treatment25:pair14  0.2558497 0.2827163 182  0.904970  0.3667
## treatment26:pair14 -0.0237869 0.2827163 182 -0.084137  0.9330
## treatment21:pair15  0.0130801 0.2827163 182  0.046266  0.9631
```

```
## treatment22:pair15  0.1636055 0.2827163 182  0.578691  0.5635
## treatment23:pair15  0.4578411 0.2827163 182  1.619437  0.1071
## treatment24:pair15  0.5368994 0.2827163 182  1.899075  0.0591
## treatment25:pair15  0.3601091 0.2827163 182  1.273747  0.2044
## treatment26:pair15  0.5771622 0.2827163 182  2.041489  0.0426
##  Correlation:
##                     (Intr) trtm21 trtm22 trtm23 trtm24 trtm25 trtm26 pair2
## treatment21         -0.707
## treatment22         -0.707  0.500
## treatment23         -0.707  0.500  0.500
## treatment24         -0.707  0.500  0.500  0.500
## treatment25         -0.707  0.500  0.500  0.500  0.500
## treatment26         -0.707  0.500  0.500  0.500  0.500  0.500
## pair2               -0.651  0.461  0.461  0.461  0.461  0.461  0.461
## pair3               -0.651  0.461  0.461  0.461  0.461  0.461  0.461  0.500
## pair4               -0.651  0.461  0.461  0.461  0.461  0.461  0.461  0.500
## pair5               -0.651  0.461  0.461  0.461  0.461  0.461  0.461  0.500
## pair6               -0.651  0.461  0.461  0.461  0.461  0.461  0.461  0.500
## pair7               -0.651  0.461  0.461  0.461  0.461  0.461  0.461  0.500
## pair9               -0.651  0.461  0.461  0.461  0.461  0.461  0.461  0.500
## pair10              -0.651  0.461  0.461  0.461  0.461  0.461  0.461  0.500
## pair11              -0.651  0.461  0.461  0.461  0.461  0.461  0.461  0.500
## pair12              -0.651  0.461  0.461  0.461  0.461  0.461  0.461  0.500
## pair13              -0.651  0.461  0.461  0.461  0.461  0.461  0.461  0.500
## pair14              -0.651  0.461  0.461  0.461  0.461  0.461  0.461  0.500
## pair15              -0.651  0.461  0.461  0.461  0.461  0.461  0.461  0.500
## treatment21:pair2    0.461 -0.651 -0.326 -0.326 -0.326 -0.326 -0.326 -0.707
## treatment22:pair2    0.461 -0.326 -0.651 -0.326 -0.326 -0.326 -0.326 -0.707
## treatment23:pair2    0.461 -0.326 -0.326 -0.651 -0.326 -0.326 -0.326 -0.707
## treatment24:pair2    0.461 -0.326 -0.326 -0.326 -0.651 -0.326 -0.326 -0.707
## treatment25:pair2    0.461 -0.326 -0.326 -0.326 -0.326 -0.651 -0.326 -0.707
## treatment26:pair2    0.461 -0.326 -0.326 -0.326 -0.326 -0.326 -0.651 -0.707
## treatment21:pair3    0.461 -0.651 -0.326 -0.326 -0.326 -0.326 -0.326 -0.354
## treatment22:pair3    0.461 -0.326 -0.651 -0.326 -0.326 -0.326 -0.326 -0.354
## treatment23:pair3    0.461 -0.326 -0.326 -0.651 -0.326 -0.326 -0.326 -0.354
## treatment24:pair3    0.461 -0.326 -0.326 -0.326 -0.651 -0.326 -0.326 -0.354
## treatment25:pair3    0.461 -0.326 -0.326 -0.326 -0.326 -0.651 -0.326 -0.354
## treatment26:pair3    0.461 -0.326 -0.326 -0.326 -0.326 -0.326 -0.651 -0.354
## treatment21:pair4    0.461 -0.651 -0.326 -0.326 -0.326 -0.326 -0.326 -0.354
## treatment22:pair4    0.461 -0.326 -0.651 -0.326 -0.326 -0.326 -0.326 -0.354
## treatment23:pair4    0.461 -0.326 -0.326 -0.651 -0.326 -0.326 -0.326 -0.354
## treatment24:pair4    0.461 -0.326 -0.326 -0.326 -0.651 -0.326 -0.326 -0.354
## treatment25:pair4    0.461 -0.326 -0.326 -0.326 -0.326 -0.651 -0.326 -0.354
## treatment26:pair4    0.461 -0.326 -0.326 -0.326 -0.326 -0.326 -0.651 -0.354
## treatment21:pair5    0.461 -0.651 -0.326 -0.326 -0.326 -0.326 -0.326 -0.354
## treatment22:pair5    0.461 -0.326 -0.651 -0.326 -0.326 -0.326 -0.326 -0.354
## treatment23:pair5    0.461 -0.326 -0.326 -0.651 -0.326 -0.326 -0.326 -0.354
## treatment24:pair5    0.461 -0.326 -0.326 -0.326 -0.651 -0.326 -0.326 -0.354
## treatment25:pair5    0.461 -0.326 -0.326 -0.326 -0.326 -0.651 -0.326 -0.354
## treatment26:pair5    0.461 -0.326 -0.326 -0.326 -0.326 -0.326 -0.651 -0.354
## treatment21:pair6    0.461 -0.651 -0.326 -0.326 -0.326 -0.326 -0.326 -0.354
## treatment22:pair6    0.461 -0.326 -0.651 -0.326 -0.326 -0.326 -0.326 -0.354
## treatment23:pair6    0.461 -0.326 -0.326 -0.651 -0.326 -0.326 -0.326 -0.354
## treatment24:pair6    0.461 -0.326 -0.326 -0.326 -0.651 -0.326 -0.326 -0.354
```

```
## treatment25:pair6   0.461 -0.326 -0.326 -0.326 -0.326 -0.651 -0.326 -0.354
## treatment26:pair6   0.461 -0.326 -0.326 -0.326 -0.326 -0.326 -0.651 -0.354
## treatment21:pair7   0.461 -0.651 -0.326 -0.326 -0.326 -0.326 -0.326 -0.354
## treatment22:pair7   0.461 -0.326 -0.651 -0.326 -0.326 -0.326 -0.326 -0.354
## treatment23:pair7   0.461 -0.326 -0.326 -0.651 -0.326 -0.326 -0.326 -0.354
## treatment24:pair7   0.461 -0.326 -0.326 -0.326 -0.651 -0.326 -0.326 -0.354
## treatment25:pair7   0.461 -0.326 -0.326 -0.326 -0.326 -0.651 -0.326 -0.354
## treatment26:pair7   0.461 -0.326 -0.326 -0.326 -0.326 -0.326 -0.651 -0.354
## treatment21:pair9   0.461 -0.651 -0.326 -0.326 -0.326 -0.326 -0.326 -0.354
## treatment22:pair9   0.461 -0.326 -0.651 -0.326 -0.326 -0.326 -0.326 -0.354
## treatment23:pair9   0.461 -0.326 -0.326 -0.651 -0.326 -0.326 -0.326 -0.354
## treatment24:pair9   0.461 -0.326 -0.326 -0.326 -0.651 -0.326 -0.326 -0.354
## treatment25:pair9   0.461 -0.326 -0.326 -0.326 -0.326 -0.651 -0.326 -0.354
## treatment26:pair9   0.461 -0.326 -0.326 -0.326 -0.326 -0.326 -0.651 -0.354
## treatment21:pair10  0.461 -0.651 -0.326 -0.326 -0.326 -0.326 -0.326 -0.354
## treatment22:pair10  0.461 -0.326 -0.651 -0.326 -0.326 -0.326 -0.326 -0.354
## treatment23:pair10  0.461 -0.326 -0.326 -0.651 -0.326 -0.326 -0.326 -0.354
## treatment24:pair10  0.461 -0.326 -0.326 -0.326 -0.651 -0.326 -0.326 -0.354
## treatment25:pair10  0.461 -0.326 -0.326 -0.326 -0.326 -0.651 -0.326 -0.354
## treatment26:pair10  0.461 -0.326 -0.326 -0.326 -0.326 -0.326 -0.651 -0.354
## treatment21:pair11  0.461 -0.651 -0.326 -0.326 -0.326 -0.326 -0.326 -0.354
## treatment22:pair11  0.461 -0.326 -0.651 -0.326 -0.326 -0.326 -0.326 -0.354
## treatment23:pair11  0.461 -0.326 -0.326 -0.651 -0.326 -0.326 -0.326 -0.354
## treatment24:pair11  0.461 -0.326 -0.326 -0.326 -0.651 -0.326 -0.326 -0.354
## treatment25:pair11  0.461 -0.326 -0.326 -0.326 -0.326 -0.651 -0.326 -0.354
## treatment26:pair11  0.461 -0.326 -0.326 -0.326 -0.326 -0.326 -0.651 -0.354
## treatment21:pair12  0.461 -0.651 -0.326 -0.326 -0.326 -0.326 -0.326 -0.354
## treatment22:pair12  0.461 -0.326 -0.651 -0.326 -0.326 -0.326 -0.326 -0.354
## treatment23:pair12  0.461 -0.326 -0.326 -0.651 -0.326 -0.326 -0.326 -0.354
## treatment24:pair12  0.461 -0.326 -0.326 -0.326 -0.651 -0.326 -0.326 -0.354
## treatment25:pair12  0.461 -0.326 -0.326 -0.326 -0.326 -0.651 -0.326 -0.354
## treatment26:pair12  0.461 -0.326 -0.326 -0.326 -0.326 -0.326 -0.651 -0.354
## treatment21:pair13  0.461 -0.651 -0.326 -0.326 -0.326 -0.326 -0.326 -0.354
## treatment22:pair13  0.461 -0.326 -0.651 -0.326 -0.326 -0.326 -0.326 -0.354
## treatment23:pair13  0.461 -0.326 -0.326 -0.651 -0.326 -0.326 -0.326 -0.354
## treatment24:pair13  0.461 -0.326 -0.326 -0.326 -0.651 -0.326 -0.326 -0.354
## treatment25:pair13  0.461 -0.326 -0.326 -0.326 -0.326 -0.651 -0.326 -0.354
## treatment26:pair13  0.461 -0.326 -0.326 -0.326 -0.326 -0.326 -0.651 -0.354
## treatment21:pair14  0.461 -0.651 -0.326 -0.326 -0.326 -0.326 -0.326 -0.354
## treatment22:pair14  0.461 -0.326 -0.651 -0.326 -0.326 -0.326 -0.326 -0.354
## treatment23:pair14  0.461 -0.326 -0.326 -0.651 -0.326 -0.326 -0.326 -0.354
## treatment24:pair14  0.461 -0.326 -0.326 -0.326 -0.651 -0.326 -0.326 -0.354
## treatment25:pair14  0.461 -0.326 -0.326 -0.326 -0.326 -0.651 -0.326 -0.354
## treatment26:pair14  0.461 -0.326 -0.326 -0.326 -0.326 -0.326 -0.651 -0.354
## treatment21:pair15  0.461 -0.651 -0.326 -0.326 -0.326 -0.326 -0.326 -0.354
## treatment22:pair15  0.461 -0.326 -0.651 -0.326 -0.326 -0.326 -0.326 -0.354
## treatment23:pair15  0.461 -0.326 -0.326 -0.651 -0.326 -0.326 -0.326 -0.354
## treatment24:pair15  0.461 -0.326 -0.326 -0.326 -0.651 -0.326 -0.326 -0.354
## treatment25:pair15  0.461 -0.326 -0.326 -0.326 -0.326 -0.651 -0.326 -0.354
## treatment26:pair15  0.461 -0.326 -0.326 -0.326 -0.326 -0.326 -0.651 -0.354
##                    pair3  pair4  pair5  pair6  pair7  pair9  pair10 pair11
## treatment21
## treatment22
## treatment23
```

```
## treatment24
## treatment25
## treatment26
## pair2
## pair3
## pair4               0.500
## pair5               0.500  0.500
## pair6               0.500  0.500  0.500
## pair7               0.500  0.500  0.500  0.500
## pair9               0.500  0.500  0.500  0.500  0.500
## pair10              0.500  0.500  0.500  0.500  0.500  0.500
## pair11              0.500  0.500  0.500  0.500  0.500  0.500  0.500
## pair12              0.500  0.500  0.500  0.500  0.500  0.500  0.500  0.500
## pair13              0.500  0.500  0.500  0.500  0.500  0.500  0.500  0.500
## pair14              0.500  0.500  0.500  0.500  0.500  0.500  0.500  0.500
## pair15              0.500  0.500  0.500  0.500  0.500  0.500  0.500  0.500
## treatment21:pair2  -0.354 -0.354 -0.354 -0.354 -0.354 -0.354 -0.354 -0.354
## treatment22:pair2  -0.354 -0.354 -0.354 -0.354 -0.354 -0.354 -0.354 -0.354
## treatment23:pair2  -0.354 -0.354 -0.354 -0.354 -0.354 -0.354 -0.354 -0.354
## treatment24:pair2  -0.354 -0.354 -0.354 -0.354 -0.354 -0.354 -0.354 -0.354
## treatment25:pair2  -0.354 -0.354 -0.354 -0.354 -0.354 -0.354 -0.354 -0.354
## treatment26:pair2  -0.354 -0.354 -0.354 -0.354 -0.354 -0.354 -0.354 -0.354
## treatment21:pair3  -0.707 -0.354 -0.354 -0.354 -0.354 -0.354 -0.354 -0.354
## treatment22:pair3  -0.707 -0.354 -0.354 -0.354 -0.354 -0.354 -0.354 -0.354
## treatment23:pair3  -0.707 -0.354 -0.354 -0.354 -0.354 -0.354 -0.354 -0.354
## treatment24:pair3  -0.707 -0.354 -0.354 -0.354 -0.354 -0.354 -0.354 -0.354
## treatment25:pair3  -0.707 -0.354 -0.354 -0.354 -0.354 -0.354 -0.354 -0.354
## treatment26:pair3  -0.707 -0.354 -0.354 -0.354 -0.354 -0.354 -0.354 -0.354
## treatment21:pair4  -0.354 -0.707 -0.354 -0.354 -0.354 -0.354 -0.354 -0.354
## treatment22:pair4  -0.354 -0.707 -0.354 -0.354 -0.354 -0.354 -0.354 -0.354
## treatment23:pair4  -0.354 -0.707 -0.354 -0.354 -0.354 -0.354 -0.354 -0.354
## treatment24:pair4  -0.354 -0.707 -0.354 -0.354 -0.354 -0.354 -0.354 -0.354
## treatment25:pair4  -0.354 -0.707 -0.354 -0.354 -0.354 -0.354 -0.354 -0.354
## treatment26:pair4  -0.354 -0.707 -0.354 -0.354 -0.354 -0.354 -0.354 -0.354
## treatment21:pair5  -0.354 -0.354 -0.707 -0.354 -0.354 -0.354 -0.354 -0.354
## treatment22:pair5  -0.354 -0.354 -0.707 -0.354 -0.354 -0.354 -0.354 -0.354
## treatment23:pair5  -0.354 -0.354 -0.707 -0.354 -0.354 -0.354 -0.354 -0.354
## treatment24:pair5  -0.354 -0.354 -0.707 -0.354 -0.354 -0.354 -0.354 -0.354
## treatment25:pair5  -0.354 -0.354 -0.707 -0.354 -0.354 -0.354 -0.354 -0.354
## treatment26:pair5  -0.354 -0.354 -0.707 -0.354 -0.354 -0.354 -0.354 -0.354
## treatment21:pair6  -0.354 -0.354 -0.354 -0.707 -0.354 -0.354 -0.354 -0.354
## treatment22:pair6  -0.354 -0.354 -0.354 -0.707 -0.354 -0.354 -0.354 -0.354
## treatment23:pair6  -0.354 -0.354 -0.354 -0.707 -0.354 -0.354 -0.354 -0.354
## treatment24:pair6  -0.354 -0.354 -0.354 -0.707 -0.354 -0.354 -0.354 -0.354
## treatment25:pair6  -0.354 -0.354 -0.354 -0.707 -0.354 -0.354 -0.354 -0.354
## treatment26:pair6  -0.354 -0.354 -0.354 -0.707 -0.354 -0.354 -0.354 -0.354
## treatment21:pair7  -0.354 -0.354 -0.354 -0.354 -0.707 -0.354 -0.354 -0.354
## treatment22:pair7  -0.354 -0.354 -0.354 -0.354 -0.707 -0.354 -0.354 -0.354
## treatment23:pair7  -0.354 -0.354 -0.354 -0.354 -0.707 -0.354 -0.354 -0.354
## treatment24:pair7  -0.354 -0.354 -0.354 -0.354 -0.707 -0.354 -0.354 -0.354
## treatment25:pair7  -0.354 -0.354 -0.354 -0.354 -0.707 -0.354 -0.354 -0.354
## treatment26:pair7  -0.354 -0.354 -0.354 -0.354 -0.707 -0.354 -0.354 -0.354
## treatment21:pair9  -0.354 -0.354 -0.354 -0.354 -0.354 -0.707 -0.354 -0.354
## treatment22:pair9  -0.354 -0.354 -0.354 -0.354 -0.354 -0.707 -0.354 -0.354
```

```
## treatment23:pair9  -0.354 -0.354 -0.354 -0.354 -0.354 -0.707 -0.354 -0.354
## treatment24:pair9  -0.354 -0.354 -0.354 -0.354 -0.354 -0.707 -0.354 -0.354
## treatment25:pair9  -0.354 -0.354 -0.354 -0.354 -0.354 -0.707 -0.354 -0.354
## treatment26:pair9  -0.354 -0.354 -0.354 -0.354 -0.354 -0.707 -0.354 -0.354
## treatment21:pair10 -0.354 -0.354 -0.354 -0.354 -0.354 -0.354 -0.707 -0.354
## treatment22:pair10 -0.354 -0.354 -0.354 -0.354 -0.354 -0.354 -0.707 -0.354
## treatment23:pair10 -0.354 -0.354 -0.354 -0.354 -0.354 -0.354 -0.707 -0.354
## treatment24:pair10 -0.354 -0.354 -0.354 -0.354 -0.354 -0.354 -0.707 -0.354
## treatment25:pair10 -0.354 -0.354 -0.354 -0.354 -0.354 -0.354 -0.707 -0.354
## treatment26:pair10 -0.354 -0.354 -0.354 -0.354 -0.354 -0.354 -0.707 -0.354
## treatment21:pair11 -0.354 -0.354 -0.354 -0.354 -0.354 -0.354 -0.354 -0.707
## treatment22:pair11 -0.354 -0.354 -0.354 -0.354 -0.354 -0.354 -0.354 -0.707
## treatment23:pair11 -0.354 -0.354 -0.354 -0.354 -0.354 -0.354 -0.354 -0.707
## treatment24:pair11 -0.354 -0.354 -0.354 -0.354 -0.354 -0.354 -0.354 -0.707
## treatment25:pair11 -0.354 -0.354 -0.354 -0.354 -0.354 -0.354 -0.354 -0.707
## treatment26:pair11 -0.354 -0.354 -0.354 -0.354 -0.354 -0.354 -0.354 -0.707
## treatment21:pair12 -0.354 -0.354 -0.354 -0.354 -0.354 -0.354 -0.354 -0.354
## treatment22:pair12 -0.354 -0.354 -0.354 -0.354 -0.354 -0.354 -0.354 -0.354
## treatment23:pair12 -0.354 -0.354 -0.354 -0.354 -0.354 -0.354 -0.354 -0.354
## treatment24:pair12 -0.354 -0.354 -0.354 -0.354 -0.354 -0.354 -0.354 -0.354
## treatment25:pair12 -0.354 -0.354 -0.354 -0.354 -0.354 -0.354 -0.354 -0.354
## treatment26:pair12 -0.354 -0.354 -0.354 -0.354 -0.354 -0.354 -0.354 -0.354
## treatment21:pair13 -0.354 -0.354 -0.354 -0.354 -0.354 -0.354 -0.354 -0.354
## treatment22:pair13 -0.354 -0.354 -0.354 -0.354 -0.354 -0.354 -0.354 -0.354
## treatment23:pair13 -0.354 -0.354 -0.354 -0.354 -0.354 -0.354 -0.354 -0.354
## treatment24:pair13 -0.354 -0.354 -0.354 -0.354 -0.354 -0.354 -0.354 -0.354
## treatment25:pair13 -0.354 -0.354 -0.354 -0.354 -0.354 -0.354 -0.354 -0.354
## treatment26:pair13 -0.354 -0.354 -0.354 -0.354 -0.354 -0.354 -0.354 -0.354
## treatment21:pair14 -0.354 -0.354 -0.354 -0.354 -0.354 -0.354 -0.354 -0.354
## treatment22:pair14 -0.354 -0.354 -0.354 -0.354 -0.354 -0.354 -0.354 -0.354
## treatment23:pair14 -0.354 -0.354 -0.354 -0.354 -0.354 -0.354 -0.354 -0.354
## treatment24:pair14 -0.354 -0.354 -0.354 -0.354 -0.354 -0.354 -0.354 -0.354
## treatment25:pair14 -0.354 -0.354 -0.354 -0.354 -0.354 -0.354 -0.354 -0.354
## treatment26:pair14 -0.354 -0.354 -0.354 -0.354 -0.354 -0.354 -0.354 -0.354
## treatment21:pair15 -0.354 -0.354 -0.354 -0.354 -0.354 -0.354 -0.354 -0.354
## treatment22:pair15 -0.354 -0.354 -0.354 -0.354 -0.354 -0.354 -0.354 -0.354
## treatment23:pair15 -0.354 -0.354 -0.354 -0.354 -0.354 -0.354 -0.354 -0.354
## treatment24:pair15 -0.354 -0.354 -0.354 -0.354 -0.354 -0.354 -0.354 -0.354
## treatment25:pair15 -0.354 -0.354 -0.354 -0.354 -0.354 -0.354 -0.354 -0.354
## treatment26:pair15 -0.354 -0.354 -0.354 -0.354 -0.354 -0.354 -0.354 -0.354
##                    pair12 pair13 pair14 pair15 tr21:2 tr22:2 tr23:2 tr24:2
## treatment21
## treatment22
## treatment23
## treatment24
## treatment25
## treatment26
## pair2
## pair3
## pair4
## pair5
## pair6
## pair7
## pair9
```

```
## pair10
## pair11
## pair12
## pair13              0.500
## pair14              0.500  0.500
## pair15              0.500  0.500  0.500
## treatment21:pair2  -0.354 -0.354 -0.354 -0.354
## treatment22:pair2  -0.354 -0.354 -0.354 -0.354  0.500
## treatment23:pair2  -0.354 -0.354 -0.354 -0.354  0.500  0.500
## treatment24:pair2  -0.354 -0.354 -0.354 -0.354  0.500  0.500  0.500
## treatment25:pair2  -0.354 -0.354 -0.354 -0.354  0.500  0.500  0.500  0.500
## treatment26:pair2  -0.354 -0.354 -0.354 -0.354  0.500  0.500  0.500  0.500
## treatment21:pair3  -0.354 -0.354 -0.354 -0.354  0.500  0.250  0.250  0.250
## treatment22:pair3  -0.354 -0.354 -0.354 -0.354  0.250  0.500  0.250  0.250
## treatment23:pair3  -0.354 -0.354 -0.354 -0.354  0.250  0.250  0.500  0.250
## treatment24:pair3  -0.354 -0.354 -0.354 -0.354  0.250  0.250  0.250  0.500
## treatment25:pair3  -0.354 -0.354 -0.354 -0.354  0.250  0.250  0.250  0.250
## treatment26:pair3  -0.354 -0.354 -0.354 -0.354  0.250  0.250  0.250  0.250
## treatment21:pair4  -0.354 -0.354 -0.354 -0.354  0.500  0.250  0.250  0.250
## treatment22:pair4  -0.354 -0.354 -0.354 -0.354  0.250  0.500  0.250  0.250
## treatment23:pair4  -0.354 -0.354 -0.354 -0.354  0.250  0.250  0.500  0.250
## treatment24:pair4  -0.354 -0.354 -0.354 -0.354  0.250  0.250  0.250  0.500
## treatment25:pair4  -0.354 -0.354 -0.354 -0.354  0.250  0.250  0.250  0.250
## treatment26:pair4  -0.354 -0.354 -0.354 -0.354  0.250  0.250  0.250  0.250
## treatment21:pair5  -0.354 -0.354 -0.354 -0.354  0.500  0.250  0.250  0.250
## treatment22:pair5  -0.354 -0.354 -0.354 -0.354  0.250  0.500  0.250  0.250
## treatment23:pair5  -0.354 -0.354 -0.354 -0.354  0.250  0.250  0.500  0.250
## treatment24:pair5  -0.354 -0.354 -0.354 -0.354  0.250  0.250  0.250  0.500
## treatment25:pair5  -0.354 -0.354 -0.354 -0.354  0.250  0.250  0.250  0.250
## treatment26:pair5  -0.354 -0.354 -0.354 -0.354  0.250  0.250  0.250  0.250
## treatment21:pair6  -0.354 -0.354 -0.354 -0.354  0.500  0.250  0.250  0.250
## treatment22:pair6  -0.354 -0.354 -0.354 -0.354  0.250  0.500  0.250  0.250
## treatment23:pair6  -0.354 -0.354 -0.354 -0.354  0.250  0.250  0.500  0.250
## treatment24:pair6  -0.354 -0.354 -0.354 -0.354  0.250  0.250  0.250  0.500
## treatment25:pair6  -0.354 -0.354 -0.354 -0.354  0.250  0.250  0.250  0.250
## treatment26:pair6  -0.354 -0.354 -0.354 -0.354  0.250  0.250  0.250  0.250
## treatment21:pair7  -0.354 -0.354 -0.354 -0.354  0.500  0.250  0.250  0.250
## treatment22:pair7  -0.354 -0.354 -0.354 -0.354  0.250  0.500  0.250  0.250
## treatment23:pair7  -0.354 -0.354 -0.354 -0.354  0.250  0.250  0.500  0.250
## treatment24:pair7  -0.354 -0.354 -0.354 -0.354  0.250  0.250  0.250  0.500
## treatment25:pair7  -0.354 -0.354 -0.354 -0.354  0.250  0.250  0.250  0.250
## treatment26:pair7  -0.354 -0.354 -0.354 -0.354  0.250  0.250  0.250  0.250
## treatment21:pair9  -0.354 -0.354 -0.354 -0.354  0.500  0.250  0.250  0.250
## treatment22:pair9  -0.354 -0.354 -0.354 -0.354  0.250  0.500  0.250  0.250
## treatment23:pair9  -0.354 -0.354 -0.354 -0.354  0.250  0.250  0.500  0.250
## treatment24:pair9  -0.354 -0.354 -0.354 -0.354  0.250  0.250  0.250  0.500
## treatment25:pair9  -0.354 -0.354 -0.354 -0.354  0.250  0.250  0.250  0.250
## treatment26:pair9  -0.354 -0.354 -0.354 -0.354  0.250  0.250  0.250  0.250
## treatment21:pair10 -0.354 -0.354 -0.354 -0.354  0.500  0.250  0.250  0.250
## treatment22:pair10 -0.354 -0.354 -0.354 -0.354  0.250  0.500  0.250  0.250
## treatment23:pair10 -0.354 -0.354 -0.354 -0.354  0.250  0.250  0.500  0.250
## treatment24:pair10 -0.354 -0.354 -0.354 -0.354  0.250  0.250  0.250  0.500
## treatment25:pair10 -0.354 -0.354 -0.354 -0.354  0.250  0.250  0.250  0.250
## treatment26:pair10 -0.354 -0.354 -0.354 -0.354  0.250  0.250  0.250  0.250
```

```
## treatment21:pair11 -0.354 -0.354 -0.354 -0.354  0.500  0.250  0.250  0.250
## treatment22:pair11 -0.354 -0.354 -0.354 -0.354  0.250  0.500  0.250  0.250
## treatment23:pair11 -0.354 -0.354 -0.354 -0.354  0.250  0.250  0.500  0.250
## treatment24:pair11 -0.354 -0.354 -0.354 -0.354  0.250  0.250  0.250  0.500
## treatment25:pair11 -0.354 -0.354 -0.354 -0.354  0.250  0.250  0.250  0.250
## treatment26:pair11 -0.354 -0.354 -0.354 -0.354  0.250  0.250  0.250  0.250
## treatment21:pair12 -0.707 -0.354 -0.354 -0.354  0.500  0.250  0.250  0.250
## treatment22:pair12 -0.707 -0.354 -0.354 -0.354  0.250  0.500  0.250  0.250
## treatment23:pair12 -0.707 -0.354 -0.354 -0.354  0.250  0.250  0.500  0.250
## treatment24:pair12 -0.707 -0.354 -0.354 -0.354  0.250  0.250  0.250  0.500
## treatment25:pair12 -0.707 -0.354 -0.354 -0.354  0.250  0.250  0.250  0.250
## treatment26:pair12 -0.707 -0.354 -0.354 -0.354  0.250  0.250  0.250  0.250
## treatment21:pair13 -0.354 -0.707 -0.354 -0.354  0.500  0.250  0.250  0.250
## treatment22:pair13 -0.354 -0.707 -0.354 -0.354  0.250  0.500  0.250  0.250
## treatment23:pair13 -0.354 -0.707 -0.354 -0.354  0.250  0.250  0.500  0.250
## treatment24:pair13 -0.354 -0.707 -0.354 -0.354  0.250  0.250  0.250  0.500
## treatment25:pair13 -0.354 -0.707 -0.354 -0.354  0.250  0.250  0.250  0.250
## treatment26:pair13 -0.354 -0.707 -0.354 -0.354  0.250  0.250  0.250  0.250
## treatment21:pair14 -0.354 -0.354 -0.707 -0.354  0.500  0.250  0.250  0.250
## treatment22:pair14 -0.354 -0.354 -0.707 -0.354  0.250  0.500  0.250  0.250
## treatment23:pair14 -0.354 -0.354 -0.707 -0.354  0.250  0.250  0.500  0.250
## treatment24:pair14 -0.354 -0.354 -0.707 -0.354  0.250  0.250  0.250  0.500
## treatment25:pair14 -0.354 -0.354 -0.707 -0.354  0.250  0.250  0.250  0.250
## treatment26:pair14 -0.354 -0.354 -0.707 -0.354  0.250  0.250  0.250  0.250
## treatment21:pair15 -0.354 -0.354 -0.354 -0.707  0.500  0.250  0.250  0.250
## treatment22:pair15 -0.354 -0.354 -0.354 -0.707  0.250  0.500  0.250  0.250
## treatment23:pair15 -0.354 -0.354 -0.354 -0.707  0.250  0.250  0.500  0.250
## treatment24:pair15 -0.354 -0.354 -0.354 -0.707  0.250  0.250  0.250  0.500
## treatment25:pair15 -0.354 -0.354 -0.354 -0.707  0.250  0.250  0.250  0.250
## treatment26:pair15 -0.354 -0.354 -0.354 -0.707  0.250  0.250  0.250  0.250
##                    tr25:2 tr26:2 tr21:3 tr22:3 tr23:3 tr24:3 tr25:3 tr26:3
## treatment21
## treatment22
## treatment23
## treatment24
## treatment25
## treatment26
## pair2
## pair3
## pair4
## pair5
## pair6
## pair7
## pair9
## pair10
## pair11
## pair12
## pair13
## pair14
## pair15
## treatment21:pair2
## treatment22:pair2
## treatment23:pair2
## treatment24:pair2
```

```
## treatment25:pair2
## treatment26:pair2   0.500
## treatment21:pair3   0.250  0.250
## treatment22:pair3   0.250  0.250  0.500
## treatment23:pair3   0.250  0.250  0.500  0.500
## treatment24:pair3   0.250  0.250  0.500  0.500  0.500
## treatment25:pair3   0.500  0.250  0.500  0.500  0.500  0.500
## treatment26:pair3   0.250  0.500  0.500  0.500  0.500  0.500  0.500
## treatment21:pair4   0.250  0.250  0.500  0.250  0.250  0.250  0.250  0.250
## treatment22:pair4   0.250  0.250  0.250  0.500  0.250  0.250  0.250  0.250
## treatment23:pair4   0.250  0.250  0.250  0.250  0.500  0.250  0.250  0.250
## treatment24:pair4   0.250  0.250  0.250  0.250  0.250  0.500  0.250  0.250
## treatment25:pair4   0.500  0.250  0.250  0.250  0.250  0.250  0.500  0.250
## treatment26:pair4   0.250  0.500  0.250  0.250  0.250  0.250  0.250  0.500
## treatment21:pair5   0.250  0.250  0.500  0.250  0.250  0.250  0.250  0.250
## treatment22:pair5   0.250  0.250  0.250  0.500  0.250  0.250  0.250  0.250
## treatment23:pair5   0.250  0.250  0.250  0.250  0.500  0.250  0.250  0.250
## treatment24:pair5   0.250  0.250  0.250  0.250  0.250  0.500  0.250  0.250
## treatment25:pair5   0.500  0.250  0.250  0.250  0.250  0.250  0.500  0.250
## treatment26:pair5   0.250  0.500  0.250  0.250  0.250  0.250  0.250  0.500
## treatment21:pair6   0.250  0.250  0.500  0.250  0.250  0.250  0.250  0.250
## treatment22:pair6   0.250  0.250  0.250  0.500  0.250  0.250  0.250  0.250
## treatment23:pair6   0.250  0.250  0.250  0.250  0.500  0.250  0.250  0.250
## treatment24:pair6   0.250  0.250  0.250  0.250  0.250  0.500  0.250  0.250
## treatment25:pair6   0.500  0.250  0.250  0.250  0.250  0.250  0.500  0.250
## treatment26:pair6   0.250  0.500  0.250  0.250  0.250  0.250  0.250  0.500
## treatment21:pair7   0.250  0.250  0.500  0.250  0.250  0.250  0.250  0.250
## treatment22:pair7   0.250  0.250  0.250  0.500  0.250  0.250  0.250  0.250
## treatment23:pair7   0.250  0.250  0.250  0.250  0.500  0.250  0.250  0.250
## treatment24:pair7   0.250  0.250  0.250  0.250  0.250  0.500  0.250  0.250
## treatment25:pair7   0.500  0.250  0.250  0.250  0.250  0.250  0.500  0.250
## treatment26:pair7   0.250  0.500  0.250  0.250  0.250  0.250  0.250  0.500
## treatment21:pair9   0.250  0.250  0.500  0.250  0.250  0.250  0.250  0.250
## treatment22:pair9   0.250  0.250  0.250  0.500  0.250  0.250  0.250  0.250
## treatment23:pair9   0.250  0.250  0.250  0.250  0.500  0.250  0.250  0.250
## treatment24:pair9   0.250  0.250  0.250  0.250  0.250  0.500  0.250  0.250
## treatment25:pair9   0.500  0.250  0.250  0.250  0.250  0.250  0.500  0.250
## treatment26:pair9   0.250  0.500  0.250  0.250  0.250  0.250  0.250  0.500
## treatment21:pair10  0.250  0.250  0.500  0.250  0.250  0.250  0.250  0.250
## treatment22:pair10  0.250  0.250  0.250  0.500  0.250  0.250  0.250  0.250
## treatment23:pair10  0.250  0.250  0.250  0.250  0.500  0.250  0.250  0.250
## treatment24:pair10  0.250  0.250  0.250  0.250  0.250  0.500  0.250  0.250
## treatment25:pair10  0.500  0.250  0.250  0.250  0.250  0.250  0.500  0.250
## treatment26:pair10  0.250  0.500  0.250  0.250  0.250  0.250  0.250  0.500
## treatment21:pair11  0.250  0.250  0.500  0.250  0.250  0.250  0.250  0.250
## treatment22:pair11  0.250  0.250  0.250  0.500  0.250  0.250  0.250  0.250
## treatment23:pair11  0.250  0.250  0.250  0.250  0.500  0.250  0.250  0.250
## treatment24:pair11  0.250  0.250  0.250  0.250  0.250  0.500  0.250  0.250
## treatment25:pair11  0.500  0.250  0.250  0.250  0.250  0.250  0.500  0.250
## treatment26:pair11  0.250  0.500  0.250  0.250  0.250  0.250  0.250  0.500
## treatment21:pair12  0.250  0.250  0.500  0.250  0.250  0.250  0.250  0.250
## treatment22:pair12  0.250  0.250  0.250  0.500  0.250  0.250  0.250  0.250
## treatment23:pair12  0.250  0.250  0.250  0.250  0.500  0.250  0.250  0.250
## treatment24:pair12  0.250  0.250  0.250  0.250  0.250  0.500  0.250  0.250
```

```
## treatment25:pair12  0.500  0.250  0.250  0.250  0.250  0.250  0.500  0.250
## treatment26:pair12  0.250  0.500  0.250  0.250  0.250  0.250  0.250  0.500
## treatment21:pair13  0.250  0.250  0.500  0.250  0.250  0.250  0.250  0.250
## treatment22:pair13  0.250  0.250  0.250  0.500  0.250  0.250  0.250  0.250
## treatment23:pair13  0.250  0.250  0.250  0.250  0.500  0.250  0.250  0.250
## treatment24:pair13  0.250  0.250  0.250  0.250  0.250  0.500  0.250  0.250
## treatment25:pair13  0.500  0.250  0.250  0.250  0.250  0.250  0.500  0.250
## treatment26:pair13  0.250  0.500  0.250  0.250  0.250  0.250  0.250  0.500
## treatment21:pair14  0.250  0.250  0.500  0.250  0.250  0.250  0.250  0.250
## treatment22:pair14  0.250  0.250  0.250  0.500  0.250  0.250  0.250  0.250
## treatment23:pair14  0.250  0.250  0.250  0.250  0.500  0.250  0.250  0.250
## treatment24:pair14  0.250  0.250  0.250  0.250  0.250  0.500  0.250  0.250
## treatment25:pair14  0.500  0.250  0.250  0.250  0.250  0.250  0.500  0.250
## treatment26:pair14  0.250  0.500  0.250  0.250  0.250  0.250  0.250  0.500
## treatment21:pair15  0.250  0.250  0.500  0.250  0.250  0.250  0.250  0.250
## treatment22:pair15  0.250  0.250  0.250  0.500  0.250  0.250  0.250  0.250
## treatment23:pair15  0.250  0.250  0.250  0.250  0.500  0.250  0.250  0.250
## treatment24:pair15  0.250  0.250  0.250  0.250  0.250  0.500  0.250  0.250
## treatment25:pair15  0.500  0.250  0.250  0.250  0.250  0.250  0.500  0.250
## treatment26:pair15  0.250  0.500  0.250  0.250  0.250  0.250  0.250  0.500
##                    tr21:4 tr22:4 tr23:4 tr24:4 tr25:4 tr26:4 tr21:5 tr22:5
## treatment21
## treatment22
## treatment23
## treatment24
## treatment25
## treatment26
## pair2
## pair3
## pair4
## pair5
## pair6
## pair7
## pair9
## pair10
## pair11
## pair12
## pair13
## pair14
## pair15
## treatment21:pair2
## treatment22:pair2
## treatment23:pair2
## treatment24:pair2
## treatment25:pair2
## treatment26:pair2
## treatment21:pair3
## treatment22:pair3
## treatment23:pair3
## treatment24:pair3
## treatment25:pair3
## treatment26:pair3
## treatment21:pair4
## treatment22:pair4   0.500
```

```
## treatment23:pair4   0.500  0.500
## treatment24:pair4   0.500  0.500  0.500
## treatment25:pair4   0.500  0.500  0.500  0.500
## treatment26:pair4   0.500  0.500  0.500  0.500  0.500
## treatment21:pair5   0.500  0.250  0.250  0.250  0.250  0.250
## treatment22:pair5   0.250  0.500  0.250  0.250  0.250  0.250  0.500
## treatment23:pair5   0.250  0.250  0.500  0.250  0.250  0.250  0.500  0.500
## treatment24:pair5   0.250  0.250  0.250  0.500  0.250  0.250  0.500  0.500
## treatment25:pair5   0.250  0.250  0.250  0.250  0.500  0.250  0.500  0.500
## treatment26:pair5   0.250  0.250  0.250  0.250  0.250  0.500  0.500  0.500
## treatment21:pair6   0.500  0.250  0.250  0.250  0.250  0.250  0.500  0.250
## treatment22:pair6   0.250  0.500  0.250  0.250  0.250  0.250  0.250  0.500
## treatment23:pair6   0.250  0.250  0.500  0.250  0.250  0.250  0.250  0.250
## treatment24:pair6   0.250  0.250  0.250  0.500  0.250  0.250  0.250  0.250
## treatment25:pair6   0.250  0.250  0.250  0.250  0.500  0.250  0.250  0.250
## treatment26:pair6   0.250  0.250  0.250  0.250  0.250  0.500  0.250  0.250
## treatment21:pair7   0.500  0.250  0.250  0.250  0.250  0.250  0.500  0.250
## treatment22:pair7   0.250  0.500  0.250  0.250  0.250  0.250  0.250  0.500
## treatment23:pair7   0.250  0.250  0.500  0.250  0.250  0.250  0.250  0.250
## treatment24:pair7   0.250  0.250  0.250  0.500  0.250  0.250  0.250  0.250
## treatment25:pair7   0.250  0.250  0.250  0.250  0.500  0.250  0.250  0.250
## treatment26:pair7   0.250  0.250  0.250  0.250  0.250  0.500  0.250  0.250
## treatment21:pair9   0.500  0.250  0.250  0.250  0.250  0.250  0.500  0.250
## treatment22:pair9   0.250  0.500  0.250  0.250  0.250  0.250  0.250  0.500
## treatment23:pair9   0.250  0.250  0.500  0.250  0.250  0.250  0.250  0.250
## treatment24:pair9   0.250  0.250  0.250  0.500  0.250  0.250  0.250  0.250
## treatment25:pair9   0.250  0.250  0.250  0.250  0.500  0.250  0.250  0.250
## treatment26:pair9   0.250  0.250  0.250  0.250  0.250  0.500  0.250  0.250
## treatment21:pair10  0.500  0.250  0.250  0.250  0.250  0.250  0.500  0.250
## treatment22:pair10  0.250  0.500  0.250  0.250  0.250  0.250  0.250  0.500
## treatment23:pair10  0.250  0.250  0.500  0.250  0.250  0.250  0.250  0.250
## treatment24:pair10  0.250  0.250  0.250  0.500  0.250  0.250  0.250  0.250
## treatment25:pair10  0.250  0.250  0.250  0.250  0.500  0.250  0.250  0.250
## treatment26:pair10  0.250  0.250  0.250  0.250  0.250  0.500  0.250  0.250
## treatment21:pair11  0.500  0.250  0.250  0.250  0.250  0.250  0.500  0.250
## treatment22:pair11  0.250  0.500  0.250  0.250  0.250  0.250  0.250  0.500
## treatment23:pair11  0.250  0.250  0.500  0.250  0.250  0.250  0.250  0.250
## treatment24:pair11  0.250  0.250  0.250  0.500  0.250  0.250  0.250  0.250
## treatment25:pair11  0.250  0.250  0.250  0.250  0.500  0.250  0.250  0.250
## treatment26:pair11  0.250  0.250  0.250  0.250  0.250  0.500  0.250  0.250
## treatment21:pair12  0.500  0.250  0.250  0.250  0.250  0.250  0.500  0.250
## treatment22:pair12  0.250  0.500  0.250  0.250  0.250  0.250  0.250  0.500
## treatment23:pair12  0.250  0.250  0.500  0.250  0.250  0.250  0.250  0.250
## treatment24:pair12  0.250  0.250  0.250  0.500  0.250  0.250  0.250  0.250
## treatment25:pair12  0.250  0.250  0.250  0.250  0.500  0.250  0.250  0.250
## treatment26:pair12  0.250  0.250  0.250  0.250  0.250  0.500  0.250  0.250
## treatment21:pair13  0.500  0.250  0.250  0.250  0.250  0.250  0.500  0.250
## treatment22:pair13  0.250  0.500  0.250  0.250  0.250  0.250  0.250  0.500
## treatment23:pair13  0.250  0.250  0.500  0.250  0.250  0.250  0.250  0.250
## treatment24:pair13  0.250  0.250  0.250  0.500  0.250  0.250  0.250  0.250
## treatment25:pair13  0.250  0.250  0.250  0.250  0.500  0.250  0.250  0.250
## treatment26:pair13  0.250  0.250  0.250  0.250  0.250  0.500  0.250  0.250
## treatment21:pair14  0.500  0.250  0.250  0.250  0.250  0.250  0.500  0.250
## treatment22:pair14  0.250  0.500  0.250  0.250  0.250  0.250  0.250  0.500
```

```
## treatment23:pair14  0.250  0.250  0.500  0.250  0.250  0.250  0.250  0.250
## treatment24:pair14  0.250  0.250  0.250  0.500  0.250  0.250  0.250  0.250
## treatment25:pair14  0.250  0.250  0.250  0.250  0.500  0.250  0.250  0.250
## treatment26:pair14  0.250  0.250  0.250  0.250  0.250  0.500  0.250  0.250
## treatment21:pair15  0.500  0.250  0.250  0.250  0.250  0.250  0.500  0.250
## treatment22:pair15  0.250  0.500  0.250  0.250  0.250  0.250  0.250  0.500
## treatment23:pair15  0.250  0.250  0.500  0.250  0.250  0.250  0.250  0.250
## treatment24:pair15  0.250  0.250  0.250  0.500  0.250  0.250  0.250  0.250
## treatment25:pair15  0.250  0.250  0.250  0.250  0.500  0.250  0.250  0.250
## treatment26:pair15  0.250  0.250  0.250  0.250  0.250  0.500  0.250  0.250
##                    tr23:5 tr24:5 tr25:5 tr26:5 tr21:6 tr22:6 tr23:6 tr24:6
## treatment21
## treatment22
## treatment23
## treatment24
## treatment25
## treatment26
## pair2
## pair3
## pair4
## pair5
## pair6
## pair7
## pair9
## pair10
## pair11
## pair12
## pair13
## pair14
## pair15
## treatment21:pair2
## treatment22:pair2
## treatment23:pair2
## treatment24:pair2
## treatment25:pair2
## treatment26:pair2
## treatment21:pair3
## treatment22:pair3
## treatment23:pair3
## treatment24:pair3
## treatment25:pair3
## treatment26:pair3
## treatment21:pair4
## treatment22:pair4
## treatment23:pair4
## treatment24:pair4
## treatment25:pair4
## treatment26:pair4
## treatment21:pair5
## treatment22:pair5
## treatment23:pair5
## treatment24:pair5   0.500
## treatment25:pair5   0.500  0.500
## treatment26:pair5   0.500  0.500  0.500
```

```
## treatment21:pair6   0.250  0.250  0.250  0.250
## treatment22:pair6   0.250  0.250  0.250  0.250  0.500
## treatment23:pair6   0.500  0.250  0.250  0.250  0.500  0.500
## treatment24:pair6   0.250  0.500  0.250  0.250  0.500  0.500  0.500
## treatment25:pair6   0.250  0.250  0.500  0.250  0.500  0.500  0.500  0.500
## treatment26:pair6   0.250  0.250  0.250  0.500  0.500  0.500  0.500  0.500
## treatment21:pair7   0.250  0.250  0.250  0.250  0.500  0.250  0.250  0.250
## treatment22:pair7   0.250  0.250  0.250  0.250  0.250  0.500  0.250  0.250
## treatment23:pair7   0.500  0.250  0.250  0.250  0.250  0.250  0.500  0.250
## treatment24:pair7   0.250  0.500  0.250  0.250  0.250  0.250  0.250  0.500
## treatment25:pair7   0.250  0.250  0.500  0.250  0.250  0.250  0.250  0.250
## treatment26:pair7   0.250  0.250  0.250  0.500  0.250  0.250  0.250  0.250
## treatment21:pair9   0.250  0.250  0.250  0.250  0.500  0.250  0.250  0.250
## treatment22:pair9   0.250  0.250  0.250  0.250  0.250  0.500  0.250  0.250
## treatment23:pair9   0.500  0.250  0.250  0.250  0.250  0.250  0.500  0.250
## treatment24:pair9   0.250  0.500  0.250  0.250  0.250  0.250  0.250  0.500
## treatment25:pair9   0.250  0.250  0.500  0.250  0.250  0.250  0.250  0.250
## treatment26:pair9   0.250  0.250  0.250  0.500  0.250  0.250  0.250  0.250
## treatment21:pair10  0.250  0.250  0.250  0.250  0.500  0.250  0.250  0.250
## treatment22:pair10  0.250  0.250  0.250  0.250  0.250  0.500  0.250  0.250
## treatment23:pair10  0.500  0.250  0.250  0.250  0.250  0.250  0.500  0.250
## treatment24:pair10  0.250  0.500  0.250  0.250  0.250  0.250  0.250  0.500
## treatment25:pair10  0.250  0.250  0.500  0.250  0.250  0.250  0.250  0.250
## treatment26:pair10  0.250  0.250  0.250  0.500  0.250  0.250  0.250  0.250
## treatment21:pair11  0.250  0.250  0.250  0.250  0.500  0.250  0.250  0.250
## treatment22:pair11  0.250  0.250  0.250  0.250  0.250  0.500  0.250  0.250
## treatment23:pair11  0.500  0.250  0.250  0.250  0.250  0.250  0.500  0.250
## treatment24:pair11  0.250  0.500  0.250  0.250  0.250  0.250  0.250  0.500
## treatment25:pair11  0.250  0.250  0.500  0.250  0.250  0.250  0.250  0.250
## treatment26:pair11  0.250  0.250  0.250  0.500  0.250  0.250  0.250  0.250
## treatment21:pair12  0.250  0.250  0.250  0.250  0.500  0.250  0.250  0.250
## treatment22:pair12  0.250  0.250  0.250  0.250  0.250  0.500  0.250  0.250
## treatment23:pair12  0.500  0.250  0.250  0.250  0.250  0.250  0.500  0.250
## treatment24:pair12  0.250  0.500  0.250  0.250  0.250  0.250  0.250  0.500
## treatment25:pair12  0.250  0.250  0.500  0.250  0.250  0.250  0.250  0.250
## treatment26:pair12  0.250  0.250  0.250  0.500  0.250  0.250  0.250  0.250
## treatment21:pair13  0.250  0.250  0.250  0.250  0.500  0.250  0.250  0.250
## treatment22:pair13  0.250  0.250  0.250  0.250  0.250  0.500  0.250  0.250
## treatment23:pair13  0.500  0.250  0.250  0.250  0.250  0.250  0.500  0.250
## treatment24:pair13  0.250  0.500  0.250  0.250  0.250  0.250  0.250  0.500
## treatment25:pair13  0.250  0.250  0.500  0.250  0.250  0.250  0.250  0.250
## treatment26:pair13  0.250  0.250  0.250  0.500  0.250  0.250  0.250  0.250
## treatment21:pair14  0.250  0.250  0.250  0.250  0.500  0.250  0.250  0.250
## treatment22:pair14  0.250  0.250  0.250  0.250  0.250  0.500  0.250  0.250
## treatment23:pair14  0.500  0.250  0.250  0.250  0.250  0.250  0.500  0.250
## treatment24:pair14  0.250  0.500  0.250  0.250  0.250  0.250  0.250  0.500
## treatment25:pair14  0.250  0.250  0.500  0.250  0.250  0.250  0.250  0.250
## treatment26:pair14  0.250  0.250  0.250  0.500  0.250  0.250  0.250  0.250
## treatment21:pair15  0.250  0.250  0.250  0.250  0.500  0.250  0.250  0.250
## treatment22:pair15  0.250  0.250  0.250  0.250  0.250  0.500  0.250  0.250
## treatment23:pair15  0.500  0.250  0.250  0.250  0.250  0.250  0.500  0.250
## treatment24:pair15  0.250  0.500  0.250  0.250  0.250  0.250  0.250  0.500
## treatment25:pair15  0.250  0.250  0.500  0.250  0.250  0.250  0.250  0.250
## treatment26:pair15  0.250  0.250  0.250  0.500  0.250  0.250  0.250  0.250
```

```
##                      tr25:6 tr26:6 tr21:7 tr22:7 tr23:7 tr24:7 tr25:7 tr26:7
## treatment21
## treatment22
## treatment23
## treatment24
## treatment25
## treatment26
## pair2
## pair3
## pair4
## pair5
## pair6
## pair7
## pair9
## pair10
## pair11
## pair12
## pair13
## pair14
## pair15
## treatment21:pair2
## treatment22:pair2
## treatment23:pair2
## treatment24:pair2
## treatment25:pair2
## treatment26:pair2
## treatment21:pair3
## treatment22:pair3
## treatment23:pair3
## treatment24:pair3
## treatment25:pair3
## treatment26:pair3
## treatment21:pair4
## treatment22:pair4
## treatment23:pair4
## treatment24:pair4
## treatment25:pair4
## treatment26:pair4
## treatment21:pair5
## treatment22:pair5
## treatment23:pair5
## treatment24:pair5
## treatment25:pair5
## treatment26:pair5
## treatment21:pair6
## treatment22:pair6
## treatment23:pair6
## treatment24:pair6
## treatment25:pair6
## treatment26:pair6   0.500
## treatment21:pair7   0.250  0.250
## treatment22:pair7   0.250  0.250  0.500
## treatment23:pair7   0.250  0.250  0.500  0.500
## treatment24:pair7   0.250  0.250  0.500  0.500  0.500
```

```
## treatment25:pair7   0.500  0.250  0.500  0.500  0.500  0.500
## treatment26:pair7   0.250  0.500  0.500  0.500  0.500  0.500  0.500
## treatment21:pair9   0.250  0.250  0.500  0.250  0.250  0.250  0.250  0.250
## treatment22:pair9   0.250  0.250  0.250  0.500  0.250  0.250  0.250  0.250
## treatment23:pair9   0.250  0.250  0.250  0.250  0.500  0.250  0.250  0.250
## treatment24:pair9   0.250  0.250  0.250  0.250  0.250  0.500  0.250  0.250
## treatment25:pair9   0.500  0.250  0.250  0.250  0.250  0.250  0.500  0.250
## treatment26:pair9   0.250  0.500  0.250  0.250  0.250  0.250  0.250  0.500
## treatment21:pair10  0.250  0.250  0.500  0.250  0.250  0.250  0.250  0.250
## treatment22:pair10  0.250  0.250  0.250  0.500  0.250  0.250  0.250  0.250
## treatment23:pair10  0.250  0.250  0.250  0.250  0.500  0.250  0.250  0.250
## treatment24:pair10  0.250  0.250  0.250  0.250  0.250  0.500  0.250  0.250
## treatment25:pair10  0.500  0.250  0.250  0.250  0.250  0.250  0.500  0.250
## treatment26:pair10  0.250  0.500  0.250  0.250  0.250  0.250  0.250  0.500
## treatment21:pair11  0.250  0.250  0.500  0.250  0.250  0.250  0.250  0.250
## treatment22:pair11  0.250  0.250  0.250  0.500  0.250  0.250  0.250  0.250
## treatment23:pair11  0.250  0.250  0.250  0.250  0.500  0.250  0.250  0.250
## treatment24:pair11  0.250  0.250  0.250  0.250  0.250  0.500  0.250  0.250
## treatment25:pair11  0.500  0.250  0.250  0.250  0.250  0.250  0.500  0.250
## treatment26:pair11  0.250  0.500  0.250  0.250  0.250  0.250  0.250  0.500
## treatment21:pair12  0.250  0.250  0.500  0.250  0.250  0.250  0.250  0.250
## treatment22:pair12  0.250  0.250  0.250  0.500  0.250  0.250  0.250  0.250
## treatment23:pair12  0.250  0.250  0.250  0.250  0.500  0.250  0.250  0.250
## treatment24:pair12  0.250  0.250  0.250  0.250  0.250  0.500  0.250  0.250
## treatment25:pair12  0.500  0.250  0.250  0.250  0.250  0.250  0.500  0.250
## treatment26:pair12  0.250  0.500  0.250  0.250  0.250  0.250  0.250  0.500
## treatment21:pair13  0.250  0.250  0.500  0.250  0.250  0.250  0.250  0.250
## treatment22:pair13  0.250  0.250  0.250  0.500  0.250  0.250  0.250  0.250
## treatment23:pair13  0.250  0.250  0.250  0.250  0.500  0.250  0.250  0.250
## treatment24:pair13  0.250  0.250  0.250  0.250  0.250  0.500  0.250  0.250
## treatment25:pair13  0.500  0.250  0.250  0.250  0.250  0.250  0.500  0.250
## treatment26:pair13  0.250  0.500  0.250  0.250  0.250  0.250  0.250  0.500
## treatment21:pair14  0.250  0.250  0.500  0.250  0.250  0.250  0.250  0.250
## treatment22:pair14  0.250  0.250  0.250  0.500  0.250  0.250  0.250  0.250
## treatment23:pair14  0.250  0.250  0.250  0.250  0.500  0.250  0.250  0.250
## treatment24:pair14  0.250  0.250  0.250  0.250  0.250  0.500  0.250  0.250
## treatment25:pair14  0.500  0.250  0.250  0.250  0.250  0.250  0.500  0.250
## treatment26:pair14  0.250  0.500  0.250  0.250  0.250  0.250  0.250  0.500
## treatment21:pair15  0.250  0.250  0.500  0.250  0.250  0.250  0.250  0.250
## treatment22:pair15  0.250  0.250  0.250  0.500  0.250  0.250  0.250  0.250
## treatment23:pair15  0.250  0.250  0.250  0.250  0.500  0.250  0.250  0.250
## treatment24:pair15  0.250  0.250  0.250  0.250  0.250  0.500  0.250  0.250
## treatment25:pair15  0.500  0.250  0.250  0.250  0.250  0.250  0.500  0.250
## treatment26:pair15  0.250  0.500  0.250  0.250  0.250  0.250  0.250  0.500
##                    tr21:9 tr22:9 tr23:9 tr24:9 tr25:9 tr26:9 t21:10 t22:10
## treatment21
## treatment22
## treatment23
## treatment24
## treatment25
## treatment26
## pair2
## pair3
## pair4
```

```
## pair5
## pair6
## pair7
## pair9
## pair10
## pair11
## pair12
## pair13
## pair14
## pair15
## treatment21:pair2
## treatment22:pair2
## treatment23:pair2
## treatment24:pair2
## treatment25:pair2
## treatment26:pair2
## treatment21:pair3
## treatment22:pair3
## treatment23:pair3
## treatment24:pair3
## treatment25:pair3
## treatment26:pair3
## treatment21:pair4
## treatment22:pair4
## treatment23:pair4
## treatment24:pair4
## treatment25:pair4
## treatment26:pair4
## treatment21:pair5
## treatment22:pair5
## treatment23:pair5
## treatment24:pair5
## treatment25:pair5
## treatment26:pair5
## treatment21:pair6
## treatment22:pair6
## treatment23:pair6
## treatment24:pair6
## treatment25:pair6
## treatment26:pair6
## treatment21:pair7
## treatment22:pair7
## treatment23:pair7
## treatment24:pair7
## treatment25:pair7
## treatment26:pair7
## treatment21:pair9
## treatment22:pair9   0.500
## treatment23:pair9   0.500  0.500
## treatment24:pair9   0.500  0.500  0.500
## treatment25:pair9   0.500  0.500  0.500  0.500
## treatment26:pair9   0.500  0.500  0.500  0.500  0.500
## treatment21:pair10  0.500  0.250  0.250  0.250  0.250  0.250
## treatment22:pair10  0.250  0.500  0.250  0.250  0.250  0.250  0.500
```

```
## treatment23:pair10  0.250  0.250  0.500  0.250  0.250  0.250  0.500  0.500
## treatment24:pair10  0.250  0.250  0.250  0.500  0.250  0.250  0.500  0.500
## treatment25:pair10  0.250  0.250  0.250  0.250  0.500  0.250  0.500  0.500
## treatment26:pair10  0.250  0.250  0.250  0.250  0.250  0.500  0.500  0.500
## treatment21:pair11  0.500  0.250  0.250  0.250  0.250  0.250  0.500  0.250
## treatment22:pair11  0.250  0.500  0.250  0.250  0.250  0.250  0.250  0.500
## treatment23:pair11  0.250  0.250  0.500  0.250  0.250  0.250  0.250  0.250
## treatment24:pair11  0.250  0.250  0.250  0.500  0.250  0.250  0.250  0.250
## treatment25:pair11  0.250  0.250  0.250  0.250  0.500  0.250  0.250  0.250
## treatment26:pair11  0.250  0.250  0.250  0.250  0.250  0.500  0.250  0.250
## treatment21:pair12  0.500  0.250  0.250  0.250  0.250  0.250  0.500  0.250
## treatment22:pair12  0.250  0.500  0.250  0.250  0.250  0.250  0.250  0.500
## treatment23:pair12  0.250  0.250  0.500  0.250  0.250  0.250  0.250  0.250
## treatment24:pair12  0.250  0.250  0.250  0.500  0.250  0.250  0.250  0.250
## treatment25:pair12  0.250  0.250  0.250  0.250  0.500  0.250  0.250  0.250
## treatment26:pair12  0.250  0.250  0.250  0.250  0.250  0.500  0.250  0.250
## treatment21:pair13  0.500  0.250  0.250  0.250  0.250  0.250  0.500  0.250
## treatment22:pair13  0.250  0.500  0.250  0.250  0.250  0.250  0.250  0.500
## treatment23:pair13  0.250  0.250  0.500  0.250  0.250  0.250  0.250  0.250
## treatment24:pair13  0.250  0.250  0.250  0.500  0.250  0.250  0.250  0.250
## treatment25:pair13  0.250  0.250  0.250  0.250  0.500  0.250  0.250  0.250
## treatment26:pair13  0.250  0.250  0.250  0.250  0.250  0.500  0.250  0.250
## treatment21:pair14  0.500  0.250  0.250  0.250  0.250  0.250  0.500  0.250
## treatment22:pair14  0.250  0.500  0.250  0.250  0.250  0.250  0.250  0.500
## treatment23:pair14  0.250  0.250  0.500  0.250  0.250  0.250  0.250  0.250
## treatment24:pair14  0.250  0.250  0.250  0.500  0.250  0.250  0.250  0.250
## treatment25:pair14  0.250  0.250  0.250  0.250  0.500  0.250  0.250  0.250
## treatment26:pair14  0.250  0.250  0.250  0.250  0.250  0.500  0.250  0.250
## treatment21:pair15  0.500  0.250  0.250  0.250  0.250  0.250  0.500  0.250
## treatment22:pair15  0.250  0.500  0.250  0.250  0.250  0.250  0.250  0.500
## treatment23:pair15  0.250  0.250  0.500  0.250  0.250  0.250  0.250  0.250
## treatment24:pair15  0.250  0.250  0.250  0.500  0.250  0.250  0.250  0.250
## treatment25:pair15  0.250  0.250  0.250  0.250  0.500  0.250  0.250  0.250
## treatment26:pair15  0.250  0.250  0.250  0.250  0.250  0.500  0.250  0.250
##                    t23:10 t24:10 t25:10 t26:10 t21:11 t22:11 t23:11 t24:11
## treatment21
## treatment22
## treatment23
## treatment24
## treatment25
## treatment26
## pair2
## pair3
## pair4
## pair5
## pair6
## pair7
## pair9
## pair10
## pair11
## pair12
## pair13
## pair14
## pair15
```

```
## treatment21:pair2
## treatment22:pair2
## treatment23:pair2
## treatment24:pair2
## treatment25:pair2
## treatment26:pair2
## treatment21:pair3
## treatment22:pair3
## treatment23:pair3
## treatment24:pair3
## treatment25:pair3
## treatment26:pair3
## treatment21:pair4
## treatment22:pair4
## treatment23:pair4
## treatment24:pair4
## treatment25:pair4
## treatment26:pair4
## treatment21:pair5
## treatment22:pair5
## treatment23:pair5
## treatment24:pair5
## treatment25:pair5
## treatment26:pair5
## treatment21:pair6
## treatment22:pair6
## treatment23:pair6
## treatment24:pair6
## treatment25:pair6
## treatment26:pair6
## treatment21:pair7
## treatment22:pair7
## treatment23:pair7
## treatment24:pair7
## treatment25:pair7
## treatment26:pair7
## treatment21:pair9
## treatment22:pair9
## treatment23:pair9
## treatment24:pair9
## treatment25:pair9
## treatment26:pair9
## treatment21:pair10
## treatment22:pair10
## treatment23:pair10
## treatment24:pair10  0.500
## treatment25:pair10  0.500  0.500
## treatment26:pair10  0.500  0.500  0.500
## treatment21:pair11  0.250  0.250  0.250  0.250
## treatment22:pair11  0.250  0.250  0.250  0.250  0.500
## treatment23:pair11  0.500  0.250  0.250  0.250  0.500  0.500
## treatment24:pair11  0.250  0.500  0.250  0.250  0.500  0.500  0.500
## treatment25:pair11  0.250  0.250  0.500  0.250  0.500  0.500  0.500  0.500
## treatment26:pair11  0.250  0.250  0.250  0.500  0.500  0.500  0.500  0.500
```

```
## treatment21:pair12  0.250  0.250  0.250  0.250  0.500  0.250  0.250  0.250
## treatment22:pair12  0.250  0.250  0.250  0.250  0.250  0.500  0.250  0.250
## treatment23:pair12  0.500  0.250  0.250  0.250  0.250  0.250  0.500  0.250
## treatment24:pair12  0.250  0.500  0.250  0.250  0.250  0.250  0.250  0.500
## treatment25:pair12  0.250  0.250  0.500  0.250  0.250  0.250  0.250  0.250
## treatment26:pair12  0.250  0.250  0.250  0.500  0.250  0.250  0.250  0.250
## treatment21:pair13  0.250  0.250  0.250  0.250  0.500  0.250  0.250  0.250
## treatment22:pair13  0.250  0.250  0.250  0.250  0.250  0.500  0.250  0.250
## treatment23:pair13  0.500  0.250  0.250  0.250  0.250  0.250  0.500  0.250
## treatment24:pair13  0.250  0.500  0.250  0.250  0.250  0.250  0.250  0.500
## treatment25:pair13  0.250  0.250  0.500  0.250  0.250  0.250  0.250  0.250
## treatment26:pair13  0.250  0.250  0.250  0.500  0.250  0.250  0.250  0.250
## treatment21:pair14  0.250  0.250  0.250  0.250  0.500  0.250  0.250  0.250
## treatment22:pair14  0.250  0.250  0.250  0.250  0.250  0.500  0.250  0.250
## treatment23:pair14  0.500  0.250  0.250  0.250  0.250  0.250  0.500  0.250
## treatment24:pair14  0.250  0.500  0.250  0.250  0.250  0.250  0.250  0.500
## treatment25:pair14  0.250  0.250  0.500  0.250  0.250  0.250  0.250  0.250
## treatment26:pair14  0.250  0.250  0.250  0.500  0.250  0.250  0.250  0.250
## treatment21:pair15  0.250  0.250  0.250  0.250  0.500  0.250  0.250  0.250
## treatment22:pair15  0.250  0.250  0.250  0.250  0.250  0.500  0.250  0.250
## treatment23:pair15  0.500  0.250  0.250  0.250  0.250  0.250  0.500  0.250
## treatment24:pair15  0.250  0.500  0.250  0.250  0.250  0.250  0.250  0.500
## treatment25:pair15  0.250  0.250  0.500  0.250  0.250  0.250  0.250  0.250
## treatment26:pair15  0.250  0.250  0.250  0.500  0.250  0.250  0.250  0.250
##                    t25:11 t26:11 t21:12 t22:12 t23:12 t24:12 t25:12 t26:12
## treatment21
## treatment22
## treatment23
## treatment24
## treatment25
## treatment26
## pair2
## pair3
## pair4
## pair5
## pair6
## pair7
## pair9
## pair10
## pair11
## pair12
## pair13
## pair14
## pair15
## treatment21:pair2
## treatment22:pair2
## treatment23:pair2
## treatment24:pair2
## treatment25:pair2
## treatment26:pair2
## treatment21:pair3
## treatment22:pair3
## treatment23:pair3
## treatment24:pair3
```

```
## treatment25:pair3
## treatment26:pair3
## treatment21:pair4
## treatment22:pair4
## treatment23:pair4
## treatment24:pair4
## treatment25:pair4
## treatment26:pair4
## treatment21:pair5
## treatment22:pair5
## treatment23:pair5
## treatment24:pair5
## treatment25:pair5
## treatment26:pair5
## treatment21:pair6
## treatment22:pair6
## treatment23:pair6
## treatment24:pair6
## treatment25:pair6
## treatment26:pair6
## treatment21:pair7
## treatment22:pair7
## treatment23:pair7
## treatment24:pair7
## treatment25:pair7
## treatment26:pair7
## treatment21:pair9
## treatment22:pair9
## treatment23:pair9
## treatment24:pair9
## treatment25:pair9
## treatment26:pair9
## treatment21:pair10
## treatment22:pair10
## treatment23:pair10
## treatment24:pair10
## treatment25:pair10
## treatment26:pair10
## treatment21:pair11
## treatment22:pair11
## treatment23:pair11
## treatment24:pair11
## treatment25:pair11
## treatment26:pair11  0.500
## treatment21:pair12  0.250  0.250
## treatment22:pair12  0.250  0.250  0.500
## treatment23:pair12  0.250  0.250  0.500  0.500
## treatment24:pair12  0.250  0.250  0.500  0.500  0.500
## treatment25:pair12  0.500  0.250  0.500  0.500  0.500  0.500
## treatment26:pair12  0.250  0.500  0.500  0.500  0.500  0.500  0.500
## treatment21:pair13  0.250  0.250  0.500  0.250  0.250  0.250  0.250  0.250
## treatment22:pair13  0.250  0.250  0.250  0.500  0.250  0.250  0.250  0.250
## treatment23:pair13  0.250  0.250  0.250  0.250  0.500  0.250  0.250  0.250
## treatment24:pair13  0.250  0.250  0.250  0.250  0.250  0.500  0.250  0.250
```

```
## treatment25:pair13  0.500  0.250  0.250  0.250  0.250  0.250  0.500  0.250
## treatment26:pair13  0.250  0.500  0.250  0.250  0.250  0.250  0.250  0.500
## treatment21:pair14  0.250  0.250  0.500  0.250  0.250  0.250  0.250  0.250
## treatment22:pair14  0.250  0.250  0.250  0.500  0.250  0.250  0.250  0.250
## treatment23:pair14  0.250  0.250  0.250  0.250  0.500  0.250  0.250  0.250
## treatment24:pair14  0.250  0.250  0.250  0.250  0.250  0.500  0.250  0.250
## treatment25:pair14  0.500  0.250  0.250  0.250  0.250  0.250  0.500  0.250
## treatment26:pair14  0.250  0.500  0.250  0.250  0.250  0.250  0.250  0.500
## treatment21:pair15  0.250  0.250  0.500  0.250  0.250  0.250  0.250  0.250
## treatment22:pair15  0.250  0.250  0.250  0.500  0.250  0.250  0.250  0.250
## treatment23:pair15  0.250  0.250  0.250  0.250  0.500  0.250  0.250  0.250
## treatment24:pair15  0.250  0.250  0.250  0.250  0.250  0.500  0.250  0.250
## treatment25:pair15  0.500  0.250  0.250  0.250  0.250  0.250  0.500  0.250
## treatment26:pair15  0.250  0.500  0.250  0.250  0.250  0.250  0.250  0.500
##                    t21:13 t22:13 t23:13 t24:13 t25:13 t26:13 t21:14 t22:14
## treatment21
## treatment22
## treatment23
## treatment24
## treatment25
## treatment26
## pair2
## pair3
## pair4
## pair5
## pair6
## pair7
## pair9
## pair10
## pair11
## pair12
## pair13
## pair14
## pair15
## treatment21:pair2
## treatment22:pair2
## treatment23:pair2
## treatment24:pair2
## treatment25:pair2
## treatment26:pair2
## treatment21:pair3
## treatment22:pair3
## treatment23:pair3
## treatment24:pair3
## treatment25:pair3
## treatment26:pair3
## treatment21:pair4
## treatment22:pair4
## treatment23:pair4
## treatment24:pair4
## treatment25:pair4
## treatment26:pair4
## treatment21:pair5
## treatment22:pair5
```

```
## treatment23:pair5
## treatment24:pair5
## treatment25:pair5
## treatment26:pair5
## treatment21:pair6
## treatment22:pair6
## treatment23:pair6
## treatment24:pair6
## treatment25:pair6
## treatment26:pair6
## treatment21:pair7
## treatment22:pair7
## treatment23:pair7
## treatment24:pair7
## treatment25:pair7
## treatment26:pair7
## treatment21:pair9
## treatment22:pair9
## treatment23:pair9
## treatment24:pair9
## treatment25:pair9
## treatment26:pair9
## treatment21:pair10
## treatment22:pair10
## treatment23:pair10
## treatment24:pair10
## treatment25:pair10
## treatment26:pair10
## treatment21:pair11
## treatment22:pair11
## treatment23:pair11
## treatment24:pair11
## treatment25:pair11
## treatment26:pair11
## treatment21:pair12
## treatment22:pair12
## treatment23:pair12
## treatment24:pair12
## treatment25:pair12
## treatment26:pair12
## treatment21:pair13
## treatment22:pair13  0.500
## treatment23:pair13  0.500  0.500
## treatment24:pair13  0.500  0.500  0.500
## treatment25:pair13  0.500  0.500  0.500  0.500
## treatment26:pair13  0.500  0.500  0.500  0.500  0.500
## treatment21:pair14  0.500  0.250  0.250  0.250  0.250  0.250
## treatment22:pair14  0.250  0.500  0.250  0.250  0.250  0.250  0.500
## treatment23:pair14  0.250  0.250  0.500  0.250  0.250  0.250  0.500  0.500
## treatment24:pair14  0.250  0.250  0.250  0.500  0.250  0.250  0.500  0.500
## treatment25:pair14  0.250  0.250  0.250  0.250  0.500  0.250  0.500  0.500
## treatment26:pair14  0.250  0.250  0.250  0.250  0.250  0.500  0.500  0.500
## treatment21:pair15  0.500  0.250  0.250  0.250  0.250  0.250  0.500  0.250
## treatment22:pair15  0.250  0.500  0.250  0.250  0.250  0.250  0.250  0.500
```

```
## treatment23:pair15  0.250  0.250  0.500  0.250  0.250  0.250  0.250  0.250
## treatment24:pair15  0.250  0.250  0.250  0.500  0.250  0.250  0.250  0.250
## treatment25:pair15  0.250  0.250  0.250  0.250  0.500  0.250  0.250  0.250
## treatment26:pair15  0.250  0.250  0.250  0.250  0.250  0.500  0.250  0.250
##                    t23:14 t24:14 t25:14 t26:14 t21:15 t22:15 t23:15 t24:15
## treatment21
## treatment22
## treatment23
## treatment24
## treatment25
## treatment26
## pair2
## pair3
## pair4
## pair5
## pair6
## pair7
## pair9
## pair10
## pair11
## pair12
## pair13
## pair14
## pair15
## treatment21:pair2
## treatment22:pair2
## treatment23:pair2
## treatment24:pair2
## treatment25:pair2
## treatment26:pair2
## treatment21:pair3
## treatment22:pair3
## treatment23:pair3
## treatment24:pair3
## treatment25:pair3
## treatment26:pair3
## treatment21:pair4
## treatment22:pair4
## treatment23:pair4
## treatment24:pair4
## treatment25:pair4
## treatment26:pair4
## treatment21:pair5
## treatment22:pair5
## treatment23:pair5
## treatment24:pair5
## treatment25:pair5
## treatment26:pair5
## treatment21:pair6
## treatment22:pair6
## treatment23:pair6
## treatment24:pair6
## treatment25:pair6
## treatment26:pair6
```

```
## treatment21:pair7
## treatment22:pair7
## treatment23:pair7
## treatment24:pair7
## treatment25:pair7
## treatment26:pair7
## treatment21:pair9
## treatment22:pair9
## treatment23:pair9
## treatment24:pair9
## treatment25:pair9
## treatment26:pair9
## treatment21:pair10
## treatment22:pair10
## treatment23:pair10
## treatment24:pair10
## treatment25:pair10
## treatment26:pair10
## treatment21:pair11
## treatment22:pair11
## treatment23:pair11
## treatment24:pair11
## treatment25:pair11
## treatment26:pair11
## treatment21:pair12
## treatment22:pair12
## treatment23:pair12
## treatment24:pair12
## treatment25:pair12
## treatment26:pair12
## treatment21:pair13
## treatment22:pair13
## treatment23:pair13
## treatment24:pair13
## treatment25:pair13
## treatment26:pair13
## treatment21:pair14
## treatment22:pair14
## treatment23:pair14
## treatment24:pair14  0.500
## treatment25:pair14  0.500  0.500
## treatment26:pair14  0.500  0.500  0.500
## treatment21:pair15  0.250  0.250  0.250  0.250
## treatment22:pair15  0.250  0.250  0.250  0.250  0.500
## treatment23:pair15  0.500  0.250  0.250  0.250  0.500  0.500
## treatment24:pair15  0.250  0.500  0.250  0.250  0.500  0.500  0.500
## treatment25:pair15  0.250  0.250  0.500  0.250  0.500  0.500  0.500  0.500
## treatment26:pair15  0.250  0.250  0.250  0.500  0.500  0.500  0.500  0.500
##                    t25:15
## treatment21
## treatment22
## treatment23
## treatment24
## treatment25
```

```
## treatment26
## pair2
## pair3
## pair4
## pair5
## pair6
## pair7
## pair9
## pair10
## pair11
## pair12
## pair13
## pair14
## pair15
## treatment21:pair2
## treatment22:pair2
## treatment23:pair2
## treatment24:pair2
## treatment25:pair2
## treatment26:pair2
## treatment21:pair3
## treatment22:pair3
## treatment23:pair3
## treatment24:pair3
## treatment25:pair3
## treatment26:pair3
## treatment21:pair4
## treatment22:pair4
## treatment23:pair4
## treatment24:pair4
## treatment25:pair4
## treatment26:pair4
## treatment21:pair5
## treatment22:pair5
## treatment23:pair5
## treatment24:pair5
## treatment25:pair5
## treatment26:pair5
## treatment21:pair6
## treatment22:pair6
## treatment23:pair6
## treatment24:pair6
## treatment25:pair6
## treatment26:pair6
## treatment21:pair7
## treatment22:pair7
## treatment23:pair7
## treatment24:pair7
## treatment25:pair7
## treatment26:pair7
## treatment21:pair9
## treatment22:pair9
## treatment23:pair9
## treatment24:pair9
```

```
## treatment25:pair9
## treatment26:pair9
## treatment21:pair10
## treatment22:pair10
## treatment23:pair10
## treatment24:pair10
## treatment25:pair10
## treatment26:pair10
## treatment21:pair11
## treatment22:pair11
## treatment23:pair11
## treatment24:pair11
## treatment25:pair11
## treatment26:pair11
## treatment21:pair12
## treatment22:pair12
## treatment23:pair12
## treatment24:pair12
## treatment25:pair12
## treatment26:pair12
## treatment21:pair13
## treatment22:pair13
## treatment23:pair13
## treatment24:pair13
## treatment25:pair13
## treatment26:pair13
## treatment21:pair14
## treatment22:pair14
## treatment23:pair14
## treatment24:pair14
## treatment25:pair14
## treatment26:pair14
## treatment21:pair15
## treatment22:pair15
## treatment23:pair15
## treatment24:pair15
## treatment25:pair15
## treatment26:pair15  0.500
##
## Standardized Within-Group Residuals:
##         Min          Q1         Med          Q3         Max
## -2.23840128 -0.43905380 -0.02047809  0.36591518  3.21135044
##
## Number of Observations: 294
## Number of Groups: 21
```

Combined analysis of both strains under the same stress

Salt:

```
data1<-read.table("~/Desktop/rsync/deteriorating treatments/salt/2344_salt_v2_[0].csv",h=T)
data2<-read.table("~/Desktop/rsync/deteriorating treatments/salt/2931_salt_v2_[0].csv",h=T)
datac<-rbind(data1,data2)
```

```r
datac<-subset(datac,treatment<7)
datac$treatment2<-as.factor(datac$treatment)
datac$pair<-as.factor(datac$pair)

fit2<-lmer(relfitav~treatment2*pair+(1|strain)+(1|plate),datac)
anova(fit2)
```

```
## Analysis of Variance Table of type III  with  Satterthwaite
## approximation for degrees of freedom
##                   Sum Sq Mean Sq NumDF  DenDF F.value    Pr(>F)
## treatment2        2.8191 0.46986     6  14.47  5.2127  0.004801 **
## pair              8.6995 0.62139    14 487.13  6.8939 5.564e-13 ***
## treatment2:pair 10.6388 0.12665    84 489.00  1.4051  0.015578 *
## ---
## Signif. codes:  0 '***' 0.001 '**' 0.01 '*' 0.05 '.' 0.1 ' ' 1
```

```r
summary(fit2)
```

```
## Linear mixed model fit by REML t-tests use Satterthwaite approximations
##   to degrees of freedom [lmerMod]
## Formula: relfitav ~ treatment2 * pair + (1 | strain) + (1 | plate)
##    Data: datac
##
## REML criterion at convergence: 410.4
##
## Scaled residuals:
##     Min      1Q  Median      3Q     Max
## -2.2199 -0.5859 -0.0813  0.4741  5.5769
##
## Random effects:
##  Groups   Name        Variance Std.Dev.
##  plate    (Intercept) 0.002661 0.05159
##  strain   (Intercept) 0.000496 0.02227
##  Residual             0.090136 0.30023
## Number of obs: 609, groups:  plate, 21; strain, 2
##
## Fixed effects:
##                       Estimate Std. Error         df  t value Pr(>|t|)
## (Intercept)           0.942267   0.127113 319.700000   7.413 1.12e-12 ***
## treatment21          -0.076819   0.178380 381.100000  -0.431  0.66697
## treatment22          -0.122317   0.178380 381.100000  -0.686  0.49331
## treatment23          -0.358136   0.178380 381.100000  -2.008  0.04538 *
## treatment24          -0.335925   0.178380 381.100000  -1.883  0.06044 .
## treatment25          -0.339334   0.178380 381.100000  -1.902  0.05789 .
## treatment26          -0.532587   0.178380 381.100000  -2.986  0.00301 **
## pair2                -0.118064   0.173336 489.000000  -0.681  0.49611
## pair3                -0.118802   0.173336 489.000000  -0.685  0.49343
## pair4                 0.051883   0.173336 489.000000   0.299  0.76482
## pair5                -0.155446   0.173336 489.000000  -0.897  0.37027
## pair6                -0.099273   0.173336 489.000000  -0.573  0.56710
## pair7                -0.044838   0.173336 489.000000  -0.259  0.79599
## pair8                 0.167418   0.212516 489.900000   0.788  0.43120
## pair9                -0.190145   0.173336 489.000000  -1.097  0.27319
```

```
## pair10               0.081711   0.173336 489.000000   0.471  0.63756
## pair11              -0.130679   0.173336 489.000000  -0.754  0.45127
## pair12              -0.328334   0.173336 489.000000  -1.894  0.05879 .
## pair13              -0.052235   0.173336 489.000000  -0.301  0.76327
## pair14               0.100735   0.173336 489.000000   0.581  0.56141
## pair15              -0.108843   0.173336 489.000000  -0.628  0.53034
## treatment21:pair2   -0.028621   0.245135 489.000000  -0.117  0.90710
## treatment22:pair2    0.340973   0.245135 489.000000   1.391  0.16487
## treatment23:pair2    0.221559   0.245135 489.000000   0.904  0.36653
## treatment24:pair2    0.270735   0.245135 489.000000   1.104  0.26995
## treatment25:pair2    0.028680   0.245135 489.000000   0.117  0.90691
## treatment26:pair2    0.180027   0.245135 489.000000   0.734  0.46306
## treatment21:pair3    0.217382   0.245135 489.000000   0.887  0.37563
## treatment22:pair3    0.226621   0.245135 489.000000   0.924  0.35569
## treatment23:pair3    0.319145   0.245135 489.000000   1.302  0.19356
## treatment24:pair3    0.293246   0.245135 489.000000   1.196  0.23217
## treatment25:pair3    0.380300   0.245135 489.000000   1.551  0.12145
## treatment26:pair3    0.333608   0.245135 489.000000   1.361  0.17417
## treatment21:pair4   -0.045441   0.245135 489.000000  -0.185  0.85301
## treatment22:pair4   -0.191122   0.245135 489.000000  -0.780  0.43597
## treatment23:pair4   -0.065320   0.245135 489.000000  -0.266  0.78999
## treatment24:pair4    0.154234   0.245135 489.000000   0.629  0.52952
## treatment25:pair4    0.275432   0.245135 489.000000   1.124  0.26174
## treatment26:pair4   -0.108063   0.245135 489.000000  -0.441  0.65953
## treatment21:pair5    0.289160   0.245135 489.000000   1.180  0.23873
## treatment22:pair5    0.326434   0.245135 489.000000   1.332  0.18359
## treatment23:pair5    0.355424   0.245135 489.000000   1.450  0.14772
## treatment24:pair5    0.278770   0.245135 489.000000   1.137  0.25601
## treatment25:pair5    0.122940   0.245135 489.000000   0.502  0.61623
## treatment26:pair5    0.312713   0.245135 489.000000   1.276  0.20267
## treatment21:pair6   -0.048765   0.245135 489.000000  -0.199  0.84240
## treatment22:pair6    0.141721   0.245135 489.000000   0.578  0.56344
## treatment23:pair6    0.300922   0.245135 489.000000   1.228  0.22020
## treatment24:pair6    0.259550   0.245135 489.000000   1.059  0.29021
## treatment25:pair6    0.137416   0.245135 489.000000   0.561  0.57534
## treatment26:pair6    0.640715   0.245135 489.000000   2.614  0.00923 **
## treatment21:pair7    0.091308   0.245135 489.000000   0.372  0.70969
## treatment22:pair7    0.294869   0.245135 489.000000   1.203  0.22960
## treatment23:pair7    0.467459   0.245135 489.000000   1.907  0.05711 .
## treatment24:pair7    0.386827   0.245135 489.000000   1.578  0.11521
## treatment25:pair7    0.184691   0.245135 489.000000   0.753  0.45156
## treatment26:pair7    0.229151   0.245135 489.000000   0.935  0.35035
## treatment21:pair8   -0.009692   0.300227 489.000000  -0.032  0.97426
## treatment22:pair8    0.219867   0.300227 489.000000   0.732  0.46432
## treatment23:pair8   -0.209223   0.300227 489.000000  -0.697  0.48621
## treatment24:pair8    0.471723   0.300227 489.000000   1.571  0.11678
## treatment25:pair8    0.751426   0.300227 489.000000   2.503  0.01264 *
## treatment26:pair8    0.328955   0.300227 489.000000   1.096  0.27376
## treatment21:pair9    0.100546   0.245135 489.000000   0.410  0.68186
## treatment22:pair9    0.081226   0.245135 489.000000   0.331  0.74052
## treatment23:pair9    0.021323   0.245135 489.000000   0.087  0.93072
## treatment24:pair9    0.312044   0.245135 489.000000   1.273  0.20364
## treatment25:pair9    0.099562   0.245135 489.000000   0.406  0.68481
## treatment26:pair9    0.223391   0.245135 489.000000   0.911  0.36259
```

```
## treatment21:pair10   0.105117   0.245135 489.000000   0.429  0.66825
## treatment22:pair10  -0.083721   0.245135 489.000000  -0.342  0.73285
## treatment23:pair10   0.420810   0.245135 489.000000   1.717  0.08668 .
## treatment24:pair10   0.077403   0.245135 489.000000   0.316  0.75232
## treatment25:pair10   0.512049   0.245135 489.000000   2.089  0.03724 *
## treatment26:pair10   0.045267   0.245135 489.000000   0.185  0.85357
## treatment21:pair11   0.413701   0.245135 489.000000   1.688  0.09212 .
## treatment22:pair11   0.069595   0.245135 489.000000   0.284  0.77660
## treatment23:pair11   0.370262   0.245135 489.000000   1.510  0.13158
## treatment24:pair11   0.265672   0.245135 489.000000   1.084  0.27900
## treatment25:pair11   0.244309   0.245135 489.000000   0.997  0.31944
## treatment26:pair11   0.664920   0.245135 489.000000   2.712  0.00691 **
## treatment21:pair12   0.100065   0.245135 489.000000   0.408  0.68330
## treatment22:pair12   0.241583   0.245135 489.000000   0.986  0.32486
## treatment23:pair12   0.395678   0.245135 489.000000   1.614  0.10715
## treatment24:pair12   0.254885   0.245135 489.000000   1.040  0.29896
## treatment25:pair12   0.277419   0.245135 489.000000   1.132  0.25832
## treatment26:pair12   0.338271   0.245135 489.000000   1.380  0.16824
## treatment21:pair13   0.212236   0.245135 489.000000   0.866  0.38703
## treatment22:pair13   0.121642   0.245135 489.000000   0.496  0.61996
## treatment23:pair13   0.266070   0.245135 489.000000   1.085  0.27828
## treatment24:pair13   0.470178   0.245135 489.000000   1.918  0.05569 .
## treatment25:pair13   0.370367   0.245135 489.000000   1.511  0.13147
## treatment26:pair13   0.601140   0.245135 489.000000   2.452  0.01454 *
## treatment21:pair14   0.014203   0.245135 489.000000   0.058  0.95382
## treatment22:pair14   0.121881   0.245135 489.000000   0.497  0.61927
## treatment23:pair14   0.267452   0.245135 489.000000   1.091  0.27579
## treatment24:pair14   0.248048   0.245135 489.000000   1.012  0.31209
## treatment25:pair14   0.090578   0.245135 489.000000   0.370  0.71191
## treatment26:pair14   0.073102   0.245135 489.000000   0.298  0.76567
## treatment21:pair15   0.202813   0.245135 489.000000   0.827  0.40844
## treatment22:pair15   0.235988   0.245135 489.000000   0.963  0.33618
## treatment23:pair15   0.291326   0.245135 489.000000   1.188  0.23524
## treatment24:pair15   0.806292   0.245135 489.000000   3.289  0.00108 **
## treatment25:pair15   0.584320   0.245135 489.000000   2.384  0.01752 *
## treatment26:pair15   0.337153   0.245135 489.000000   1.375  0.16964
## ---
## Signif. codes:  0 '***' 0.001 '**' 0.01 '*' 0.05 '.' 0.1 ' ' 1

##
## Correlation matrix not shown by default, as p = 105 > 20.
## Use print(x, correlation=TRUE)  or
##   vcov(x)     if you need it
```

Phosphate

```r
data1<-read.table("~/Desktop/rsync/deteriorating treatments/phosphate/2344_phosphate_v2_[0].csv",h=T)
data2<-read.table("~/Desktop/rsync/deteriorating treatments/phosphate/2931_phosphate_v2_[0].csv",h=T)
datac<-rbind(data1,data2)
datac$treatment2<-as.factor(datac$treatment)
datac$pair<-as.factor(datac$pair)

fit2<-lmer(relfitav~treatment2*pair+(1|strain)+(1|plate),datac)
anova(fit2)
```

```
## Analysis of Variance Table of type III  with  Satterthwaite
## approximation for degrees of freedom
##                 Sum Sq  Mean Sq NumDF DenDF F.value   Pr(>F)
## treatment2      0.4199 0.069982     6   504 0.91043 0.486947
## pair            3.2123 0.229447    14   504 2.98498 0.000204 ***
## treatment2:pair 5.0172 0.059728    84   504 0.77703 0.923227
## ---
## Signif. codes:  0 '***' 0.001 '**' 0.01 '*' 0.05 '.' 0.1 ' ' 1
```

```r
summary(fit2)
```

```
## Linear mixed model fit by REML t-tests use Satterthwaite approximations
##   to degrees of freedom [lmerMod]
## Formula: relfitav ~ treatment2 * pair + (1 | strain) + (1 | plate)
##    Data: datac
##
## REML criterion at convergence: 320.5
##
## Scaled residuals:
##     Min      1Q  Median      3Q     Max
## -2.1702 -0.5579 -0.0057  0.5367  5.4102
##
## Random effects:
##  Groups   Name        Variance  Std.Dev.
##  plate    (Intercept) 2.789e-16 1.670e-08
##  strain   (Intercept) 8.782e-18 2.963e-09
##  Residual             7.687e-02 2.772e-01
## Number of obs: 609, groups:  plate, 21; strain, 2
##
## Fixed effects:
##                      Estimate Std. Error         df t value Pr(>|t|)
## (Intercept)         8.789e-01  1.132e-01  5.040e+02   7.765 4.57e-14 ***
## treatment21        -1.569e-01  1.601e-01  5.040e+02  -0.980   0.3275
## treatment22        -1.440e-01  1.601e-01  5.040e+02  -0.900   0.3688
## treatment23        -5.321e-02  1.601e-01  5.040e+02  -0.332   0.7397
## treatment24        -4.931e-02  1.601e-01  5.040e+02  -0.308   0.7582
## treatment25        -5.241e-02  1.601e-01  5.040e+02  -0.327   0.7435
## treatment26         3.310e-02  1.601e-01  5.040e+02   0.207   0.8363
## pair2              -9.432e-02  1.601e-01  5.040e+02  -0.589   0.5559
## pair3              -4.611e-02  1.601e-01  5.040e+02  -0.288   0.7734
## pair4              -7.227e-02  1.601e-01  5.040e+02  -0.451   0.6518
## pair5               7.018e-02  1.601e-01  5.040e+02   0.438   0.6612
## pair6               1.639e-01  1.601e-01  5.040e+02   1.024   0.3064
## pair7               1.488e-01  1.601e-01  5.040e+02   0.929   0.3531
## pair8               2.486e-01  1.960e-01  5.040e+02   1.268   0.2054
## pair9              -5.422e-02  1.601e-01  5.040e+02  -0.339   0.7350
## pair10             -1.336e-02  1.601e-01  5.040e+02  -0.083   0.9335
## pair11              3.860e-03  1.601e-01  5.040e+02   0.024   0.9808
## pair12             -2.581e-01  1.601e-01  5.040e+02  -1.612   0.1075
## pair13              1.108e-01  1.601e-01  5.040e+02   0.692   0.4890
## pair14              2.186e-01  1.601e-01  5.040e+02   1.366   0.1727
## pair15              2.822e-02  1.601e-01  5.040e+02   0.176   0.8602
## treatment21:pair2   3.093e-01  2.264e-01  5.040e+02   1.366   0.1725
## treatment22:pair2   4.240e-01  2.264e-01  5.040e+02   1.873   0.0617 .
```

```
## treatment23:pair2   3.020e-01  2.264e-01  5.040e+02   1.334   0.1828
## treatment24:pair2   2.177e-01  2.264e-01  5.040e+02   0.961   0.3368
## treatment25:pair2   2.247e-01  2.264e-01  5.040e+02   0.992   0.3214
## treatment26:pair2  -6.442e-03  2.264e-01  5.040e+02  -0.028   0.9773
## treatment21:pair3   1.994e-01  2.264e-01  5.040e+02   0.881   0.3789
## treatment22:pair3   1.153e-01  2.264e-01  5.040e+02   0.509   0.6108
## treatment23:pair3   5.699e-02  2.264e-01  5.040e+02   0.252   0.8013
## treatment24:pair3   1.228e-01  2.264e-01  5.040e+02   0.543   0.5877
## treatment25:pair3   4.789e-01  2.264e-01  5.040e+02   2.116   0.0349 *
## treatment26:pair3  -1.167e-02  2.264e-01  5.040e+02  -0.052   0.9589
## treatment21:pair4   1.846e-01  2.264e-01  5.040e+02   0.815   0.4152
## treatment22:pair4   3.614e-01  2.264e-01  5.040e+02   1.596   0.1110
## treatment23:pair4  -2.722e-03  2.264e-01  5.040e+02  -0.012   0.9904
## treatment24:pair4   1.835e-02  2.264e-01  5.040e+02   0.081   0.9354
## treatment25:pair4   9.907e-02  2.264e-01  5.040e+02   0.438   0.6618
## treatment26:pair4  -2.307e-02  2.264e-01  5.040e+02  -0.102   0.9189
## treatment21:pair5   1.320e-01  2.264e-01  5.040e+02   0.583   0.5600
## treatment22:pair5   1.938e-01  2.264e-01  5.040e+02   0.856   0.3923
## treatment23:pair5   2.669e-02  2.264e-01  5.040e+02   0.118   0.9062
## treatment24:pair5   8.318e-02  2.264e-01  5.040e+02   0.367   0.7134
## treatment25:pair5  -6.301e-02  2.264e-01  5.040e+02  -0.278   0.7809
## treatment26:pair5   4.983e-03  2.264e-01  5.040e+02   0.022   0.9824
## treatment21:pair6   3.932e-02  2.264e-01  5.040e+02   0.174   0.8622
## treatment22:pair6   7.869e-03  2.264e-01  5.040e+02   0.035   0.9723
## treatment23:pair6   4.805e-03  2.264e-01  5.040e+02   0.021   0.9831
## treatment24:pair6  -6.186e-02  2.264e-01  5.040e+02  -0.273   0.7848
## treatment25:pair6   5.947e-04  2.264e-01  5.040e+02   0.003   0.9979
## treatment26:pair6  -2.738e-01  2.264e-01  5.040e+02  -1.210   0.2270
## treatment21:pair7   7.729e-02  2.264e-01  5.040e+02   0.341   0.7329
## treatment22:pair7   2.149e-01  2.264e-01  5.040e+02   0.949   0.3430
## treatment23:pair7  -5.129e-02  2.264e-01  5.040e+02  -0.227   0.8209
## treatment24:pair7   1.598e-02  2.264e-01  5.040e+02   0.071   0.9437
## treatment25:pair7   1.276e-01  2.264e-01  5.040e+02   0.564   0.5732
## treatment26:pair7  -3.142e-02  2.264e-01  5.040e+02  -0.139   0.8897
## treatment21:pair8   1.504e-01  2.772e-01  5.040e+02   0.542   0.5878
## treatment22:pair8  -8.348e-03  2.772e-01  5.040e+02  -0.030   0.9760
## treatment23:pair8  -7.175e-02  2.772e-01  5.040e+02  -0.259   0.7959
## treatment24:pair8  -2.514e-02  2.772e-01  5.040e+02  -0.091   0.9278
## treatment25:pair8  -3.114e-01  2.772e-01  5.040e+02  -1.123   0.2619
## treatment26:pair8  -1.791e-01  2.772e-01  5.040e+02  -0.646   0.5185
## treatment21:pair9   2.541e-01  2.264e-01  5.040e+02   1.123   0.2621
## treatment22:pair9   7.883e-03  2.264e-01  5.040e+02   0.035   0.9722
## treatment23:pair9  -2.740e-02  2.264e-01  5.040e+02  -0.121   0.9037
## treatment24:pair9   1.685e-01  2.264e-01  5.040e+02   0.744   0.4570
## treatment25:pair9   2.473e-01  2.264e-01  5.040e+02   1.092   0.2752
## treatment26:pair9  -3.413e-02  2.264e-01  5.040e+02  -0.151   0.8802
## treatment21:pair10  2.139e-01  2.264e-01  5.040e+02   0.945   0.3451
## treatment22:pair10  6.049e-02  2.264e-01  5.040e+02   0.267   0.7894
## treatment23:pair10  2.025e-01  2.264e-01  5.040e+02   0.895   0.3715
## treatment24:pair10  6.314e-02  2.264e-01  5.040e+02   0.279   0.7804
## treatment25:pair10  4.172e-02  2.264e-01  5.040e+02   0.184   0.8539
## treatment26:pair10 -1.045e-01  2.264e-01  5.040e+02  -0.462   0.6445
## treatment21:pair11  1.362e-01  2.264e-01  5.040e+02   0.602   0.5477
## treatment22:pair11  7.955e-02  2.264e-01  5.040e+02   0.351   0.7254
```

```
## treatment23:pair11 -1.254e-02  2.264e-01  5.040e+02  -0.055   0.9559
## treatment24:pair11  1.872e-02  2.264e-01  5.040e+02   0.083   0.9341
## treatment25:pair11 -1.343e-01  2.264e-01  5.040e+02  -0.593   0.5534
## treatment26:pair11 -2.494e-01  2.264e-01  5.040e+02  -1.102   0.2711
## treatment21:pair12  3.041e-01  2.264e-01  5.040e+02   1.343   0.1798
## treatment22:pair12  3.505e-01  2.264e-01  5.040e+02   1.548   0.1222
## treatment23:pair12  1.381e-01  2.264e-01  5.040e+02   0.610   0.5420
## treatment24:pair12  2.257e-01  2.264e-01  5.040e+02   0.997   0.3193
## treatment25:pair12  1.673e-01  2.264e-01  5.040e+02   0.739   0.4603
## treatment26:pair12  8.197e-02  2.264e-01  5.040e+02   0.362   0.7174
## treatment21:pair13  1.034e-01  2.264e-01  5.040e+02   0.457   0.6481
## treatment22:pair13  2.044e-01  2.264e-01  5.040e+02   0.903   0.3671
## treatment23:pair13  9.219e-02  2.264e-01  5.040e+02   0.407   0.6840
## treatment24:pair13  3.211e-02  2.264e-01  5.040e+02   0.142   0.8872
## treatment25:pair13 -9.085e-02  2.264e-01  5.040e+02  -0.401   0.6883
## treatment26:pair13 -1.030e-01  2.264e-01  5.040e+02  -0.455   0.6492
## treatment21:pair14 -4.294e-02  2.264e-01  5.040e+02  -0.190   0.8496
## treatment22:pair14 -4.130e-02  2.264e-01  5.040e+02  -0.182   0.8553
## treatment23:pair14 -1.240e-01  2.264e-01  5.040e+02  -0.548   0.5842
## treatment24:pair14 -1.130e-01  2.264e-01  5.040e+02  -0.499   0.6179
## treatment25:pair14 -2.483e-01  2.264e-01  5.040e+02  -1.097   0.2733
## treatment26:pair14 -5.600e-01  2.264e-01  5.040e+02  -2.474   0.0137 *
## treatment21:pair15  5.129e-02  2.264e-01  5.040e+02   0.227   0.8208
## treatment22:pair15  2.809e-01  2.264e-01  5.040e+02   1.241   0.2152
## treatment23:pair15  1.858e-01  2.264e-01  5.040e+02   0.821   0.4122
## treatment24:pair15 -2.778e-02  2.264e-01  5.040e+02  -0.123   0.9024
## treatment25:pair15 -1.839e-01  2.264e-01  5.040e+02  -0.812   0.4169
## treatment26:pair15 -1.609e-01  2.264e-01  5.040e+02  -0.711   0.4777
## ---
## Signif. codes:  0 '***' 0.001 '**' 0.01 '*' 0.05 '.' 0.1 ' ' 1
```

```
##
## Correlation matrix not shown by default, as p = 105 > 20.
## Use print(x, correlation=TRUE)  or
##   vcov(x)     if you need it
```

Does genetic variance for relative fitness depend on environment?

Relative fitness, based on 0 growth, avanc, scaled by generation time.

```
data<-read.table("~/Desktop/rsync/deteriorating treatments/varG_v2_[0].csv",h=T)
data$env<-as.factor(data$env)
fit<-lmer(varG~env+(1|strain)+(1|stress),data)
anova(fit)
```

```
## Analysis of Variance Table of type III  with  Satterthwaite
## approximation for degrees of freedom
##        Sum Sq    Mean Sq NumDF  DenDF F.value Pr(>F)
## env 0.0020405 0.00034008     6 19.001  1.1828 0.3566
```

```
summary(fit)
```

```
## Linear mixed model fit by REML t-tests use Satterthwaite approximations
##   to degrees of freedom [lmerMod]
## Formula: varG ~ env + (1 | strain) + (1 | stress)
##    Data: data
##
## REML criterion at convergence: -99.1
##
## Scaled residuals:
##     Min      1Q  Median      3Q     Max
## -1.1907 -0.6568 -0.1029  0.4881  2.0704
##
## Random effects:
##  Groups   Name        Variance  Std.Dev.
##  strain   (Intercept) 4.596e-05 0.006780
##  stress   (Intercept) 8.788e-05 0.009375
##  Residual             2.875e-04 0.016956
## Number of obs: 28, groups:  strain, 2; stress, 2
##
## Fixed effects:
##              Estimate Std. Error        df t value Pr(>|t|)
## (Intercept)  0.008664   0.011781  4.606000   0.735   0.4978
## env1        -0.001306   0.011990 19.001000  -0.109   0.9144
## env2         0.008844   0.011990 19.001000   0.738   0.4698
## env3         0.010076   0.011990 19.001000   0.840   0.4111
## env4         0.011035   0.011990 19.001000   0.920   0.3689
## env5         0.026346   0.011990 19.001000   2.197   0.0406 *
## env6         0.013598   0.011990 19.001000   1.134   0.2708
## ---
## Signif. codes:  0 '***' 0.001 '**' 0.01 '*' 0.05 '.' 0.1 ' ' 1
##
## Correlation of Fixed Effects:
##      (Intr) env1   env2   env3   env4   env5
## env1 -0.509
## env2 -0.509  0.500
## env3 -0.509  0.500  0.500
## env4 -0.509  0.500  0.500  0.500
## env5 -0.509  0.500  0.500  0.500  0.500
## env6 -0.509  0.500  0.500  0.500  0.500  0.500
```

```r
fit<-lmer(var~env+(1|strain)+(1|stress),data)
anova(fit)
```

```
## Analysis of Variance Table of type III  with  Satterthwaite
## approximation for degrees of freedom
##        Sum Sq    Mean Sq NumDF  DenDF F.value Pr(>F)
## env 0.0020416 0.00034026     6 19.001  1.1831 0.3565
```

```r
summary(fit)
```

```
## Linear mixed model fit by REML t-tests use Satterthwaite approximations
##   to degrees of freedom [lmerMod]
## Formula: var ~ env + (1 | strain) + (1 | stress)
##    Data: data
```

```
##
## REML criterion at convergence: -99.1
##
## Scaled residuals:
##     Min      1Q  Median      3Q     Max
## -1.1908 -0.6566 -0.1031  0.4878  2.0708
##
## Random effects:
##  Groups   Name        Variance  Std.Dev.
##  strain   (Intercept) 4.597e-05 0.006780
##  stress   (Intercept) 8.787e-05 0.009374
##  Residual             2.876e-04 0.016959
## Number of obs: 28, groups:  strain, 2; stress, 2
##
## Fixed effects:
##              Estimate Std. Error        df t value Pr(>|t|)
## (Intercept)  0.008665   0.011782  4.607000   0.735   0.4978
## env1        -0.001308   0.011992 19.001000  -0.109   0.9143
## env2         0.008839   0.011992 19.001000   0.737   0.4701
## env3         0.010074   0.011992 19.001000   0.840   0.4113
## env4         0.011036   0.011992 19.001000   0.920   0.3690
## env5         0.026353   0.011992 19.001000   2.198   0.0406 *
## env6         0.013599   0.011992 19.001000   1.134   0.2709
## ---
## Signif. codes:  0 '***' 0.001 '**' 0.01 '*' 0.05 '.' 0.1 ' ' 1
##
## Correlation of Fixed Effects:
##      (Intr) env1   env2   env3   env4   env5
## env1 -0.509
## env2 -0.509  0.500
## env3 -0.509  0.500  0.500
## env4 -0.509  0.500  0.500  0.500
## env5 -0.509  0.500  0.500  0.500  0.500
## env6 -0.509  0.500  0.500  0.500  0.500  0.500
```

```r
fit<-lmer(evolv~env+(1|strain)+(1|stress),data)
anova(fit)
```

```
## Analysis of Variance Table of type III  with  Satterthwaite
## approximation for degrees of freedom
##         Sum Sq   Mean Sq NumDF  DenDF F.value Pr(>F)
## env 1.5828e-07 2.638e-08     6 25.158 0.43061 0.8515
```

```r
summary(fit)
```

```
## Linear mixed model fit by REML t-tests use Satterthwaite approximations
##   to degrees of freedom [lmerMod]
## Formula: evolv ~ env + (1 | strain) + (1 | stress)
##    Data: data
##
## REML criterion at convergence: -225.7
##
## Scaled residuals:
```

```
##      Min       1Q   Median       3Q      Max
## -1.76354 -0.63532 -0.00997  0.54727  1.52384
##
## Random effects:
##  Groups   Name        Variance  Std.Dev.
##  strain   (Intercept) 0.000e+00 0.0000000
##  stress   (Intercept) 0.000e+00 0.0000000
##  Residual             6.126e-08 0.0002475
## Number of obs: 24, groups:  strain, 2; stress, 2
##
## Fixed effects:
##               Estimate Std. Error         df  t value Pr(>|t|)
## (Intercept)  1.000e+00  1.238e-04  2.516e+01 8080.471   <2e-16 ***
## env1        -1.616e-04  2.144e-04  2.516e+01   -0.754    0.458
## env2        -4.588e-05  1.750e-04  2.516e+01   -0.262    0.795
## env3        -1.074e-04  1.750e-04  2.516e+01   -0.614    0.545
## env4         1.951e-05  1.890e-04  2.516e+01    0.103    0.919
## env5         9.440e-05  1.750e-04  2.516e+01    0.539    0.594
## env6        -1.245e-04  1.890e-04  2.516e+01   -0.659    0.516
## ---
## Signif. codes:  0 '***' 0.001 '**' 0.01 '*' 0.05 '.' 0.1 ' ' 1
##
## Correlation of Fixed Effects:
##      (Intr) env1   env2   env3   env4   env5
## env1 -0.577
## env2 -0.707  0.408
## env3 -0.707  0.408  0.500
## env4 -0.655  0.378  0.463  0.463
## env5 -0.707  0.408  0.500  0.500  0.463
## env6 -0.655  0.378  0.463  0.463  0.429  0.463
```
